# Anchoring Gold Nanoparticles on Functionalized Halloysite Nanotubes: Density Functional Theory and Experimental Studies

**DOI:** 10.1021/acs.jpcc.5c05165

**Published:** 2025-09-09

**Authors:** Ludovico Guercio, Francesco Ferrante, Marco Bertini, Chiara Ferlito, Lorenzo Lisuzzo, Giuseppe Lazzara, Dario Duca

**Affiliations:** Dipartimento di Fisica e Chimica “Emilio Segrè”, 325565 Università degli Studi di Palermo, Viale delle Scienze Ed. 17, 90128 Palermo, Italy

## Abstract

The structural and catalytic properties of gold nanoparticles are known to be highly sensitive to cluster size, dimensionality, and interactions with the support. In this work, a combined theoretical and experimental approach was employed to investigate systems in which gold nanoclusters are anchored on functionalized halloysite nanotubes. Density functional theory calculations were performed to explore the geometric and electronic characteristics of the anchored Au_*n*_ (*n* = 1 – 20) clusters and their interactions with the amino-functionalized support. Multiple anchoring configurations were assessed, with optimized geometries and Au–N interaction distances analyzed in detail. Results indicate a transition from a two-dimensional to a three-dimensional cluster structure occurring at a lower number of atoms if compared with the case of isolated Au_*n*_ clusters, mainly due to the overall interaction with the halloysite silanolic groups. Complementarily, gold nanoparticles were synthesized and deposited onto functionalized HNTs, and the resulting materials were characterized using experimental techniques. The study demonstrates that halloysite-based hybrid materials offer a promising platform for stabilizing small gold clusters, with potential application as heterogeneous catalysts.

## Graphical abstract

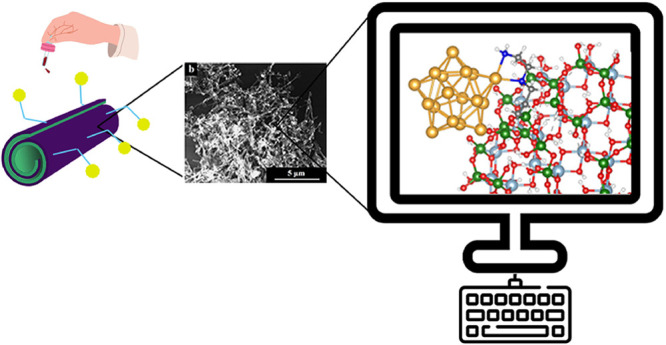

## Introduction

Extensive efforts have been dedicated to investigating the key factors that influence the catalytic performance of noble metal clusters. These include the size, composition, and phase of the clusters, the coordination and valence of the active metal components, as well as facets, terminations, defects, and surface area of the supporting materials.^1^ It seems that all of these variables can affect both the electronic properties and the geometric configuration of a cluster catalyst, leading to significant changes in its overall reactivity and selectivity. By altering structural and electronic characteristics, the catalyst's ability to interact with reactants and to facilitate chemical reactions is ultimately transformed, resulting in variations in its performance and efficiency.^2–4^ It was demonstrated that metal species of varying sizes (i.e., ranging from single atoms to nanoclusters and nanoparticles) exhibit different catalytic behaviors across various heterogeneous catalytic reactions. The differences in size directly influence the catalytic properties, leading to different reaction mechanisms.^5^ Since their crucial role in catalytic applications was discovered, particularly when supported on activated carbon or other reducible materials, the study of catalysts based on metal nanoparticles has been rapidly expanding.^6^ Small metal nanoparticles, ranging from 1 to 10 nm, demonstrate remarkable catalytic activity, occasionally surpassing that of their corresponding metal complexes. The high efficiency of nanocatalysts is attributed to various key factors, encompassing the high surface-to-volume ratio, surface geometric effects (such as surface atom arrangement and low-coordinated atoms), electronic, and quantum size effects.^7^ Further, due to the advantages lying in the simple isolation of the product and facilitated recyclability of the catalyst,^8^ there is an increasing use of metal nanoparticles suspended in solution.

Due to their remarkable properties, i.e., fluorescence, catalytic activity, and biocompatibility, gold nanoparticles and nanoclusters (which will be denoted collectively as AuNPs), especially those having a diameter close to 2 nm, can be used in various fields, starting from imaging and therapy in biomedical applications up to catalysis.^9–12^ Though gold is dubbed as a relatively unreactive metal, AuNPs, featuring high specific surface area and numerous unsaturated sites, can show high catalytic activity and exceptional selectivity in reactions such as oxidation, hydrogenation, and C–C coupling.^13^ The focusing on gold particles highlights a broader scientific interest, and the experimental and theoretical exploration of metallic and ionic clusters has proven to be crucial for analyzing the effects of their electronic, optical, and magnetic properties.^14–17^

The choice of the preparation method proved to be extremely important for the formation of high-performance gold catalysts.^18^ Various approaches are available for synthesizing gold nanoparticles, including chemical, biological, physical, and hybrid techniques.^19–21^ Each of them has specific advantages and drawbacks. Among the most commonly employed techniques, the deposition-precipitation, coprecipitation, and reduction-deposition methods can be found.^22,23^ The production of gold nanoparticles started in 1951, when Turkevich^24^ introduced the use of citric acid as a stabilizing agent. This pioneering approach gained widespread acceptance and, in 1973, was modified by Frens^25^ to obtain AuNPs with diameters ranging from 15 to 150 nm.

It is a matter of fact that heterogeneous catalysts often provide high stability and can operate under a wide range of temperatures and pressures, making them suitable for large-scale, continuous industrial processes.^26^ Another key advantage is that heterogeneous catalysts can be easily regenerated through processes like combustion, pyrolysis, or thermal treatments.^27^ The use of nanoparticles deposited on a sustainable support could represent an auspicable alternative to many industrial catalysts, and, as supports, clay-based materials could represent a valid candidate.^28–30^ Clay minerals are naturally abundant, cost-effective materials with a heterogeneous nature, with great thermal and chemical stability; further, they have no harmful impact on the environment when used in chemical reactions.^31,32^ In particular, due to their natural origin and low toxicity, halloysite nanotubes (HNTs) offer versatility for various applications by allowing selective functionalization based on specific requirements.^33–35^ The typical dimensions, polydispersity, and mineral purity of HNTs depend on their specific geological origin, leading to variations in length from the submicron scale to 2–3 μm,^36^ outer diameter from 15 to 200 nm, and inner diameter from 10 to 100 nm.^37–39^ These nanoclays exhibit a hollow tubular structure with aluminosilicate layers arranged in a spiral-like morphology.^40,41^ The unique chemical properties of halloysite are due to the differences between the inner and outer surfaces. Indeed, the external surface is negatively charged due to a tetrahedral SiO_4_-based sheet overlapped with an octahedral AlO_3_(OH)_3_ sheet,^42,43^ which, in contrast, shows a positively charged surface. This charge separation is maintained within the pH range of 2–8.^44,45^ The distinctive tubular structure of halloysite, together with its unique chemical environment separation, allows for precise and selective modifications.^46^ Functionalization of the positively charged inner surface can be achieved using molecules containing nucleophilic groups, while modification of the outer surface with positive species, such as polymers, surfactants, and biopolymers, can be accomplished through electrostatic interactions.^47^ It is also worth noting that the geological nature of this system causes it to show, in addition to its well-known variability regarding the geometric characteristics (length, diameter, number of windings), a large number of defects, irregularities, variable structural patterns, and composition. For instance, recently it was shown that, likely due to exposure of the underlying alumina layer, HNTs with length in the micrometers regime have positively charged regions on the ends and side surfaces, which contribute to their self-assembly.^48^

In this work, halloysite nanotubes were investigated as supports for decoration with gold from both computational and experimental perspectives. The anchoring of Au clusters ranging from 1 to 20 atoms onto the external surface of HNTs functionalized with organosilanes (fHNT) was studied by means of density functional theory (DFT), focusing on both the geometries of the clusters and the interactions between the gold centers and the amino-termination of *N*-[3-(trimethoxysilyl)­propyl]­ethylenediamine (AEAPTMS). Thermodynamics features regarding cluster growth are also discussed. Also, AuNPs supported on functionalized halloysite were experimentally synthesized and characterized.

It is worth noting that the proposed Au-halloysite composite system could be explored as a catalyst in the oxidation of 5-hydroximethylfurfural (HMF) to 2,5-furandicarboxylic acid, as a main route for the valorization of biomass and the production of added-value chemicals.^32^ In particular, the first part of the said process, the dehydration of fructose to HMF, is notoriously conducted in an acidic environment, which could be represented by the inner surface of the halloysite nanotube; the following step, the oxidation of HMF to furandicarboxylic acid, requires a basic environment, present on the outer surface of HNT, and a metal catalyst. For this reason, even if in principle the internal lumen of the nanotube could also be modified by appropriate chemical species, only the functionalization of the silicic surface, and the subsequent AuNP anchoring, was considered for the present investigation.

## Materials and Methods

### Materials

Halloysite nanotubes (HNTs) are a gift from I-Minerals, Inc., mined in the geological deposit of Latah County, with physicochemical properties detailed elsewhere.^49^ Gold­(III) chloride trihydrate (HAuCl_4_·3H_2_O, Mw = 393.83 g mol^–1^, purity ≥ 99.9%), *N*-[3-(trimethoxysilyl)­propyl]­ethylenediamine ((CH_3_O)_3_Si­(CH_2_)_3_NHCH_2_CH_2_NH_2_, Mw = 222.357 g mol^–1^, 97%), toluene (anhydrous, 99.8%), and trisodium citrate dihydrate (Na-CA, C_6_H_5_Na_3_O_7_·2H_2_O, Mw = 294.10 g mol^–1^, >99%) were purchased from Sigma-Aldrich. Absolute ethanol (>99.8%) is a Honeywell product.

### Functionalization of Halloysite with AEAPTMS

HNTs functionalization with *N*-[3-(trimethoxysilyl)­propyl]­ethylenediamine was conducted by using the standard procedure reported in the literature.^50^ Briefly, 50 cm^3^ of AEAPTMS 0.4 M solution in toluene were prepared, and 0.5 g HNTs were added to the reaction flask and kept under stirring for 16 h at room temperature. The dispersion was then separated by centrifugation (5000 rpm, 5 min), and the functionalized nanoclay was washed three times with ethanol and dried overnight.

### Synthesis of Gold Nanoparticles

The colloidal dispersion of citrate-stabilized gold nanoparticles was prepared by chemical reduction of HAuCl_4_ according to the Turkevich method refined by Frens.^24,25^ In particular, 70 cm^3^ of a HAuCl_4_ aqueous solution (0.01 %_wt_) in deionized water was prepared and heated until boiling. Hence, 0.84 cm^3^ of Na-CA 0.4 M solution was added. Upon boiling for 5 min, a color change from blue to red was observed, indicating the formation of AuNPs. After cooling the dispersion at room temperature, 0.04 mmol of solid Na-CA was added to further stabilize the nanoparticles.

### Loading of AuNPs onto HNTs

The last step of the preparation protocol involved the loading of AuNPs on the surface of the aminoalkylated halloysite nanotubes, and it was carried out by using a procedure similar to that reported in the literature for silica nanoparticles.^51^ Briefly, 0.03 g of HNTs were dispersed in 6 cm^3^ of water and sonicated for 5 min. The dispersion was then added dropwise to 60 cm^3^ of citrate-stabilized AuNPs, and the system was kept under stirring overnight at room temperature. The product was then separated by centrifugation (6000 rpm, 6 min) and washed three times with deionized water to remove any excess.

### Experimental Analysis

Thermogravimetry (TG) was conducted by using a Q5000 IR apparatus (TA Instruments) operating with a N_2_ flow at 25 cm^3^ min^–1^ for the sample and 10 cm^3^ min^–1^ for the balance, respectively. The investigated temperature range was set from room temperature to 800 °C (1073 K), and the heating rate was 20 °C min^–1^. The calibration was previously carried out on the basis of the Curie temperatures of standards.^52^ UV–vis analysis on the obtained samples was conducted by means of a Specord S600 Analytik Jena UV–Vis spectrophotometer; ca. 10 mg of each sample was placed into water, and the spectra were recorded at 25 °C. Dynamic light scattering (DLS) and ζ-potential measurements were performed through a Zetasizer Nano-ZS (Malvern Instruments) under isothermal conditions set at 25 °C. As regards the ζ-potential measurements, a disposable folded capillary cell was used. For DLS tests, instead, the wavelength and the scattering angle were 632.8 nm and 173°, respectively. The field-time autocorrelation functions were analyzed by ILT to obtain the distribution of the aqueous diffusion coefficients. The morphological features of the samples were investigated by using an ESEM FEI QUANTA 200F microscope. The energy of the beam was fixed at 30 kV, and the working distance was 10 mm. Each sample was deposited on a silicon wafer, and the pristine halloysite sample was coated with gold in argon by means of an Edwards Sputter Coater S150A to avoid charging under the electron beam.

### Computational Details

The species involved in the present investigation, which will be labeled as Au_*n*_/fHNT (*n* = 1–20), were modeled by using two-layer ONIOM calculations performed with Gaussian 16 software.^53^ This method involves initially selecting a subset (the “model system” in ONIOM terminology) of the entire system (the “real system”), which requires a more detailed description. High-level and low-level calculations are performed for the model system, whereas for the real system, only low-level calculation is applied. The real system was built starting from the HNT minimax model already used in our previous computational investigations on halloysite^32,50,54–56^ and labeled 7AlSiHex in reference,^57^ modified in such a way to mimic the effects of a strong alkaline treatment, resulting in the presence of three silanolic moieties on the surface;^58^ these protruding groups bond the AEAPTMS residue, which in turn coordinates the gold cluster through its amino groups. The portion composed by these last three components (the silanol groups, the AEAPTMS residue, and the cluster) defines the ONIOM model system, as represented in Figure 1. Hydrogen atoms were used as links. The chosen theoretical approaches were: (i) for the high level: M06 exchange correlation-functional joined with the cc-pVDZ basis set for light atoms, along with the cc-pVDZ-PP pseudopotential and the associated valence basis set for Au; (ii) molecular mechanics based on the universal force field (UFF) for the low level of calculation. As a result of various trials, it was found that excluding the microiterations from the ONIOM optimization protocol stabilizes the convergence behavior of the algorithm; furthermore, the charge embedding scheme was used to consider the electronic influence on the model system deriving from the remaining portion of the real system. The use of the ONIOM method has allowed us to calculate the vibrational frequencies of the system; the model can therefore be used in the future in a homogeneous way for the study of catalyzed processes, where the frequencies are essential for the characterization of transition states. All energetics are discussed in terms of SCF electronic energy plus the vibrational zero-point contribution.

**Figure 1:**
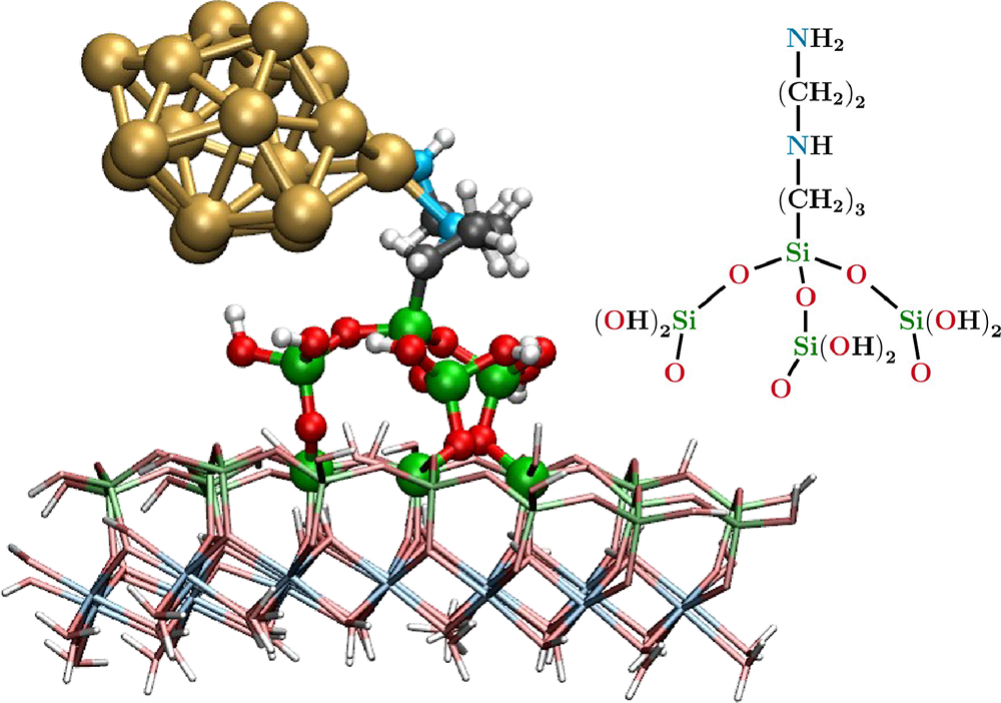
Au_17_/fHNT system is reported as an example for the definition of the model system in the ONIOM approach, which in this work comprises all of the atomic centers in ball-and-stick representation, with the silicon atoms at the level of the surface replaced by hydrogen link atoms. The inset shows the active portion of the modified halloysite model, along with the AEAPTMS residue.

## Results and Discussion

### Computational Studies of the Au_*n*_/fHNT Systems

For structural studies, small gold nanoclusters are more conveniently investigated by using computational methods rather than spectroscopic techniques. While valuable, spectroscopic approaches are often prohibitively expensive and insufficient when used alone, requiring integration with other methodologies for a comprehensive analysis. Consequently, most studies in this area are theoretical in nature. DFT investigations in the literature consistently indicate that gold nanoclusters start to adopt three-dimensional structures as the number of atoms increases. There is, however, some disagreement regarding the exact two- to three-dimensional transition point, which varies depending on the exchange-correlation functional and the basis sets employed.^59^ As a result, researchers have commonly focused on clusters with higher atom counts, as their growth patterns are generally more predictable. The present study shifts the focus to smaller clusters, aiming to elucidate their behavior and structural tendencies when attached to fHNT.

The anchoring of gold nanoclusters on the HNT model functionalized with AEAPTMS was examined by exploring a large number of possible configurations. The initial geometry of Au_*n*_/fHNT systems was built by locating in different positions the Au_*n*_ cluster (*n* = 1,2, ···, 20), whose structure was preliminarily optimized in the isolated state, at interaction distance with the amino moieties of the organosilane residue.

The first phase of the investigation, the one involving the study of isolated Au_*n*_, allowed discrimination of those cluster structures showing the lowest energies, which were selected as the basis for the investigation of the anchored clusters. Through extensive analysis of various geometries, it was found that clusters consisting of 3–12 gold atoms favor a two-dimensional configuration, whereas larger clusters do prefer a three-dimensional structure. The behavior obtained with the M06 exchange-correlation functional employed in this work aligns with that of the revTPSS, which, after a comprehensive investigation,^60^ was deemed as the most appropriate for gold clusters in the considered size range. As a matter of fact, M06 can be considered as the better compromise for a homogeneous treatment of the cluster and the organic portion of the systems studied. As regards the spin multiplicities of gold clusters, it was ultimately determined that all structures composed of an even number of atoms exhibited a singlet multiplicity state, while for clusters with an odd number of atoms, the preferred spin state is a doublet. Triplet and quartet spin multiplicities are therefore ruled out. The same spin states were used for the clusters interacting with the fHNT.

The characteristics of the relevant local portion of the Au_*n*_/fHNT systems are described in the following. For the sake of a convenient representation, the images corresponding to the optimized geometries of the investigated systems were separated into three groups: Figure 2 shows the Au/fHNT–Au_9_/fHNT systems, where the clusters have stable planar arrangements, and Figure 3 reports the geometries of the Au_10_/fHNT–Au_13_/fHNT systems, showing the difference between the anchoring of the two- and three-dimensional clusters, and those of the Au_13_/fHNT–Au_20_/fHNT aggregates, all bearing three-dimensional clusters, are reported in Figure 4. Finally, the anchoring distances between Au sites and the two amino groups of AEAPTMS were collected in Table 1. The optimized geometries of all of the systems investigated in this work are reported in the Supporting Information.

**Table 1:** Au–N Coordination Bond Lengths (Å) in the Optimized Geometry of Au_*n*_/fHNT Investigated Systems^a^

*n*	Au–NH_2_	Au–NH	n	Au–NH_2_	Au–NH
1	2.991	2.489	11	2.407	2.591
2		2.239	12	2.391	2.577
3	2.204		13	2.436	2.497
4	2.261	2.926	14	2.484	2.433
5	2.319		15	2.457	2.491
6	2.495	2.499	16	2.371	2.646
7	2.478	2.529	17	2.531	2.423
8	2.482	2.457	18	2.280	
9	2.631	2.389	19	2.458	2.550
10	2.348	2.730	20	2.325	

a Missing data indicate a distance longer than 3 Å.

**Figure 2:**
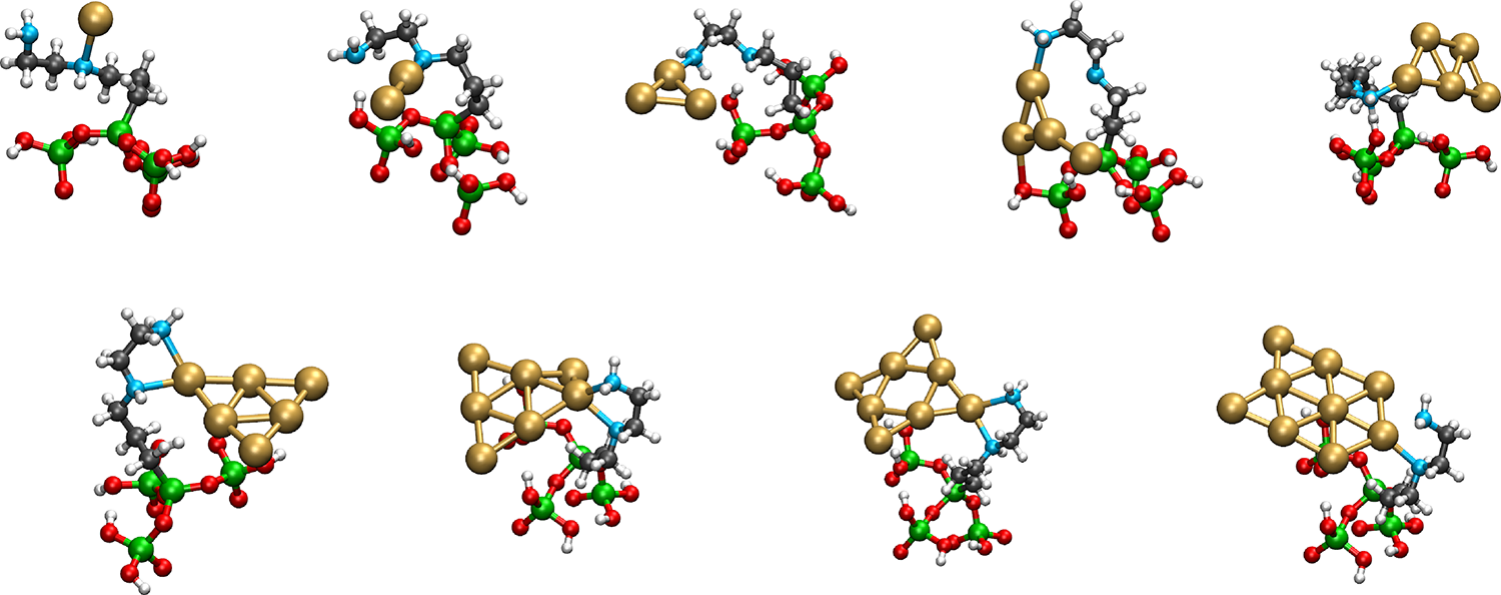
Schematic representation of the optimized geometries corresponding to the most stable systems where a gold atom or a gold Au_*n*_ cluster (*n* = 2 – 9) is anchored on the modified halloysite model bearing an AEAPTMS residue. For the sake of clarity, only the model system of the ONIOM calculation is shown.

**Figure 3:**
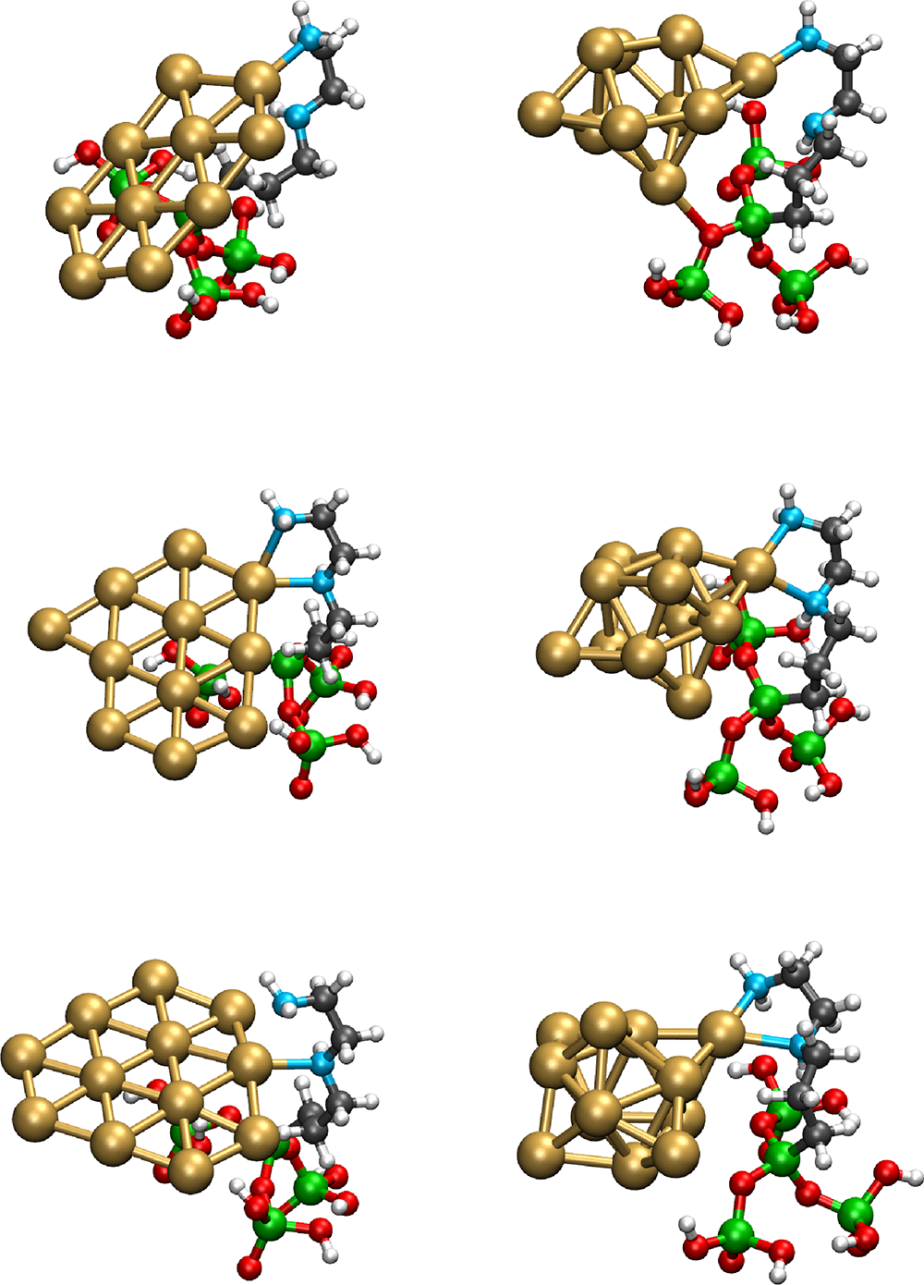
Schematic representation of the optimized geometries of systems where a gold Au_*n*_ cluster (*n* = 10–12), in its planar (left column) and three-dimensional (right column) forms, is anchored on the modified halloysite model bearing an AEAPTMS residue. For the sake of clarity, only the model system of the ONIOM calculation is shown.

**Figure 4:**
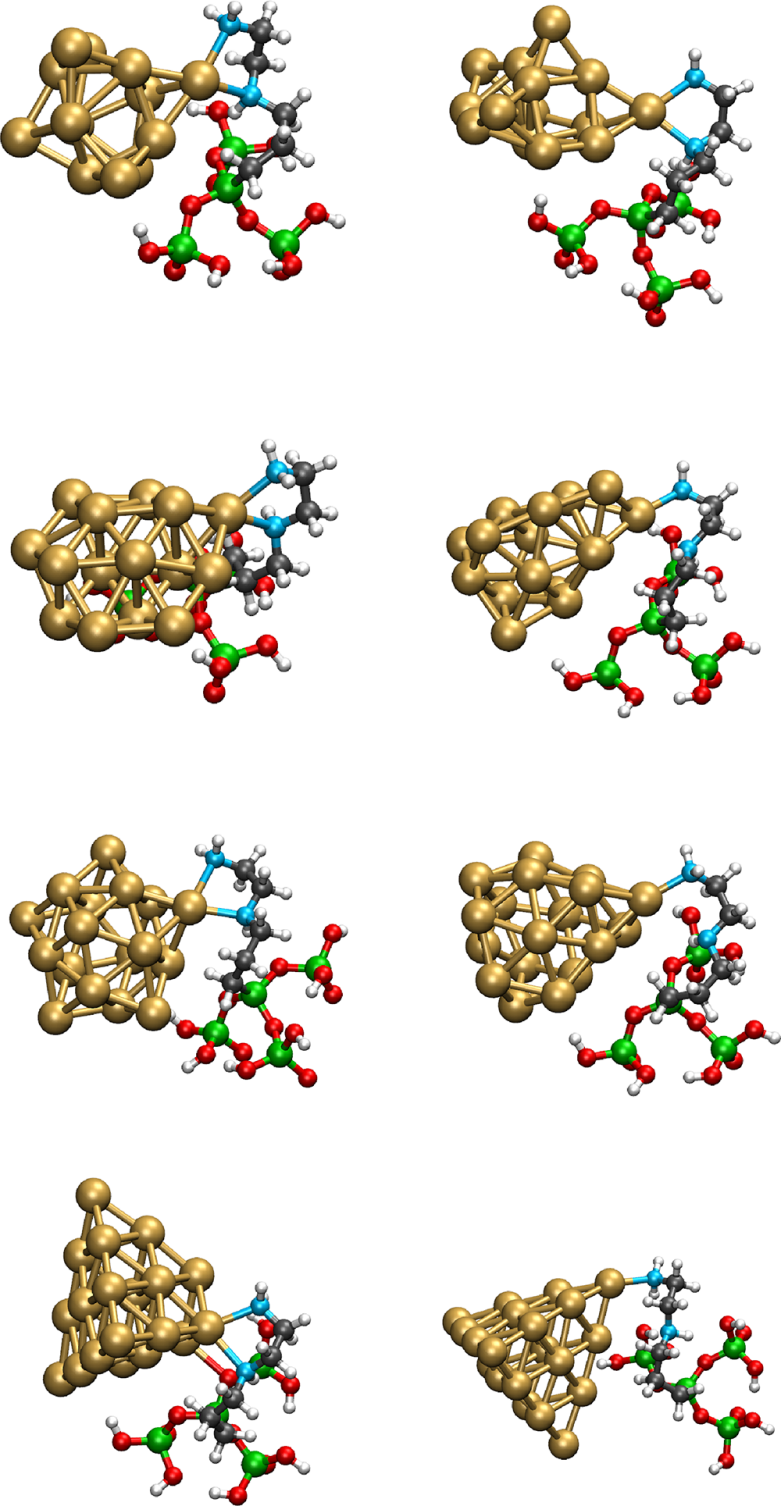
Schematic representation of the optimized geometries of systems where a gold Au_*n*_ cluster (*n* = 13–20) is anchored on the modified halloysite model bearing an AEAPTMS residue. For the sake of clarity, only the model system of the ONIOM calculation is shown.

#### The Anchoring Characteristics

The geometry of Au_*n*_/fHNT systems is governed by a large set of variables: the interaction of gold with the two nitrogen atoms of the AEAPTMS chain can be thought to be the predominant factor, but it is also necessary to take into account the steric effect suffered by the cluster originating from the surface of the halloysite model as well as the weak forces that can manifest themselves in many ways: hydrogen bonds between the amino groups and the surface silanols, interactions between Au and the silanols themselves, interactions that regulate the flexibility of the chain belonging to the AEAPTMS fragment. As will be discussed, the presence of these interactions has a greater effect on smaller clusters, generating many interaction modes in a narrow energy window.

The interaction of a gold atom with the organosilane-modified halloysite has already been discussed by Bertini et al.,^32^ who reported an interaction energy between Au and the amino group of (EtO)_3_Si­(CH_2_)_3_NH_2_ (APTES, an organosilane similar to AEAPTMS but with a single amino group) equal to 42.8 kJ mol^–1^. In the case examined in this work, the presence of two amino groups in the organosilane residue could suggest the occurrence of a chelation of gold. As a matter of fact, if the value of 2.39 Å corresponding to the Au–N distance in Au-APTES-HNT is taken as a reference, in the system with the AEAPTMS residue, the situation shown in the optimized geometry cannot be said to correspond to a proper chelation. In fact, the Au–N distances are equal to 2.489 and 2.991 Å, and the interaction energy, equal to 37.1 kJ mol^–1^, certainly does not indicate an interaction stronger than the one occurring in the system with the APTES residue.

The coordination mode varies sensibly in the Au_2_/fHNT system, since in this case the weak interaction with NH_2_ is lost (this group prefers to act as an acceptor of a hydrogen bond from a surface silanol) and the Au–NH distance becomes significantly shorter. This structure is, however, only slightly more stable (4.1 kJ mol^–1^) than a second possible one in which Au_2_ binds exclusively to the terminal NH_2_ group (with a distance of 2.214 Å), with the chain of the AEAPTMS residue bending toward the surface and the formation of a different network of hydrogen bonds. This would allow one to think that the geometry of the Au_2_/fHNT system is discriminated not only by the interactions between gold and nitrogen but also by the possibility for the organosilane chain to interact in different ways with the surface of the HNT model. In the case of the most stable configuration of Au_3_ in fHNT, it seems that the AEAPTMS chain, in particular in relation to its unfolding and the formation of hydrogen bonds with the silanol groups, cannot adapt well to interact with the cluster, which prefers to leave the interaction with NH and to bind exclusively to the nitrogen of the primary amino group. At slightly higher energy (10.5 kJ mol^–1^), there is a second quasi-chelated structure (Au–NH_2_ = 2.271 Å, Au–NH = 2.863 Å), which also shows an interaction with an oxygen of the underlying halloysitic surface. This kind of anchoring geometry is, on the other hand, the most stable situation for the Au_4_ cluster, and manifests itself with an interaction energy of 18.2 kJ mol^–1^, higher than the one corresponding to a structure that would be associated with a growth starting from the preferred geometry of the Au_3_/fHNT system. It is important to underline that in Au_4_/fHNT the cluster loses the rhombic geometry that it assumes in the isolated state and adopts a geometry in which an Au atom is placed on a vertex of an Au_3_ triangle. It is evident that this geometry is stabilized by coordination interactions involving not only the nitrogen of the terminal amino group but also a surface silanol, which produces a Au–O bond of length equal to ca. 2.5 Å. In the case of Au_5_, finally, the interactions between gold atoms seem to prevail, and the anchored cluster assumes again the shape it would have in the isolated state, that is, that of an isosceles trapezoid. The cluster is coordinated only by the NH_2_ group and, according to our calculations, an alternative chelated structure would be 18.6 kJ mol^–1^ less stable.

Starting from the Au_6_/fHNT system, the geometric characteristics of cluster anchoring begin to show some regularity, at least as concerns the size regime where clusters show a planar arrangement. The Au_6_ cluster shows a nearly perfect chelation; its interaction with the silanol base leads to slight distortions from planarity, quantifiable with an angle of 12° between the triangular portion bound to the AEAPTMS and the underlying trapezoidal portion of the cluster. Distortions from planarity become much more evident in the cases of anchoring of the Au_7_ and Au_8_ clusters. In vacuum, the Au_7_ cluster should have a planar geometry in which an Au atom is arranged on one of the exposed sides of the triangles that form Au_6_. In Au_7_/fHNT, even though there is no hindrance that prevents it from assuming the correct position in space, this additional gold atom prefers to bend, by about 50°, with respect to the Au_6_ plane. This is probably due to a weak interaction with an oxygen atom of the silanol base. In the structure just described, Au_7_ bound to the AEAPTMS residue has a 3-coordinated Au atom; a second geometry (16.6 kJ mol^–1^ less stable than the first) was found in which Au_7_ is chelated by AEAPTMS (Au–NH = 2.910 Å, Au–NH_2_ = 2.316 Å) by means of a 2-coordinated gold atom while a 3-coordinated one approaches and binds a silanol OH group. Incidentally, the atomic center that binds to this oxygen is exactly the same as that in the first structure examined, the most stable, which was bound to nitrogen, suggesting that this particular 3-coordinated Au is the most reactive site in this cluster. According to the M06 functional, the geometry of isolated Au_8_ shows D_4h_ symmetry, with four atoms located on the sides of a Au_4_ planar square arrangement. Following the interaction with fHNT, the symmetry of the cluster is lowered to D_2d_, with a structure in which the atoms on the sides of the square are arranged alternately above and below the plane defined by it. This structure is slightly distorted by interactions with the silanol groups. Although the geometry of Au_8_ may appear very different from that of Au_7_, it can be seen that it easily falls into a regular growth pattern. In fact, by adding a gold atom, Au_7_ can be transformed into Au_8_ simply by converting its central rhombus into a square. The chelating coordinations of Au_6_, Au_7_, and Au_8_ with the AEAPTMS fragment are very similar to each other, with Au–NH distances between 2.46 and 2.53 Å and Au–NH_2_ distances within a narrow range around 2.48 Å.

The distortions from planarity appear to decrease when the anchoring of the clusters Au_9_-Au_12_ is considered. The Au_9_ cluster, which exhibits a hexagonal shape, with two triangles connected to two adjacent edges of the hexagon, is coordinated asymmetrically with a Au–NH_2_ distance considerably longer than the Au–NH one; it actually shows only a very slight (ca. 8°) helical twist. This is also the case for Au_10_, ideally composed of two hexagons, fused together in such a way that the vertex of the first is the center of the second one, whose distortion from planarity is concentrated on the bonded Au site, forming an angle of 15° with the plane of the other Au atoms. The cluster, which is arranged almost parallel to the organosilane residue chain, is mostly coordinated by the terminal NH_2_ group. At slightly higher energy (8.9 kJ mol^–1^, which is essentially the same as that calculated in vacuum), a structure with a three-dimensional Au_10_ cluster exists. Here, the gold particle has the geometry of a triangular prism capped on all faces but one (Figure 3); the anchoring site is one of the capping atoms (that on the triangular face), which is mostly chelated by the primary amino group (Au–NH_2_ = 2.407 Å versus Au–NH = 2.636 Å). The Au atom located on the square face of the prism is bound to an oxygen atom of a Si–O–Si portion of fHNF, which could contribute to the stabilization of the 3D form.

In the Au_11_ case, the three-dimensional arrangement in the vacuum is essentially isoenergetic to the two-dimensional one (3.8 kJ mol^–1^ in favor of 2D), but when anchored on the fHNT system, the 3D geometry of the cluster becomes the most stable (by 13.9 kJ mol^–1^). In its planar form, the Au_11_ cluster, which is the least symmetric of the set we are considering, is coordinated in the most symmetric way by both amino portions of the AEAPTMS, with an average Au–N distance equal to 2.52 Å (Au–NH_2_ = 2.569 Å and Au–NH = 2.472 Å). It is noted that moving from the 3D to the planar, slightly twisted Au_11_, there is a variation in the Au atom that is bound. When the globular form is considered, Au_11_ assumes a structure consisting of a core having the shape of a triangular-based prism (such as the 3D geometry of Au_10_), each face of which is capped by an Au atom. One of these last Au sites is chelated by AEAPTMS in a more irregular way if compared to the 2D case, with a large propensity toward the NH_2_ moiety (see Table 1).

Similarly, Au_12_ is predicted in vacuum as a planar cluster by the M06 functional, with an energy difference of −10.2 kJ mol^–1^ relative to the three-dimensional geometry (to note: the 3D form of this cluster is not as regular as that of Au_11_ above, while the reverse situation can be stated for the planar one). According to the results obtained (see Figure 3), things change again when this cluster interacts with fHNT, as the Au_12_/fHNT system shows a three-dimensional cluster that is 26.6 mol^–1^ more stable than the one with the planar counterpart. The 3D Au_12_ cluster can be seen as the result of the bonding of one Au atom, just the one that is chelated by AEAPTMS, with one side of the triangular prism and with the atom capping an adjacent face. Again, the cluster shows peculiar chelation distances for this size range, favoring the interaction with the primary amino group. In the case of the planar arrangement, the cluster is chelated at larger distances (Au–NH_2_ = 2.615 Å, Au–NH = 2.468 Å), and the atom interacting with the amino groups is part of a chain of three atoms, which would make Au_12_ the planar cluster where the bound gold center has the highest number of first neighbors. This form would have very slight distortions from the exact planarity, consisting of a curvature from the center outward.

It is worth emphasizing that is the Au_12_(3D)/fHNT system as a whole that shows larger stability than the Au_12_(2D)/fHNT system, and this can be due to different aspects that influence the energetics of the composite aggregate. For example, the interactions of a 3D cluster with silanols can be more favorable, because the same number of vertices a 3D cluster shows a large number of edges with respect to the 2D ones. If a given Au atom of a planar cluster approaches a silanol group, the entire plane should follow it, possibly resulting in steric hindrance. This does not always have to be the case, but this aspect could become increasingly important as the size increases: indeed, it should be noted that the coordination to the AEAPTMS occurs in a region very close to the possibly interacting silanols, and when the cluster is large (and planar), its orientation with respect to the fHNT might not be favorable for the establishment of secondary interactions. A globular cluster, on the other hand, could more easily rotate around the chelation site and arrange itself to interact better. Then, the energy of the composite system is also affected by the position of the AEAPTMS chain, which can interact differently with the underlying part in a way that depends on the geometry of the cluster, whether planar or three-dimensional. The fact that in the vacuum a given cluster shows a 2D structure, while when anchored on fHNT it adopts a 3D geometry due to favorable interactions, would mean that the energy associated with these interactions is larger than the 2D–3D energy difference. In fact, this is exactly the case if we consider the 2D–3D energy differences for the Au_11_–Au_13_ particles in the vacuum calculated at the M06 level. Therefore, it is not a surprise that a transition from 2D to 3D geometry in the Au_*n*_/fHNT composite system could occur when the cluster size is close to the 2D-3D inversion point in vacuum.

From now on, except for a few cases, regular growth will not necessarily be respected. Despite cluster formation could occur through a growth process from smaller clusters after the anchoring on halloysite is occurred (our group is conducting a detailed investigation in this direction), or even atom by atom, we can hypothesize that, over a certain period of time and thanks to the high fluxionality typical of metal clusters,^61^ the particle could deform to adapt to its most stable shape. It is crucial to point out that this is not necessarily the shape it would have in a vacuum, as in the systems investigated interactions with the AEAPTMS chain and a significant number of interactions with the halloysite surface exist. Even if these interactions could be considered weak if compared to the bonds between gold atoms, as already discussed, they can significantly influence the cluster structure, particularly when it can have different possible geometries within a narrow energy window.

According to the M06 exchange-correlation functional used in this work, Au_13_ is the first gold cluster showing a three-dimensional geometry as the preferred structure in the vacuum; in Au_13_/fHNT, the globular geometry is indeed 32 kJ mol^–1^ more stable than the planar one ideally deriving from planar Au_12_ through one-atom growth. The 32 kJ mol^–1^ energy difference above is almost twice the difference calculated for the 2D and 3D forms in a vacuum, which corroborates the hypothesis that three-dimensional clusters can be stabilized by the interactions with fHNT. When bonded to the nanotube surface, the Au_13_ cluster shows a slightly distorted C_s_ symmetry structure that can be seen as the result of the union of a square-based pyramid with a tetrahedron, with four additional atoms acting as linkers: each of the four sides of the pyramid coordinates an Au atom, two vertices of the tetrahedron are bonded to four atoms, and one vertex is linked to three. This cluster is chelated in a regular manner by the two amino groups of the AEAPTMS residue, an aspect that is common to the Au_13_–Au_15_ set (see Table 1). On the other hand, the optimized geometry of the cluster in the Au_14_/fHNT system can be viewed as a tetrahedron placed at the center of a triangular arrangement of 10 gold atoms, whose planarity is modified by a sharp curvature starting from the center of the triangle. This geometry possesses formal C_3v_ symmetry. Au_14_ is linked to AEAPTMS through one of the vertices of the triangular arrangement; coordination of the site at the vertex of the tetrahedron, although possible, would have been at a higher energy since steric hindrance would have caused the cluster to be exposed upward and the stabilizing interactions with the halloysite surface to be lost. By comparison, when optimized in the composite system, the planar form of this cluster is found at an energy of approximately 150 kJ mol^–1^ with respect to the most stable form. In a second, higher-energy structure (110 kJ mol^–1^), the above planar structure crumples due to interactions with the fHNT. This behavior would indicate that in larger gold nanoclusters, the planar form, if it exists as a minimum in the potential energy surface, is increasingly less stable as the number of atoms increases and increasingly susceptible to transitioning to a three-dimensional geometry following perturbing interactions, even weak ones, with the surrounding environment. This argument would rule out the presence of coordinated planar clusters as stable structures after Au_13_. The Au_15_ cluster consists of two single-atom-centered hexagons, slightly distorted from planarity and stacked on top of each other so that one vertex of one hexagon overlaps the center of the other; the 15th gold atom is bonded to the sides of the two hexagons and is chelated in an essentially regular manner to the two amino groups of the fHNT system.

The final block is formed by the Au_*n*_/fHNT (*n* = 16–20) systems. In agreement with the data reported in Table 1, the gold clusters in these cases exhibit significant irregularity in chelation. In particular, Au_18_ and Au_20_ do not interact with the secondary amino group of the organosilane residue.

Au_16_ can be viewed as derived from Au_15_, with a second gold atom joining the two hexagons, close to the one chelated in Au_15_. The two hexagons are significantly more distorted, leading the cluster to assume a more globular shape. The chain of the AEAPTMS residue to which the cluster is chelated, with a certain preference for the primary amino group, is arranged essentially parallel to the surface of the halloysite model, a configuration that evidently minimizes steric effects while maintaining the cluster interactions with that surface. This aspect is also found in the optimized geometries of the larger clusters. The Au_17_ species bound to fHNT appears as a solid five-pointed star: it can be described as consisting of two regular pentagons capped at the center and connected to each other by five gold atoms, one on each side of the pentagons. If the slight distortions due to the interaction with fHNT were not taken into account, then the cluster would have a remarkable D_5h_ symmetry. It displays ten atoms with 6-coordination, two with 5-coordination, and five with 4-coordination; within the set we are discussing, Au_17_ (followed by Au_19_) is the cluster most regularly chelated to the two amino groups, and chelation occurs by one vertex of the star. The least regularly chelated one is instead Au_18_, which shows an Au–NH_2_ coordination distance of 2.28 Å, which would seem more appropriate for very small clusters. Although the secondary amino group is in the correct orientation for chelation, the Au–NH distance is 3.18 Å, and this amino group prefers to form a strong hydrogen bond by acting as a donor to the oxygen of a Si–O–Si moiety on the surface. Au_18_ can be seen as a superposition of three structures: it has a hexagonal base with a central atom positioned outside the plane of the polygon; a second hexagon (this time without a central atom) is rotated 30° with respect to the first and superimposed on it; and finally, above the second hexagon is a pentahedron with its base bent along the main diagonal. This pentahedron lowers the symmetry of the cluster from C_6v_ to C_2v_. It is noteworthy that this cluster is hollow inside: if we measure the diagonal of the central hexagon (6.6 Å), use the value of 1.44 Å for the metallic radius of gold, and assume the cavity to be spherical, we obtain a volume of 27 Å^3^. In contrast, the clusters in the Au_19_/fHNT and Au_20_/fHNT systems are densely packed. They essentially have the same structure: Au_20_ is a tetrahedral object in which each side is made up of four atoms, while one atom is placed at the center of each triangular face. Au_19_ is the same, except that one of the vertices of the tetrahedron is missing. Strange as it may be, this latter species prefers to be chelated, quite regularly, via an Au atom that is part of the truncated portion of the tetrahedron, therefore having coordination 5. In fact, in the case of Au_20_, this same atom has coordination 6 and the balance between steric hindrance and weak interactions with the fHNT results in the fact that the preferred bonding arrangement for Au_20_ is only through interaction between a 3-coordinated Au site of one vertex and the −NH_2_ group, while the distant secondary amino group is engaged in two hydrogen bonds, one as an acceptor and one as a donor with respect to two silanol groups. Incidentally, the largest species considered in this work, Au_20_, inspired the synthesis of the first atomically precise gold nanocluster.^62^

### Adsorption Energetics

To the investigated Au_*n*_/fHNT systems described above, different kinds of energetics could be associated, which are analyzed in the following from a purely thermodynamic point of view. The simplest processes are the anchoring of the Au_20_ cluster, eq 1, and the atom-by-atom growing, eq 2:

$${\mathrm{Au}}_{n}+\mathrm{fHNT}\to {\mathrm{Au}}_{n}/\mathrm{fHNT}$$ 1

$${\mathrm{Au}}_{n}/\mathrm{fHNT}+\mathrm{Au}/\mathrm{fHNT}\to {\mathrm{Au}}_{n+1}/\mathrm{fHNT}+\mathrm{fHNT}$$ 2

The first reaction corresponds to a process according to which a preformed Au_*n*_ cluster simply attaches on the AEAPTMS residue of the modified HNT surface; the latter, on the other hand, is associated to a hypothetical growing process where a given Au_*n*+1_ particle forms on the surface by an Au atom attachment on Au_*n*_, with one AEAPTMS residue acting as anchoring support for the growing cluster and another AEAPTMS residue acting as the supplier of a new gold atom. Since in the used HNT model there is only one AEAPTMS moiety, the Au supplier is considered far away from the growing center (actually on a separate system); this is correct from the thermodynamic point of view, except for the loss of the contribution due to the possible interactions between two close AEAPTMS residues. The reaction energy associated with the two processes, 1 and 2, will be indicated as *E*_a_ and *E*_g_, respectively, and their values are reported in Table 2. The energetics associated with the anchoring process do not show a regular trend; it is noted that the lowest *E*_a_ magnitude corresponds to the anchoring of the Au_6_ cluster (the process is the least exothermic), and the highest corresponds to the anchoring of Au_16_. A detailed analysis shows that there is no simple correlation between *E*_a_ and the chelation geometry, the Au–N interaction distance(s), the spin multiplicity of the cluster, or even the coordination number of the gold atom bound to the organosilane residue. Generally speaking, however, it can be stated that the anchoring energy is higher for globular clusters; the average *E*_a_ value for cases showing clusters with a planar geometry (*n* = 1 – 10) is 155 kJ mol^–1^, while that for cases with three-dimensional clusters is 179 kJ mol^–1^. The explanation for the stronger interaction shown by the 3D clusters may lie in the greater number of interactions that they can establish with the silanol groups of the fHNT and in the fact that the presence of the cluster can force the AEAPTMS chain to assume a certain position that allows it to form a number of interactions, hydrogen bonds in particular, with its surroundings.

**Table 2:** Energy (kJ mol^–1^) Values Corresponding to the Anchoring Process Described in Eq 1, *E*_a_, and to the Growing Process by One Atom Attachment Illustrated in Eq 2, *E*_g_, Associated with the Formation of the Putative Most stable Au_*n*_/fHNT Systems

*n*	*E*_a_	*E*_g_	*n*	*E*_a_	*E*_g_
1	–37.1		11	–167.8	–140.6
2	–156.2	–288.2	12	–175.7	–233.5
3	–176.4	–84.3	13	–184.6	–157.3
4	–184.6	–201.5	14	–173.4	–220.4
5	–151.2	–126.3	15	–146.0	–179.5
6	–120.9	–213.5	16	–198.1	–267.8
7	–147.4	–123.0	17	–170.5	–194.4
8	–151.9	–226.2	18	–194.9	–258.0
9	–147.2	–114.8	19	–183.3	–166.8
10	–160.7	–239.5	20	–194.5	–268.7

To test this hypothesis, already mentioned to explain the stabilization of the three-dimensional shape of the Au_11_ and Au_12_ clusters, calculations were performed on systems in which no secondary interactions other than chelation exist. In particular, the Au_*n*_ clusters (*n* = 5, 9, 10, 15, 20) were selected, and the energy associated with their interaction with *N*-propylethylenediamine, i.e., the silicon- and silanol-free AEAPTMS chain, was evaluated. The above clusters were chosen because they exhibit two spin states and a variety of interaction geometries: Au_5_ is bonded only to the primary amino group, Au_9_ shows a chelation shifted toward the secondary amino group unlike that of Au_10_, which is instead shifted toward the primary amino group; Au_15_ has an almost perfectly symmetric chelation with respect to the two amino groups and Au_20_, finally, represents a large cluster coordinated only to −NH_2_. Obviously, this is only a rough guide to study a sort of interaction reference; however, the results obtained (Table S1 in the Supporting Information) show that the interaction energy is essentially the same regardless of the spin, chelation geometry, and size of the cluster. It has an average value of 126 kJ mol^–1^, and all values fall within an energy window of approximately 12 kJ mol^–1^. The reference system also allowed us to evaluate the effect of the basis set superposition error: it could be the case that the anchoring energy of larger clusters on fHNT appeared higher due to a higher BSSE. In fact, in the small model, the BSSE remains in a range from 18 to 27 kJ mol^–1^ (Table S1), so that it too can be considered essentially independent of the characteristics of the system. These data can therefore be considered as a clue supporting the fact that the modified halloysite support can intervene in a rather sensitive way in the stabilization of larger nanoclusters.

Taking a look at the energy values corresponding to the cluster growth by adding one atom at a time (*E*_g_ in Table 2), it can be noted that the formation of the Au_2_ dimer is the most exothermic reaction and that this cluster is the least likely to grow (conversion to Au_3_ is by far the least exothermic process, which is in agreement with the dissociation energy values reported by Ding et al.^63^), evidently proving to be extremely stable. The formation of clusters in the doublet state (odd *n*) is always less exothermic than the formation of clusters with singlet spin multiplicities. Leaving out the smaller clusters, the transition from Au_2*k*+1_ to Au_2*k*+2_ involves a higher energy release, by an amount in the range of 60–120 kJ mol^–1^, than that relating to the transition from Au_2*k*_ to Au_2*k*+1_. Even in this case, although this is a very different process from anchoring, we find that larger clusters (both singlets and doublets) exhibit higher *E*_g_. Here too, we can assume that there is a contribution due to the interaction with silanols, but in this case, this contribution should be less significant since such interactions can also exist in the reagent with one fewer atom. It is therefore likely that the effect is inherent in the nature of larger clusters and can be traced back to the increase in the average coordination number (Table S2) that each gold atom can experience in an increasingly globular cluster.

Finally, in extension of reaction ([Disp-formula eq2]), a more general growth process can be considered

$${\mathrm{Au}}_{i}/\mathrm{fHNT}+{\mathrm{Au}}_{j}/\mathrm{fHNT}\to {\mathrm{Au}}_{i+j}/\mathrm{fHNT}+\mathrm{fHNT}$$ 3

where the cluster Au_*i*+*j*_ is formed by the coalescence of two preformed Au_*i*_ and Au_*j*_ clusters anchored on separate fHNT systems, simulating nearby AEAPTMS residues in a single surface. The coalescence energy, *E*_c_(*i*, *j*), associated with the cluster growth is illustrated in Figure 5. The overall number of atoms present in a given cluster is indicated in the diagonal lines, so that the different ways this number can be achieved through different combinations of *i* and *j* are confined within the points along the corresponding connecting line. Specifically, the circle radius shown at the intersection of a given (*i*,*j*) pair coalescing in Au_*i*+*j*_ is proportional to the magnitude of the *E*_c_(*i*, *j*) value relative to the other clusters in the diagonal line; the color of a circle, on the other hand, is determined by the value of *E*_c_(*i*, *j*) relative to the whole set of clusters.

**Figure 5:**
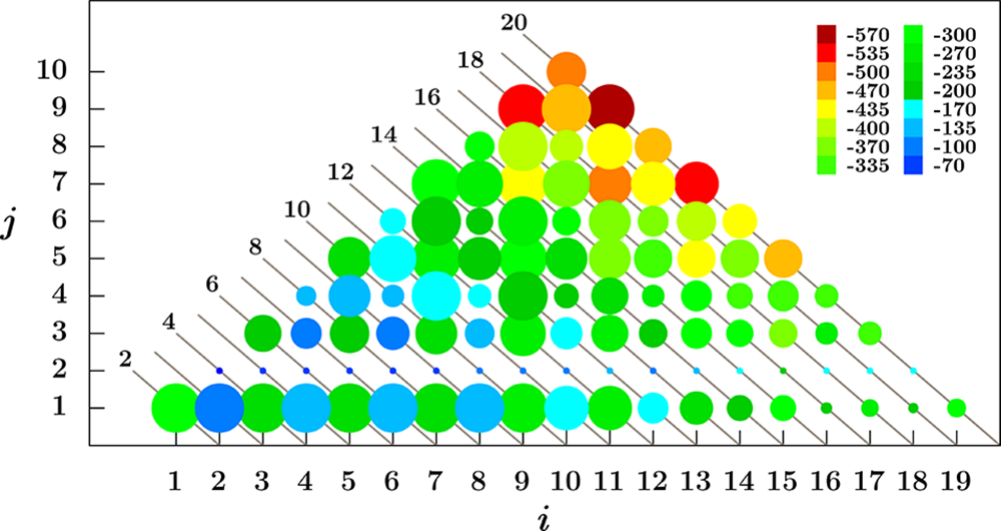
Graphical representation of the energetics associated with clusters coalescence on fHNT showed through the *i* + *j* correlation according to the process ([Disp-formula eq3]). The radius of each circle along the diagonal lines is proportional to the energy released by the formation of the Au_*i*+*j*_/fHNT system for different (*i*,*j*) pairs sharing the same value of *i* + *j*. The color of the circles is instead generated by the comparison between the energy release associated with the formation of Au_*i*+*j*_/fHNT (the warmer, the higher) when different *i* + *j*, i.e., different cluster sizes, are considered. The values reported in the color key are the lower end of the range associated with each bin; the last bin includes all the energy values lower than 70 kJ mol^–1^ in magnitude. The calculated coalescence energy values collected in this diagram can be found in the Supporting Information.

The analysis of Figure 5 and of the corresponding energy values (see Supporting Information) shows indeed the existence of some trends. It is evident that the coalescence processes starting from clusters in the doublet spin state and ending in aggregates in the singlet state always represent the most exothermic ones, so that the formation of clusters with an even number of gold atoms is thermodynamically favored. For planar clusters up to Au_9_, the preferred coalescence mode is the attachment of a single atom; this aspect becomes less prominent as one approaches larger planar clusters, where other coalescence modes compete with the (*i*,1) one. For example, if *E*_c_(5, 1) is ca. 45 kJ mol^–1^ larger in magnitude than *E*_c_(3, 3), the difference between *E*_c_(9, 1) and *E*_c_(5, 5) is reduced to 23 kJ mol^–1^. As cluster size increases and one proceeds toward globular particles, coalescence from preformed medium-sized clusters becomes largely favored. In fact, the higher calculated magnitude for energy releases is associated with the formation of the largest singlet clusters: *E*_c_(11, 9), *E*_c_(13, 7), and *E*_c_(9, 9), which are equal to −568.7, −518.9, and −513.2 kJ mol^–1^, respectively. It is worth noting that starting from Au_13_, the coalescence involving the Au_9_ cluster is always the most exothermic process; even in the Au_12_ case, the *E*_c_(9, 3) value is only 13 kJ mol^–1^ smaller in magnitude than those associated with the preferred (7,5) mode. Indeed, besides the fact that Au_9_ is in the doublet state, by inspecting the coordination numbers reported in Table S2, it can be argued that this cluster contains five atoms with different coordination numbers, many of which have low coordination. This situation is evidently fine for the Au_9_ cluster as such, but if we examine things considering the process ([Disp-formula eq3]), we note that in the case of the coalescence of Au_9_, a good number of Au atoms will find themselves, in the resulting cluster, with a notable increase in their coordination number. To a slightly lesser extent, this also applies to Au_7_, whose coalescence is, in fact, also highly exothermic. The coalescence of a larger, already globular cluster will not necessarily lead to an increase in the coordination number for a number of atoms (since the coordination in the starting cluster is already quite high), so this process, which is, however, extremely exothermic, is not favored as much as that of Au_7_ and Au_9_. A final consideration to be made concerns the Au_2_ cluster: even if *E*_c_(*i*, 2) is always negative (except for the case of *E*_c_(2, 2)), it seems that no cluster is inclined to coalesce with it, generalizing the observation made above on the high stability of this species. It is worth noting that, although it may be considered affected to some extent by the support, this behavior of Au_2_ with respect to coalescence does not appear to be a prerogative of the investigated systems but rather a phenomenon inherent in gold clusters. Indeed, even in the case of isolated clusters, the formation of Au_*i*+*j*_ is always less exothermic if *i* or *j* = 2 (see the selected cases in Table S3 of the Supporting Information).

### Halloysite Decoration with AuNPs: Experimental Studies

The synthesis of gold decorated functionalized halloysite was performed in two steps, which was demonstrated to ensure uniform coverage. At first, the gold nanoparticles were prepared, and the halloysite nanotubes were separately functionalized with AEAPTMS for the creation of specific anchoring sites. The two components were then combined. The attempts for a single-step synthesis, conversely, resulted in poor nanotube coverage and too large gold aggregates. Thermogravimetric analysis was conducted on the pristine halloysite nanotubes, on the AEAPTMS functionalized HNTs, and on the aminoalkylated nanoclays decorated with AuNPs. The thermograms are reported in Figure 6, while the thermogravimetric parameters, i.e., the mass residue remaining at 800 °C (MR800) and the percentage of degraded matter at 800 °C (MD800), are reported in Table 3. As shown in the curves, the major difference lies in the residues at a higher temperature. In particular, the pristine halloysite nanotubes sample (p-HNTs) shows the highest MR800 value compared to the others, namely 85.2%, which is due to the loss of moisture and to the typical dehydroxylation of the inorganic nanotubes occurring between 450 and 500 °C.^64^ On the other hand, the residue for the functionalized HNTs-AEAPTMS (a-HNTs) sample is the lowest, with a value of MR800 = 61.5%, due to the successful functionalization with the organosilane. Indeed, this value strictly depends on the amount of organic matter within the sample, which undergoes complete degradation upon heating. For this reason, the higher the amount of organic moiety, the lower the residue that can be observed at high temperatures.^65^ After the loading protocol of AuNPs onto the surface of the functionalized nanotubes was carried out, instead, the mass residue increased to 84.1% for the composite HNTs-AEAPTMS-AuNPs (ga-HNTs) sample, accounting for a higher fraction of inorganic matter compared to a-HNTs due to the presence of gold. The observed increase in residue after decoration could be tentatively attributed both to the additional inorganic gold fraction and to the stabilizing effect of AuNPs on the nanotube structure. Indeed, according to the derivative thermogravimetry (DTG) curves reported in Figure S1 of the Supporting Information, the interaction between gold nanoparticles and the organic moiety of the functionalized clay also accounts for improved thermal stabilization of the sample. In fact, while a-HNTs display two degradation peaks at 438° and 507 °C, the ga-HNTs sample displays just one degradation peak occurring at ca. 491 °C. The values of degraded matter confirm these results and show a similar trend, being 13.8%, 30.9%, and 15.0% for *p*-HNTs, a-HNTs, and ga-HNTs, respectively. In light of this, it is possible to state that decoration with gold nanoparticles is most likely to occur.

**Figure 6:**
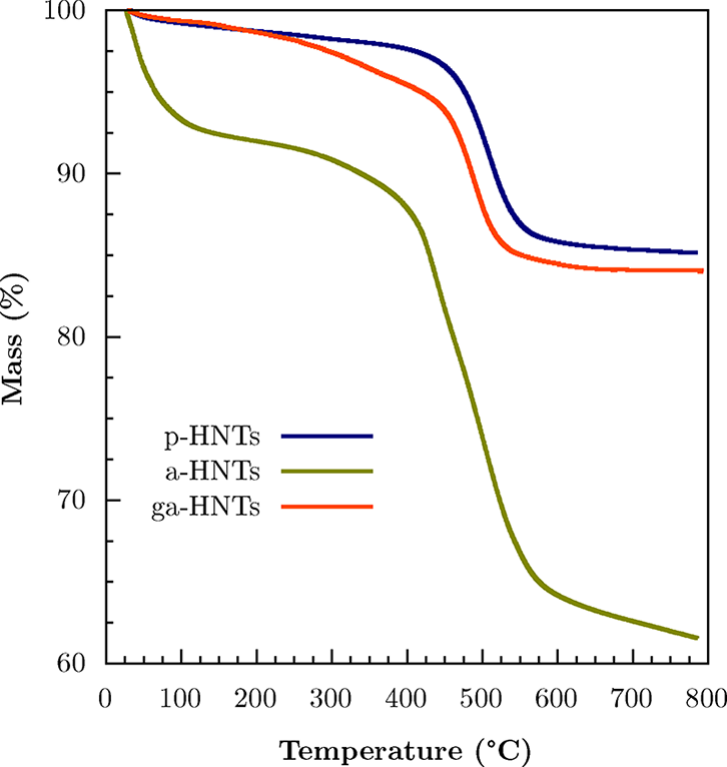
Thermogravimetric curves of the pristine halloysite (p-HNTs), AEAPTMS functionalized halloysite (a-HNTs), and AEAPTMS functionalized halloysite decorated with gold nanoparticles (ga-HNTs).

**Table 3:** Thermogravimetric Parameters (%_wt_) of Pristine HNTs (p-HNTs), AEAPTMS Functionalized HNTs (a-HNTs), and AEAPTMS Functionalized HNTs Decorated with AuNPs (ga-HNTs)

sample	MR800	MD800
p-HNTs	85.2	13.8
a-HNTs	61.5	30.9
ga-HNTs	84.1	15.0

Then, UV–vis analysis was carried out to have more insights into the formation, the shape, and the size of AuNPs and to evaluate their attachment to the halloysite surface. The adsorption spectra are reported in Figure 7. As can be observed, AuNPs display a single adsorption band in the visible region, which is typical for gold nanoparticles with a spherical shape.^66^ It should be noted that conducting the synthesis in a manner that enables the production of spherical nanoparticles, along with precise shape control, is crucial in order to maximize the catalytic efficiency of the entire system. Herein, the peak is centered at 520 nm and, as a consequence, it is possible to state that the AuNPs possess a diameter ranging between 10.9 and 13 nm, in agreement with the literature.^67,68^ As far as the halloysite-based samples are concerned, instead, pristine halloysite nanotubes and AEAPTMS functionalized HNTs do not show any adsorption in the whole spectra. Most importantly, the ga-HNTs sample displays the same peak in the visible region, which is direct proof of the presence of gold nanoparticles attached to the surface of the functionalized clay. Therefore, the aqueous reduction of tetrachloroauric acid by sodium citrate allowed for the production of spherical and nanosized AuNPs whose shape and dimension did not change after loading on the clay-based support.

**Figure 7:**
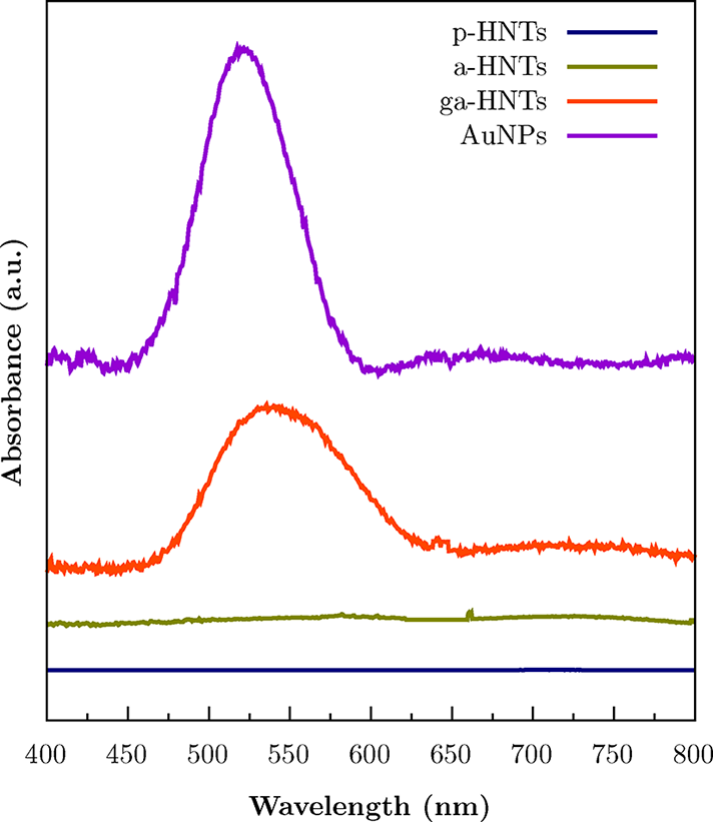
UV–vis spectra of the pristine halloysite (p-HNTs), AEAPTMS functionalized halloysite (a-HNTs), AEAPTMS functionalized halloysite decorated with gold nanoparticles (ga-HNTs), and bare AuNPs.

In order to have further details, DLS and ζ-potential measurements were performed, and the results are collected in Table 4. One of the most important aspects to keep under control during the synthesis is the growth, hence the size of the AuNPs, since these parameters can have major effects on the activity of the catalysts. For this reason, DLS measurements were conducted on the AuNPs sample right after their preparation, and they showed that the particles possess a diameter of ca. 12 nm. Also, the ζ-potential experiments resulted in an overall charge for AuNPs equal to −36.5 mV. These results are in good agreement with previous findings from UV–vis spectrophotometry concerning the dimensions of the gold particles. The negative ζ-potential value is in agreement with the preparation protocol since, upon boiling, aqueous chloroaurate ions are reduced by citric acid/sodium citrate, resulting in AuNPs stabilized by electrostatic repulsions of surface-bound negative citrate ions.^66^ As far as halloysite nanotubes are concerned, instead, the ζ-potential and the diameter of p-HNTs are −26 mV and 369 nm, respectively. However, after the functionalization protocol with AEAPTMS was carried out, the charge decreased to −15.6 mV. Literature reports that the decrease of ζ-potential can be the result of the screening of halloysite negative charges on the external surface due to the interaction with other molecules.^69^ This result, together with the increase in a-HNTs size up to ca. 540 nm, represents further proof of the clay functionalization with the aminosilane. Most importantly, it can be observed that the loading of gold nanoparticles on the external surface has no major effects on the ζ-potential of the system, being its value −13 mV for the ga-HNTs sample. This is in agreement with the computational study, which showed the gold atoms interacting with the nitrogen centers of AEAPTMS in the catalytic system, without any direct bonds with the halloysite structure, and as a consequence, no significant changes in the overall net charge compared to the a-HNTs sample. However, the dimensions of ga-HNTs soared up to ca. 854 nm due to the presence of gold nanoparticles on the external shell. These results represent further proof of halloysite functionalization with the organosilanes, and they confirm the successful deposition of AuNPs by interaction with the amino groups protruding from the clay.

**Table 4:** Thermogravimetric Parameters of Gold Nanoparticles (AuNPs), Pristine HNTs (p-HNTs), AEAPTMS Functionalized HNTs (a-HNTs), and AEAPTMS Functionalized HNTs Decorated with AuNPs (ga-HNTs)

sample	ζ-potential (mV)	hydrodynamic diameter (nm)
AuNPs	–36.5	12.5
p-HNTs	–26.0	369.2
a-HNTs	–15.6	540.3
ga-HNTs	–13.0	853.9

Morphological investigations were finally carried out by scanning electron microscopy (Figure 8). As can be observed by the comparison between p-HNTs and ga-HNTs samples, the hollow nanotubular morphology of the clay is maintained without any noticeable differences. This result suggests that neither the functionalization using the organosilane nor the decoration with gold nanoparticles altered the overall halloysite structure. Moreover, the spherical AuNPs are clearly observed, and they are homogeneously dispersed within the sample. Such findings, i.e., the nanotubular morphology and the dispersion of gold centers, are prominent features for the catalytic application of the designed materials.

**Figure 8:**
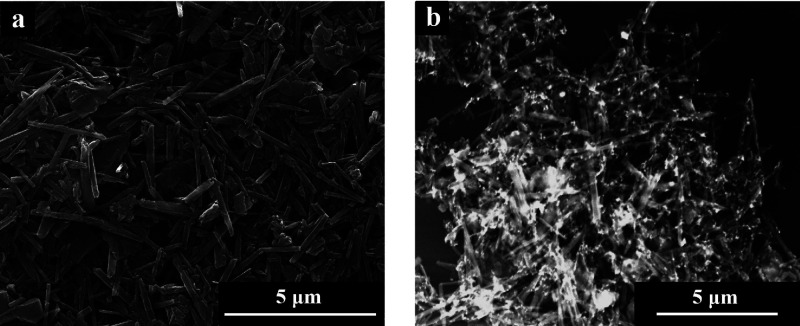
SEM micrographs of (a) pristine halloysite and (b) AEAPTMS functionalized halloysite with gold nanoparticles.

## Conclusions

The computational investigation reported in this work allowed to obtain insights about the properties and the stable geometries of gold subnanometric clusters after interaction with AEAPTMS-functionalized halloysite nanotubes. They shed light on the energetics of both cluster formation and the anchoring of preformed particles on the support surface. As usual, when dealing with subnanometer clusters, it is not possible to draw simple (or even complex) rules that determine the systematic variation of properties as a function of the size. Many cluster characteristics can change dramatically even with the addition of a single atom. In the systems investigated in this work, any possible systematicity for the anchoring geometry and the energetics is further compromised by the large number and changing nature of interactions between the cluster and the support, as well as between the various parts of the support itself. Interactions with the silanol groups of the modified halloysite, for example, can make the anchoring of larger clusters more exothermic, since these could better adapt to the surroundings than the planar ones. Furthermore, computational data obtained from simulated reactive processes indicate that in the considered size range, nanocluster coalescence occurs preferentially starting from medium-sized particles, where many metal atoms can benefit from an increase in the coordination number.

Direct comparison between computational and experimental results on gold-halloysite composite systems is truly difficult, as the two approaches are applied to different scales. However, they aim to address complementary issues: the former is for atomistic details; the latter gives ensemble properties. As a matter of fact, the evidence of the experimental investigations, conducted to characterize the attachment of gold nanoparticles on the functionalized halloysite, showed that the presence of the aminosilane AEAPTMS moiety is crucial for achieving a homogeneous decoration of the outer surface with Au spherical particles of about 12 nm in diameter. Notably, the nanoclay maintains its peculiar hollow nanotubular shape with two separate chemically different environments, i.e., the inner lumen and the external surface decorated with gold, thus making the resulting material promising for technological and catalytic applications, in particular for the one-pot conversions of biomass to highly relevant products.

## Supplementary Material



## Notes

Supporting Information containing DTG curves, the interaction energies between selected gold clusters and *N*-propylethylenediamine, the coordination numbers of gold atoms in the Au_*n*_/fHNT systems, the coalescence energy values for selected isolated Au clusters, the coalescence energy values corresponding to the process associated with reaction ([Disp-formula eq3]) in the main text, which were used to build the diagram in Figure, the optimized geometries (*xyz* format) of the entire Au_*n*_/fHNT systems (*n* = 1, 2, ··· , 20) and the detail of the model systems (*xyz* format) chosen for the Au_*n*_/fHNT aggregates (PDF)
