# Supplementary material for: Anchoring Gold Nanoparticles on Functionalized Halloysite Nanotubes: Density Functional Theory and Experimental Studies
Source: J Phys Chem C Nanomater Interfaces. 2025 Sep 9;129(37):16944–57. doi: 10.1021/acs.jpcc.5c05165 (PMC12451941; doi:10.1021/acs.jpcc.5c05165)
Supplement: Supplementary file 1 [file jp5c05165_si_001.pdf]

Supporting information for

Anchoring Gold Nanoparticles on Functionalized Halloysite Nanotubes: DFT and Experimental Studies

Ludovico Guercio,[a] Francesco Ferrante,*[a] Marco Bertini,[a] Chiara Ferlito,[a] Lorenzo Lisuzzo,[a] Giuseppe Lazzara,[a] Dario Duca[a]

[a] *Dipartimento di Fisica e Chimica "E. Segrè", Università degli Studi di Palermo, Viale delle Scienze - 90128 Palermo, Italy.*

In this supporting information the following is collected

- Figure S1: derivative thermogravimetry curves for the p-HNT, a-HNT and ga-HNT samples
- Table S1: interaction energies between selected gold clusters and N-propylethylenediamine
- Table S2: coordination numbers of gold atoms in the Au_n/fHNT systems
- Table S3: comparison between the formation energetics of selected isolated gold clusters with the involvement or not of the Au_2 species.
- Coalescence energy values corresponding to the process associated to reaction (3) in the main text, which were used to build the diagram in Figure 5
- Optimized geometries (xyz format) of the entire Au_n/fHNT systems ($n = 1, 2, \ldots, 20$)
- Detail of the model systems (xyz format) chosen for the Au_n/fHNT aggregates ($n = 1, 2, \ldots, 20$), without link atoms

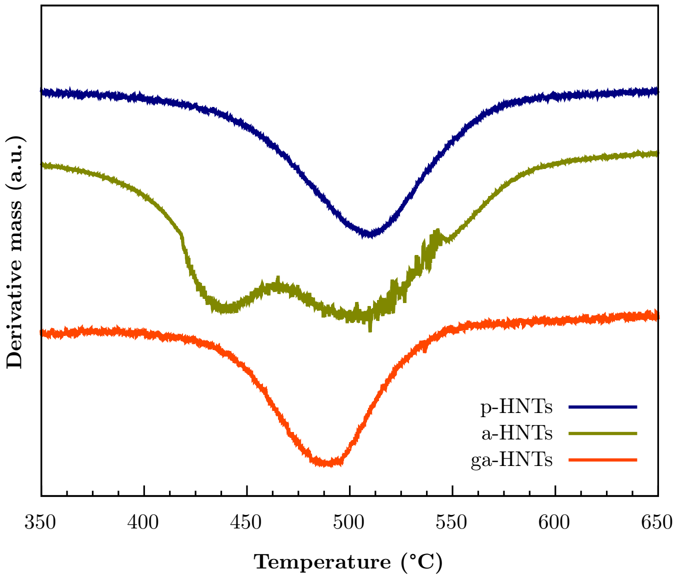

Figure S1: DTG curves of the pristine halloysite samples (p-HNTs), AEAPTMS-functionalized halloysite (a-HNTs) and functionalized halloysite decorated with gold nanoparticles (ga-HNTs).

Table S1: Reaction Energy Values (kJ mol^{-1}) Corresponding to the Formation of the Au$_n$–N-propylethylenediamine Complex and the Associated Basis Set Superposition Errors.

**n**		E$_a$	**BSSE**
5	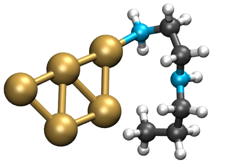	-126.5	18.4
9	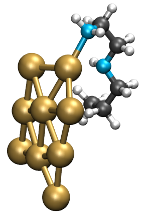	-131.5	26.7
10	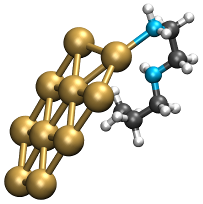	-129.4	26.7
15	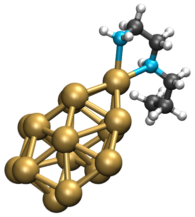	-123.7	26.9
20	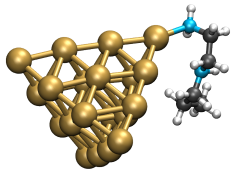	-119.1	21.6

Table S2: The Number of Atoms Having a Given Coordination Number in the Au_n Gold Cluster Anchored on the Functionalized Halloysite Nanotube Model. Two Gold Atoms Were Considered Bounded if Their Distance Is Smaller Than 3.4 Å.

**n**	1	2	3	4	5	6	7	8	9
**2**	2								
**3**		3							
**4**	1	2	1						
**5**		2	2	1					
**6**		3		3					
**7**		3	1	2	1				
**8**		4		4					
**9**		2	3	2	1	1			
**10**			6	2		2			
**11**			2	3		6			
**12**			3	2	1	4	2		
**13**			4	2		7			
**14**			4		6	1	2	1	
**15**				3	4	4	4		
**16**				6		6	4		
**17**				5	2	10			
**18**				1	10	5	2		
**19**			3		3	9			4
**20**			4			12			4

Table S3: Coalescence Energies (E_c kJ mol^{-1}) for a Selection of Isolated Gold Clusters[a] (Calculated with the Same Method Used for the Accurate Level of the ONIOM Approach), Corresponding to Processes in which Au_2 is Involved or Not.

**Au_{i+j}**	**(i,j)**	**E_c**	**(i,j)**	**E_c**
Au_6	(2,4)	-271.5	(3,3)	-400.7
Au_{10}	(2,8)	-213.6	(5,5)	-358.9
Au_{11}	(2,9)	-227.5	(5,6)	-248.0
Au_{12}	(2,10)	-227.0	(4,8)	-315.7
Au_{14}	(2,12)	-248.0	(7,7)	-417.0
			(4,10)	-349.7
Au_{17}	(2,15)	-305.7	(5,12)	-475.6
Au_{18}	(2,16)	-323.6	(9,9)	-612.8
Au_{19}	(2,17)	-280.1	(9,10)	-565.1
Au_{20}	(2,18)	-303.9	(10,10)	-596.6
			(11,9)	-689.2

[a]It should be noted that the coalescence energy values of the isolated clusters are much higher in magnitude than those related to the coalescence in the halloysitic systems; this is mainly because, for the same number of new Au−Au bonds that form following coalescence, in the case of the isolated clusters there are no Au−N bonds breaking.

Coalescence energies associated to the process

$\mathbf{Au_i/fHNT + Au_j/fHNT \longrightarrow Au_{i+j}/fHNT + fHNT}$

i+j	i	j	Ecoalescence, kJ/mol
2	1	1	-288.2
3	2	1	-84.3
4	3	1	-201.5
5	4	1	-126.3
6	5	1	-213.5
7	6	1	-123.0
8	7	1	-226.2
9	8	1	-114.8
10	9	1	-239.5
11	10	1	-140.6
12	11	1	-233.5
13	12	1	-157.3
14	13	1	-220.4
15	14	1	-179.5
16	15	1	-267.8
17	16	1	-194.4
18	17	1	-258.0
19	18	1	-166.8
20	19	1	-268.7
4	2	2	2.5
5	3	2	-39.6
6	4	2	-51.6
7	5	2	-48.3
8	6	2	-61.0
9	7	2	-52.8
10	8	2	-66.1
11	9	2	-91.9
12	10	2	-85.8
13	11	2	-102.5
14	12	2	-89.5
15	13	2	-111.8
16	14	2	-159.2
17	15	2	-174.0
18	16	2	-164.2
19	17	2	-136.6
20	18	2	-147.3
6	3	3	-168.8
7	4	3	-90.3

8	5	3	-190.2
9	6	3	-91.5
10	7	3	-208.0
11	8	3	-122.4
12	9	3	-241.0
13	10	3	-158.8
14	11	3	-238.7
15	12	3	-184.8
16	13	3	-295.3
17	14	3	-269.2
18	15	3	-347.7
19	16	3	-246.7
20	17	3	-321.0
8	4	4	-115.1
9	5	4	-103.5
10	6	4	-129.6
11	7	4	-147.2
12	8	4	-154.4
13	9	4	-196.9
14	10	4	-177.8
15	11	4	-216.8
16	12	4	-251.1
17	13	4	-288.2
18	14	4	-325.8
19	15	4	-313.0
20	16	4	-313.9
10	5	5	-216.7
11	6	5	-143.8
12	7	5	-254.3
13	8	5	-185.4
14	9	5	-291.0
15	10	5	-231.0
16	11	5	-358.3
17	12	5	-319.1
18	13	5	-419.9
19	14	5	-366.2
20	15	5	-455.4
12	6	6	-163.8
13	7	6	-198.1
14	8	6	-192.3
15	9	6	-257.0
16	10	6	-285.4
17	11	6	-339.2
18	12	6	-363.7
19	13	6	-373.2

20	14	6	-421.5
14	7	7	-295.5
15	8	7	-248.9
16	9	7	-401.9
17	10	7	-356.7
18	11	7	-474.2
19	12	7	-407.5
20	13	7	-518.9
16	8	8	-290.5
17	9	8	-370.0
18	10	8	-388.5
19	11	8	-414.7
20	12	8	-450.0
18	9	9	-513.2
19	10	9	-440.5
20	11	9	-568.7
20	10	10	-469.7

Optimized geometries of the entire Au_n/fHNT systems

Au/fHNT

```
 304
O    -6.530951    -5.705339    -4.349873
O    -7.801838    -3.055992    -4.657142
O    -3.579749    -8.411135     0.275950
O    -8.208874    -5.502323    -0.478306
O    -5.872244    -6.502217    -2.037524
O    -0.162367     9.507980    -2.378472
O   -10.263880    -1.489535    -1.056582
O     3.677063     8.452303     0.058456
O    -2.646031     9.891495     1.518296
O     1.680083     9.655619     1.904903
O     1.987275    -9.397028    -1.272252
O     9.501632    -2.343732    -1.087030
O     0.959807    -8.601048     1.092436
O    -2.384940    -8.219412    -3.723214
O    -1.206711    -9.330406    -1.637305
O     6.139176    -5.718066     1.588592
O     9.252733     1.197293    -3.183075
O     9.434735    -3.650009    -3.242414
O     7.811520    -6.830896    -2.870075
O     6.701909    -7.068701    -0.721096
O     3.544981    -8.585368    -3.144093
O   -10.101644     3.683491    -0.601609
O    -7.502395     7.545598     0.288259
O    -7.561057     2.253815    -4.492203
O     8.216152    -2.825673     1.308018
O     8.504099     2.320976     1.445815
O     6.239550     6.994411     1.676410
O     8.025893     5.697443    -0.470644
O     6.682606     6.536362    -2.460456
O    -4.555356     7.040202    -3.444446
O     9.669342     2.556149    -0.964171
O     2.684497     9.332803    -2.100041
H     8.898741     0.280485    -3.219456
H     9.483148    -3.136530    -4.057461
H    -7.410361     2.914522    -5.180928
H    -7.722725    -2.539911    -5.468957
H     7.318527    -7.330527    -3.531024
H    -0.597818     9.419097    -3.234731
H     5.722043     6.773920    -2.368967
```

H	-3.042471	-7.481678	-3.709846
H	3.621386	-9.257436	-2.443259
H	-4.796823	6.860354	-4.361548
H	1.751876	9.617426	-2.166981
H	-7.176295	-4.984048	-4.469585
H	8.761373	5.221223	-0.080123
H	-1.792955	-9.478546	-0.886075
H	4.496973	8.105733	0.425107
H	-5.321328	-7.068551	-1.494480
H	-10.620319	-1.189633	-0.216114
H	-3.938018	-8.642249	1.137945
H	1.494196	-9.034978	0.381610
H	6.613652	-5.295187	2.312968
H	-9.007706	-5.251742	-0.004611
H	8.903911	-2.679326	0.611333
H	9.139121	2.462915	0.694656
H	-10.511874	2.822335	-0.712725
H	-8.396865	7.456012	0.648150
H	-3.586903	9.783740	1.684244
H	2.598976	9.609276	1.587356
H	7.107483	7.068871	1.248779
H	9.966198	3.432741	-1.228208
H	1.144361	-9.864888	-1.359674
H	6.712855	-6.879181	0.232447
H	9.536261	-1.379413	-1.049215
H	10.036452	-3.182457	-2.609950
H	7.718637	-7.364541	-2.030877
H	4.231402	-7.901702	-2.968372
H	-2.790806	-8.971950	-3.267165
H	-6.948652	-6.369668	-3.767497
H	-8.505887	-2.617337	-4.119971
H	-8.302408	2.629317	-3.959921
H	-5.355177	7.408246	-3.006724
H	-0.884709	9.611883	-1.702893
H	3.085698	9.817229	-1.356613
H	7.174923	7.104302	-1.847907
H	10.026006	1.164660	-2.594181
H	-3.858681	0.726326	-0.452876
H	0.918530	-0.964933	2.253508
H	0.652332	3.329521	0.294064
Al	-5.256440	-5.050602	-2.880822
O	-5.144881	-3.376129	-3.879880
Al	-6.462017	-2.311941	-3.070936
O	-3.976953	-4.491134	-1.560392
Al	-2.352248	-5.078755	-2.375894

O	-2.385069	-6.751170	-1.424542
Si	-2.706398	-7.016811	0.171068
O	-6.676260	-3.972622	-2.194529
Si	-7.211869	-4.219718	-0.642583
O	-3.665404	-5.724835	-3.684905
Si	-4.283909	-4.484508	0.068039
O	-3.585709	-3.180406	0.737396
Si	-2.543980	-1.897520	0.572657
O	-3.496320	-0.590151	0.955429
O	-5.891145	-4.386701	0.320230
O	-3.642933	-5.813713	0.767145
O	-8.088812	-2.900724	-0.201120
Si	-8.616941	-1.586820	-1.057249
O	-8.062217	-0.277793	-0.246879
Si	-7.040548	0.985352	-0.332705
O	-6.459855	1.140827	-1.901361
Al	-6.290459	2.812173	-2.779613
O	-5.897017	4.460048	-3.718694
Al	-4.788900	5.226450	-2.322551
O	-3.198329	4.633256	-3.128615
Al	-1.888742	5.094527	-1.815270
O	-0.751493	5.878819	-0.530442
Al	-0.315788	7.478303	-1.369583
O	-8.109000	-1.688391	-2.591233
O	-6.080480	-0.699578	-4.032773
Al	-5.010564	0.154617	-2.661636
O	-3.732008	0.735329	-1.414499
Al	-2.130086	-0.015937	-2.067287
O	-0.996267	0.815811	-0.811739
Al	-0.603213	2.420021	-1.693415
O	0.801174	2.939946	-0.580543
Al	2.334336	2.392320	-1.466605
O	0.946835	1.689313	-2.614016
O	-5.439554	-1.465227	-1.733543
O	-3.427260	-0.620813	-3.370792
O	-4.880620	1.841263	-3.644602
O	-1.779233	1.651029	-2.976277
Al	-0.795618	-2.657207	-2.028634
O	-2.187028	-1.668055	-1.059136
O	-0.827502	-1.009568	-2.997113
O	-0.518355	4.097081	-2.676000
O	-1.977230	3.441262	-0.749742
Si	-2.153016	3.442125	0.915216
O	-1.459826	6.755502	-2.691725
O	-3.502207	5.813267	-1.058234

Si	-3.837533	5.982379	0.566899
O	-5.448492	5.990596	0.798574
Si	-6.765699	6.163660	-0.196986
O	-6.178186	6.229193	-1.713419
O	1.047983	8.082020	-0.228275
Al	2.636552	7.545538	-1.116072
O	-1.594406	8.549629	-0.612839
Si	-1.994925	8.483596	0.961465
O	1.225808	7.045072	-2.336377
Si	0.971924	8.255095	1.425588
O	-0.611204	8.199863	1.838667
O	1.749818	6.933508	2.066114
Si	2.634232	5.721525	1.427620
O	2.682746	5.849162	-0.220775
Al	3.847425	4.875127	-1.359239
O	3.906508	6.646392	-2.225085
O	-3.123031	7.299524	1.216675
O	-3.255817	4.631665	1.298019
O	-0.683684	3.928774	1.472660
O	-1.215536	-4.260194	-1.092683
O	-2.049985	-3.423822	-3.250484
O	-0.962009	-6.008209	-3.225290
Al	-0.968070	-7.708336	-2.351753
O	0.390173	-7.088685	-1.119747
Al	1.899740	-7.775180	-2.099258
O	0.576247	-2.146185	-0.878221
Al	2.133374	-2.712952	-1.713332
O	3.395951	-1.836206	-0.598494
Al	3.692483	-0.234233	-1.586714
O	3.686441	1.463058	-2.479464
O	0.776984	-3.354965	-2.919470
O	2.292879	-4.358322	-0.734418
Al	3.535134	-5.352432	-1.805417
O	3.186629	-6.968189	-0.909286
O	3.565696	-3.624485	-2.742466
O	2.324312	-1.018338	-2.624608
Si	2.052232	-4.690003	0.872803
O	3.552894	-5.014142	1.488497
Si	5.038914	-4.695476	0.909155
O	5.499148	-3.155639	1.286540
Si	6.780924	-2.210141	0.846897
O	6.495083	-0.749390	1.550916
Si	5.371658	0.353566	1.097061
O	5.221947	0.263338	-0.544198
Al	6.578007	-0.379864	-1.749474

O	7.764739	-1.334207	-2.975582
Al	7.966700	-2.972946	-1.928286
O	1.400692	-3.423657	1.677753
O	0.987906	-5.876734	1.180237
Si	0.290279	-7.235419	0.527075
O	0.553900	-8.213330	-3.363294
O	-1.291810	-7.126168	0.982724
O	5.036680	-4.869213	-0.723682
Al	6.518208	-5.583650	-1.741294
O	6.652041	-3.992766	-2.862987
O	5.007184	-6.141948	-2.829978
O	2.238949	-6.081718	-2.943519
O	5.040199	-0.937714	-2.707159
O	2.501190	0.753708	-0.440693
Si	2.372415	0.513232	1.232749
O	6.759717	-2.014989	-0.796554
O	8.032966	0.453731	-0.864116
Al	8.079012	2.148373	-1.778709
O	6.662444	1.254602	-2.686132
O	7.921795	-4.582665	-0.981796
O	-5.795287	0.841933	0.722779
O	-7.875841	2.318223	0.086035
Si	-8.448218	3.624279	-0.746964
O	-7.928047	3.460495	-2.289511
O	-7.887695	4.977617	-0.015673
O	-5.207182	3.591582	-1.469573
O	5.841990	1.841717	1.565022
Si	7.020257	2.903440	1.079927
O	3.896499	0.044562	1.747667
O	6.566069	4.301097	1.815905
Si	5.616881	5.538439	1.283886
O	6.936239	3.088827	-0.563938
Al	6.818450	4.780272	-1.473803
O	4.154325	5.567699	2.025692
O	5.372461	5.358707	-0.338555
O	1.891650	4.325303	1.886420
O	1.342685	-0.804715	1.388733
O	3.613959	3.251216	-0.351256
O	2.494556	4.065271	-2.416325
O	5.244672	4.284238	-2.448707
O	7.917656	3.807953	-2.665307
H	1.196045	6.451514	-3.087971
H	1.006460	1.922286	-3.544900
H	0.806569	-3.145718	-3.856937
H	0.548186	-9.134831	-3.639784

H	5.173813	3.720792	-3.218702
H	4.820157	-1.874215	-2.815674
H	5.032273	-5.874317	-3.755020
H	-3.251063	3.853377	-3.686862
H	-3.148461	-0.532951	-4.285221
H	-3.497711	-5.465048	-4.596106
H	8.115256	4.015025	-3.578896
H	7.288976	-1.416328	-3.811356
H	-0.672500	4.023096	-3.622898
H	-0.068336	-0.528804	-3.333103
H	-0.651845	-5.910583	-4.126673
H	5.788636	1.671858	-2.617358
H	5.754953	-3.634203	-2.929843
H	-2.296729	7.219349	-2.829822
H	-2.661215	2.051062	-3.003910
H	-2.801555	-2.813236	-3.295413
H	3.688986	6.656751	-3.162944
H	3.524890	1.356073	-3.422123
H	3.285250	-3.733984	-3.658871
H	-4.940355	1.754410	-4.600686
H	-4.494698	-3.053220	-4.505197
H	1.604086	4.451574	-2.357218
H	1.537584	-0.481373	-2.441729
H	1.420704	-5.559045	-2.989128
H	-6.725984	4.948233	-3.594166
H	-6.896449	-0.191690	-4.137391
H	8.311290	0.402768	0.059195
H	8.460682	-4.838059	-0.229605
H	-0.291777	5.407276	0.177838
H	-0.397560	0.341733	-0.225341
H	-0.454717	-4.796363	-0.830843
H	4.445732	2.766803	-0.244128
H	4.105991	-2.293637	-0.132022
H	3.815917	-7.455107	-0.367176
H	-4.517635	3.037823	-1.077834
H	-4.656843	-1.956388	-1.447355
H	-0.311432	3.627542	2.315929
H	-3.731162	-0.455662	1.889589
H	1.808860	-2.551392	1.555665
H	2.332787	3.557994	1.486432
H	-5.330446	-0.007426	0.714031
H	0.483010	-1.929344	0.059086
Si	1.834495	1.508570	3.838648
O	1.611113	1.694051	2.203253
O	1.271730	0.095145	4.465897

O	0.911822	2.768690	4.483976
O	3.412995	1.651953	4.318124
H	1.349925	3.203542	5.224911
H	3.935538	2.273327	3.799680
Si	-2.336067	1.741991	3.236643
O	-2.627516	1.934086	1.596447
O	-1.135574	0.633016	3.459842
O	-3.667998	0.962954	3.896611
O	-2.000714	3.102071	4.109195
H	-4.533238	1.307957	3.650220
H	-1.061143	3.132818	4.372572
Si	-1.709400	-2.495633	3.210201
O	-1.231751	-2.032094	1.670552
O	-0.629827	-1.831981	4.300193
O	-1.672383	-4.146980	3.354800
O	-3.250002	-2.107007	3.673694
H	-1.006940	-4.573582	2.805343
H	-3.402800	-1.174269	3.904224
Si	-0.365802	-0.226051	4.663085
C	-0.744985	0.215023	6.423996
H	-0.303823	1.221784	6.542798
H	-0.077729	-0.449152	7.012637
C	-2.148535	0.201959	7.025946
H	-2.045415	0.381638	8.111742
H	-2.742118	1.037677	6.613778
C	-2.947437	-1.072474	6.814340
H	-3.392708	-1.075280	5.799679
H	-3.799645	-1.086996	7.517905
N	-2.180109	-2.292575	7.031611
C	-2.979893	-3.496921	6.814201
H	-1.395744	-2.324784	6.372968
C	-2.124919	-4.745624	6.749409
H	-3.554393	-3.415160	5.867894
H	-3.714149	-3.572400	7.640611
H	-2.784509	-5.585896	6.443541
H	-1.395891	-4.625720	5.926100
N	-1.399651	-4.965884	7.988193
H	-2.046626	-5.188947	8.746177
H	-0.764222	-5.757966	7.905347
Au	-1.099049	-2.282461	9.274088

Au_2/fHNT

305

O	10.138815	-0.047377	-1.771537
O	9.408799	-2.832731	-2.432951
O	8.502649	3.739059	2.746765
O	10.108181	-1.511850	2.190793
O	9.539167	0.882310	0.507052
O	-4.780082	-6.861103	-4.396395
O	9.126292	-5.814644	1.103695
O	-7.499463	-3.573613	-2.501222
O	-4.364143	-9.009073	-0.308158
O	-7.383411	-5.896302	-0.647322
O	5.633888	8.331230	0.501149
O	-4.308567	8.323034	-2.218472
O	5.181723	6.945471	2.771883
O	8.655509	4.591256	-1.340778
O	7.955054	6.114870	0.699197
O	-0.492980	8.364964	1.691861
O	-5.837562	5.665155	-4.973850
O	-2.809636	9.323113	-3.981871
O	0.295885	10.514044	-2.629479
O	0.632159	9.834595	-0.320550
O	4.530712	8.886916	-1.749020
O	5.532474	-9.491810	0.357724
O	0.940003	-10.566844	-0.108448
O	5.752209	-6.543143	-3.501335
O	-3.758702	7.690404	0.411813
O	-7.335368	4.132290	-0.654516
O	-8.824021	-0.830392	-1.076511
O	-8.645815	1.429642	-3.160608
O	-7.679211	-0.012846	-5.018688
O	0.228806	-8.020455	-4.093880
O	-7.636035	4.863100	-3.222914
O	-6.760775	-4.799213	-4.592118
H	-4.982745	6.092543	-4.741897
H	-2.947650	9.017832	-4.886335
H	5.410901	-6.891254	-4.335547
H	9.246446	-3.119057	-3.340313
H	1.154276	10.569835	-3.064866
H	-4.173669	-7.056633	-5.120774
H	-7.178583	-0.846653	-4.814660
H	8.639469	3.603722	-1.376595
H	4.715154	9.398051	-0.940685
H	0.773869	-8.014427	-4.890491
H	-6.265929	-5.641274	-4.555403
H	10.162170	-1.008784	-1.933416

H	-8.967809	2.262311	-2.809615
H	8.255179	5.789090	1.555798
H	-7.958534	-2.776614	-2.216916
H	9.363401	1.647768	1.056898
H	8.948377	-6.313568	1.905439
H	8.663976	3.624744	3.687892
H	5.284036	7.658028	2.092957
H	-1.306362	8.349650	2.208432
H	10.378507	-2.261282	2.729854
H	-4.144877	8.084520	-0.409982
H	-7.666045	4.495926	-1.518261
H	6.412045	-9.140137	0.515530
H	1.530374	-11.128896	0.414223
H	-3.674672	-9.581134	0.040791
H	-7.914913	-5.220390	-1.103387
H	-9.366781	-0.272502	-1.656067
H	-8.339963	4.439085	-3.723854
H	6.558433	8.099543	0.670219
H	0.233831	9.662317	0.549474
H	-4.968399	7.642612	-2.403361
H	-3.716241	9.366268	-3.585803
H	0.471668	10.802548	-1.689522
H	3.552390	8.850544	-1.853977
H	9.302341	4.841980	-0.664191
H	10.701405	0.125472	-0.991415
H	9.472332	-3.657130	-1.891680
H	5.884448	-7.346825	-2.944090
H	0.433841	-8.854555	-3.615314
H	-4.525609	-7.460228	-3.644547
H	-7.567486	-4.910300	-4.058083
H	-8.567783	-0.116741	-4.644818
H	-6.529397	6.191865	-4.538246
H	2.985491	-3.075775	0.059511
H	-0.061904	1.304096	2.189497
H	-2.105091	-1.920416	-0.599880
Al	8.399550	0.282841	-0.733699
O	7.516394	-0.816912	-2.084620
Al	7.532534	-2.528041	-1.314224
O	6.760243	0.692333	0.182007
Al	6.219407	2.266925	-0.755154
O	7.058874	3.421107	0.535693
Si	7.010387	3.325110	2.181362
O	8.513920	-1.503174	-0.065869
Si	8.617414	-1.757279	1.571560
O	7.935483	1.896978	-1.634664

Si	6.515912	0.406617	1.795950
O	4.988814	-0.095706	2.023688
Si	3.469369	-0.312890	1.393902
O	3.186103	-1.930629	1.639418
O	7.519930	-0.774748	2.299145
O	6.726793	1.782330	2.649610
O	8.259968	-3.336968	1.855229
Si	8.023238	-4.617701	0.834495
O	6.554819	-5.227405	1.221761
Si	5.037158	-5.445225	0.677738
O	4.965774	-5.092830	-0.963230
Al	4.009759	-6.156047	-2.207824
O	2.927374	-7.046325	-3.544986
Al	1.254079	-6.908022	-2.572629
O	0.738395	-5.353150	-3.493489
Al	-0.856641	-4.851220	-2.567975
O	-2.530898	-4.701162	-1.711459
Al	-3.639930	-5.531662	-2.949459
O	8.160155	-4.129320	-0.703094
O	6.487965	-3.399594	-2.663184
Al	4.791875	-3.350272	-1.727647
O	3.160034	-2.953659	-0.886778
Al	2.695632	-1.282560	-1.627445
O	1.001326	-1.168169	-0.808968
Al	-0.068688	-2.029430	-2.081323
O	-1.712485	-1.496610	-1.377767
Al	-2.194589	-0.010682	-2.375359
O	-0.434354	-0.397063	-3.074152
O	5.884369	-2.504239	-0.401003
O	4.372175	-1.671866	-2.513675
O	3.883290	-4.447030	-3.069097
O	1.622607	-2.217210	-2.932502
Al	3.450822	1.552097	-1.236572
O	3.523223	-0.162147	-0.282345
O	2.678066	0.372486	-2.526893
O	-0.939190	-3.150022	-3.412282
O	-0.022156	-3.754035	-1.162091
Si	-0.367005	-3.948139	0.464314
O	-1.990227	-5.729520	-3.854267
O	-0.392341	-6.511037	-1.718088
Si	-0.720630	-6.934608	-0.138627
O	0.349585	-8.052149	0.365600
Si	1.449829	-9.035277	-0.395339
O	1.417227	-8.614980	-1.967388
O	-5.317304	-5.088912	-2.230215

Al	-5.845362	-3.573145	-3.241723
O	-3.641038	-7.219373	-2.237495
Si	-3.756771	-7.513982	-0.642810
O	-4.179429	-4.119768	-4.051460
Si	-5.840051	-5.339334	-0.669750
O	-4.799196	-6.399596	0.017614
O	-5.714656	-3.872806	0.101969
Si	-5.376485	-2.357343	-0.395782
O	-5.030578	-2.345039	-2.013197
Al	-4.904609	-0.789404	-3.092761
O	-5.850721	-2.001371	-4.328305
O	-2.262536	-7.433953	0.064757
O	-0.463401	-5.584924	0.761131
O	-1.878086	-3.321751	0.631827
O	4.523799	2.391719	0.092261
O	5.178906	1.306337	-2.015995
O	6.075589	3.931663	-1.607264
Al	6.935908	5.127727	-0.389557
O	5.226665	5.551118	0.414074
Al	4.877486	7.126150	-0.637991
O	1.825248	2.067512	-0.489329
Al	1.324281	3.581543	-1.438026
O	-0.450868	3.757372	-0.787201
Al	-1.421068	2.836430	-2.144146
O	-2.265613	1.634992	-3.377527
O	3.039442	3.174562	-2.212211
O	2.002295	4.846305	-0.160690
Al	2.067389	6.466661	-1.184762
O	3.109213	7.366144	0.096135
O	1.189530	5.269856	-2.473877
O	0.347214	2.517542	-2.723273
Si	1.936196	4.853071	1.496616
O	0.910569	6.088287	1.894726
Si	-0.185465	6.897529	1.006319
O	-1.614884	6.074033	0.943644
Si	-3.011597	6.280908	0.085396
O	-3.953201	4.990622	0.485008
Si	-3.744709	3.438941	0.001909
O	-3.119295	3.474101	-1.525162
Al	-3.324402	4.922520	-2.776282
O	-3.202185	6.482728	-3.948147
Al	-2.577736	7.768701	-2.614200
O	1.351150	3.449949	2.100799
O	3.372314	4.976373	2.243587
Si	4.929946	5.517665	2.043304

O	6.469373	6.580430	-1.514992
O	5.851546	4.336446	2.734623
O	0.387248	7.093982	-0.520034
Al	0.086730	8.671873	-1.597457
O	-0.723613	7.653598	-3.051235
O	1.824176	8.093058	-2.250437
O	3.777342	6.161449	-1.884856
O	-1.605071	4.319336	-3.299038
O	-1.539373	1.252118	-1.055800
Si	-1.762414	1.263050	0.626202
O	-2.661447	6.196149	-1.530059
O	-5.143371	5.271364	-2.369753
Al	-6.016934	4.108421	-3.632640
O	-4.179955	3.830629	-4.053671
O	-1.768960	8.869163	-1.339869
O	3.951813	-4.535515	1.502202
O	4.647607	-7.005555	0.929193
Si	4.440798	-8.311835	-0.059824
O	4.611771	-7.769399	-1.593902
O	2.961781	-8.946394	0.240726
O	2.369808	-6.040436	-1.316540
O	-5.173224	2.656033	0.035809
Si	-6.559172	2.709469	-0.873700
O	-2.682631	2.626970	0.954555
O	-7.349661	1.348640	-0.399703
Si	-7.329644	-0.178381	-1.018421
O	-6.158098	2.588893	-2.476291
Al	-6.914889	1.316063	-3.705061
O	-6.521243	-1.232128	-0.055975
O	-6.584472	-0.143604	-2.490781
O	-4.074996	-1.868075	0.486324
O	-0.223177	1.515201	1.249906
O	-3.970380	0.189789	-1.723075
O	-3.125065	-1.078222	-3.687220
O	-5.205292	0.644008	-4.251779
O	-6.728769	2.828005	-4.824318
H	-3.562753	-3.674871	-4.634446
H	-0.366089	-0.485288	-4.029103
H	3.146225	3.083379	-3.162879
H	7.148034	7.259873	-1.573584
H	-4.573857	1.039673	-4.851878
H	-0.812094	4.855811	-3.155471
H	1.892824	7.955724	-3.201430
H	1.437729	-4.796701	-3.845506
H	4.374642	-1.505245	-3.459118

H	7.904344	1.862251	-2.595770
H	-6.746262	2.852231	-5.781228
H	-2.577353	6.253811	-4.647285
H	-0.516163	-3.160100	-4.276467
H	1.923392	0.556035	-3.090032
H	6.045854	4.112043	-2.547898
H	-3.850801	2.926238	-3.926788
H	-0.301857	6.781975	-3.037197
H	-1.659290	-6.633571	-3.942607
H	1.995795	-3.110419	-2.892758
H	5.328195	0.349671	-2.062770
H	-5.440003	-2.116615	-5.191448
H	-1.817167	1.643698	-4.228981
H	1.716430	5.197962	-3.278642
H	4.250367	-4.382778	-3.955846
H	7.022404	-0.580230	-2.870742
H	-2.761346	-1.971033	-3.559766
H	0.505108	1.580496	-2.529028
H	4.030989	5.223254	-1.900931
H	3.163172	-7.974158	-3.388362
H	6.766152	-4.322875	-2.732937
H	-5.566766	5.458390	-1.522207
H	-2.196377	9.390745	-0.656727
H	-2.749882	-4.074339	-1.008258
H	0.719774	-0.439132	-0.246556
H	4.258832	3.291021	0.328777
H	-4.275668	1.106655	-1.658939
H	-0.788350	4.555641	-0.363104
H	2.826772	8.127367	0.613565
H	2.130226	-5.182744	-0.938993
H	5.567902	-1.623818	-0.155034
H	-2.183545	-2.885041	1.442055
H	3.003074	-2.230034	2.546493
H	0.531330	3.098969	1.717300
H	-3.777972	-0.990037	0.196192
H	4.175323	-3.598616	1.601301
H	1.487560	1.804813	0.377396
Si	-2.821308	0.042481	2.969976
O	-2.235900	-0.168697	1.431834
O	-1.683533	0.626035	4.072178
O	-3.141205	-1.529940	3.481152
O	-4.116829	1.041430	2.968958
H	-4.053718	-1.652441	3.804534
H	-4.309406	1.560286	3.786143
Si	0.188620	-2.903657	3.087802

```
 O     0.752319    -3.205335     1.538935
 O     0.016428    -1.276237     3.306000
 O     1.439985    -3.273877     4.145873
 O    -1.176630    -3.677374     3.587850
 H     1.925386    -4.086716     3.967866
 H    -1.947887    -3.077033     3.644322
Si     2.492686     0.595000     3.898529
 O     2.317073     0.637695     2.230192
 O     0.964456     0.917472     4.498465
 O     3.559357     1.767525     4.376624
 O     3.055071    -0.787366     4.603195
 H     3.600316     2.509946     3.765546
 H     2.486359    -1.576734     4.588764
Si    -0.279007    -0.182205     4.521317
 C    -0.352034    -0.977632     6.213921
 H     0.124323    -1.964267     6.055047
 H     0.371576    -0.414501     6.832675
 C    -1.657848    -1.173557     6.987044
 H    -2.427936    -1.658005     6.356255
 H    -1.467938    -1.877761     7.814139
 C    -2.234136     0.093540     7.601880
 H    -1.412751     0.708715     8.026265
 H    -2.911485    -0.155349     8.436905
 N    -3.003580     0.900331     6.635659
 C    -3.316970     2.250413     7.124988
 H    -2.448424     0.997334     5.772275
 C    -3.563110     3.233598     5.998400
 H    -4.190000     2.192469     7.801409
 H    -2.472105     2.636590     7.728779
 H    -3.725736     4.227221     6.460627
 H    -2.641459     3.315678     5.392083
 N    -4.646992     2.824683     5.108961
 H    -5.497656     2.632315     5.644625
 H    -4.881860     3.591305     4.476416
Au    -6.706949    -1.714686     5.003905
Au    -4.789151    -0.264326     5.955505
```

Au_3/fHNT

```
  306
 O     1.062345    -9.291871    -3.442543
 O    -1.460719    -8.292890    -4.610773
 O     3.645075    -8.676408     2.069258
```

O	-1.333467	-9.843229	-0.007169
O	1.411518	-9.143859	-0.938130
O	-4.633826	6.241484	-4.911820
O	-5.217486	-8.432418	-1.900289
O	-1.868761	8.396560	-1.828898
O	-7.730708	5.270989	-1.600029
O	-4.578615	8.109658	-0.637406
O	8.698264	-5.753009	1.540420
O	9.533428	4.488149	0.721876
O	6.803268	-5.608296	3.455903
O	5.476396	-8.186959	-1.654224
O	6.461653	-7.935447	0.782892
O	8.543418	0.070614	3.780704
O	7.664221	6.619619	-2.270982
O	10.911433	3.251282	-0.989201
O	11.677988	-0.104701	0.031230
O	10.444296	-0.784895	2.011437
O	9.810832	-4.320830	-0.276195
O	-8.534190	-4.547857	-2.850696
O	-9.381278	0.113905	-2.731705
O	-4.729018	-4.290022	-5.865566
O	8.262132	3.540639	2.981736
O	5.139505	7.455292	1.751113
O	0.460810	9.292131	0.425115
O	3.161642	9.332307	-1.053701
O	2.205513	8.783284	-3.345406
O	-5.918589	1.333302	-5.797075
O	6.486353	8.141246	-0.470740
O	-2.553358	8.097973	-4.250010
H	8.006309	5.713555	-2.100114
H	10.841123	3.558587	-1.900752
H	-4.854643	-3.792029	-6.683985
H	-1.511525	-7.962310	-5.516187
H	11.824570	-0.878843	-0.524400
H	-4.655114	5.779362	-5.758387
H	1.338594	8.306863	-3.440152
H	4.528633	-8.105215	-1.922258
H	10.103513	-4.670022	0.584667
H	-5.725709	0.931516	-6.653278
H	-3.386907	7.656053	-4.505343
H	0.170321	-9.229137	-3.831650
H	3.887455	9.539173	-0.461574
H	5.929499	-8.355840	1.468502
H	-1.158677	8.751717	-1.284433
H	2.020626	-9.110403	-0.198528

H	-5.895591	-8.362768	-1.223021
H	3.299379	-8.987385	2.911039
H	7.659397	-5.637314	2.960561
H	8.415260	0.784292	4.415461
H	-2.197271	-10.154884	0.279212
H	8.853405	4.035643	2.361114
H	5.710630	7.904654	1.073228
H	-8.247601	-5.461141	-2.773151
H	-10.065025	-0.521175	-2.473296
H	-8.383017	4.568415	-1.527519
H	-3.801988	8.668656	-0.815488
H	1.153737	9.890251	0.102911
H	6.211264	8.944173	-0.924470
H	8.416042	-6.677053	1.480821
H	10.069669	-0.529790	2.871590
H	8.931176	5.209795	0.500380
H	10.871266	4.073504	-0.438748
H	11.722639	-0.454139	0.965871
H	9.818431	-3.338471	-0.210065
H	5.541237	-8.952929	-1.064213
H	1.027656	-9.987758	-2.757335
H	-2.394129	-8.396947	-4.303263
H	-5.647484	-4.465634	-5.550144
H	-6.848385	1.101239	-5.577035
H	-5.404206	5.900302	-4.383526
H	-2.778750	8.807944	-3.622743
H	2.028021	9.600440	-2.854608
H	8.079115	7.194542	-1.605303
H	-2.199698	-2.381083	-1.141645
H	1.571550	-0.009330	2.485853
H	-0.829362	2.668079	-0.583273
Al	1.156463	-7.776528	-2.061923
O	0.439379	-6.612503	-3.456651
Al	-1.408921	-6.654919	-3.134585
O	1.355796	-6.343660	-0.796721
Al	3.122000	-5.748606	-1.216799
O	3.907402	-6.862797	0.141459
Si	3.409299	-7.087679	1.697448
O	-0.740809	-7.893680	-1.873261
Si	-1.392694	-8.257088	-0.390217
O	2.950742	-7.265509	-2.451702
Si	0.684896	-6.359054	0.718421
O	0.168883	-4.864823	1.088791
Si	0.138953	-3.249334	0.708275
O	-1.483328	-2.915239	0.604548

O	-0.601127	-7.360165	0.735899
O	1.803838	-6.794345	1.825183
O	-2.987105	-7.857631	-0.434257
Si	-3.972198	-7.374382	-1.672928
O	-4.633046	-5.958403	-1.186865
Si	-4.683617	-4.360209	-1.486711
O	-3.936169	-4.033752	-2.955315
Al	-4.642813	-2.818249	-4.226783
O	-5.156860	-1.473274	-5.522526
Al	-5.233705	-0.000110	-4.262203
O	-3.491038	0.568647	-4.675942
Al	-3.205336	1.950700	-3.387144
O	-3.242191	3.443687	-2.234373
Al	-3.722317	4.794477	-3.416391
O	-3.122092	-7.278572	-3.047552
O	-1.902574	-5.346278	-4.443850
Al	-2.056149	-3.839061	-3.235918
O	-1.850984	-2.400108	-2.046561
Al	-0.041077	-1.919359	-2.269670
O	-0.102721	-0.398004	-1.158153
Al	-0.604855	0.922021	-2.387285
O	-0.233643	2.387769	-1.294188
Al	1.460548	2.940646	-1.803687
O	1.228033	1.350740	-2.877969
O	-1.582555	-5.189529	-1.962635
O	-0.228706	-3.395033	-3.508557
O	-2.772234	-2.651803	-4.616333
O	-0.606163	-0.586305	-3.547075
Al	2.596091	-2.900341	-1.352279
O	0.698466	-3.028815	-0.865592
O	1.784777	-1.850284	-2.728062
O	-1.347275	2.071530	-3.770244
O	-2.503449	0.825464	-1.931926
Si	-3.086661	0.900254	-0.368761
O	-3.719442	3.336701	-4.622317
O	-5.031307	1.450813	-3.057106
Si	-5.825596	1.532506	-1.592674
O	-7.051376	0.461988	-1.570929
Si	-7.835335	-0.431977	-2.729531
O	-7.039763	-0.159455	-4.123034
O	-3.441666	6.295996	-2.323888
Al	-1.714313	6.894641	-2.831279
O	-5.533214	4.777488	-3.141388
Si	-6.210018	4.639434	-1.669623
O	-2.073103	5.425982	-4.032988

Si	-4.060158	6.561433	-0.801371
O	-5.275074	5.485558	-0.585830
O	-2.831694	6.218569	0.264125
Si	-1.246374	5.878187	0.091943
O	-0.841490	5.810786	-1.510358
Al	0.929936	5.775141	-2.190133
O	0.076755	6.988393	-3.490389
O	-6.332868	3.044974	-1.242575
O	-4.744212	1.044801	-0.456793
O	-2.495156	2.321539	0.215617
O	3.063280	-4.231535	-0.074948
O	2.520266	-4.453473	-2.464059
O	4.947724	-5.563955	-1.605118
Al	5.791371	-6.688827	-0.310340
O	6.033010	-5.169052	0.863747
Al	7.824687	-4.743112	0.299866
O	2.939354	-1.458867	-0.224904
Al	4.649059	-0.897402	-0.677631
O	4.689653	0.728072	0.303099
Al	4.148821	1.967939	-1.038943
O	3.303558	3.080238	-2.352780
O	4.415957	-2.428974	-1.820745
O	5.547728	-1.857192	0.723128
Al	7.369244	-1.846261	0.122718
O	7.906793	-3.142377	1.374222
O	6.542786	-0.692017	-1.237242
O	3.952051	0.345734	-1.984309
Si	5.146647	-2.072954	2.318011
O	6.263080	-1.206294	3.177463
Si	7.285322	-0.026903	2.719769
O	6.527622	1.439796	2.718269
Si	6.963973	2.946411	2.198744
O	5.631503	3.883341	2.439197
Si	4.243561	3.854091	1.568969
O	4.643448	3.495083	0.008365
Al	6.359044	3.820648	-0.801589
O	8.157733	3.804034	-1.567677
Al	9.063924	2.886074	-0.098232
O	3.648203	-1.514530	2.662850
O	5.056985	-3.619528	2.803445
Si	5.603874	-5.150752	2.463452
O	7.484651	-6.127603	-0.952570
O	4.272850	-6.102653	2.675118
O	7.842238	-0.343989	1.207936
Al	9.642093	0.038196	0.611830

O	9.028071	1.143059	-0.874526
O	9.212203	-1.525385	-0.460803
O	7.216562	-3.391424	-0.924373
O	5.873739	2.254340	-1.752577
O	2.347341	1.996225	-0.359041
Si	1.953349	1.932886	1.292384
O	7.274244	2.880402	0.574292
O	6.628299	5.520068	-0.004671
Al	5.828116	6.663597	-1.332249
O	5.630960	4.944610	-2.129204
O	9.801873	1.808126	1.236874
O	-3.986709	-3.487200	-0.287773
O	-6.250767	-3.924892	-1.551112
Si	-7.268949	-3.474890	-2.770989
O	-6.367923	-3.416455	-4.135400
O	-7.932310	-2.032315	-2.371734
O	-4.722210	-1.362798	-3.055233
O	3.501349	5.301119	1.669192
Si	3.801475	6.815123	1.062043
O	3.203356	2.697424	2.093380
O	2.379686	7.595090	1.329538
Si	1.052354	7.772703	0.369516
O	4.072991	6.699013	-0.567830
Al	3.155891	7.728559	-1.910091
O	-0.220075	6.878565	0.890698
O	1.436301	7.286646	-1.160399
O	-1.012433	4.418976	0.818141
O	2.010747	0.295495	1.668777
O	1.524687	4.565014	-0.815536
O	0.765875	4.142670	-3.144934
O	2.609924	6.180061	-2.898699
O	4.893726	7.643114	-2.648971
H	-1.508911	4.890979	-4.592953
H	1.376922	1.450463	-3.822575
H	4.560246	-2.367657	-2.768850
H	8.145692	-6.826519	-0.967012
H	3.130390	5.636430	-3.489435
H	6.344398	1.417448	-1.628826
H	9.312522	-1.423699	-1.413465
H	-2.877157	-0.093504	-5.003549
H	0.165896	-3.247616	-4.371218
H	3.154700	-7.070175	-3.371565
H	5.153537	7.820734	-3.553137
H	8.097555	3.321540	-2.401460
H	-1.151217	1.802214	-4.672957

H	2.114602	-1.023751	-3.086422
H	5.355026	-5.386516	-2.454127
H	4.717660	4.654207	-2.283805
H	8.172738	0.778670	-1.145513
H	-4.579366	3.080944	-4.982526
H	-1.487908	-0.905950	-3.790020
H	1.602262	-4.534385	-2.764722
H	0.170876	6.737370	-4.415118
H	3.514242	2.782604	-3.243383
H	6.662500	-1.069830	-2.116656
H	-2.497607	-2.866785	-5.512754
H	0.871172	-6.007924	-4.061679
H	-0.136949	3.817460	-3.302408
H	2.993448	0.214385	-2.051817
H	6.307071	-3.581314	-1.209988
H	-6.098601	-1.675448	-5.637663
H	-2.784806	-5.552126	-4.781501
H	6.607803	5.781847	0.924644
H	10.146168	2.081376	2.090233
H	-2.804490	3.502116	-1.373932
H	0.469827	-0.260414	-0.396362
H	3.880909	-4.065447	0.413712
H	2.402551	4.798870	-0.479943
H	5.364381	0.939889	0.959507
H	8.521646	-2.998248	2.100967
H	-3.980133	-1.243016	-2.446508
H	-0.784659	-4.973229	-1.460270
H	-2.266163	2.458322	1.147949
H	-1.994000	-2.870402	1.431056
H	3.416922	-0.621885	2.359427
H	-0.095244	4.122609	0.698627
H	-3.107213	-3.780664	-0.008605
H	2.476883	-1.257373	0.599579
Si	0.271042	2.653742	3.482570
O	0.403639	2.395464	1.838175
O	0.478744	1.315337	4.432289
O	-1.302837	3.206670	3.670861
O	1.308106	3.793699	4.077039
H	-1.929974	2.581724	3.278822
H	1.302240	4.622843	3.587145
Si	-2.748167	-0.182101	2.270111
O	-2.650166	-0.434441	0.624210
O	-1.239884	0.054108	2.886973
O	-3.280012	-1.557893	3.052247
O	-3.686203	1.131369	2.675299

H	-4.241318	-1.637267	3.188565
H	-3.717736	1.353037	3.653651
Si	0.395181	-2.774652	3.493623
O	0.858640	-2.308937	1.945609
O	0.472428	-1.379772	4.409622
O	1.455836	-3.906450	4.068584
O	-1.090893	-3.458079	3.692480
H	2.337342	-3.829285	3.690719
H	-1.866358	-2.872419	3.656878
Si	-0.498958	-0.043000	4.387998
C	-1.741180	-0.104456	5.779747
H	-2.665611	-0.551309	5.360216
H	-1.347178	-0.880076	6.458797
C	-2.060963	1.177701	6.567067
H	-1.900235	0.986674	7.640296
H	-1.354427	1.990853	6.312860
C	-3.479853	1.701894	6.421199
H	-4.214986	0.875673	6.516695
H	-3.689262	2.410446	7.250752
N	-3.708805	2.369228	5.133930
C	-4.926333	3.177004	5.167504
H	-2.912365	2.999914	4.978466
C	-5.187685	3.942580	3.885191
H	-5.783897	2.508275	5.380150
H	-4.887519	3.911415	6.000139
H	-5.973833	4.687335	4.084996
H	-4.275752	4.500680	3.592419
N	-5.644357	3.100962	2.758871
H	-5.970739	3.697743	1.997320
H	-4.854600	2.549839	2.391571
Au	-7.045148	1.463146	3.218795
Au	-8.668662	-0.554019	3.712956
Au	-6.040393	-0.741905	4.751870

Au_4/fHNT

307

O	10.344022	-2.560755	-0.391208
O	9.082215	-5.034004	-1.400898
O	8.854757	1.376138	4.048630
O	9.165042	-4.091571	3.365424
O	9.560187	-1.579495	1.808473
O	-4.988174	-5.129636	-6.240553

O	7.366887	-7.948439	1.768201
O	-7.111877	-1.305629	-4.609249
O	-5.940756	-7.440900	-2.335287
O	-7.951810	-3.635253	-2.962232
O	7.734477	6.629841	1.702529
O	-1.151960	9.320033	-2.774405
O	6.509564	5.339679	3.731343
O	10.020253	2.288394	0.141326
O	9.337352	3.876515	2.137585
O	1.702641	8.233458	1.755692
O	-2.725710	7.246447	-5.970934
O	0.867721	9.947137	-4.147052
O	3.843082	10.236817	-2.158372
O	3.535360	9.419506	0.109489
O	7.272811	7.527336	-0.657642
O	3.183892	-10.526890	0.080685
O	-1.346077	-10.342590	-1.301056
O	4.894980	-7.617730	-3.418489
O	-1.308912	8.481512	-0.148910
O	-5.382515	6.023600	-2.137427
O	-7.958022	1.642205	-3.229454
O	-6.809618	3.841478	-5.053553
O	-5.890006	2.255882	-6.816437
O	-0.593334	-7.572842	-5.126686
O	-4.976952	6.889379	-4.648820
O	-6.307747	-2.614770	-6.623976
H	-1.853999	7.426692	-5.552835
H	0.838579	9.717873	-5.083476
H	4.648580	-7.837150	-4.326633
H	9.035351	-5.238503	-2.343039
H	4.756357	10.079086	-2.424001
H	-4.319862	-5.454323	-6.855883
H	-5.665605	1.313787	-6.593745
H	9.763739	1.341406	0.022256
H	7.416682	7.945996	0.209945
H	0.081790	-7.684734	-5.807426
H	-6.057897	-3.557961	-6.566954
H	10.156276	-3.488867	-0.624474
H	-6.974527	4.717831	-4.699965
H	9.370818	3.456157	3.004852
H	-7.402489	-0.425544	-4.348905
H	9.477704	-0.812715	2.378030
H	6.914665	-8.408470	2.480409
H	8.793221	1.193463	4.990785
H	6.919655	6.021662	3.143235

H	0.826181	8.415809	2.112390
H	9.126357	-4.902514	3.881356
H	-1.413592	8.989258	-0.992143
H	-5.434000	6.488716	-3.014153
H	4.074642	-10.423794	0.424428
H	-1.031147	-11.056361	-0.727494
H	-5.500172	-8.184803	-1.914708
H	-8.195461	-2.829267	-3.450462
H	-8.217665	2.341275	-3.850421
H	-5.651782	6.681350	-5.302712
H	8.518894	6.158193	2.017623
H	2.942976	9.330189	0.875251
H	-1.911841	8.843058	-3.131630
H	-0.058597	10.214207	-3.920612
H	3.896789	10.438848	-1.181667
H	6.357370	7.752553	-0.941716
H	10.562748	2.338692	0.942696
H	10.766684	-2.566741	0.489869
H	8.828293	-5.863002	-0.926631
H	4.708219	-8.445305	-2.914353
H	-0.703248	-8.446357	-4.688875
H	-5.046051	-5.798263	-5.506679
H	-7.205421	-2.527088	-6.256811
H	-6.831842	2.377115	-6.620530
H	-3.334702	7.922172	-5.626936
H	2.443093	-3.661030	-0.148969
H	0.237016	1.295079	1.742308
H	-1.959762	-1.188820	-1.627726
Al	8.574334	-1.818668	0.335910
O	7.727389	-2.604133	-1.238935
Al	7.160336	-4.283136	-0.621579
O	6.943388	-1.022481	0.968077
Al	7.011827	0.667981	0.080515
O	7.842915	1.519329	1.593005
Si	7.448200	1.386918	3.188768
O	8.101754	-3.592411	0.864520
Si	7.812906	-3.916949	2.466923
O	8.718319	-0.111387	-0.498954
Si	6.321645	-1.285402	2.481501
O	4.703396	-1.376034	2.385184
Si	3.333626	-1.167627	1.472763
O	2.609549	-2.659846	1.529611
O	6.876597	-2.704435	3.060516
O	6.699083	-0.041364	3.469278
O	7.020939	-5.355532	2.550091

Si	6.675854	-6.495849	1.401696
O	5.054729	-6.710542	1.463567
Si	3.669218	-6.504634	0.636124
O	4.013911	-6.093630	-0.955829
Al	3.086145	-6.828122	-2.436768
O	2.100395	-7.359692	-4.017238
Al	0.357862	-6.817915	-3.358012
O	0.441958	-5.153595	-4.226639
Al	-1.125724	-4.280205	-3.569207
O	-2.843361	-3.723139	-3.022698
Al	-3.858885	-4.193231	-4.505954
O	7.231731	-6.011713	-0.039850
O	6.217456	-4.806204	-2.205182
Al	4.438207	-4.343074	-1.593490
O	2.825860	-3.558878	-1.034347
Al	2.952234	-1.801699	-1.707532
O	1.214133	-1.272547	-1.204874
Al	0.234620	-1.781454	-2.717208
O	-1.327712	-0.858378	-2.283608
Al	-1.214002	0.733086	-3.226959
O	0.494550	-0.079502	-3.622658
O	5.424481	-3.856491	-0.025115
O	4.617724	-2.589138	-2.302377
O	3.566052	-5.119275	-3.163443
O	1.957860	-2.379543	-3.258481
Al	4.303663	0.721127	-0.954293
O	3.752873	-0.981531	-0.146818
O	3.529286	-0.172282	-2.456200
O	-0.609514	-2.590976	-4.271899
O	-0.336385	-3.486190	-1.949587
Si	-1.031387	-3.632717	-0.433170
O	-2.167011	-4.788402	-5.108179
O	-1.270903	-6.029967	-2.788098
Si	-2.000071	-6.402680	-1.335073
O	-1.366545	-7.777581	-0.737748
Si	-0.421191	-8.990330	-1.363567
O	-0.036267	-8.526229	-2.875353
O	-5.478781	-3.348698	-4.070491
Al	-5.398364	-1.715945	-5.033402
O	-4.424864	-5.843255	-3.947071
Si	-4.923195	-6.147911	-2.429817
O	-3.797585	-2.654754	-5.568142
Si	-6.344907	-3.502753	-2.657298
O	-5.760921	-4.820555	-1.881178
O	-6.009466	-2.146067	-1.757931

Si	-5.209686	-0.757520	-2.059647
O	-4.559777	-0.784834	-3.580406
Al	-3.836194	0.716770	-4.487564
O	-4.793774	-0.164062	-5.970040
O	-3.626797	-6.485773	-1.457706
O	-1.594356	-5.197388	-0.295820
O	-2.340092	-2.639185	-0.494518
O	5.269400	1.206582	0.612145
O	6.033025	0.055169	-1.423313
O	7.462351	2.338391	-0.643977
Al	8.338158	3.226987	0.804085
O	6.666550	4.058525	1.314837
Al	6.939368	5.702747	0.349644
O	2.745603	1.621236	-0.475995
Al	2.838863	3.243193	-1.372567
O	1.072683	3.858180	-1.043866
Al	0.189423	3.268207	-2.625797
O	-0.669697	2.370785	-4.086784
O	4.514397	2.424985	-1.852041
O	3.547445	4.244015	0.106345
Al	4.218680	5.822249	-0.752221
O	5.179411	6.375146	0.766485
O	3.340152	4.939825	-2.273507
O	1.898971	2.514772	-2.897239
Si	3.159594	4.214958	1.718735
O	2.419919	5.662807	2.023329
Si	1.760171	6.759447	1.019565
O	0.210983	6.342710	0.630129
Si	-0.891123	6.936454	-0.448308
O	-2.186684	5.926675	-0.334215
Si	-2.284106	4.390961	-0.896938
O	-1.381428	4.309395	-2.276374
Al	-0.964676	5.800035	-3.420749
O	-0.225290	7.309983	-4.418656
Al	0.426706	8.343521	-2.892294
O	2.133151	2.996187	2.088976
O	4.404034	3.932833	2.722418
Si	6.055600	4.052149	2.854398
O	8.483482	4.786405	-0.264421
O	6.495385	2.648894	3.602738
O	2.653339	6.847244	-0.355531
Al	2.977986	8.482253	-1.336470
O	2.240817	7.759524	-2.992144
O	4.607508	7.488610	-1.707227
O	5.900260	5.101186	-1.151695

O	0.615864	4.783489	-3.669209
O	-0.535844	1.736655	-1.710990
Si	-1.075519	1.755372	-0.101986
O	-0.261995	6.814357	-1.974340
O	-2.680670	6.601185	-3.325201
Al	-3.551784	5.749450	-4.817226
O	-1.797994	5.012553	-4.917871
O	1.218459	9.151651	-1.405988
O	2.706985	-5.368628	1.320877
O	2.858053	-7.915088	0.683467
Si	2.528638	-9.088912	-0.430495
O	3.129686	-8.561704	-1.857959
O	0.908324	-9.322021	-0.457562
O	1.385491	-6.314396	-1.852767
O	-3.841280	4.010044	-1.188252
Si	-4.961666	4.454626	-2.327547
O	-1.669891	3.298555	0.163359
O	-6.146456	3.334754	-2.118826
Si	-6.389511	1.876654	-2.846749
O	-4.295831	4.284203	-3.834636
Al	-5.090668	3.294739	-5.280976
O	-6.078408	0.617398	-1.842913
O	-5.384245	1.761517	-4.150789
O	-4.027422	-0.655678	-0.918046
O	0.322645	1.571033	0.809610
O	-2.974880	1.371959	-2.894922
O	-2.105388	-0.010135	-4.769509
O	-3.531946	2.215098	-5.560341
O	-4.313248	4.739678	-6.219654
H	-2.986361	-2.369047	-5.990497
H	0.725419	-0.152007	-4.553152
H	4.780173	2.339374	-2.771564
H	9.308988	5.265270	-0.142415
H	-2.715745	2.449998	-6.000816
H	1.473852	5.087999	-3.340028
H	4.825607	7.368519	-2.637803
H	1.313998	-4.789399	-4.398468
H	4.848468	-2.398902	-3.214625
H	8.869669	-0.105998	-1.449199
H	-4.134969	4.798202	-7.158462
H	0.447099	6.947455	-5.008638
H	-0.040745	-2.684191	-5.042406
H	2.971455	0.220918	-3.130416
H	7.665094	2.550200	-1.556252
H	-1.738606	4.049888	-4.807805

H	2.418450	6.807745	-2.973086
H	-2.063393	-5.744332	-5.208642
H	2.078973	-3.340205	-3.224855
H	5.943091	-0.905123	-1.520464
H	-4.263325	-0.355495	-6.750401
H	-0.074629	2.288603	-4.838644
H	3.980077	4.757819	-2.971941
H	4.104952	-5.125393	-3.960153
H	7.473915	-2.221034	-2.079780
H	-2.010448	-0.970728	-4.651635
H	1.774582	1.563443	-2.755052
H	5.907801	4.130319	-1.198235
H	2.059561	-8.321394	-3.896963
H	6.262587	-5.767418	-2.298622
H	-3.202001	6.865670	-2.556540
H	0.809895	9.745079	-0.771690
H	-3.031915	-3.083603	-2.322131
H	1.019774	-0.513509	-0.645267
H	5.197868	2.135873	0.869424
H	-3.046197	2.334284	-2.812450
H	0.870027	4.703091	-0.624293
H	5.001178	7.166894	1.284495
H	1.299726	-5.436404	-1.456090
H	5.297536	-2.932185	0.230493
H	-2.679513	-2.163695	0.279557
H	2.181885	-2.929487	2.360470
H	1.343713	2.885276	1.535036
H	-3.467908	0.122332	-1.076144
H	3.134865	-4.526981	1.535596
H	2.188587	1.428899	0.290162
Si	-2.800658	0.723294	1.939112
O	-2.060486	0.485006	0.473842
O	-1.819148	1.010374	3.231713
O	-3.647797	-0.696964	2.125537
O	-3.808264	2.082026	1.928659
H	-4.458381	-0.675571	2.671832
H	-4.482018	2.069325	1.235626
Si	-0.768890	-2.890721	2.338189
O	0.001123	-3.229272	0.885708
O	-0.631781	-1.288876	2.712030
O	0.130373	-3.603749	3.561475
O	-2.339804	-3.384314	2.457023
H	0.421587	-4.508646	3.405872
H	-2.973746	-2.642340	2.445195
Si	2.153850	-0.124248	3.826949

```
 O     2.310547     0.018350     2.161137
 O     0.675112     0.572526     4.161702
 O     3.377578     0.697179     4.580493
 O     2.203641    -1.630465     4.501507
 H     3.669979     1.472603     4.091033
 H     1.462548    -2.236501     4.327422
Si    -0.798553    -0.140183     3.910508
 C    -1.474719    -0.772616     5.515114
 H    -0.670405    -1.341963     6.019560
 H    -1.636118     0.146142     6.112850
 C    -2.757149    -1.584705     5.389817
 H    -3.514815    -0.997725     4.835487
 H    -2.574669    -2.495779     4.792296
 C    -3.373672    -2.005268     6.710446
 H    -2.706739    -2.730433     7.232557
 H    -4.319195    -2.538635     6.499258
 N    -3.696127    -0.864573     7.556838
 C    -4.440378    -1.211240     8.744959
 H    -2.836458    -0.382085     7.825922
 C    -4.842448     0.018653     9.534976
 H    -5.352851    -1.756423     8.429631
 H    -3.894478    -1.902166     9.426632
 H    -5.212692    -0.287176    10.530304
 H    -3.959993     0.661010     9.701195
 N    -5.854539     0.825919     8.827469
 H    -6.735975     0.306746     8.789443
 H    -6.059132     1.676051     9.355510
Au    -6.471699    -2.743930     3.561640
Au    -4.771077     2.089372     4.232072
Au    -6.062656    -0.335121     4.507893
Au    -5.393476     1.350959     6.676861
```

Au_5/fHNT

```
  308
 O     4.982400    -2.738207     7.754135
 O     5.041383     0.197945     8.076786
 O     5.122535    -6.406160     2.859676
 O     7.809792    -1.956664     4.714275
 O     5.561726    -3.748820     5.504922
 O    -5.019770     8.921242     1.437167
 O     7.968491     2.540839     5.352381
 O    -7.037609     6.468309    -1.984205
```

O	-1.552941	10.173997	-1.375696
O	-5.043921	8.317541	-3.188706
O	0.097418	-9.405643	2.525083
O	-8.734387	-5.715482	-1.299934
O	1.593822	-8.305131	0.567872
O	2.525635	-6.638425	6.124607
O	2.682140	-8.133747	3.953653
O	-3.669681	-7.597372	-2.111355
O	-10.535182	-2.321197	0.193473
O	-9.034778	-6.877267	0.917394
O	-6.397850	-9.213076	1.611737
O	-4.553856	-9.036446	0.038725
O	-2.226283	-9.223091	3.600316
O	6.208154	7.264355	4.094118
O	2.970983	9.849113	1.791185
O	3.057120	5.021438	7.039548
O	-6.562155	-5.701003	-3.003499
O	-8.540631	-1.046120	-4.007209
O	-8.126585	4.133814	-4.146100
O	-10.019820	2.280555	-2.576356
O	-9.904084	3.585322	-0.396513
O	-0.790199	8.306792	4.310789
O	-10.529675	-1.243481	-2.211124
O	-7.301129	7.680849	0.224847
H	-9.927036	-3.035945	0.487525
H	-9.560704	-6.411680	1.578405
H	2.439782	5.583393	7.526054
H	4.489386	0.654308	8.723834
H	-6.049593	-9.482596	2.469398
H	-4.936687	9.012323	2.393977
H	-9.126424	4.167143	-0.186205
H	2.840216	-5.707163	6.227398
H	-1.795638	-9.881467	3.026062
H	-0.865185	8.241014	5.270877
H	-6.623351	8.297343	0.565206
H	5.242167	-1.825395	7.978506
H	-10.340124	1.557789	-3.119704
H	3.519748	-8.057270	3.481907
H	-7.484373	5.833932	-2.554088
H	5.488591	-4.486895	4.897436
H	8.486939	2.944410	4.651159
H	5.834594	-6.493762	2.219281
H	1.017597	-8.901366	1.107900
H	-3.951605	-7.392586	-3.009830
H	8.587638	-1.427849	4.512751

H	-7.465882	-5.818057	-2.617002
H	-9.417989	-1.146752	-3.551408
H	6.817255	6.623138	4.468379
H	3.906070	10.100283	1.779388
H	-0.644602	10.427886	-1.189051
H	-5.936992	7.930850	-3.204049
H	-9.059094	3.879360	-4.060123
H	-11.187727	-0.541269	-2.201056
H	0.951060	-9.519527	2.967106
H	-4.270399	-8.875011	-0.877178
H	-9.083971	-4.836126	-1.492454
H	-9.476082	-6.678219	0.053513
H	-5.817647	-9.680785	0.946705
H	-2.986982	-8.851201	3.097828
H	3.306603	-7.186606	5.955452
H	5.790477	-3.201560	7.458688
H	5.696479	0.864435	7.755404
H	3.764322	5.643587	6.745089
H	-0.065701	8.945111	4.125198
H	-4.180946	9.283273	1.044367
H	-7.534218	7.970113	-0.675332
H	-10.293622	3.918841	-1.219407
H	-10.967039	-2.649599	-0.613653
H	1.922976	2.166809	2.251881
H	-0.579339	-1.231594	-1.646308
H	-2.574886	2.865369	-0.387227
Al	4.212559	-2.628674	5.855035
O	3.161010	-1.110108	6.489568
Al	4.228531	0.364288	6.033171
O	3.415581	-2.607672	4.106332
Al	1.916172	-3.756943	4.390912
O	2.881400	-5.302858	3.773157
Si	3.849507	-5.443995	2.445516
O	5.317442	-1.101444	5.545583
Si	6.446857	-1.144032	4.329422
O	2.776402	-3.845535	6.153713
Si	4.289325	-2.502566	2.702199
O	3.489259	-1.565680	1.644705
Si	2.089357	-0.769650	1.252891
O	2.597060	0.796471	1.020859
O	5.731627	-1.807497	3.007505
O	4.461763	-3.982536	2.034261
O	6.909616	0.403824	4.023403
Si	6.586488	1.828700	4.800293
O	5.960962	2.822736	3.660737

Si	4.613075	3.607224	3.197630
O	3.470357	3.548130	4.427570
Al	2.415601	5.040700	4.930700
O	1.153553	6.427543	5.416559
Al	0.460629	6.704081	3.625494
O	-1.003959	5.562443	3.913940
Al	-1.795454	5.481232	2.176245
O	-2.561473	5.764476	0.475572
Al	-3.805502	7.089516	0.861564
O	5.608367	1.558730	6.061977
O	2.980455	1.722988	6.549554
Al	2.280544	2.095497	4.781575
O	1.449449	2.137273	3.097895
Al	0.087451	0.843408	3.264235
O	-0.703021	1.172201	1.584747
Al	-1.928036	2.518183	2.024137
O	-2.896698	2.457533	0.430675
Al	-4.357808	1.380609	0.806126
O	-3.353202	1.265709	2.453410
O	3.559317	0.748146	4.313930
O	0.921769	0.786875	5.010243
O	1.215030	3.618232	5.393016
O	-1.133265	2.263719	3.734011
Al	-0.129930	-2.106488	3.167364
O	1.087849	-0.675185	2.599145
O	-1.037413	-0.558912	3.827016
O	-2.951332	4.049035	2.652193
O	-0.746070	3.972860	1.468929
Si	0.030289	4.022210	-0.012356
O	-3.068987	6.866148	2.589869
O	-0.373130	6.748259	1.922160
Si	0.467551	7.014596	0.506428
O	1.936294	7.628297	0.845454
Si	2.634086	8.296406	2.195577
O	1.536304	8.150906	3.388371
O	-4.757606	7.121510	-0.756880
Al	-6.270854	6.033985	-0.400593
O	-2.793195	8.556515	0.439195
Si	-1.834187	8.630318	-0.871557
O	-5.343849	6.116159	1.291656
Si	-4.130368	7.293310	-2.289232
O	-2.595317	7.835860	-2.118221
O	-4.100300	5.769230	-2.951227
Si	-4.680623	4.319673	-2.481379
O	-5.386296	4.436631	-0.990008

Al	-6.477902	3.106712	-0.189368
O	-7.467803	4.733336	0.325194
O	-0.358519	7.957478	-0.540667
O	0.710270	5.536757	-0.167695
O	-1.191151	3.912519	-1.109417
O	1.138147	-3.441744	2.686768
O	0.754291	-2.329780	4.847360
O	0.723948	-5.133797	4.839277
Al	1.646529	-6.714652	4.288580
O	0.727765	-6.666761	2.585592
Al	-0.700097	-7.862376	3.076719
O	-1.053454	-2.163472	1.551680
Al	-2.509374	-3.267709	1.874563
O	-3.479028	-2.944430	0.274276
Al	-4.660351	-1.562972	0.845979
O	-5.578789	0.020039	1.419071
O	-1.574728	-3.337264	3.556339
O	-1.708436	-4.861501	1.160934
Al	-2.834404	-6.240026	1.875358
O	-1.638253	-7.613656	1.408985
O	-3.811545	-4.641995	2.472258
O	-3.603117	-1.761390	2.397363
Si	-0.782431	-5.094393	-0.195292
O	-1.728403	-5.967712	-1.233863
Si	-3.333759	-6.227956	-1.257098
O	-4.120811	-4.980039	-1.997751
Si	-5.715244	-4.584215	-2.174690
O	-5.710894	-3.131099	-2.949023
Si	-5.298086	-1.680866	-2.307953
O	-5.755250	-1.690820	-0.722010
Al	-7.151058	-2.786026	0.024668
O	-8.301388	-4.105137	0.895947
Al	-7.513530	-5.709539	0.103322
O	-0.358496	-3.684191	-0.907821
O	0.658959	-5.794315	0.065632
Si	1.483613	-6.781980	1.116250
O	0.133090	-7.746576	4.777726
O	2.976800	-6.087990	1.222615
O	-3.885566	-6.371015	0.282831
Al	-5.293977	-7.581520	0.823149
O	-6.381825	-6.147486	1.576233
O	-4.211262	-7.516383	2.436721
O	-1.895685	-6.413835	3.486519
O	-5.996231	-2.711718	1.526027
O	-3.547891	-0.210195	0.045445

Si	-2.722434	-0.399850	-1.428075
O	-6.382537	-4.378456	-0.674073
O	-8.357387	-2.572989	-1.422740
Al	-9.327869	-1.011873	-0.846960
O	-8.141972	-1.294897	0.616759
O	-6.561461	-7.192785	-0.515220
O	3.989365	2.993124	1.812297
O	5.026688	5.152437	2.896219
Si	4.752993	6.586514	3.668103
O	3.782702	6.254409	4.942905
O	4.077417	7.620205	2.593136
O	1.707139	5.339546	3.226018
O	-6.041867	-0.485555	-3.128188
Si	-7.604697	0.057449	-3.244896
O	-3.678611	-1.416506	-2.355921
O	-7.421779	1.515397	-3.981316
Si	-7.234659	3.025047	-3.348116
O	-8.214771	0.277890	-1.720789
Al	-9.041549	1.897867	-1.092449
O	-5.708911	3.596870	-3.536004
O	-7.572337	2.967813	-1.733495
O	-3.386257	3.302675	-2.439921
O	-1.324523	-1.234499	-1.015338
O	-5.335740	1.680904	-0.797799
O	-5.432097	2.878872	1.378406
O	-7.883693	2.043179	0.427920
O	-10.097424	0.594821	-0.220372
H	-5.395238	5.585648	2.087910
H	-3.835150	1.468558	3.260237
H	-2.025106	-3.144973	4.383180
H	0.353253	-8.594740	5.174982
H	-7.917124	1.556260	1.250829
H	-5.523513	-3.494545	1.843737
H	-4.673342	-7.268465	3.244797
H	-0.898374	4.866099	4.567067
H	0.307912	0.772418	5.748208
H	2.199691	-3.658881	6.901018
H	-10.682472	0.721387	0.526800
H	-8.178289	-3.992788	1.846652
H	-3.149247	4.048599	3.593838
H	-1.982734	-0.397041	3.804233
H	0.084667	-5.151196	5.552869
H	-7.509678	-0.579242	0.791097
H	-5.760179	-5.475959	1.893116
H	-2.562326	7.613165	2.936123

H	-0.524207	2.967500	4.003101
H	1.171816	-1.480118	5.055749
H	-7.637057	4.834823	1.267556
H	-5.757306	-0.008205	2.364312
H	-3.877143	-4.627936	3.434517
H	0.936980	3.570300	6.312581
H	2.255253	-1.050228	6.796227
H	-4.778394	3.572268	1.572118
H	-3.044215	-0.968965	2.417898
H	-1.390652	-5.620366	3.731787
H	1.743711	7.191352	5.513074
H	3.466465	2.502523	6.850942
H	-8.231797	-2.734883	-2.366453
H	-6.653334	-7.640606	-1.359283
H	-2.526920	5.146890	-0.267898
H	-0.832880	0.501036	0.906702
H	0.768443	-4.228262	2.262977
H	-5.842523	0.917130	-1.110492
H	-3.755424	-3.640991	-0.333505
H	-1.806590	-8.307796	0.763379
H	1.453634	4.562329	2.709065
H	3.164357	-0.005221	3.853237
H	-1.089622	3.484369	-1.973605
H	3.103559	0.999932	0.215891
H	-1.057353	-3.029486	-1.066982
H	-3.649978	2.429787	-2.105453
H	3.880792	2.031244	1.788456
H	-0.696028	-1.937230	0.682593
Si	-1.604746	0.720854	-3.815228
O	-2.104407	0.967926	-2.248272
O	-0.355898	-0.324378	-4.073058
O	-1.081715	2.268309	-4.260936
O	-2.822140	0.220026	-4.821493
H	-1.283698	2.463557	-5.183494
H	-3.688661	0.582888	-4.608137
Si	1.637554	2.511001	-1.843806
O	1.208362	2.791070	-0.253992
O	1.036560	1.041248	-2.305804
O	3.305567	2.425781	-1.915593
O	1.183050	3.642230	-2.984809
H	3.570045	2.488920	-2.872225
H	0.429569	3.325191	-3.521633
Si	2.535522	-1.710659	-1.378933
O	1.408489	-1.418879	-0.176186
O	1.705515	-1.521514	-2.802494

O	3.126721	-3.261524	-1.262807
O	3.904004	-0.770376	-1.356890
H	2.498183	-3.894076	-0.896984
H	3.835541	0.139427	-1.690530
Si	1.209847	-0.106883	-3.512382
C	2.461284	0.392171	-4.778765
H	3.383901	0.533413	-4.175035
H	2.694958	-0.489619	-5.402375
C	2.182931	1.636795	-5.627813
H	1.813660	1.330590	-6.620228
H	1.373244	2.251101	-5.191467
C	3.407630	2.518237	-5.812746
H	4.275550	1.890895	-6.099396
H	3.242791	3.236732	-6.643973
N	3.753154	3.221141	-4.575355
C	4.935146	4.060167	-4.723707
H	2.954276	3.806526	-4.302233
C	5.445167	4.589670	-3.397436
H	5.729953	3.445962	-5.188529
H	4.755935	4.918236	-5.406777
H	6.278817	5.280041	-3.600917
H	4.652367	5.182734	-2.900559
N	5.926558	3.525087	-2.493221
H	6.677778	3.878582	-1.900830
H	5.167003	3.244628	-1.860880
Au	6.384041	1.459392	-3.442570
Au	6.740647	0.050945	-5.834516
Au	5.544077	-2.482833	-6.099882
Au	5.672687	-1.160447	-3.558932
Au	4.396280	-3.568977	-3.870950

Au_6/fHNT

309			
O	-11.070672	1.119125	-0.243602
O	-10.848221	-1.703095	0.601489
O	-7.933218	4.547671	-4.222377
O	-10.205226	-0.479208	-4.057001
O	-9.814982	1.898567	-2.300682
O	1.841611	-7.149506	6.143330
O	-10.018243	-4.821096	-2.725474
O	5.302096	-4.239654	4.980531
O	2.233020	-9.383842	2.099198

O	5.398158	-6.600711	3.174575
O	-5.228124	8.884182	-1.352228
O	3.640211	7.911959	3.810740
O	-4.375707	7.378829	-3.423052
O	-9.016373	5.555054	-0.314747
O	-7.657453	6.922339	-2.119574
O	0.964771	8.222421	-0.942869
O	4.116301	5.205132	6.883308
O	1.866572	9.127772	5.126997
O	-0.643295	10.594545	3.019703
O	-0.459837	9.873750	0.706117
O	-4.673550	9.398155	1.100795
O	-7.164689	-8.831843	-1.062735
O	-2.989021	-10.373274	0.565633
O	-8.019446	-5.742189	2.592218
O	3.704750	7.249117	1.131664
O	6.474299	3.369806	3.098976
O	7.242251	-1.705303	3.921104
O	6.798248	0.632995	5.875124
O	5.238537	-0.631721	7.435686
O	-3.026046	-7.778149	4.582787
O	6.196882	4.154593	5.654030
O	3.926981	-5.304937	6.822767
H	3.401189	5.712769	6.438231
H	1.736696	8.841632	6.038894
H	-7.941004	-6.095042	3.488278
H	-10.953312	-1.972892	1.522256
H	-1.571943	10.756874	3.221622
H	1.054022	-7.253574	6.690657
H	4.715418	-1.414076	7.116694
H	-9.120693	4.573322	-0.269102
H	-4.589486	9.897190	0.268581
H	-3.750311	-7.685926	5.214178
H	3.366262	-6.090205	6.667093
H	-11.241747	0.172005	-0.086279
H	7.289650	1.413676	5.611921
H	-7.766201	6.600384	-3.022066
H	5.904413	-3.506679	4.816987
H	-9.421460	2.621034	-2.792955
H	-9.700800	-5.364133	-3.451913
H	-7.863527	4.418061	-5.172723
H	-4.565541	8.121751	-2.797611
H	1.875190	8.102247	-1.235042
H	-10.412975	-1.214103	-4.641878
H	3.912646	7.628572	2.021944

H	6.614851	3.726372	4.015894
H	-7.930796	-8.394184	-1.441890
H	-3.487499	-10.887273	-0.086248
H	1.594323	-9.891089	1.590123
H	5.869633	-5.969647	3.746254
H	7.680145	-1.188356	4.615891
H	6.699456	3.675889	6.320744
H	-6.100002	8.746569	-1.749642
H	0.123296	9.629382	-0.032481
H	4.151439	7.172017	4.162403
H	2.842726	9.060010	4.974688
H	-0.542640	10.866788	2.063981
H	-3.763851	9.261409	1.451795
H	-9.439160	5.849276	-1.135545
H	-11.395088	1.323328	-1.142507
H	-10.865066	-2.534609	0.067687
H	-8.095970	-6.546356	2.025320
H	-3.195804	-8.602003	4.073636
H	1.719658	-7.744247	5.355679
H	4.824662	-5.520183	6.512679
H	6.175272	-0.842895	7.301279
H	4.950221	5.639369	6.634660
H	-4.072858	-2.717437	-0.170445
H	-0.115398	1.234623	-1.484392
H	0.782787	-2.088876	1.756030
Al	-9.100075	1.225179	-0.806356
O	-8.715540	0.085905	0.732668
Al	-8.728396	-1.639869	-0.004399
O	-7.247489	1.424974	-1.277034
Al	-6.787745	2.965018	-0.244072
O	-7.139353	4.155839	-1.714257
Si	-6.688117	3.997192	-3.292550
O	-9.241623	-0.560914	-1.468747
Si	-8.956260	-0.860174	-3.076685
O	-8.700544	2.811426	0.171770
Si	-6.637301	1.058047	-2.773423
O	-5.168423	0.388043	-2.601049
Si	-3.891493	0.031479	-1.603148
O	-3.738509	-1.613914	-1.757315
O	-7.607675	-0.026556	-3.507517
O	-6.470294	2.417408	-3.662264
O	-8.718186	-2.477992	-3.248841
Si	-8.891875	-3.739833	-2.192524
O	-7.451163	-4.515913	-2.188607
Si	-6.154270	-4.874967	-1.274358

O	-6.460370	-4.474491	0.328132
Al	-5.974876	-5.588981	1.782648
O	-5.371908	-6.541833	3.357658
Al	-3.502755	-6.617135	2.842760
O	-3.065280	-5.094579	3.853691
Al	-1.242406	-4.798557	3.361601
O	0.599749	-4.858192	2.958882
Al	1.260058	-5.757986	4.444374
O	-9.356837	-3.185671	-0.744022
O	-8.162652	-2.569666	1.572253
Al	-6.290859	-2.734517	1.099391
O	-4.465783	-2.544082	0.699266
Al	-4.019083	-0.906928	1.522075
O	-2.171259	-1.002799	1.161496
Al	-1.560570	-1.927786	2.670484
O	0.256605	-1.598434	2.405229
Al	0.634709	-0.138097	3.482285
O	-1.276757	-0.309690	3.712242
O	-6.911157	-1.824181	-0.467714
O	-5.897753	-1.083734	1.954622
O	-5.879158	-3.873860	2.635715
O	-3.421981	-1.904006	3.063966
Al	-4.328492	1.976517	0.931830
O	-4.349368	0.247156	0.002690
O	-4.043718	0.767522	2.384689
O	-1.185494	-3.087122	4.187128
O	-1.566577	-3.668965	1.781847
Si	-0.845616	-3.951832	0.298104
O	-0.576045	-5.746762	4.900382
O	-1.660174	-6.428367	2.433127
Si	-0.993450	-6.940012	0.992439
O	-2.020031	-7.954173	0.240034
Si	-3.379755	-8.786912	0.702419
O	-3.698121	-8.317261	2.227910
O	3.103368	-5.522268	4.173135
Al	3.525471	-4.036689	5.275147
O	1.251655	-7.460267	3.768039
Si	1.732389	-7.821640	2.257571
O	1.658672	-4.373649	5.637534
Si	3.971599	-5.881921	2.799145
O	3.025884	-6.848760	1.876730
O	4.210264	-4.438542	2.010682
Si	3.929478	-2.879042	2.395211
O	3.190141	-2.772844	3.870995
Al	2.970947	-1.175515	4.871904

O	3.432533	-2.437078	6.316189
O	0.483846	-7.607776	1.192114
O	-0.862270	-5.603200	0.047396
O	0.718436	-3.500617	0.516991
O	-4.930199	2.878324	-0.632374
O	-6.213648	1.944073	1.246960
O	-6.678003	4.633967	0.604866
Al	-7.063419	5.871209	-0.800201
O	-5.170349	6.081314	-1.144785
Al	-4.923762	7.646287	-0.049546
O	-2.519755	2.288975	0.619810
Al	-2.108030	3.773500	1.654195
O	-0.217938	3.735976	1.475897
Al	0.268778	2.765313	3.041670
O	0.634474	1.524966	4.457912
O	-3.997515	3.579292	1.968682
O	-2.295479	5.057463	0.237433
Al	-2.434905	6.710655	1.199739
O	-3.012060	7.670315	-0.310595
O	-2.050739	5.473326	2.678330
O	-1.612652	2.657421	3.153390
Si	-1.812861	4.998640	-1.348346
O	-0.588178	6.102636	-1.480663
Si	0.331686	6.821256	-0.348026
O	1.597378	5.852776	0.082535
Si	2.746317	5.939718	1.266839
O	3.607533	4.543095	1.129370
Si	3.111276	3.040513	1.554275
O	2.128671	3.196035	2.871326
Al	2.172353	4.657582	4.123228
O	1.933979	6.262313	5.214314
Al	1.814678	7.559645	3.756310
O	-1.255344	3.520692	-1.773682
O	-2.990750	5.247916	-2.437402
Si	-4.477676	5.958929	-2.644377
O	-6.736272	7.304704	0.396743
O	-5.321335	4.859044	-3.539385
O	-0.582008	7.131405	0.980627
Al	-0.388269	8.705302	2.087969
O	-0.090545	7.658384	3.707331
O	-2.287839	8.338445	2.280781
O	-4.289275	6.614402	1.443196
O	0.320332	4.259983	4.194903
O	0.479609	1.139996	2.030517
Si	1.119991	1.064494	0.459658

O	1.992517	5.949849	2.740327
O	4.062289	4.795930	4.191110
Al	4.452742	3.591781	5.643007
O	2.549801	3.526468	5.584061
O	1.482377	8.694396	2.310291
O	-4.801060	-4.115834	-1.801452
O	-5.891301	-6.475835	-1.407320
Si	-6.088605	-7.760990	-0.389117
O	-6.579352	-7.149608	1.046934
O	-4.662381	-8.559837	-0.298473
O	-4.160778	-5.680415	1.337809
O	4.405027	2.109053	1.890936
Si	5.513481	2.046483	3.123141
O	2.239957	2.313294	0.368191
O	6.240329	0.593250	2.875737
Si	5.893748	-0.900136	3.479629
O	4.709970	2.026000	4.571466
Al	4.984487	0.723906	5.961393
O	5.241611	-1.894995	2.350394
O	4.809809	-0.734158	4.713267
O	2.956114	-2.285239	1.207217
O	-0.173903	1.460363	-0.536222
O	2.528548	-0.151255	3.302567
O	1.078194	-1.251786	4.995370
O	3.128053	0.257679	6.059202
O	4.692564	2.285633	6.985666
H	0.968696	-3.845238	6.041032
H	-1.593302	-0.356371	4.618882
H	-4.350330	3.533627	2.861388
H	-7.327165	8.054276	0.275729
H	2.414054	0.739347	6.475833
H	-0.345428	4.872544	3.850284
H	-2.609258	8.242867	3.183840
H	-3.763894	-4.454662	4.012007
H	-6.120093	-0.884533	2.867044
H	-8.917109	2.807523	1.109312
H	4.470552	2.341617	7.915260
H	1.131316	6.126143	5.732775
H	-1.811331	-3.021530	4.915066
H	-3.439969	0.889323	3.120216
H	-6.866637	4.843237	1.520746
H	2.164101	2.658415	5.383849
H	-0.590055	6.836749	3.592415
H	-1.017746	-6.606690	4.907828
H	-3.870743	-2.753177	2.936718

H	-6.476172	1.011028	1.260863
H	2.807001	-2.477335	7.046993
H	-0.010475	1.611485	5.166932
H	-2.768340	5.486472	3.322779
H	-6.448585	-3.739577	3.399206
H	-8.412664	0.296168	1.617026
H	0.660716	-2.104667	4.785718
H	-1.820409	1.736282	2.931902
H	-4.642279	5.709755	1.400695
H	-5.663002	-7.444183	3.152636
H	-8.551118	-3.454983	1.575301
H	4.704115	4.906713	3.478114
H	2.124071	9.142993	1.754949
H	1.057977	-4.283505	2.330301
H	-1.676976	-0.328246	0.684279
H	-4.515032	3.735337	-0.799877
H	2.940925	0.724996	3.311901
H	0.302946	4.478242	1.146192
H	-2.524669	8.378343	-0.744314
H	-3.739074	-4.867020	1.027757
H	-6.446224	-0.991755	-0.631104
H	1.265481	-3.127838	-0.191717
H	-3.367198	-1.962956	-2.585703
H	-0.603556	3.098059	-1.191516
H	2.695830	-1.370847	1.405936
H	-4.885842	-3.164530	-1.960792
H	-2.005872	1.961340	-0.130414
Si	2.554591	-0.340058	-1.560609
O	1.616253	-0.440728	-0.189396
O	1.815787	0.348624	-2.875248
O	2.859155	-1.954798	-1.917396
O	3.931771	0.549681	-1.319894
H	3.618249	-2.051769	-2.525307
H	4.556423	0.186083	-0.679355
Si	-0.604142	-2.985414	-2.406582
O	-1.551015	-3.103633	-1.024376
O	-0.193278	-1.421729	-2.737371
O	-1.620232	-3.379342	-3.684851
O	0.740360	-3.942674	-2.467078
H	-2.310003	-4.019105	-3.476804
H	1.562145	-3.427275	-2.343084
Si	-2.225658	0.741545	-3.784338
O	-2.461630	0.814162	-2.124233
O	-0.578019	0.915648	-3.980366
O	-3.044011	1.972276	-4.529550

```
 O    -2.725918    -0.610535    -4.595820
 H    -3.059014     2.785066    -4.014110
 H    -2.253136    -1.441508    -4.426069
Si     0.532575    -0.291918    -3.734830
 C     1.073851    -1.005153    -5.376909
 H     0.346918    -0.654866    -6.133197
 H     2.047226    -0.528016    -5.615539
 C     1.174557    -2.525725    -5.387204
 H     1.868913    -2.869257    -4.596087
 H     0.191533    -2.962234    -5.139821
 C     1.609379    -3.151469    -6.695729
 H     0.921176    -2.851071    -7.514661
 H     1.522529    -4.247553    -6.599510
 N     3.005072    -2.871332    -7.053249
 C     3.522968    -3.754253    -8.095931
 H     3.086851    -1.899560    -7.362555
 C     5.037663    -3.841149    -8.082427
 H     3.112855    -4.763517    -7.905255
 H     3.174651    -3.467518    -9.107701
 H     5.366103    -4.449143    -8.947252
 H     5.474576    -2.834869    -8.212279
 N     5.499077    -4.367958    -6.798771
 H     5.217422    -5.343497    -6.684204
 H     6.516517    -4.346042    -6.732747
Au     6.576623     1.080425    -2.808150
Au     7.848849    -0.713211    -1.233196
Au     5.054568     2.676281    -4.381919
Au     4.584327    -0.000907    -4.603907
Au     6.219905    -1.817290    -3.151231
Au     4.649811    -2.667106    -5.182876
```

Au_7/fHNT

```
  310
 O    10.676387    -3.708663    -0.155976
 O     9.176844    -5.954906    -1.353622
 O     9.460203     0.150472     4.432837
 O     9.145548    -5.272484     3.457427
 O     9.912158    -2.751799     2.061283
 O    -4.557975    -4.085957    -6.763704
 O     6.976325    -8.794909     1.574103
 O    -6.283485    -0.118860    -5.008980
 O    -5.964824    -6.458558    -3.036506
```

O	-7.473604	-2.408939	-3.530991
O	9.089812	5.613784	2.341046
O	0.812145	9.592603	-2.338496
O	7.624275	4.381709	4.242415
O	10.910747	1.110451	0.633890
O	10.328945	2.666831	2.686381
O	3.297754	7.937294	2.236749
O	-0.845052	7.891468	-5.708078
O	2.955266	10.037123	-3.589879
O	5.846108	9.860068	-1.467797
O	5.335397	8.972551	0.736147
O	8.851528	6.679433	0.017975
O	2.599521	-10.754307	-0.425373
O	-1.805028	-9.948340	-1.979129
O	4.810875	-7.902282	-3.682845
O	0.431428	8.647519	0.227210
O	-3.808171	6.809500	-2.061297
O	-6.835069	2.836482	-3.501919
O	-5.346252	4.968658	-5.151228
O	-4.540489	3.374039	-6.961062
O	-0.545229	-7.100912	-5.609368
O	-3.183069	7.745456	-4.501458
O	-5.547178	-1.412580	-7.059284
H	0.021130	7.942474	-5.245151
H	2.943371	9.860901	-4.538091
H	4.583368	-8.043646	-4.611198
H	9.150613	-6.104126	-2.306875
H	6.745495	9.605650	-1.704194
H	-3.904900	-4.458221	-7.368358
H	-4.441352	2.401523	-6.782534
H	10.548616	0.209142	0.451496
H	9.003146	7.032865	0.912839
H	0.143190	-7.259765	-6.267064
H	-5.415070	-2.380893	-7.045042
H	10.390133	-4.593871	-0.448412
H	-5.421626	5.839531	-4.756116
H	10.270616	2.202113	3.529356
H	-6.478641	0.775792	-4.711831
H	9.895148	-2.010551	2.669076
H	6.438965	-9.231775	2.240260
H	9.332571	-0.070844	5.359949
H	8.140543	5.037432	3.710776
H	2.433499	8.207004	2.566884
H	8.985574	-6.097736	3.925058
H	0.428461	9.206337	-0.589809

H	-3.761901	7.321263	-2.911937
H	3.478874	-10.778329	-0.040167
H	-1.605433	-10.723381	-1.434064
H	-5.636966	-7.271056	-2.640593
H	-7.595523	-1.555523	-3.982838
H	-6.979348	3.592602	-4.092834
H	-3.846142	7.654818	-5.193096
H	9.796248	5.034512	2.661092
H	4.700895	8.917546	1.470769
H	0.018435	9.230729	-2.752749
H	2.057899	10.403498	-3.386981
H	5.877189	10.004253	-0.479923
H	7.984174	7.028876	-0.290309
H	11.416698	1.053428	1.458382
H	11.052978	-3.810880	0.739963
H	8.803244	-6.769655	-0.937389
H	4.502628	-8.725338	-3.233982
H	-0.779623	-7.975527	-5.226097
H	-4.730286	-4.778846	-6.071463
H	-6.444304	-1.234648	-6.724885
H	-5.469260	3.599304	-6.797438
H	-1.384379	8.618197	-5.351987
H	2.698027	-3.846622	-0.299874
H	1.014056	1.239481	1.774279
H	-1.301869	-0.783441	-1.816922
Al	8.975792	-2.793821	0.538455
O	8.116611	-3.389119	-1.111344
Al	7.323901	-5.015421	-0.612863
O	7.423772	-1.837460	1.146890
Al	7.736222	-0.125234	0.359055
O	8.590529	0.540465	1.949693
Si	8.107482	0.376529	3.517998
O	8.269636	-4.521070	0.946965
Si	7.868250	-4.888713	2.515461
O	9.362672	-1.076796	-0.192765
Si	6.703860	-2.099080	2.616357
O	5.092844	-1.985911	2.449054
Si	3.801655	-1.565420	1.495121
O	2.903368	-2.959710	1.437664
O	7.056768	-3.602590	3.137214
O	7.180241	-0.961950	3.687048
O	6.906653	-6.222157	2.485306
Si	6.482408	-7.252100	1.261603
O	4.846143	-7.270058	1.244836
Si	3.536121	-6.854801	0.374143

O	4.002745	-6.408801	-1.176791
Al	3.065062	-6.948463	-2.733366
O	2.098905	-7.274857	-4.380231
Al	0.404473	-6.558443	-3.763646
O	0.728422	-4.874978	-4.533411
Al	-0.752819	-3.851124	-3.892856
O	-2.415375	-3.116876	-3.386841
Al	-3.408311	-3.383623	-4.934500
O	7.160073	-6.767027	-0.126532
O	6.401423	-5.338464	-2.260619
Al	4.663421	-4.693111	-1.697369
O	3.131873	-3.747232	-1.161697
Al	3.499591	-1.986819	-1.729540
O	1.815471	-1.275489	-1.269684
Al	0.855030	-1.583639	-2.847010
O	-0.604379	-0.499845	-2.426790
Al	-0.256227	1.111575	-3.273979
O	1.359538	0.117526	-3.644238
O	5.625284	-4.410760	-0.065072
O	5.085144	-2.940564	-2.298846
O	3.780117	-5.276396	-3.342801
O	2.517940	-2.359495	-3.349908
Al	5.106158	0.310637	-0.781265
O	4.317571	-1.350694	-0.093879
O	4.302514	-0.404205	-2.361400
O	-0.005241	-2.204154	-4.477982
O	0.048064	-3.242536	-2.200322
Si	-0.730782	-3.379812	-0.725668
O	-1.773217	-4.149670	-5.499291
O	-1.143388	-5.607408	-3.217683
Si	-1.980286	-5.961351	-1.818973
O	-1.544905	-7.431912	-1.274198
Si	-0.722940	-8.717740	-1.927758
O	-0.213868	-8.228089	-3.394093
O	-4.934247	-2.370748	-4.519136
Al	-4.613251	-0.713341	-5.384878
O	-4.193711	-4.978369	-4.492682
Si	-4.796409	-5.296405	-3.016600
O	-3.112849	-1.812300	-5.905349
Si	-5.878701	-2.489310	-3.153496
O	-5.494244	-3.906206	-2.429200
O	-5.426283	-1.231097	-2.166483
Si	-4.452556	0.062876	-2.356832
O	-3.739131	0.033521	-3.848810
Al	-2.798681	1.479554	-4.639922

O	-3.783381	0.798558	-6.207459
O	-3.597426	-5.838890	-2.012658
O	-1.482503	-4.869074	-0.698017
O	-1.906763	-2.232204	-0.783311
O	6.047572	0.594490	0.848176
O	6.763588	-0.536891	-1.215622
O	8.417431	1.512212	-0.251563
Al	9.323605	2.212638	1.278725
O	7.741258	3.215133	1.766347
Al	8.254534	4.860527	0.906857
O	3.646127	1.368891	-0.317540
Al	3.975417	3.010664	-1.117185
O	2.281999	3.819289	-0.827189
Al	1.410518	3.422572	-2.474608
O	0.520480	2.712009	-4.017575
O	5.561770	2.019317	-1.572692
O	4.727734	3.841190	0.443319
Al	5.623142	5.367112	-0.297168
O	6.569974	5.720833	1.288251
O	4.718487	4.676848	-1.900269
O	3.028512	2.480719	-2.717541
Si	4.263081	3.778019	2.034289
O	3.688497	5.288049	2.389008
Si	3.213232	6.506519	1.422111
O	1.645536	6.302383	0.946748
Si	0.674920	7.080181	-0.140970
O	-0.736189	6.231713	-0.136875
Si	-0.989983	4.749911	-0.788339
O	-0.039107	4.628746	-2.132009
Al	0.607014	6.113409	-3.172961
O	1.568442	7.570383	-4.053493
Al	2.266310	8.437841	-2.446142
O	3.081658	2.676541	2.293226
O	5.415712	3.295509	3.070739
Si	7.061540	3.205382	3.276790
O	9.705198	3.794779	0.306191
O	7.294082	1.722803	3.962670
O	4.174760	6.554170	0.091870
Al	4.739078	8.184900	-0.781719
O	4.000108	7.642479	-2.504134
O	6.253629	7.019765	-1.140498
O	7.223325	4.467261	-0.667178
O	2.064443	4.925351	-3.413037
O	0.464528	1.946661	-1.677585
Si	-0.145375	1.947270	-0.094373

O	1.356637	6.959682	-1.644373
O	-1.003276	7.112252	-3.103067
Al	-1.898294	6.450019	-4.674953
O	-0.242587	5.510507	-4.744784
O	3.077676	9.066833	-0.885660
O	2.685439	-5.645966	1.081495
O	2.560557	-8.156203	0.309039
Si	2.146202	-9.223018	-0.881551
O	2.873152	-8.701482	-2.251372
O	0.512760	-9.254820	-0.988041
O	1.412374	-6.261154	-2.191681
O	-2.565979	4.577279	-1.164136
Si	-3.569696	5.212542	-2.321679
O	-0.562061	3.538289	0.233350
O	-4.888636	4.236622	-2.224817
Si	-5.269721	2.857910	-3.042699
O	-2.858264	5.038699	-3.807390
Al	-3.696302	4.228315	-5.338296
O	-5.159674	1.520830	-2.099140
O	-4.224707	2.687118	-4.308840
O	-3.322149	-0.038436	-1.163817
O	1.176316	1.549907	0.862822
O	-1.941695	1.943062	-2.979085
O	-1.155967	0.561946	-4.890996
O	-2.266600	2.981951	-5.613629
O	-2.707754	5.613205	-6.161825
H	-2.254117	-1.606766	-6.277411
H	1.624013	0.064622	-4.567077
H	5.858734	1.948614	-2.483901
H	10.575380	4.162436	0.488590
H	-1.408156	3.137435	-6.006413
H	2.936118	5.105719	-3.032405
H	6.499711	6.921266	-2.066620
H	1.644969	-4.611767	-4.648661
H	5.380059	-2.733936	-3.188764
H	9.558523	-1.041767	-1.134208
H	-2.479379	5.697033	-7.087776
H	2.219825	7.158828	-4.634816
H	0.584199	-2.326949	-5.228538
H	3.828428	0.087849	-3.034807
H	8.687164	1.743646	-1.141448
H	-0.304204	4.543463	-4.686524
H	4.061375	6.676397	-2.531206
H	-1.780174	-5.104767	-5.648861
H	2.521399	-3.328219	-3.365270

H	6.564014	-1.472842	-1.370070
H	-3.243228	0.583581	-6.974925
H	1.136447	2.595987	-4.747836
H	5.364443	4.453693	-2.580990
H	4.351620	-5.307888	-4.115841
H	7.951048	-2.935803	-1.939068
H	-1.182440	-0.407784	-4.823380
H	2.784482	1.545728	-2.634134
H	7.116744	3.506358	-0.767732
H	1.937531	-8.229225	-4.315842
H	6.335504	-6.292012	-2.405878
H	-1.525084	7.399118	-2.342803
H	2.713467	9.672793	-0.236368
H	-2.559031	-2.495444	-2.659836
H	1.687066	-0.527753	-0.676838
H	6.075711	1.511313	1.154006
H	-1.901090	2.901467	-2.845780
H	2.162291	4.660264	-0.369562
H	6.463449	6.501135	1.842098
H	1.413635	-5.400506	-1.750251
H	5.597953	-3.492025	0.236541
H	-2.222835	-1.758412	0.001551
H	2.407413	-3.216823	2.233783
H	2.311815	2.691098	1.702046
H	-2.666618	0.672423	-1.254976
H	3.200352	-4.874850	1.360425
H	3.034343	1.207432	0.413151
Si	-2.092527	1.068857	1.796795
O	-1.290073	0.767944	0.373222
O	-1.132358	1.151263	3.147591
O	-3.073295	-0.286548	1.928222
O	-2.932012	2.503797	1.796897
H	-3.916901	-0.127673	2.388414
H	-3.739723	2.508887	1.259274
Si	-0.514690	-2.817112	2.085638
O	0.280495	-3.172375	0.652602
O	-0.196515	-1.275375	2.575082
O	0.220241	-3.722221	3.294788
O	-2.140653	-3.112265	2.109382
H	0.458041	-4.625941	3.060581
H	-2.670087	-2.291101	2.106744
Si	2.628713	-0.509224	3.852346
O	2.893777	-0.300803	2.207111
O	1.232916	0.351099	4.166569
O	3.904302	0.108051	4.708444

```
 O     2.455935    -2.043145     4.440947
 H     4.314101     0.866630     4.280538
 H     1.655628    -2.539671     4.197579
Si    -0.304341    -0.148886     3.800480
 C    -1.137017    -0.790867     5.338352
 H    -0.350747    -1.260790     5.959610
 H    -1.469403     0.119148     5.877436
 C    -2.291706    -1.755947     5.093874
 H    -3.041112    -1.291160     4.423385
 H    -1.925537    -2.654217     4.569767
 C    -3.001299    -2.224318     6.347683
 H    -2.279454    -2.727994     7.028509
 H    -3.763618    -2.976938     6.074659
 N    -3.701182    -1.142872     7.040613
 C    -4.422374    -1.584948     8.223389
 H    -3.027752    -0.419682     7.306757
 C    -5.338866    -0.498331     8.752192
 H    -5.030362    -2.463202     7.931636
 H    -3.744133    -1.928394     9.031554
 H    -5.753801    -0.814524     9.727967
 H    -4.752245     0.419975     8.935208
 N    -6.372533    -0.180001     7.768785
 H    -7.059797    -0.933603     7.708678
 H    -6.886566     0.662110     8.025946
Au    -5.948004     1.271821     3.120763
Au    -5.304282     0.129511     5.554255
Au    -6.532733     2.246537     0.603883
Au    -6.427516    -0.438757     0.849919
Au    -5.961508    -1.618949     3.450980
Au    -6.086833    -3.106567     1.128209
Au    -3.871205     2.314506     4.629276
```

Au_8/fHNT

```
  311
 O   -11.652762    -0.427541    -1.186549
 O   -11.089137    -3.121536    -0.112628
 O    -8.703014     3.195976    -5.136019
 O   -10.196866    -2.105010    -4.779897
 O   -10.338585     0.409456    -3.183608
 O     1.689873    -6.151654     6.881835
 O    -9.485503    -6.272042    -3.109650
 O     4.767244    -2.812932     5.796928
```

O	2.797160	-8.544803	3.067754
O	5.389686	-5.237673	4.191160
O	-6.968303	8.073941	-2.387044
O	1.412336	8.830123	3.566266
O	-5.705327	6.594815	-4.257363
O	-10.293058	4.262693	-1.422329
O	-8.989932	5.711031	-3.204943
O	-0.813941	8.419456	-1.406279
O	1.995842	6.428687	6.865793
O	-0.641549	9.832159	4.631164
O	-3.131378	10.752321	2.213397
O	-2.621729	9.926308	-0.014571
O	-6.733869	8.820670	0.057639
O	-6.231769	-9.673400	-0.910211
O	-2.045935	-10.435522	1.178274
O	-7.886773	-6.534254	2.412737
O	1.831073	8.019751	0.960708
O	4.953314	4.753760	3.445599
O	6.395378	-0.076538	4.714872
O	5.419887	2.280388	6.440064
O	3.927012	0.886379	7.955194
O	-2.856313	-7.632818	4.967959
O	4.318555	5.643164	5.900634
O	3.399107	-3.963817	7.592275
H	1.258113	6.788988	6.324392
H	-0.813139	9.586649	5.547997
H	-7.841821	-6.813803	3.336541
H	-11.239648	-3.346492	0.813864
H	-4.088851	10.779130	2.323099
H	0.878420	-6.343714	7.366965
H	3.560574	0.013239	7.653804
H	-10.252118	3.281542	-1.310828
H	-6.647032	9.273795	-0.800160
H	-3.643048	-7.616221	5.526969
H	2.980544	-4.835540	7.450182
H	-11.693367	-1.378086	-0.972384
H	5.810859	3.110568	6.160497
H	-8.962404	5.320311	-4.086165
H	5.265043	-2.006140	5.629391
H	-10.013319	1.152597	-3.694582
H	-9.022078	-6.802773	-3.763053
H	-8.524329	3.019863	-6.064388
H	-6.063683	7.336287	-3.708779
H	0.127846	8.425703	-1.610408
H	-10.234799	-2.898456	-5.322406

H	1.893602	8.481948	1.833789
H	4.950488	5.184486	4.341199
H	-7.015412	-9.385964	-1.385085
H	-2.396838	-11.061069	0.527750
H	2.293656	-9.176796	2.546736
H	5.704023	-4.505987	4.750782
H	6.682134	0.545082	5.402676
H	4.821666	5.291846	6.642051
H	-7.767594	7.776629	-2.845069
H	-1.940719	9.730905	-0.680402
H	1.993357	8.203019	4.015365
H	0.343690	9.909112	4.567544
H	-2.982269	10.976811	1.251615
H	-5.851595	8.850607	0.493701
H	-10.675176	4.435090	-2.295994
H	-11.917075	-0.333299	-2.122622
H	-10.929532	-3.977054	-0.580878
H	-7.786858	-7.374254	1.904473
H	-2.850674	-8.503255	4.510689
H	1.734570	-6.806146	6.134422
H	4.344394	-4.054393	7.376531
H	4.893369	0.816911	7.916980
H	2.774661	6.972083	6.656099
H	-4.195918	-3.105810	-0.229299
H	-0.772186	1.328704	-1.501941
H	0.303582	-1.603776	2.047118
Al	-9.676087	-0.048767	-1.587150
O	-9.272722	-1.015254	0.061367
Al	-8.955060	-2.764239	-0.540194
O	-7.838426	0.409649	-1.913410
Al	-7.716582	2.064166	-0.965916
O	-8.101877	3.090925	-2.547194
Si	-7.483535	2.906904	-4.064949
O	-9.483083	-1.872746	-2.120793
Si	-9.003942	-2.223250	-3.671284
O	-9.615219	1.638443	-0.702755
Si	-7.040169	0.050191	-3.320405
O	-5.510161	-0.368378	-2.973951
Si	-4.294559	-0.457222	-1.847549
O	-3.882058	-2.064733	-1.861895
O	-7.761528	-1.216790	-4.048956
O	-6.995904	1.360943	-4.293205
O	-8.509482	-3.791182	-3.698644
Si	-8.590918	-4.996234	-2.567523
O	-7.056718	-5.534738	-2.382570

Si	-5.813640	-5.627979	-1.337216
O	-6.327918	-5.181310	0.198380
Al	-5.820892	-6.112794	1.769580
O	-5.234116	-6.859002	3.458077
Al	-3.334491	-6.671918	3.110296
O	-3.229818	-5.039481	4.035353
Al	-1.433852	-4.492247	3.677963
O	0.426144	-4.286957	3.438153
Al	1.069879	-4.977453	5.038511
O	-9.269996	-4.432637	-1.210079
O	-8.408543	-3.493465	1.145027
Al	-6.496835	-3.391602	0.846070
O	-4.691564	-2.942357	0.588294
Al	-4.577157	-1.207282	1.318469
O	-2.710119	-1.034164	1.123784
Al	-2.113701	-1.755799	2.745162
O	-0.349944	-1.162478	2.610159
Al	-0.300632	0.403439	3.600649
O	-2.177502	-0.051416	3.680615
O	-7.095008	-2.689527	-0.832792
O	-6.440301	-1.649459	1.602901
O	-6.066470	-4.354063	2.494205
O	-3.986323	-2.000045	2.976951
Al	-5.259892	1.549337	0.485694
O	-4.931160	-0.216445	-0.306985
O	-4.935934	0.493087	2.045524
O	-1.714451	-2.744963	4.372229
O	-1.772430	-3.528359	1.994874
Si	-0.881731	-3.792818	0.606621
O	-0.781956	-5.226185	5.335472
O	-1.510872	-6.222172	2.844665
Si	-0.640200	-6.711662	1.508848
O	-1.425966	-7.919393	0.751964
Si	-2.682497	-8.924765	1.159990
O	-3.211931	-8.416647	2.612769
O	2.873983	-4.472673	4.906864
Al	2.960423	-2.873410	5.923934
O	1.382594	-6.698796	4.495867
Si	2.054045	-7.073766	3.063471
O	1.139775	-3.475979	6.151880
Si	3.913455	-4.776598	3.642684
O	3.216505	-5.935570	2.720161
O	4.005939	-3.365702	2.769302
Si	3.457999	-1.848700	3.008495
O	2.573847	-1.768000	4.404059

Al	2.022063	-0.165580	5.257935
O	2.528702	-1.246414	6.828182
O	0.894831	-7.125457	1.883089
O	-0.622997	-5.432489	0.478869
O	0.571261	-3.082842	0.918477
O	-5.838726	2.246102	-1.188036
O	-7.139950	1.241071	0.641908
O	-7.941009	3.779333	-0.240681
Al	-8.372772	4.850602	-1.763669
O	-6.508856	5.333375	-1.962018
Al	-6.606438	6.982915	-0.972578
O	-3.497449	2.121779	0.304779
Al	-3.414617	3.714153	1.253867
O	-1.532167	3.962766	1.239377
Al	-1.056194	3.180172	2.910253
O	-0.644452	2.103450	4.442888
O	-5.274533	3.245734	1.421401
O	-3.657511	4.861822	-0.267574
Al	-4.135543	6.529416	0.550429
O	-4.704110	7.290423	-1.072151
O	-3.712006	5.462454	2.146362
O	-2.901883	2.785444	2.870409
Si	-3.022618	4.780649	-1.797738
O	-1.971312	6.052782	-1.909971
Si	-1.282432	6.976165	-0.761824
O	0.067882	6.247175	-0.153107
Si	1.072430	6.587145	1.113976
O	2.143404	5.337221	1.156865
Si	1.841025	3.804848	1.651800
O	0.725205	3.886064	2.865327
Al	0.428624	5.411587	4.001156
O	-0.151743	7.023918	4.942441
Al	-0.325665	8.192887	3.384769
O	-2.210782	3.384926	-2.060305
O	-4.115396	4.773463	-2.998316
Si	-5.665912	5.227909	-3.383944
O	-8.380966	6.389547	-0.656334
O	-6.245004	3.955534	-4.260450
O	-2.354858	7.221331	0.457251
Al	-2.506940	8.872124	1.453567
O	-2.210569	7.988050	3.167506
O	-4.339165	8.224360	1.512554
O	-5.968898	6.158624	0.642887
O	-1.340640	4.733423	3.946169
O	-0.507471	1.548233	2.047594

Si	0.293265	1.487939	0.547459
O	0.188743	6.570745	2.513372
O	2.260957	5.848880	4.217806
Al	2.688226	4.813942	5.785217
O	0.831239	4.447148	5.570772
O	-0.685816	9.168920	1.833414
O	-4.546271	-4.700210	-1.804454
O	-5.300974	-7.172852	-1.324826
Si	-5.398500	-8.406968	-0.231787
O	-6.110386	-7.791799	1.106738
O	-3.883391	-8.964774	0.039843
O	-3.979688	-5.945880	1.487980
O	3.222328	3.110888	2.166772
Si	4.205002	3.300127	3.489441
O	1.206322	2.877255	0.455306
O	5.162775	1.966450	3.416075
Si	4.989230	0.477805	4.100782
O	3.279412	3.244179	4.861974
Al	3.613268	2.090635	6.365496
O	4.605089	-0.675603	2.999744
O	3.780069	0.548216	5.222080
O	2.523982	-1.490377	1.700602
O	-0.954121	1.601239	-0.582114
O	1.581981	0.676573	3.583197
O	0.159368	-0.530192	5.226413
O	1.847517	1.345773	6.341335
O	2.993059	3.648006	7.239157
H	0.342770	-3.038302	6.454068
H	-2.568383	-0.090537	4.558147
H	-5.699896	3.201047	2.281958
H	-9.063811	7.028029	-0.883757
H	1.032667	1.734427	6.658110
H	-2.055186	5.211161	3.500727
H	-4.727102	8.136052	2.389860
H	-4.028756	-4.508555	4.084711
H	-6.776060	-1.431090	2.475370
H	-9.917085	1.659105	0.210674
H	2.677567	3.726343	8.139655
H	-0.970440	6.796050	5.400071
H	-2.409516	-2.733135	5.037511
H	-4.430318	0.753820	2.818001
H	-8.245450	4.013137	0.637307
H	0.601611	3.518526	5.405549
H	-2.567308	7.092635	3.073857
H	-1.087703	-6.142631	5.371199

H	-4.287809	-2.915403	2.877452
H	-7.258990	0.281090	0.704784
H	1.849607	-1.338689	7.504422
H	-1.359681	2.131722	5.086162
H	-4.481512	5.402971	2.724947
H	-6.719777	-4.263458	3.194332
H	-9.090673	-0.705277	0.949514
H	-0.102919	-1.449436	5.047968
H	-2.946393	1.831107	2.703145
H	-6.175871	5.208909	0.639906
H	-5.365028	-7.806876	3.298693
H	-8.657664	-4.426851	1.182825
H	2.943761	6.014337	3.555250
H	-0.069074	9.677152	1.301677
H	0.850340	-3.687822	2.808513
H	-2.279926	-0.321492	0.639946
H	-5.543395	3.145520	-1.384799
H	1.854817	1.605549	3.560413
H	-1.100055	4.755563	0.899167
H	-4.289885	8.037723	-1.515914
H	-3.657761	-5.097322	1.153480
H	-6.747392	-1.806273	-1.019384
H	1.120693	-2.673425	0.232249
H	-3.385026	-2.402148	-2.626761
H	-1.561238	3.107021	-1.394553
H	2.111136	-0.617588	1.806193
H	-4.757861	-3.785833	-2.042574
H	-2.870924	1.832710	-0.372099
Si	2.225114	-0.003841	-1.309196
O	1.221373	0.126159	-0.026471
O	1.521086	0.473537	-2.735110
O	2.574526	-1.639241	-1.436262
O	3.600802	0.907220	-1.183165
H	3.526545	-1.847745	-1.457381
H	4.175130	0.742114	-0.421189
Si	-0.564370	-3.021927	-2.134927
O	-1.603332	-3.188593	-0.831934
O	-0.320386	-1.420809	-2.428812
O	-1.380838	-3.522238	-3.513314
O	0.871644	-3.833038	-2.042932
H	-1.898624	-4.330326	-3.432181
H	1.612696	-3.213558	-1.877860
Si	-2.527855	0.352193	-3.919111
O	-2.955586	0.507006	-2.303166
O	-0.895966	0.700908	-3.962662

O	-3.385102	1.437590	-4.829700
O	-2.790998	-1.093517	-4.676621
H	-3.590996	2.249028	-4.354812
H	-2.244217	-1.853753	-4.415583
Si	0.316322	-0.347592	-3.534004
C	0.967734	-1.159766	-5.072631
H	0.098453	-1.548495	-5.636981
H	1.363905	-0.304853	-5.655828
C	2.024052	-2.237422	-4.857379
H	2.860995	-1.826802	-4.256687
H	1.603078	-3.071630	-4.269465
C	2.582789	-2.807033	-6.148484
H	1.773214	-3.310051	-6.721867
H	3.349885	-3.571106	-5.926863
N	3.215503	-1.770104	-6.967124
C	3.664266	-2.223598	-8.274967
H	2.540341	-1.012657	-7.102617
C	4.289533	-1.075899	-9.046501
H	4.406850	-3.029756	-8.120528
H	2.838072	-2.661468	-8.872723
H	4.529426	-1.410696	-10.072775
H	3.545054	-0.265974	-9.146888
N	5.449133	-0.533219	-8.337642
H	6.277051	-1.108879	-8.501395
H	5.683929	0.403035	-8.665883
Au	5.499422	-1.739034	-3.391690
Au	6.889338	0.532891	-4.239372
Au	6.071053	-1.434102	-0.609270
Au	7.333974	1.002934	-1.459069
Au	8.669712	2.402843	-3.337500
Au	6.656837	0.544506	1.133569
Au	5.223839	-3.807652	-1.597443
Au	5.167211	-0.746020	-5.881343

Au_9/fHNT

312

O	-11.799851	1.460452	0.095319
O	-10.862108	4.031525	-1.017694
O	-9.666508	-2.210658	4.499979
O	-10.587818	3.180109	3.758573
O	-10.799402	0.610180	2.261424
O	2.838045	5.500887	-6.634986

O	-9.295048	7.171368	2.009711
O	5.437917	1.952723	-5.071168
O	3.751031	7.975854	-2.821663
O	6.115252	4.389488	-3.506339
O	-8.126922	-7.363968	2.168929
O	0.738979	-9.199064	-2.758245
O	-6.937145	-5.912986	4.108031
O	-10.938484	-3.316840	0.676880
O	-9.985266	-4.785101	2.654028
O	-1.965623	-8.327239	1.908255
O	1.910595	-7.036072	-6.066270
O	-1.274458	-10.060790	-4.007256
O	-4.091039	-10.620330	-1.851954
O	-3.749257	-9.730621	0.383552
O	-7.701257	-8.254843	-0.200498
O	-5.504337	10.138835	0.055688
O	-1.061374	10.401517	-1.573691
O	-7.085553	6.998148	-3.301557
O	0.948114	-8.296939	-0.157590
O	4.626668	-5.466713	-2.404329
O	6.663290	-0.862688	-3.699510
O	5.656358	-3.205574	-5.425762
O	4.480954	-1.759935	-7.156690
O	-1.723300	7.493117	-5.312226
O	4.179702	-6.419658	-4.876832
O	4.392734	3.130555	-7.056088
H	1.086578	-7.298095	-5.597747
H	-1.320218	-9.848313	-4.946969
H	-6.913020	7.224094	-4.225009
H	-10.887970	4.221116	-1.963756
H	-5.028848	-10.564168	-2.068447
H	2.107008	5.741729	-7.216597
H	4.170844	-0.842220	-6.935062
H	-10.790020	-2.350777	0.530438
H	-7.753170	-8.668991	0.679542
H	-2.442202	7.520367	-5.955776
H	4.048298	4.043423	-6.998451
H	-11.723846	2.398299	-0.161032
H	5.931562	-4.052731	-5.069669
H	-10.016008	-4.353460	3.515836
H	5.833301	1.113142	-4.815120
H	-10.605941	-0.132364	2.836187
H	-8.856068	7.690133	2.689088
H	-9.573819	-2.003946	5.434582
H	-7.304199	-6.645587	3.553312

H	-1.056860	-8.409927	2.218064
H	-10.607045	4.000707	4.260131
H	1.060076	-8.807557	-0.998148
H	4.679509	-5.941164	-3.275958
H	-6.359403	9.950063	0.449959
H	-1.418376	11.089689	-0.993525
H	3.257829	8.677831	-2.387645
H	6.415802	3.603865	-3.996049
H	6.961286	-1.542957	-4.324128
H	4.792571	-6.155245	-5.570173
H	-8.938532	-6.970706	2.520617
H	-3.129135	-9.564764	1.113776
H	1.423960	-8.652471	-3.163919
H	-0.314288	-10.225084	-3.829081
H	-4.070369	-10.807498	-0.871039
H	-6.783847	-8.388762	-0.531974
H	-11.428618	-3.407671	1.507869
H	-12.172627	1.439679	0.998416
H	-10.671765	4.891738	-0.569982
H	-6.960137	7.850521	-2.820261
H	-1.682610	8.381827	-4.893506
H	2.864639	6.186290	-5.914921
H	5.313147	3.144424	-6.738338
H	5.439514	-1.778203	-7.011908
H	2.605091	-7.637532	-5.747317
H	-4.057422	3.384754	-0.118665
H	-1.242417	-1.275349	1.715974
H	0.495251	1.357297	-1.805754
Al	-9.925157	0.921854	0.732991
O	-9.251947	1.760186	-0.897467
Al	-8.832552	3.501133	-0.336269
O	-8.187644	0.313049	1.284367
Al	-8.125309	-1.392506	0.425776
O	-8.779198	-2.295922	1.994015
Si	-8.315109	-2.091442	3.563283
O	-9.614346	2.745376	1.209529
Si	-9.275273	3.129965	2.788634
O	-9.933218	-0.807312	-0.068542
Si	-7.516356	0.669564	2.756909
O	-5.924111	0.926530	2.569399
Si	-4.589481	0.844201	1.585531
O	-4.026770	2.405252	1.580475
O	-8.185715	2.033901	3.346032
O	-7.706679	-0.587431	3.781567
O	-8.636028	4.644780	2.807360

Si	-8.475539	5.791958	1.625649
O	-6.885113	6.176031	1.594046
Si	-5.532296	6.099671	0.693627
O	-5.917143	5.623478	-0.870721
Al	-5.153239	6.421429	-2.411220
O	-4.315741	7.021696	-4.051473
Al	-2.492788	6.678094	-3.482874
O	-2.447730	4.997240	-4.322053
Al	-0.763551	4.305599	-3.740858
O	1.030220	3.941959	-3.283186
Al	1.909010	4.486134	-4.827182
O	-9.054260	5.223962	0.224255
O	-8.036894	4.088404	-1.977141
Al	-6.188342	3.825896	-1.459150
O	-4.474509	3.225670	-0.979814
Al	-4.451154	1.451985	-1.620204
O	-2.642308	1.117316	-1.207851
Al	-1.804973	1.695733	-2.779384
O	-0.132909	0.949718	-2.420704
Al	-0.128980	-0.663268	-3.333861
O	-1.932492	-0.041634	-3.645042
O	-7.031753	3.270069	0.168429
O	-6.219967	2.049114	-2.132282
O	-5.488855	4.657750	-3.085996
O	-3.608306	2.099690	-3.232114
Al	-5.486454	-1.182896	-0.757500
O	-5.075326	0.583588	-0.006042
O	-4.892734	-0.243357	-2.312649
O	-1.135989	2.558108	-4.389943
O	-1.376167	3.465641	-2.068803
Si	-0.620389	3.717173	-0.600608
O	0.133803	4.889245	-5.342450
O	-0.761715	6.076082	-2.993638
Si	0.001650	6.551560	-1.588907
O	-0.740272	7.864340	-0.976507
Si	-1.840586	8.959056	-1.565596
O	-2.255458	8.427452	-3.046923
O	3.630027	3.824248	-4.470902
Al	3.670322	2.173294	-5.404857
O	2.327238	6.196992	-4.323897
Si	2.871820	6.582013	-2.841198
O	1.952992	2.928950	-5.862641
Si	4.550077	4.095888	-3.110481
O	3.873172	5.360564	-2.321559
O	4.408532	2.729647	-2.174892

Si	3.744464	1.259442	-2.412071
O	3.014187	1.188604	-3.894472
Al	2.405027	-0.398034	-4.738751
O	3.183025	0.548506	-6.284500
O	1.601387	6.801736	-1.803183
O	-0.218556	5.331327	-0.511801
O	0.781383	2.861762	-0.715906
O	-6.309674	-1.735489	0.866947
O	-7.299144	-0.710667	-1.138709
O	-8.435685	-3.114850	-0.249005
Al	-9.133526	-4.061254	1.257934
O	-7.358467	-4.703322	1.686317
Al	-7.508134	-6.385827	0.760968
O	-3.818651	-1.905637	-0.354275
Al	-3.788669	-3.545882	-1.221443
O	-1.952358	-3.966355	-0.983102
Al	-1.222806	-3.318756	-2.619953
O	-0.543210	-2.365577	-4.138953
O	-5.564517	-2.916864	-1.618186
O	-4.306968	-4.585799	0.308560
Al	-4.853450	-6.242205	-0.489055
O	-5.667190	-6.862287	1.088441
O	-4.156209	-5.303104	-2.069162
O	-3.014445	-2.753571	-2.806182
Si	-3.837903	-4.484475	1.896259
O	-2.934380	-5.840807	2.179408
Si	-2.217843	-6.882166	1.155895
O	-0.744497	-6.313642	0.674910
Si	0.354246	-6.810216	-0.454856
O	1.540417	-5.668546	-0.427314
Si	1.444914	-4.142489	-1.016063
O	0.465545	-4.182999	-2.344041
Al	0.147098	-5.731383	-3.442404
O	-0.481772	-7.329767	-4.376292
Al	-0.937873	-8.395357	-2.801627
O	-2.927173	-3.158046	2.191594
O	-5.049165	-4.314102	2.963695
Si	-6.669413	-4.602682	3.189190
O	-9.171109	-5.648159	0.221147
O	-7.213638	-3.238521	3.941142
O	-3.169674	-7.089994	-0.165911
Al	-3.372626	-8.769065	-1.104220
O	-2.806167	-8.006183	-2.808375
O	-5.115426	-7.958642	-1.397457
O	-6.620760	-5.708824	-0.804240

O	-1.542877	-4.890650	-3.616599
O	-0.614759	-1.702068	-1.768512
Si	0.017141	-1.636526	-0.191966
O	-0.365671	-6.784742	-1.945007
O	1.940622	-6.346814	-3.431442
Al	2.635316	-5.438424	-4.981493
O	0.810760	-4.890930	-4.994540
O	-1.558669	-9.252211	-1.262320
O	-4.420138	5.084189	1.339531
O	-4.872957	7.587814	0.675911
Si	-4.729701	8.767353	-0.471021
O	-5.347975	8.152088	-1.855235
O	-3.146821	9.167892	-0.591628
O	-3.378888	6.099854	-1.915781
O	2.935251	-3.606817	-1.399027
Si	4.033137	-3.954367	-2.592664
O	0.776997	-3.099505	0.061448
O	5.102810	-2.712930	-2.466022
Si	5.151179	-1.252108	-3.226723
O	3.272699	-3.884302	-4.062535
Al	3.879599	-2.846029	-5.564654
O	4.763696	-0.012629	-2.224851
O	4.070483	-1.268460	-4.474186
O	2.642856	1.057249	-1.205029
O	-1.348709	-1.577127	0.793408
O	1.704775	-1.107979	-3.091551
O	0.594564	0.138333	-4.933942
O	2.202967	-1.940890	-5.771803
O	3.209307	-4.382575	-6.438199
H	1.154802	2.551750	-6.235075
H	-2.219624	-0.012074	-4.562134
H	-5.887100	-2.877706	-2.522562
H	-9.933746	-6.208141	0.395677
H	1.393501	-2.268450	-6.162762
H	-2.344973	-5.276556	-3.235876
H	-5.394864	-7.880435	-2.316020
H	-3.284518	4.540592	-4.439889
H	-6.478259	1.817803	-3.027489
H	-10.134211	-0.847290	-1.008704
H	2.987792	-4.477973	-7.364697
H	-1.219479	-7.051220	-4.932899
H	-1.752147	2.576118	-5.128777
H	-4.333758	-0.589391	-3.011333
H	-8.663902	-3.364694	-1.145502
H	0.656256	-3.937749	-4.894835

H	-3.081847	-7.077981	-2.792784
H	-0.075263	5.826942	-5.450363
H	-3.828033	3.042916	-3.205273
H	-7.316305	0.251657	-1.254180
H	2.594022	0.667991	-7.036685
H	-1.183332	-2.360909	-4.857554
H	-4.848503	-5.202810	-2.733321
H	-6.067599	4.591763	-3.851436
H	-9.005246	1.389101	-1.745682
H	0.405394	1.085894	-4.824435
H	-2.983538	-1.791828	-2.684491
H	-6.733096	-4.745171	-0.863887
H	-4.370076	7.984592	-3.947150
H	-8.188070	5.037604	-2.081381
H	2.527642	-6.540120	-2.689405
H	-1.056199	-9.787035	-0.643528
H	1.322662	3.339612	-2.585326
H	-2.339123	0.393963	-0.649374
H	-6.126817	-2.646941	1.132832
H	1.881478	-2.055768	-2.999708
H	-1.639424	-4.777000	-0.563679
H	-5.378801	-7.620673	1.606755
H	-3.179773	5.243651	-1.512238
H	-6.794478	2.369326	0.429492
H	1.210069	2.439448	0.044533
H	-3.585461	2.734343	2.382266
H	-2.184871	-2.976248	1.593011
H	2.160756	0.222064	-1.320776
H	-4.744704	4.206858	1.589540
H	-3.244489	-1.641035	0.376879
Si	1.871241	-0.231285	1.791220
O	0.989042	-0.322121	0.423366
O	1.011079	-0.560225	3.172695
O	2.416977	1.352226	1.882409
O	3.133493	-1.309698	1.791803
H	3.351652	1.431453	2.145802
H	3.649559	-1.342622	0.975282
Si	-0.667133	3.062583	2.185010
O	-1.544819	3.249038	0.771484
O	-0.599054	1.468661	2.594311
O	-1.577286	3.712935	3.438238
O	0.836633	3.742680	2.210555
H	-2.023270	4.546438	3.252446
H	1.540578	3.065443	2.155386
Si	-3.131021	-0.020481	3.869061

```
 O    -3.404994    -0.213614     2.224837
 O    -1.556966    -0.536219     4.096571
 O    -4.187428    -0.961887     4.727683
 O    -3.304656     1.481411     4.536520
 H    -4.433757    -1.771074     4.268398
 H    -2.662159     2.171804     4.299801
Si    -0.209877     0.383814     3.800509
 C     0.345606     1.196883     5.380707
 H    -0.553688     1.447364     5.973144
 H     0.870642     0.386446     5.924223
 C     1.235821     2.417599     5.175160
 H     2.037841     2.187532     4.444557
 H     0.646707     3.238004     4.732817
 C     1.878511     2.946404     6.440280
 H     1.098415     3.219920     7.183022
 H     2.449450     3.864806     6.213701
 N     2.823599     1.990525     7.030320
 C     3.441164     2.477587     8.261515
 H     2.322837     1.122233     7.239483
 C     4.537134     1.546429     8.747189
 H     3.871496     3.473592     8.044725
 H     2.686294     2.623357     9.060019
 H     4.828524     1.842485     9.773651
 H     4.132154     0.520189     8.819506
 N     5.646278     1.527504     7.804449
 H     6.224358     2.363921     7.893852
 H     6.257627     0.724882     7.948702
Au     4.487246     1.319584     5.452232
Au     5.669274     0.511935     3.077110
Au     6.681889    -0.436512     0.300797
Au     4.993918     3.201209     3.348332
Au     5.107362    -1.427583     4.976987
Au     6.097864     2.242010     0.942571
Au     6.129286    -2.161849     2.473270
Au     7.186479     1.586551    -1.478389
Au     6.859316    -3.155290     0.036558
```

Au_{10}/fHNT, 2D cluster

```
  313
 O    -8.064091    -7.402914    -1.153621
 O    -9.770468    -5.291462     0.011992
 O    -2.247881    -9.058804    -0.226779
```

O	-6.666021	-7.763410	2.816408
O	-5.855824	-7.962475	-0.046233
O	-5.773361	9.033640	-0.458981
O	-9.417926	-4.605958	4.582033
O	-1.195774	9.236263	-1.352822
O	-4.963537	8.588770	4.084888
O	-1.620829	9.734362	1.548302
O	0.826833	-8.220575	-5.146543
O	4.587720	0.849922	-8.281923
O	1.524365	-7.804285	-2.573381
O	-4.161080	-8.476375	-3.895306
O	-1.670235	-9.167925	-3.355879
O	4.820901	-3.430842	-4.894912
O	2.181389	4.140791	-8.893484
O	3.383399	-0.398407	-10.111626
O	3.231047	-3.931792	-9.492329
O	3.962954	-4.520804	-7.251066
O	0.463936	-6.946772	-7.345355
O	-10.186374	0.349128	5.942242
O	-8.653999	4.831103	5.776322
O	-10.711598	-0.180172	1.138156
O	5.419158	-0.027725	-5.800105
O	4.530202	4.943912	-4.759742
O	2.026872	8.657094	-2.120046
O	2.122745	8.005040	-5.128136
O	-0.356020	8.385705	-5.550484
O	-9.000601	5.306387	1.030003
O	3.661268	5.549501	-7.229432
O	-3.552811	9.768461	-2.114890
H	2.119941	3.159262	-8.893720
H	2.747870	0.108372	-10.630834
H	-11.223965	0.497979	0.678620
H	-10.382928	-4.772495	-0.523931
H	2.553941	-4.558313	-9.772520
H	-6.636788	8.815980	-0.830210
H	-1.020872	8.306000	-4.816245
H	-4.781774	-7.985224	-3.303466
H	1.146015	-7.563769	-7.024785
H	-9.748531	5.064277	0.469911
H	-4.316102	9.743119	-1.504947
H	-8.762490	-6.922766	-0.671173
H	3.011028	7.784410	-5.415345
H	-1.538210	-9.498362	-2.459580
H	-0.294383	9.165495	-1.683381
H	-4.972774	-8.327907	-0.122616

H	-9.167789	-4.439111	5.494703
H	-1.862343	-9.396259	0.587033
H	1.517257	-8.042540	-3.533760
H	5.545611	-2.883319	-4.572911
H	-6.962178	-7.781583	3.731434
H	5.395477	0.332896	-6.721625
H	4.433779	5.283941	-5.688567
H	-10.350511	-0.597038	5.935114
H	-9.015808	4.460618	6.594527
H	-5.484829	8.187401	4.785994
H	-1.182026	9.983444	0.715981
H	2.327781	9.005192	-2.974444
H	3.488780	6.476410	-7.422646
H	0.285516	-8.931037	-4.773355
H	4.572021	-4.342793	-6.514428
H	4.415745	1.775255	-8.065779
H	4.124688	0.232318	-9.929025
H	3.856435	-4.471921	-8.931256
H	0.905847	-6.081733	-7.506215
H	-3.963510	-9.321240	-3.463804
H	-7.809637	-8.168557	-0.602244
H	-10.000618	-5.101556	0.954123
H	-10.956630	-0.061968	2.086788
H	-9.348791	5.399498	1.944816
H	-5.845704	8.899513	0.523750
H	-2.880640	10.351021	-1.718498
H	0.272165	9.077062	-5.290599
H	3.128284	4.351763	-8.961977
H	-5.037510	-0.477204	1.409257
H	0.524670	-0.581563	-0.025261
H	-1.974640	3.417631	-0.360706
Al	-6.329212	-6.385936	-0.744787
O	-7.312035	-4.757044	-1.186575
Al	-7.931194	-4.169722	0.484780
O	-4.669481	-5.456938	-0.469395
Al	-3.949154	-5.494742	-2.238498
O	-2.943979	-7.096468	-1.881813
Si	-2.028424	-7.458978	-0.558886
O	-7.106350	-5.817422	0.904993
Si	-6.374526	-6.230025	2.337000
O	-5.604807	-6.516634	-2.502823
Si	-3.784281	-5.556845	0.927922
O	-3.142935	-4.102361	1.258496
Si	-2.822705	-2.555760	0.750526
O	-3.528609	-1.618578	1.927150

O	-4.760763	-5.974970	2.164059
O	-2.557213	-6.618088	0.742331
O	-6.992850	-5.259490	3.511473
Si	-8.243652	-4.176126	3.505838
O	-7.608424	-2.763205	4.033194
Si	-7.242127	-1.240885	3.591527
O	-7.932638	-0.900780	2.098350
Al	-8.792561	0.742823	1.707823
O	-9.531467	2.435526	1.123930
Al	-7.988009	3.506055	1.609969
O	-7.283912	3.452084	-0.131544
Al	-5.585322	4.297545	0.097934
O	-4.100888	5.394882	0.487238
Al	-4.731490	7.054539	-0.060672
O	-8.902502	-4.103060	2.028671
O	-8.685648	-2.514342	-0.116001
Al	-7.206030	-1.372526	0.395240
O	-5.601407	-0.423269	0.621888
Al	-4.749317	-0.624379	-1.048509
O	-3.298019	0.517027	-0.668200
Al	-3.988321	2.167884	-1.219107
O	-2.372498	3.100115	-1.185175
Al	-1.773157	3.071604	-2.938904
O	-3.358422	1.971277	-3.049035
O	-6.505880	-3.049859	0.999924
O	-6.399554	-1.602160	-1.310021
O	-8.167906	0.273658	-0.043809
O	-5.502066	1.072760	-1.578799
Al	-3.182024	-2.706562	-2.450894
O	-3.727295	-2.214831	-0.630103
O	-4.238345	-1.148747	-2.784174
O	-4.979279	3.790676	-1.631097
O	-4.546259	2.695721	0.578516
Si	-3.550137	2.636455	1.916307
O	-6.260057	6.013573	-0.459697
O	-6.367477	4.463588	1.845391
Si	-5.541732	4.508810	3.293901
O	-6.514081	4.004648	4.497758
Si	-8.148936	3.756654	4.645658
O	-8.778672	4.013322	3.166941
O	-3.144121	8.053215	0.038485
Al	-2.509743	8.052847	-1.749561
O	-5.360648	7.661135	1.549135
Si	-4.560821	7.462945	2.950636
O	-4.207163	7.137641	-1.854292

Si	-2.118438	8.184225	1.343029
O	-2.934037	7.627718	2.648825
O	-0.836952	7.173926	1.028284
Si	-0.371287	6.308323	-0.272727
O	-1.481692	6.453747	-1.489973
Al	-1.212837	5.905392	-3.286733
O	-2.164535	7.608723	-3.575691
O	-4.905194	5.980958	3.602456
O	-4.329498	3.408208	3.165129
O	-2.250028	3.556025	1.493514
O	-2.474079	-4.365069	-1.842554
O	-4.709866	-3.824599	-2.714975
O	-3.337200	-5.930894	-3.957048
Al	-2.359563	-7.549989	-3.680850
O	-0.718380	-6.538458	-3.507871
Al	-0.166818	-6.705639	-5.345621
O	-1.561505	-1.793284	-2.370439
Al	-0.906522	-1.832637	-4.106161
O	0.529344	-0.606958	-3.902111
Al	-0.300044	1.011763	-4.470168
O	-1.298494	2.624436	-4.753390
O	-2.523789	-2.864929	-4.266155
O	0.245635	-3.347888	-3.846412
Al	0.619026	-3.891065	-5.647537
O	1.352929	-5.538829	-5.117039
O	-0.390825	-2.237467	-5.980117
O	-1.779068	-0.159953	-4.529723
Si	1.240682	-3.747519	-2.581006
O	2.781706	-3.582563	-3.159106
Si	3.356295	-2.806099	-4.467687
O	3.579448	-1.201874	-4.148350
Si	3.961468	0.103579	-5.086459
O	3.901729	1.394510	-4.066134
Si	2.555024	2.086906	-3.440395
O	1.360850	1.962828	-4.573024
Al	1.642335	1.789270	-6.469319
O	1.862807	1.267054	-8.340451
Al	3.089406	-0.228828	-8.056071
O	1.038054	-2.757444	-1.294633
O	0.976872	-5.212093	-1.932137
Si	0.359413	-6.718270	-2.263050
O	-1.862736	-7.540933	-5.510519
O	-0.464479	-7.118680	-0.890672
O	2.290209	-2.962674	-5.706756
Al	2.802810	-3.165043	-7.560720

O	1.771846	-1.607691	-8.125326
O	1.137554	-4.167605	-7.516932
O	-0.890011	-4.987414	-5.815017
O	0.046843	0.777927	-6.311800
O	-0.585617	1.564830	-2.647777
Si	0.522952	1.291054	-1.386149
O	2.790094	0.290911	-6.240699
O	3.067508	3.036254	-6.566879
Al	2.091343	4.663059	-6.900103
O	0.691937	3.371282	-6.856509
O	4.068415	-1.774896	-7.681547
O	-5.623898	-0.987901	3.540293
O	-7.849868	-0.243858	4.725633
Si	-9.114182	0.817911	4.763644
O	-9.755961	0.835889	3.258765
O	-8.538578	2.275124	5.238720
O	-7.327715	1.818284	2.149190
O	2.858393	3.644817	-3.072097
Si	3.110511	5.027940	-3.952054
O	2.034002	1.322672	-2.084174
O	2.969112	6.205490	-2.814167
Si	1.660536	7.080987	-2.328226
O	1.897159	5.186096	-5.068502
Al	0.810291	6.757894	-5.294051
O	1.132238	6.641050	-0.839080
O	0.433412	6.841899	-3.405881
O	-0.259412	4.746224	0.235900
O	0.199668	-0.290431	-0.898779
O	-0.320900	4.285748	-2.749293
O	-2.688205	4.714210	-3.375835
O	-0.836234	5.793868	-5.112696
O	0.997064	6.190698	-7.087581
H	-4.599869	6.569369	-2.518328
H	-3.999953	2.216204	-3.721960
H	-3.185321	-2.651981	-4.929870
H	-1.841827	-8.415198	-5.911619
H	-1.277066	5.241232	-5.757397
H	0.034858	-0.179419	-6.455235
H	0.467846	-3.900774	-8.155879
H	-7.519055	2.698778	-0.679140
H	-6.843222	-1.425459	-2.142822
H	-6.163573	-6.212112	-3.224590
H	0.469732	6.454714	-7.841759
H	0.983472	1.042910	-8.669302
H	-5.710614	3.676811	-2.245944

H	-4.045004	-0.450294	-3.412768
H	-3.752339	-5.735059	-4.798190
H	0.031098	3.489637	-6.155332
H	1.016472	-1.551642	-7.521945
H	-7.045714	6.188945	0.075671
H	-6.229878	1.172701	-0.947154
H	-5.406545	-3.483524	-2.133603
H	-2.953491	7.554710	-4.124886
H	-2.024557	2.476909	-5.367574
H	-1.181737	-2.425222	-6.499392
H	-8.836430	0.177566	-0.728674
H	-7.354343	-4.241265	-1.992935
H	-3.360124	4.797976	-2.677870
H	-2.329324	0.098916	-3.774130
H	-1.613591	-4.750846	-5.210639
H	-10.139526	2.635053	1.852942
H	-9.444145	-2.290756	0.440228
H	3.898516	3.071120	-6.076178
H	5.013001	-1.850380	-7.528330
H	-3.191693	5.089530	0.611638
H	-2.373428	0.254006	-0.723121
H	-1.641317	-4.633821	-2.253856
H	0.444945	4.089428	-3.308665
H	1.446881	-0.818246	-4.113002
H	2.271908	-5.804092	-5.226947
H	-6.452912	1.509551	1.875363
H	-5.651677	-3.269115	0.602363
H	-1.349892	3.383732	1.810492
H	-3.091975	-1.570576	2.794880
H	1.040455	-1.800115	-1.455507
H	-0.044849	4.160531	-0.508598
H	-5.109232	-1.646031	3.050865
H	-1.042600	-1.622305	-1.573093
Si	1.663093	2.215552	1.303057
O	0.543815	2.167425	0.122817
O	2.039824	0.712160	1.910989
O	0.980175	3.114315	2.556730
O	3.040410	3.021556	0.886665
H	1.479460	3.926365	2.709737
H	3.829246	2.482742	0.701021
Si	-1.757055	0.965728	3.387102
O	-3.106036	1.051580	2.402105
O	-0.516003	0.351932	2.497490
O	-2.056626	-0.215925	4.543384
O	-1.274403	2.338750	4.166684

H	-2.947453	-0.227007	4.910372
H	-0.479348	2.730869	3.753324
Si	-0.287093	-2.828899	2.014662
O	-1.133849	-2.274706	0.676842
O	1.019719	-1.798024	2.178136
O	0.279915	-4.361112	1.739417
O	-1.076071	-2.918642	3.463751
H	0.421828	-4.557076	0.807293
H	-1.265298	-2.080544	3.918490
Si	1.010128	-0.255322	2.796371
C	1.381140	-0.252053	4.617826
H	0.398390	-0.371714	5.119094
H	1.962843	-1.169377	4.833515
C	2.103730	0.992184	5.131173
H	3.167178	0.948806	4.820873
H	1.683915	1.902501	4.668072
C	2.016886	1.151361	6.637908
H	0.966653	1.398586	6.920078
H	2.641349	2.005949	6.958435
N	2.494241	-0.028932	7.342686
C	2.465977	0.081857	8.784481
H	1.953469	-0.846020	7.049676
C	3.149401	-1.100544	9.445403
H	2.996361	1.013394	9.065343
H	1.438283	0.179910	9.197925
H	2.962690	-1.070723	10.534825
H	2.709238	-2.040374	9.066086
N	4.586559	-1.130000	9.139761
H	5.060068	-0.350772	9.602261
H	5.016115	-1.980660	9.505478
Au	4.998020	-0.990834	6.832477
Au	5.231454	-0.887888	4.079574
Au	4.925057	-0.401503	1.263235
Au	6.619978	0.970753	5.610835
Au	4.079057	-3.152769	5.194058
Au	6.272513	1.461030	2.867979
Au	3.720936	-2.654437	2.449137
Au	6.170462	1.784924	0.093694
Au	3.659102	-2.255628	-0.326088
Au	4.678179	0.058906	-1.531015

Au_{10}/fHNT, 3D cluster

313

O	3.985036	-8.644395	4.489249
O	6.662756	-7.801238	3.568643
O	-1.593368	-8.278068	2.004017
O	3.492324	-9.744259	0.440397
O	2.065405	-8.691072	2.837370
O	9.451952	6.308066	-0.246478
O	7.675352	-8.563511	-0.889278
O	5.370216	8.439546	-1.014933
O	9.559693	4.733864	-4.606610
O	6.604188	7.774788	-3.638038
O	-4.981633	-4.826491	5.315654
O	-4.912741	5.478545	5.091242
O	-4.831192	-5.047878	2.630625
O	-0.488044	-7.269911	5.905237
O	-2.837616	-7.144084	4.706394
O	-6.265088	0.723567	2.764757
O	-1.497919	7.675693	5.802400
O	-4.827903	4.547361	7.432656
O	-6.127778	1.206224	7.629222
O	-6.527381	0.227979	5.440787
O	-4.588732	-3.130277	7.201792
O	10.845262	-4.960910	-2.880874
O	11.469229	-0.438007	-4.152821
O	10.000008	-4.048806	1.819008
O	-5.473577	4.206180	2.706388
O	-2.262673	7.883637	1.046179
O	2.145398	9.355033	-1.283255
O	1.105033	9.800641	1.580669
O	3.331999	9.369716	2.730662
O	10.927985	1.397015	0.230476
O	-1.787466	8.900960	3.487564
O	7.483446	8.295787	0.375451
H	-1.880634	6.796735	6.021798
H	-4.166359	4.927887	8.022459
H	10.644018	-3.493394	2.277651
H	7.305979	-7.396255	4.163532
H	-5.880516	0.504587	8.242487
H	10.022352	5.925249	0.430865
H	4.036620	8.819366	2.296948
H	0.400230	-7.259566	5.472063
H	-5.383721	-3.525377	6.801079
H	11.345366	1.096354	1.047254
H	8.270076	7.797605	0.078297
H	4.911597	-8.636254	4.185143

H	0.172253	10.024427	1.588644
H	-2.900104	-7.676500	3.904770
H	4.483004	8.813014	-1.001851
H	1.119638	-8.664263	2.682621
H	7.734871	-8.624291	-1.846429
H	-1.897340	-8.697619	1.193841
H	-5.143966	-4.945572	3.563816
H	-6.579481	1.359919	2.112922
H	3.944364	-10.165810	-0.296669
H	-5.497315	4.812523	3.488427
H	-2.234052	8.447952	1.863717
H	10.565871	-5.844079	-2.627539
H	11.800280	-1.159389	-4.707472
H	9.989468	3.965720	-4.993439
H	6.149245	8.422781	-3.071928
H	1.849239	10.046531	-0.670198
H	-1.268880	9.710256	3.536158
H	-4.744540	-5.764874	5.299446
H	-6.813521	0.364715	4.521706
H	-4.304471	6.153308	4.763762
H	-5.150666	5.307778	6.886517
H	-6.786046	0.779265	7.011124
H	-4.623761	-2.162260	7.025254
H	-0.939073	-8.076102	5.612579
H	3.546420	-9.399820	4.051167
H	7.155947	-8.026026	2.742273
H	10.475714	-4.343695	1.006192
H	11.474580	1.053760	-0.511266
H	9.673234	5.844576	-1.098059
H	7.246726	8.919400	-0.334045
H	3.151496	10.116780	2.139511
H	-2.241320	8.226241	5.502298
H	5.003052	-2.334473	-0.290131
H	-0.190473	0.062754	-0.832474
H	3.681779	2.754654	-0.487109
Al	3.021605	-7.259491	3.320910
O	4.500233	-6.050976	3.730266
Al	5.669436	-6.307787	2.285016
O	2.054815	-5.935234	2.318382
Al	1.020147	-5.130157	3.708223
O	-0.484739	-6.279110	3.363991
Si	-1.146917	-6.693762	1.911622
O	4.314798	-7.583094	1.952750
Si	3.814051	-8.143776	0.472426
O	1.944649	-6.537630	4.717913

Si	1.551163	-6.155742	0.754998
O	1.716220	-4.760780	-0.060509
Si	2.012271	-3.128727	-0.014513
O	3.302094	-2.951604	-1.045975
O	2.488194	-7.278393	0.033733
O	-0.025675	-6.575798	0.724798
O	5.042498	-7.903948	-0.593905
Si	6.608190	-7.411046	-0.384440
O	6.801449	-6.118760	-1.369640
Si	7.061709	-4.513048	-1.395369
O	7.480814	-3.980717	0.141847
Al	8.870670	-2.731399	0.459530
O	10.134382	-1.332260	0.904057
Al	9.378030	0.004674	-0.281296
O	8.356449	0.780736	1.091876
Al	7.308427	2.061302	0.135326
O	6.593841	3.431578	-0.946551
Al	7.756851	4.829511	-0.565058
O	6.884745	-7.105503	1.181315
O	6.926320	-4.941381	2.757873
Al	6.262640	-3.573620	1.556512
O	5.341727	-2.235750	0.613348
Al	4.142331	-1.557795	1.900910
O	3.473802	-0.157823	0.829613
Al	4.684110	1.212128	1.235818
O	3.703166	2.601445	0.469483
Al	2.780986	3.365751	1.884204
O	3.643862	1.865072	2.744291
O	5.044003	-4.980202	1.102929
O	5.082213	-2.925509	2.897252
O	7.731365	-2.343307	1.952811
O	5.431720	-0.177000	2.299886
Al	1.545412	-2.348514	3.080109
O	2.640864	-2.710749	1.492571
O	3.080506	-1.264124	3.429101
O	6.173642	2.402251	1.621749
O	5.801726	0.883192	-0.334050
Si	5.206024	0.757407	-1.888747
O	8.532723	3.495446	0.529278
O	8.448983	1.352578	-1.239558
Si	8.073524	1.220997	-2.859559
O	8.960925	0.034799	-3.534910
Si	10.302662	-0.823970	-3.067422
O	10.635160	-0.347079	-1.547206
O	6.844616	6.245708	-1.395202

Al	5.897036	7.058374	0.033508
O	8.929642	4.605926	-1.954267
Si	8.458344	4.267050	-3.472901
O	6.940641	5.676848	0.889126
Si	6.301994	6.307817	-2.967686
O	7.052494	5.100341	-3.779307
O	4.671026	5.994209	-2.907858
Si	3.593620	5.831717	-1.694781
O	4.351956	5.951661	-0.229724
Al	3.476328	6.156024	1.441368
O	4.994886	7.391051	1.684753
O	8.243597	2.636075	-3.655825
O	6.504185	0.741245	-2.928381
O	4.393757	2.171093	-2.126232
O	0.329415	-3.737132	2.616863
O	2.316585	-3.793590	4.065954
O	-0.086170	-4.728635	5.168693
Al	-1.592687	-5.876543	4.911299
O	-2.529551	-4.453602	3.992519
Al	-3.490377	-3.800440	5.528651
O	0.562478	-0.988812	2.273352
Al	-0.408685	-0.219658	3.654878
O	-1.065377	1.305540	2.733691
Al	0.247698	2.600878	3.209474
O	1.767807	3.738954	3.481494
O	0.501096	-1.656198	4.557812
O	-2.024192	-1.208185	3.332565
Al	-2.989247	-0.959874	4.971315
O	-4.240507	-2.304210	4.568468
O	-1.451955	0.224808	5.284465
O	0.997710	1.061163	4.001321
Si	-2.784624	-1.607610	1.913663
O	-4.177254	-0.715331	1.890169
Si	-4.621477	0.597469	2.741439
O	-4.031713	1.974756	2.048191
Si	-3.991597	3.558609	2.517235
O	-3.139920	4.331425	1.338591
Si	-1.524410	4.242393	1.079547
O	-0.794351	4.069180	2.549616
Al	-1.536593	4.637672	4.232202
O	-2.375180	4.871494	5.982783
Al	-4.041255	3.918939	5.608719
O	-1.883490	-1.236122	0.599876
O	-3.062210	-3.193183	1.702616
Si	-3.268728	-4.624399	2.520114

O	-2.425928	-5.092070	6.422901
O	-2.426905	-5.720341	1.618558
O	-4.040610	0.477039	4.272594
Al	-4.986838	1.090614	5.843917
O	-3.525458	2.259198	6.396907
O	-3.977000	-0.403853	6.569808
O	-2.203551	-2.410614	5.857466
O	-0.565960	3.123794	4.831757
O	1.145547	2.388404	1.518411
Si	0.341904	2.140579	0.041649
O	-3.147746	3.672445	3.936539
O	-2.241437	6.275736	3.587543
Al	-0.745432	7.449473	3.895306
O	-0.094866	5.799247	4.589869
O	-5.494705	2.803244	5.245727
O	5.758074	-3.687503	-1.947002
O	8.283780	-4.233104	-2.433530
Si	9.861292	-3.778182	-2.255247
O	10.092410	-3.505481	-0.658666
O	10.114380	-2.451870	-3.181174
O	8.174832	-1.404177	-0.659158
O	-1.014047	5.592471	0.323469
Si	-0.813821	7.177963	0.767289
O	-1.111647	2.944542	0.164392
O	0.084488	7.783709	-0.468635
Si	1.717079	7.914056	-0.649398
O	0.061898	7.238774	2.171973
Al	1.653458	8.289208	2.429344
O	2.310672	6.852989	-1.750299
O	2.436902	7.610498	0.804586
O	2.915498	4.343237	-1.884358
O	0.029188	0.483411	0.020704
O	2.101919	4.891406	0.973019
O	4.208097	4.609117	2.263471
O	2.694683	6.790372	3.014676
O	0.841400	8.446262	4.129119
H	6.881738	5.254452	1.747149
H	4.160232	2.065058	3.530183
H	1.022610	-1.494710	5.348665
H	-2.921428	-5.716894	6.961154
H	2.688786	6.357834	3.868058
H	-1.012747	2.330605	5.161063
H	-3.418945	-0.206188	7.329718
H	8.104416	0.216155	1.826978
H	5.360995	-2.661204	3.777079

H	2.404765	-6.239769	5.508778
H	1.249049	8.730753	4.947323
H	-1.784450	4.463440	6.627874
H	6.621443	2.237380	2.457315
H	3.083478	-0.379517	3.800244
H	0.174513	-4.434557	6.042643
H	0.686915	5.433989	4.145148
H	-2.710998	1.837064	6.086644
H	9.411387	3.188599	0.267685
H	6.248055	-0.560097	1.945579
H	3.201792	-3.938474	3.698438
H	5.533851	7.236110	2.467384
H	2.196199	3.546427	4.321454
H	-0.964062	-0.057324	6.067280
H	8.117000	-2.447019	2.827875
H	4.587132	-5.353492	4.381415
H	4.983335	4.210901	1.832153
H	1.758020	0.841027	3.440783
H	-1.336283	-2.663951	5.499053
H	10.912590	-1.616550	0.399489
H	7.807452	-5.203053	2.458147
H	-2.838385	6.448295	2.848328
H	-6.314148	3.030695	4.800536
H	5.696623	3.454929	-1.306828
H	2.542226	-0.033516	0.620348
H	-0.604111	-3.535065	2.767357
H	1.225800	5.180581	1.267666
H	-2.002371	1.523130	2.659795
H	-5.180416	-2.165986	4.412130
H	7.217587	-1.266552	-0.641113
H	4.116936	-4.731662	1.223572
H	3.606235	2.245232	-2.687041
H	3.135433	-3.032912	-2.000828
H	-1.496032	-0.347263	0.553427
H	2.303876	4.152522	-1.154357
H	4.910387	-3.915480	-1.538323
H	0.364979	-0.910721	1.330420
Si	0.355683	2.483566	-2.986564
O	1.055227	2.419906	-1.528520
O	-0.507915	1.110182	-3.389538
O	1.578646	2.598410	-4.133797
O	-0.657805	3.784540	-3.100464
H	1.744482	3.507501	-4.412867
H	-1.491165	3.626989	-3.565581
Si	3.241480	-0.526221	-3.519272

O	4.216749	-0.619165	-2.166372
O	1.682388	-0.307434	-3.029699
O	3.189934	-2.049696	-4.225501
O	3.632557	0.596268	-4.661783
H	4.037830	-2.481834	-4.377942
H	2.992526	1.334013	-4.668698
Si	-0.038468	-2.848818	-1.965876
O	0.664496	-2.236375	-0.582757
O	-0.679715	-1.529557	-2.774780
O	-1.240667	-3.944237	-1.654625
O	0.922954	-3.667916	-3.049820
H	-2.060235	-3.540884	-1.322653
H	1.614512	-3.166570	-3.518301
Si	0.152541	-0.409161	-3.671872
C	0.166009	-0.795301	-5.487697
H	0.924247	-1.585479	-5.655392
H	0.594425	0.109183	-5.957008
C	-1.178507	-1.171477	-6.140634
H	-2.038929	-0.834438	-5.524243
H	-1.279422	-0.641736	-7.101495
C	-1.326113	-2.657302	-6.424491
H	-0.473441	-2.985487	-7.062984
H	-2.246136	-2.825960	-7.015318
N	-1.392831	-3.453212	-5.205275
C	-1.133096	-4.876606	-5.364443
H	-0.737308	-3.087667	-4.516578
C	-2.398729	-5.691311	-5.558731
H	-0.617345	-5.224807	-4.451180
H	-0.446148	-5.074121	-6.213569
H	-2.959183	-5.324916	-6.437503
H	-2.119496	-6.742452	-5.765149
N	-3.264249	-5.558578	-4.385886
H	-4.123524	-6.097450	-4.490799
H	-2.790271	-5.911728	-3.550585
Au	-3.577209	-1.367725	-1.808161
Au	-4.576203	-0.587815	-4.465825
Au	-6.158696	-2.314975	-2.920415
Au	-4.794771	0.919292	-0.659720
Au	-5.799209	1.661691	-3.327984
Au	-7.523775	-0.114150	-1.875489
Au	-7.061938	2.566432	-0.936736
Au	-3.744450	-3.225828	-4.036818
Au	-3.056598	1.306114	-2.992668
Au	-5.934327	-1.688313	-0.154470

Au_{11}/fHNT, 2D cluster

```
 314
O    11.981449   -2.330994    0.328489
O    10.852328   -4.831400   -0.767682
O    10.068831    1.586305    4.620997
O    10.539787   -3.875799    3.986476
O    11.007118   -1.360168    2.455475
O    -2.787143   -5.219740   -6.702424
O     8.949769   -7.771700    2.267839
O    -5.108013   -1.434886   -5.259242
O    -3.999531   -7.539957   -2.875263
O    -6.029364   -3.776759   -3.674300
O     9.034087    6.810807    2.172715
O     0.477811    9.312091   -3.002470
O     7.678391    5.501440    4.103689
O    11.521146    2.512028    0.814291
O    10.651522    4.091099    2.744011
O     2.986481    8.293837    1.743149
O    -0.797342    7.199661   -6.304875
O     2.586865    9.976009   -4.213929
O     5.389542   10.329901   -1.997782
O     4.919790    9.512183    0.241985
O     8.742717    7.693110   -0.220125
O     4.964776  -10.437265    0.265804
O     0.555367  -10.346813   -1.469919
O     6.889286   -7.503135   -3.099072
O     0.130495    8.477054   -0.394203
O    -3.724068    5.933448   -2.688147
O    -6.118430    1.498851   -3.962416
O    -4.873073    3.716121   -5.699014
O    -3.786010    2.144870   -7.377413
O     1.553251   -7.571996   -5.235313
O    -3.138518    6.800979   -5.163157
O    -4.121600   -2.732662   -7.198927
H     0.034895    7.398296   -5.820003
H     2.636312    9.743909   -5.148801
H     6.719768   -7.729659   -4.022941
H    10.884076   -5.039107   -1.709752
H     6.323877   10.189778   -2.189858
H    -2.066046   -5.532533   -7.261771
H    -3.561383    1.207993   -7.133916
H    11.293548    1.559818    0.679157
H     8.809324    8.116688    0.654419
```

H	2.282115	-7.672062	-5.860183
H	-3.858500	-3.670535	-7.118566
H	11.831045	-3.263232	0.084874
H	-5.082634	4.589873	-5.363075
H	10.624649	3.673625	3.612898
H	-5.435566	-0.560140	-5.026193
H	10.864808	-0.593790	3.013654
H	8.451851	-8.238915	2.943975
H	9.936668	1.404736	5.556134
H	8.120143	6.190025	3.547047
H	2.081158	8.459534	2.028817
H	10.476451	-4.686083	4.501004
H	0.082772	8.980537	-1.245104
H	-3.715288	6.395289	-3.568066
H	5.823381	-10.315567	0.678366
H	0.838005	-11.052744	-0.870420
H	-3.578976	-8.273882	-2.418229
H	-6.249519	-2.976998	-4.183478
H	-6.341931	2.191072	-4.604749
H	-3.755353	6.577917	-5.867431
H	9.800301	6.355685	2.550639
H	4.270640	9.412941	0.958952
H	-0.241930	8.819167	-3.416637
H	1.640473	10.225092	-4.062388
H	5.361933	10.535362	-1.020707
H	7.848318	7.899312	-0.576475
H	11.997544	2.575110	1.655772
H	12.333227	-2.326371	1.240200
H	10.578118	-5.664134	-0.311604
H	6.679627	-8.333033	-2.607923
H	1.426327	-8.446453	-4.804032
H	-2.889631	-5.887583	-5.972724
H	-5.047027	-2.662012	-6.904119
H	-4.742584	2.247763	-7.256950
H	-1.444801	7.863940	-6.012742
H	4.109297	-3.588143	-0.049237
H	1.663505	1.327591	1.642054
H	-0.210834	-1.207944	-1.880847
Al	10.145578	-1.622586	0.910665
O	9.441329	-2.428665	-0.722944
Al	8.860471	-4.117131	-0.145521
O	8.454417	-0.857525	1.408921
Al	8.559439	0.831745	0.522730
O	9.251512	1.703224	2.092662
Si	8.734655	1.566881	3.652809

O	9.667757	-3.404105	1.407458
Si	9.259678	-3.730407	2.983354
O	10.321398	0.085173	0.082907
Si	7.720387	-1.129311	2.869537
O	6.116862	-1.252027	2.646330
Si	4.818391	-1.073111	1.628004
O	4.122942	-2.579790	1.633253
O	8.255698	-2.535376	3.496287
O	7.994006	0.124614	3.879071
O	8.492114	-5.184302	3.009540
Si	8.261363	-6.334097	1.842089
O	6.644963	-6.580952	1.776671
Si	5.325360	-6.404772	0.841626
O	5.786569	-5.990880	-0.719794
Al	4.993345	-6.747379	-2.266375
O	4.146194	-7.302398	-3.917570
Al	2.346694	-6.793892	-3.400129
O	2.466336	-5.130367	-4.266098
Al	0.834692	-4.286790	-3.737989
O	-0.931367	-3.762813	-3.331000
Al	-1.817084	-4.256723	-4.887882
O	8.919733	-5.842504	0.446980
O	8.056127	-4.662801	-1.796489
Al	6.225340	-4.233774	-1.329017
O	4.558722	-3.480547	-0.902043
Al	4.703229	-1.722872	-1.570206
O	2.920763	-1.227264	-1.208454
Al	2.074058	-1.759325	-2.791279
O	0.464415	-0.866567	-2.486126
Al	0.620921	0.724535	-3.423967
O	2.371053	-0.054762	-3.680281
O	7.074854	-3.723754	0.310344
O	6.425703	-2.478347	-2.028578
O	5.495395	-5.031084	-2.959774
O	3.846061	-2.324236	-3.192470
Al	5.941006	0.828287	-0.722783
O	5.361675	-0.883023	0.045471
O	5.305405	-0.084085	-2.277555
O	1.371447	-2.589364	-4.404528
O	1.477807	-3.473257	-2.064373
Si	0.668431	-3.633489	-0.611059
O	-0.071528	-4.819484	-5.352284
O	0.662827	-6.037235	-2.963718
Si	-0.171621	-6.420761	-1.571331
O	0.439915	-7.781350	-0.920305

Si	1.455476	-8.976519	-1.464674
O	1.949404	-8.508542	-2.943215
O	-3.482643	-3.443624	-4.585043
Al	-3.358597	-1.811961	-5.545165
O	-2.392824	-5.916337	-4.368782
Si	-3.003355	-6.227152	-2.894382
O	-1.702391	-2.719942	-5.948101
Si	-4.454497	-3.611464	-3.244016
O	-3.907794	-4.915437	-2.418924
O	-4.217947	-2.246149	-2.326445
Si	-3.424347	-0.842660	-2.569677
O	-2.655922	-0.860732	-4.034270
Al	-1.892666	0.652781	-4.887517
O	-2.712599	-0.250624	-6.437397
O	-1.781397	-6.536664	-1.821645
O	0.127495	-5.205230	-0.508113
O	-0.651020	-2.662977	-0.775307
O	6.770186	1.336766	0.912994
O	7.714759	0.195828	-1.051186
O	9.032787	2.509030	-0.170659
Al	9.773786	3.418485	1.338427
O	8.050996	4.217744	1.712290
Al	8.366759	5.864709	0.765033
O	4.332559	1.698288	-0.372621
Al	4.464376	3.319577	-1.265558
O	2.665974	3.899997	-1.079765
Al	1.922275	3.288611	-2.724091
O	1.199072	2.370649	-4.244710
O	6.188348	2.533800	-1.607982
O	5.034059	4.337972	0.260632
Al	5.739792	5.927176	-0.548617
O	6.566466	6.502867	1.038946
O	5.001745	5.023659	-2.130937
O	3.662403	2.568800	-2.856712
Si	4.520693	4.305140	1.837482
O	3.730885	5.738680	2.076912
Si	3.131013	6.819466	1.019798
O	1.625944	6.370935	0.510976
Si	0.600991	6.939912	-0.653468
O	-0.679394	5.904759	-0.637927
Si	-0.701740	4.366040	-1.200407
O	0.308489	4.299109	-2.503943
Al	0.784888	5.794955	-3.617842
O	1.570899	7.316905	-4.560344
Al	2.079789	8.366996	-2.991452

O	3.492424	3.067037	2.130450
O	5.687272	4.050375	2.937387
Si	7.320579	4.202955	3.198838
O	9.972310	4.977863	0.278458
O	7.727437	2.810600	3.985197
O	4.128115	6.921694	-0.280849
Al	4.496961	8.560444	-1.239675
O	3.907272	7.819081	-2.945448
O	6.169939	7.598615	-1.476651
O	7.461555	5.238841	-0.811208
O	2.399844	4.809545	-3.736685
O	1.157264	1.745175	-1.863232
Si	0.484852	1.762790	-0.302129
O	1.350988	6.826650	-2.124576
O	-0.948743	6.561927	-3.661266
Al	-1.682318	5.689313	-5.213955
O	0.088120	4.987323	-5.172888
O	2.735577	9.194416	-1.450553
O	4.289844	-5.286519	1.443713
O	4.540938	-7.831015	0.830470
Si	4.323744	-9.013912	-0.301285
O	5.025175	-8.478320	-1.678964
O	2.715542	-9.279381	-0.455226
O	3.242081	-6.266267	-1.820505
O	-2.223258	3.953393	-1.612183
Si	-3.258683	4.372723	-2.838140
O	-0.151865	3.288711	-0.090573
O	-4.433964	3.229944	-2.719083
Si	-4.589978	1.765499	-3.458045
O	-2.472645	4.211925	-4.287272
Al	-3.131091	3.203244	-5.787843
O	-4.334396	0.515173	-2.427725
O	-3.482766	1.667247	-4.678195
O	-2.338198	-0.714437	-1.338738
O	1.817029	1.602871	0.717925
O	-1.172933	1.328943	-3.234510
O	-0.131004	-0.040121	-5.029042
O	-1.534203	2.154246	-5.938956
O	-2.310570	4.661102	-6.668037
H	-0.866125	-2.419139	-6.306252
H	2.676116	-0.124932	-4.589342
H	6.527567	2.451254	-2.503306
H	10.776002	5.473408	0.463315
H	-0.690544	2.404308	-6.314574
H	3.222968	5.131939	-3.342024

H	6.463193	7.480616	-2.386617
H	3.341880	-4.749257	-4.370024
H	6.723996	-2.285790	-2.920520
H	10.547206	0.091249	-0.852441
H	-2.059872	4.720867	-7.590071
H	2.294806	6.966422	-5.093948
H	2.001024	-2.673097	-5.127354
H	4.794943	0.296244	-2.995276
H	9.302751	2.722603	-1.064929
H	0.157584	4.026308	-5.054604
H	4.101564	6.871087	-2.908597
H	0.058520	-5.773398	-5.440408
H	3.983057	-3.282205	-3.145537
H	7.651719	-0.766304	-1.151269
H	-2.118495	-0.433348	-7.172677
H	1.853176	2.298516	-4.946917
H	5.698305	4.852746	-2.775943
H	6.095564	-5.028401	-3.711431
H	9.247572	-2.052768	-1.582708
H	-0.026774	-0.998335	-4.900174
H	3.545939	1.615529	-2.720961
H	7.491875	4.268207	-0.853099
H	4.114943	-8.264426	-3.797024
H	8.127430	-5.623149	-1.882207
H	-1.534276	6.817841	-2.937224
H	2.266568	9.781126	-0.852877
H	-1.187238	-3.125449	-2.650082
H	2.667892	-0.470887	-0.668997
H	6.660258	2.265073	1.160082
H	-1.269490	2.289852	-3.161799
H	2.414201	4.741721	-0.680903
H	6.332304	7.292169	1.538081
H	3.107965	-5.389190	-1.435374
H	6.909927	-2.801541	0.551413
H	-1.059595	-2.192157	-0.032478
H	3.636268	-2.855832	2.428757
H	2.751551	2.939032	1.516377
H	-1.783381	0.074195	-1.455309
H	4.682759	-4.435974	1.688147
H	3.720658	1.496755	0.347946
Si	-1.529531	0.553778	1.657679
O	-0.615681	0.552976	0.308173
O	-0.674032	0.831854	3.051818
O	-2.213347	-0.974887	1.754795
O	-2.701819	1.731061	1.628856

H	-3.169232	-0.961365	1.943080
H	-3.188556	1.814017	0.798443
Si	0.706986	-2.936623	2.167769
O	1.595974	-3.225061	0.778131
O	0.765667	-1.331253	2.533552
O	1.531264	-3.625809	3.457263
O	-0.850577	-3.484575	2.178942
H	1.888934	-4.508585	3.313448
H	-1.494939	-2.753316	2.091299
Si	3.391524	-0.045806	3.861497
O	3.716561	0.094481	2.221384
O	1.861375	0.603011	4.040146
O	4.502015	0.821437	4.729940
O	3.423892	-1.544195	4.557018
H	4.828227	1.596348	4.261453
H	2.732720	-2.183949	4.314398
Si	0.449198	-0.203071	3.719279
C	-0.215261	-0.924380	5.296222
H	0.619037	-1.391762	5.850983
H	-0.514868	-0.028569	5.875515
C	-1.377688	-1.889574	5.099123
H	-2.121032	-1.451096	4.401747
H	-1.020259	-2.816056	4.617143
C	-2.092778	-2.268740	6.380004
H	-1.386322	-2.759064	7.084743
H	-2.891585	-3.001464	6.161108
N	-2.729564	-1.113687	7.019456
C	-3.413786	-1.443461	8.262765
H	-2.013230	-0.409633	7.215949
C	-4.117706	-0.232138	8.845684
H	-4.155640	-2.233649	8.036622
H	-2.718246	-1.867255	9.015955
H	-4.487853	-0.484102	9.857842
H	-3.384055	0.584054	8.974520
N	-5.165429	0.241226	7.947944
H	-5.994105	-0.353024	8.004866
H	-5.468122	1.186232	8.181200
Au	-6.499753	-3.528019	-0.601706
Au	-5.813142	-1.411690	1.095801
Au	-5.263737	0.614886	2.949108
Au	-5.172056	-4.085181	1.766660
Au	-6.904225	-0.906310	-1.445466
Au	-4.879922	-2.085531	3.694051
Au	-6.264475	1.212271	0.275001
Au	-4.348739	0.006031	5.523807

Au	-5.742262	3.309275	2.165954
Au	-4.888546	2.708984	4.720000
Au	-6.456775	3.874154	-0.395546

Au_{11}/fHNT, 3D cluster

314

O	4.292725	-8.641040	4.480031
O	6.930677	-7.839527	3.418241
O	-1.399132	-8.224511	2.275846
O	3.582790	-9.767917	0.471064
O	2.291715	-8.676991	2.927441
O	9.696309	6.201336	-0.657626
O	7.707499	-8.651312	-1.078094
O	5.607395	8.377994	-1.238350
O	9.564489	4.589995	-5.003506
O	6.699124	7.676035	-3.914290
O	-4.573348	-4.703056	5.723218
O	-4.389657	5.598076	5.404174
O	-4.561383	-4.948466	3.036091
O	-0.085989	-7.198317	6.107323
O	-2.491374	-7.052596	5.027307
O	-5.915879	0.841607	3.191067
O	-0.916630	7.757535	5.922859
O	-4.198127	4.685231	7.746512
O	-5.527246	1.362580	8.037961
O	-6.048781	0.371468	5.881206
O	-4.064974	-2.996466	7.572039
O	10.816703	-5.105742	-3.258757
O	11.431010	-0.601757	-4.600616
O	10.221049	-4.144269	1.469460
O	-5.085726	4.313304	3.061989
O	-1.917935	7.935934	1.209549
O	2.384598	9.332057	-1.351976
O	1.495689	9.814422	1.556658
O	3.772430	9.364789	2.596773
O	11.134276	1.276062	-0.212075
O	-1.307649	8.967246	3.614767
O	7.786208	8.218920	0.045023
H	-1.298517	6.885340	6.169052
H	-3.503037	5.062187	8.298836
H	10.894149	-3.593305	1.890128
H	7.608027	-7.437841	3.976350

H	-5.257950	0.662944	8.644193
H	10.295447	5.816915	-0.006510
H	4.447461	8.801991	2.133011
H	0.779343	-7.202809	5.629873
H	-4.883961	-3.384744	7.215409
H	11.588648	0.976878	0.585273
H	8.550670	7.708369	-0.286951
H	5.202781	-8.647158	4.129570
H	0.567312	10.050076	1.609631
H	-2.600771	-7.590768	4.234606
H	4.726616	8.762778	-1.183901
H	1.339741	-8.639468	2.820306
H	7.717868	-8.720732	-2.036451
H	-1.748736	-8.646841	1.485769
H	-4.825367	-4.834511	3.982910
H	-6.254962	1.476498	2.550277
H	3.991843	-10.201230	-0.284070
H	-5.062522	4.926327	3.838819
H	-1.841165	8.506563	2.019567
H	10.539663	-5.983178	-2.983841
H	11.724775	-1.331828	-5.164817
H	9.964746	3.813299	-5.404664
H	6.281314	8.334391	-3.331751
H	2.128261	10.032286	-0.730938
H	-0.777397	9.770274	3.629988
H	-4.348887	-5.644472	5.703414
H	-6.379262	0.504238	4.976531
H	-3.790477	6.262348	5.040489
H	-4.538713	5.445150	7.210600
H	-6.221061	0.938920	7.457626
H	-4.097013	-2.029571	7.388905
H	-0.561060	-8.001110	5.844927
H	3.823330	-9.394430	4.071324
H	7.378727	-8.077357	2.570100
H	10.651464	-4.451859	0.636359
H	11.638479	0.919799	-0.977338
H	9.868620	5.728070	-1.515127
H	7.521624	8.839613	-0.657153
H	3.571475	10.109182	2.008859
H	-1.667435	8.314972	5.655717
H	5.145354	-2.384160	-0.400381
H	-0.038374	0.074403	-0.702003
H	3.878255	2.719515	-0.575699
Al	3.288572	-7.253713	3.349468
O	4.800672	-6.060710	3.673069

Al	5.892184	-6.344235	2.173085
O	2.288589	-5.925583	2.385243
Al	1.335406	-5.096047	3.818183
O	-0.198854	-6.228626	3.560445
Si	-0.938544	-6.646810	2.147019
O	4.506980	-7.604958	1.920820
Si	3.925303	-8.171433	0.472639
O	2.292443	-6.506758	4.792494
Si	1.704956	-6.152791	0.851033
O	1.843420	-4.766835	0.016702
Si	2.161538	-3.137915	0.032076
O	3.400113	-2.985341	-1.064039
O	2.589700	-7.292949	0.093646
O	0.122718	-6.552910	0.904137
O	5.101192	-7.956002	-0.656316
Si	6.681382	-7.481284	-0.530380
O	6.840468	-6.199720	-1.535496
Si	7.118746	-4.597704	-1.588554
O	7.621416	-4.058073	-0.079190
Al	9.040735	-2.823902	0.156967
O	10.342322	-1.437283	0.524828
Al	9.543521	-0.100686	-0.632728
O	8.602149	0.699569	0.783233
Al	7.523039	1.985253	-0.130638
O	6.771486	3.355642	-1.187270
Al	7.969310	4.741812	-0.877282
O	7.040358	-7.166371	1.016680
O	7.187981	-4.990025	2.569872
Al	6.481295	-3.623921	1.391425
O	5.530430	-2.282357	0.484040
Al	4.405741	-1.578371	1.824257
O	3.701360	-0.179127	0.775596
Al	4.947272	1.178252	1.108148
O	3.945996	2.574006	0.379865
Al	3.105970	3.361470	1.832395
O	3.992673	1.857202	2.661163
O	5.224176	-5.018619	1.012273
O	5.378079	-2.949839	2.784113
O	7.983115	-2.409095	1.702248
O	5.730649	-0.211069	2.145566
Al	1.862563	-2.326639	3.139909
O	2.872227	-2.715669	1.501727
O	3.426321	-1.258939	3.401373
O	6.468917	2.352929	1.407993
O	5.980133	0.823539	-0.513176

Si	5.305501	0.691917	-2.034403
O	8.783040	3.407084	0.188399
O	8.583842	1.251003	-1.554892
Si	8.125707	1.110092	-3.152500
O	8.963092	-0.092142	-3.861260
Si	10.316090	-0.963933	-3.454440
O	10.730732	-0.478777	-1.957199
O	7.033734	6.162575	-1.672932
Al	6.169520	6.998946	-0.205566
O	9.067633	4.491931	-2.321758
Si	8.516148	4.146548	-3.811652
O	7.237991	5.611399	0.608616
Si	6.413214	6.218591	-3.216571
O	7.106934	4.995044	-4.054224
O	4.783628	5.926195	-3.071869
Si	3.766932	5.787386	-1.804657
O	4.599681	5.909780	-0.380788
Al	3.812086	6.139021	1.330485
O	5.356030	7.356640	1.486016
O	8.272489	2.516939	-3.969102
O	6.548772	0.651292	-3.138471
O	4.499378	2.113733	-2.243143
O	0.607582	-3.703384	2.750688
O	2.664534	-3.773156	4.098281
O	0.309283	-4.668501	5.328944
Al	-1.222255	-5.799296	5.157973
O	-2.186812	-4.372214	4.274968
Al	-3.060784	-3.694277	5.851699
O	0.856712	-0.961178	2.371535
Al	-0.033971	-0.168470	3.793357
O	-0.717537	1.357207	2.892969
Al	0.633488	2.640853	3.290240
O	2.179277	3.760714	3.475393
O	0.902537	-1.608993	4.662034
O	-1.675704	-1.139019	3.561615
Al	-2.553677	-0.864986	5.244637
O	-3.840052	-2.196623	4.917287
O	-0.988140	0.302649	5.469413
O	1.403702	1.097281	4.057118
Si	-2.511638	-1.540455	2.186427
O	-3.892644	-0.630813	2.225207
Si	-4.277209	0.694490	3.086092
O	-3.706372	2.058424	2.351816
Si	-3.623230	3.645455	2.804168
O	-2.822709	4.397527	1.577507

Si	-1.223698	4.286038	1.237926
O	-0.422477	4.115631	2.670856
Al	-1.071778	4.707381	4.383612
O	-1.817947	4.966268	6.172090
Al	-3.512324	4.031865	5.890908
O	-1.673501	-1.191270	0.825664
O	-2.818925	-3.124067	2.003713
Si	-3.001421	-4.545760	2.843248
O	-1.968445	-4.991883	6.702614
O	-2.219676	-5.659685	1.910187
O	-3.621296	0.579333	4.587047
Al	-4.479399	1.217793	6.198538
O	-2.977760	2.372272	6.666807
O	-3.452581	-0.283322	6.885866
O	-1.742072	-2.318216	6.102918
O	-0.090744	3.186364	4.946900
O	1.442266	2.400860	1.559104
Si	0.562904	2.151775	0.126303
O	-2.707466	3.760280	4.178089
O	-1.788114	6.348904	3.760786
Al	-0.264215	7.506073	3.982359
O	0.400327	5.853514	4.657882
O	-4.995805	2.931735	5.611519
O	5.799132	-3.760271	-2.081113
O	8.290181	-4.341849	-2.689409
Si	9.880136	-3.905513	-2.594873
O	10.194889	-3.622609	-1.014447
O	10.102379	-2.590197	-3.544110
O	8.305614	-1.497233	-0.936978
O	-0.735596	5.623241	0.445188
Si	-0.493764	7.209669	0.864293
O	-0.872661	2.975210	0.314142
O	0.348353	7.793764	-0.420647
Si	1.971210	7.901903	-0.684581
O	0.452437	7.270951	2.222478
Al	2.067708	8.303205	2.390006
O	2.495423	6.824352	-1.804530
O	2.759751	7.601241	0.733927
O	3.061923	4.306112	-1.946611
O	0.227187	0.499193	0.136117
O	2.400408	4.888108	0.943177
O	4.565388	4.589895	2.128162
O	3.118720	6.796164	2.935415
O	1.344488	8.484555	4.127073
H	7.217314	5.196874	1.472231

H	4.550435	2.056983	3.418371
H	1.465273	-1.447422	5.424089
H	-2.443754	-5.605914	7.270671
H	3.150619	6.370787	3.791815
H	-0.529911	2.401602	5.305382
H	-2.854493	-0.086490	7.614911
H	8.380660	0.144307	1.535070
H	5.704156	-2.681854	3.646370
H	2.795515	-6.208245	5.556499
H	1.796380	8.770591	4.921143
H	-1.200443	4.556090	6.790196
H	6.956270	2.189303	2.221358
H	3.458831	-0.371358	3.763998
H	0.617340	-4.370556	6.186006
H	1.154125	5.474717	4.177598
H	-2.185235	1.937300	6.319664
H	9.643547	3.086985	-0.114419
H	6.523270	-0.607305	1.753864
H	3.528253	-3.932247	3.687951
H	5.931883	7.201335	2.241844
H	2.647153	3.569686	4.294386
H	-0.464839	0.020817	6.229126
H	8.411139	-2.510469	2.557658
H	4.928873	-5.359039	4.312796
H	5.313021	4.178126	1.662168
H	2.131965	0.862891	3.460954
H	-0.897174	-2.585477	5.703529
H	11.090523	-1.735569	-0.015763
H	8.049585	-5.265311	2.228469
H	-2.419464	6.522917	3.051082
H	-5.833831	3.165880	5.206176
H	5.857569	3.387402	-1.502070
H	2.762003	-0.044753	0.612465
H	-0.314577	-3.488301	2.946253
H	1.543901	5.190778	1.278983
H	-1.654424	1.586160	2.864386
H	-4.784892	-2.047785	4.807281
H	7.352258	-1.347336	-0.871951
H	4.307496	-4.757356	1.177258
H	3.685490	2.192540	-2.764468
H	3.185992	-3.073220	-2.008670
H	-1.278024	-0.307809	0.751866
H	2.485647	4.129194	-1.185059
H	4.970430	-3.974103	-1.628239
H	0.612801	-0.888394	1.439083

Si	0.428652	2.469453	-2.901765
O	1.201416	2.408183	-1.479864
O	-0.472688	1.099981	-3.227202
O	1.599806	2.546617	-4.106826
O	-0.559570	3.788458	-3.011629
H	1.691797	3.435817	-4.471381
H	-1.454146	3.611679	-3.345296
Si	3.245856	-0.583555	-3.549720
O	4.287576	-0.675138	-2.247355
O	1.715799	-0.355122	-2.978710
O	3.147720	-2.111977	-4.242114
O	3.587247	0.523839	-4.721359
H	3.983842	-2.552241	-4.431812
H	2.966252	1.277632	-4.694371
Si	0.031621	-2.852098	-1.826983
O	0.795914	-2.235932	-0.477974
O	-0.643979	-1.539052	-2.621751
O	-1.162967	-3.936073	-1.447153
O	0.925418	-3.690191	-2.950153
H	-1.870108	-3.567658	-0.896274
H	1.593420	-3.198576	-3.462228
Si	0.156622	-0.416744	-3.554927
C	0.108454	-0.787409	-5.374594
H	0.870916	-1.566521	-5.573592
H	0.519433	0.130868	-5.833416
C	-1.240762	-1.161750	-6.016178
H	-2.096002	-0.817235	-5.398266
H	-1.342672	-0.639742	-6.981211
C	-1.393893	-2.649022	-6.287624
H	-0.544287	-2.988835	-6.923516
H	-2.316491	-2.822232	-6.872734
N	-1.460490	-3.430479	-5.058041
C	-1.217137	-4.859913	-5.198496
H	-0.798207	-3.060499	-4.378811
C	-2.495731	-5.664559	-5.342490
H	-0.678246	-5.196973	-4.294614
H	-0.556071	-5.076329	-6.062741
H	-3.070223	-5.315489	-6.219325
H	-2.233418	-6.724449	-5.524692
N	-3.333464	-5.484752	-4.156307
H	-4.205553	-6.008129	-4.227554
H	-2.849854	-5.819951	-3.319282
Au	-3.305279	-1.243893	-1.708368
Au	-4.662698	-0.515468	-4.203091
Au	-6.046244	-2.306531	-2.517063

Au	-4.422772	0.852945	-0.318714
Au	-5.801778	1.733386	-2.758592
Au	-7.264248	-0.151257	-1.049060
Au	-6.698102	2.490632	-0.134244
Au	-3.756903	-3.129123	-3.897321
Au	-7.589514	-0.171371	-3.912498
Au	-3.071925	1.532879	-3.034034
Au	-5.410566	-1.865208	0.198222

Au_{12}/fHNT, 2D cluster

315

O	-12.011699	2.781634	0.105751
O	-10.758870	5.162769	-1.114594
O	-10.304979	-0.990714	4.609717
O	-10.507496	4.446461	3.685057
O	-11.092178	1.878832	2.286418
O	2.901067	4.574473	-6.985461
O	-8.724507	8.164146	1.772027
O	5.030539	0.763399	-5.331567
O	4.213513	7.032783	-3.279431
O	6.060078	3.138571	-3.867360
O	-9.518534	-6.381657	2.446421
O	-1.078726	-9.565479	-2.541739
O	-8.106484	-5.039035	4.313101
O	-11.789228	-2.045435	0.849108
O	-11.003320	-3.560299	2.864317
O	-3.549057	-8.176740	2.129453
O	0.307678	-7.695341	-5.943903
O	-3.213965	-10.189670	-3.728191
O	-6.037183	-10.289349	-1.512103
O	-5.534890	-9.377800	0.683953
O	-9.263298	-7.402627	0.105158
O	-4.608454	10.523572	-0.345702
O	-0.203495	10.127642	-2.049632
O	-6.663459	7.511952	-3.561656
O	-0.698984	-8.611191	0.020702
O	3.281730	-6.382559	-2.382813
O	5.893005	-2.143002	-3.876058
O	4.546370	-4.386228	-5.500091
O	3.542147	-2.855305	-7.265132
O	-1.324018	7.208785	-5.668786
O	2.662056	-7.350648	-4.811796

O	4.114314	2.002825	-7.342527
H	-0.534695	-7.827427	-5.453865
H	-3.249247	-10.005323	-4.674273
H	-6.480341	7.680709	-4.495237
H	-10.777644	5.321544	-2.066472
H	-6.963006	-10.114483	-1.716513
H	2.197735	4.891763	-7.564550
H	3.362679	-1.897054	-7.072690
H	-11.515128	-1.113878	0.665164
H	-9.353075	-7.775531	1.000460
H	-2.045267	7.310800	-6.302120
H	3.896956	2.955288	-7.313258
H	-11.815344	3.691270	-0.185900
H	4.712126	-5.250076	-5.117330
H	-10.958756	-3.099154	3.710089
H	5.314408	-0.112613	-5.050841
H	-10.989030	1.137120	2.885062
H	-8.206458	8.641858	2.425319
H	-10.166939	-0.766475	5.534637
H	-8.579571	-5.733934	3.791135
H	-2.653738	-8.370748	2.428511
H	-10.406322	5.278854	4.156435
H	-0.673282	-9.160786	-0.802153
H	3.253110	-6.889419	-3.237158
H	-5.473201	10.465669	0.067918
H	-0.453220	10.877224	-1.489821
H	3.827828	7.809419	-2.864131
H	6.242553	2.303218	-4.332372
H	6.084459	-2.878313	-4.479696
H	3.291131	-7.195387	-5.523416
H	-10.262807	-5.870543	2.795515
H	-4.883830	-9.272277	1.398287
H	-0.334597	-9.130665	-2.977334
H	-2.281282	-10.475947	-3.558441
H	-6.022543	-10.443831	-0.525405
H	-8.378937	-7.670534	-0.234820
H	-12.270654	-2.040665	1.690078
H	-12.366014	2.842402	1.014459
H	-10.445808	6.004231	-0.701588
H	-6.415110	8.355577	-3.113845
H	-1.155956	8.097690	-5.283571
H	3.033763	5.274301	-6.291475
H	5.034314	1.903130	-7.039248
H	4.492210	-2.997910	-7.134076
H	0.921143	-8.373802	-5.613504

H	-4.086567	3.633977	-0.293946
H	-1.888204	-1.297995	1.668128
H	0.118009	0.953458	-1.973243
Al	-10.214252	2.016938	0.734733
O	-9.466691	2.700218	-0.935203
Al	-8.806036	4.386775	-0.444497
O	-8.563854	1.198290	1.282101
Al	-8.748351	-0.528495	0.485757
O	-9.486746	-1.280970	2.095625
Si	-8.968545	-1.087339	3.649254
O	-9.651739	3.797028	1.139436
Si	-9.232983	4.186234	2.698158
O	-10.470539	0.278239	-0.002680
Si	-7.821846	1.511244	2.730393
O	-6.213633	1.543997	2.509966
Si	-4.922171	1.248995	1.509614
O	-4.154297	2.717965	1.439325
O	-8.289928	2.972810	3.279062
O	-8.159226	0.327293	3.803178
O	-8.395614	5.600559	2.651829
Si	-8.105647	6.674324	1.426609
O	-6.478955	6.838730	1.357258
Si	-5.166698	6.549489	0.440185
O	-5.642830	6.076262	-1.099752
Al	-4.809074	6.710390	-2.679670
O	-3.930953	7.135400	-4.353117
Al	-2.159910	6.568414	-3.799533
O	-2.357818	4.869088	-4.577124
Al	-0.770772	3.976582	-3.996137
O	0.966442	3.389948	-3.552214
Al	1.879826	3.757136	-5.128040
O	-8.782987	6.141968	0.055751
O	-7.971127	4.804548	-2.117463
Al	-6.164816	4.312669	-1.617800
O	-4.538148	3.503243	-1.142378
Al	-4.766018	1.721641	-1.717609
O	-3.010931	1.160150	-1.320266
Al	-2.134599	1.565909	-2.924217
O	-0.571247	0.613611	-2.563412
Al	-0.802217	-1.015522	-3.416812
O	-2.511559	-0.166984	-3.723651
O	-7.043068	3.932030	0.041443
O	-6.448320	2.534409	-2.224816
O	-5.392021	4.986109	-3.284281
O	-3.875768	2.193978	-3.364552

Al	-6.129133	-0.717962	-0.743607
O	-5.469528	1.001694	-0.063579
O	-5.445196	0.078836	-2.340798
O	-1.387555	2.274244	-4.575113
O	-1.457731	3.285055	-2.285544
Si	-0.646170	3.481148	-0.838740
O	0.165145	4.378432	-5.631225
O	-0.516211	5.755234	-3.314405
Si	0.331792	6.171104	-1.939585
O	-0.214756	7.592339	-1.364712
Si	-1.169275	8.804791	-1.977068
O	-1.680969	8.283598	-3.431570
O	3.502899	2.881460	-4.773423
Al	3.302426	1.209135	-5.646742
O	2.534138	5.412071	-4.694081
Si	3.154655	5.770637	-3.234767
O	1.693614	2.173688	-6.106274
Si	4.477745	3.072839	-3.437732
O	3.992717	4.443674	-2.685691
O	4.172241	1.771148	-2.450694
Si	3.311973	0.396856	-2.623911
O	2.549736	0.374500	-4.091675
Al	1.716249	-1.143329	-4.868065
O	2.583844	-0.364148	-6.458888
O	1.946005	6.195429	-2.186695
O	-0.029449	5.029595	-0.815347
O	0.625237	2.442014	-0.943606
O	-6.986987	-1.098249	0.912102
O	-7.868992	-0.018578	-1.114805
O	-9.300732	-2.215255	-0.120745
Al	-10.089661	-3.006462	1.430097
O	-8.408963	-3.867342	1.855228
Al	-8.801708	-5.544955	0.994495
O	-4.566015	-1.645121	-0.339035
Al	-4.773947	-3.303162	-1.145850
O	-3.006502	-3.959404	-0.919657
Al	-2.229047	-3.472774	-2.589770
O	-1.457383	-2.672849	-4.152704
O	-6.456588	-2.454004	-1.538879
O	-5.397120	-4.210425	0.428776
Al	-6.176972	-5.804210	-0.299366
O	-7.035456	-6.254204	1.311744
O	-5.391062	-5.022663	-1.923042
O	-3.931597	-2.677525	-2.769934
Si	-4.887496	-4.118984	2.004529

O	-4.169152	-5.574412	2.323703
Si	-3.619454	-6.737510	1.328480
O	-2.092809	-6.390053	0.805084
Si	-1.093291	-7.068956	-0.321970
O	0.235935	-6.097709	-0.353962
Si	0.334807	-4.593894	-0.996750
O	-0.666983	-4.547217	-2.307630
Al	-1.212323	-6.075157	-3.343640
O	-2.068684	-7.605035	-4.208838
Al	-2.632808	-8.544596	-2.589557
O	-3.801041	-2.918389	2.237451
O	-6.043579	-3.750038	3.082933
Si	-7.683155	-3.809204	3.342968
O	-10.360704	-4.608371	0.452840
O	-8.024086	-2.359047	4.052445
O	-4.616445	-6.860083	0.029508
Al	-5.061775	-8.527542	-0.843507
O	-4.431572	-7.907084	-2.582727
O	-6.685214	-7.499672	-1.140248
O	-7.862378	-5.048121	-0.607521
O	-2.777020	-5.020315	-3.523334
O	-1.392257	-1.924800	-1.807397
Si	-0.725985	-1.891075	-0.243727
O	-1.832468	-6.997606	-1.801162
O	0.482024	-6.926477	-3.336845
Al	1.261875	-6.173966	-4.929326
O	-0.472395	-5.385803	-4.935242
O	-3.332727	-9.256425	-1.010803
O	-4.188675	5.415846	1.106230
O	-4.313726	7.933438	0.358120
Si	-4.035796	9.042769	-0.833277
O	-4.758340	8.469538	-2.184646
O	-2.416120	9.221447	-0.992054
O	-3.084661	6.169272	-2.199271
O	1.875902	-4.277854	-1.421255
Si	2.893349	-4.811258	-2.617583
O	-0.165149	-3.433801	0.051518
O	4.122520	-3.722083	-2.552476
Si	4.351876	-2.308148	-3.366839
O	2.120423	-4.689584	-4.077540
Al	2.831703	-3.794953	-5.625578
O	4.154393	-0.994055	-2.405403
O	3.254427	-2.221156	-4.596624
O	2.217182	0.386849	-1.393991
O	-2.052065	-1.614024	0.758981

O	0.959489	-1.695251	-3.185704
O	-0.009154	-0.374338	-5.055792
O	1.288251	-2.679276	-5.840756
O	1.943808	-5.255929	-6.432139
H	0.844771	1.895211	-6.452699
H	-2.810122	-0.130385	-4.636829
H	-6.788673	-2.402686	-2.439198
H	-11.188124	-5.053885	0.659119
H	0.434546	-2.907709	-6.207344
H	-3.616040	-5.281070	-3.116790
H	-6.969644	-7.415994	-2.056795
H	-3.250565	4.525888	-4.665664
H	-6.752960	2.309533	-3.106996
H	-10.693569	0.233548	-0.937646
H	1.693264	-5.352258	-7.351114
H	-2.773062	-7.248653	-4.764227
H	-2.010133	2.349962	-5.304848
H	-4.951711	-0.363291	-3.034584
H	-9.578093	-2.462589	-1.003983
H	-0.495334	-4.417634	-4.868247
H	-4.579580	-6.950167	-2.597058
H	0.081965	5.331515	-5.770310
H	-3.966087	3.158602	-3.369025
H	-7.758876	0.932709	-1.265191
H	2.001549	-0.192072	-7.206088
H	-2.105095	-2.606414	-4.861375
H	-6.076535	-4.852618	-2.580043
H	-5.989352	4.972684	-4.038080
H	-9.288890	2.270332	-1.772830
H	-0.066982	0.593283	-4.978271
H	-3.769260	-1.725167	-2.684047
H	-7.845272	-4.080744	-0.700764
H	-3.853255	8.099818	-4.283356
H	-7.995323	5.761328	-2.254156
H	1.052227	-7.171735	-2.597051
H	-2.894640	-9.832674	-0.380382
H	1.188930	2.777917	-2.837183
H	-2.796815	0.422061	-0.740232
H	-6.923140	-2.016388	1.208460
H	1.008921	-2.654508	-3.061831
H	-2.797219	-4.790013	-0.475518
H	-6.841504	-7.026363	1.853150
H	-2.994569	5.308372	-1.767632
H	-6.923967	3.016982	0.331777
H	1.008140	1.991939	-0.174720

H	-3.657214	3.011983	2.221856
H	-3.052975	-2.859169	1.621614
H	1.624973	-0.379033	-1.471855
H	-4.623272	4.599496	1.393032
H	-3.947191	-1.435575	0.373288
Si	1.325128	-0.669047	1.666895
O	0.425802	-0.700057	0.309002
O	0.446118	-0.844459	3.063466
O	2.064803	0.833150	1.683806
O	2.449960	-1.892787	1.717866
H	2.928558	0.810331	2.137789
H	2.997292	-1.980091	0.926910
Si	-0.725483	2.966709	1.978382
O	-1.596051	3.178222	0.562517
O	-0.860842	1.408536	2.498113
O	-1.525503	3.818893	3.184002
O	0.853707	3.456991	1.941369
H	-1.882698	4.676165	2.927388
H	1.472109	2.702940	1.868884
Si	-3.555729	0.276973	3.802919
O	-3.877467	0.065103	2.169847
O	-2.062943	-0.438222	4.028751
O	-4.716592	-0.480166	4.707211
O	-3.514546	1.811161	4.417781
H	-5.072051	-1.268937	4.285411
H	-2.788991	2.398959	4.150162
Si	-0.605429	0.280811	3.702920
C	0.112122	1.013002	5.262722
H	-0.707005	1.165090	5.988517
H	0.755456	0.206497	5.667434
C	0.891917	2.302645	5.029569
H	1.580550	2.183700	4.169720
H	0.193324	3.108636	4.746705
C	1.706299	2.785013	6.211160
H	1.050985	2.963977	7.091103
H	2.176608	3.754637	5.960013
N	2.789420	1.861386	6.556965
C	3.628939	2.345295	7.648679
H	2.380615	0.964209	6.833713
C	4.795749	1.413305	7.916721
H	4.011640	3.342437	7.356455
H	3.042590	2.490494	8.578998
H	5.281355	1.712082	8.865966
H	4.411427	0.387781	8.071230
N	5.705342	1.384951	6.779330

```
 H     6.289835     2.221340     6.751664
 H     6.333030     0.582352     6.816312
Au     3.961015     3.771582     3.347962
Au     5.305938     1.685238     2.052624
Au     6.279947    -0.402747     0.480504
Au     5.200613     4.256916     0.930504
Au     4.205802     1.206385     4.644407
Au     6.392307     2.247744    -0.548633
Au     5.108403    -0.915602     2.953422
Au     7.280907     0.082240    -2.099116
Au     6.172119    -3.101453     1.397714
Au     7.204788    -2.499599    -1.140888
Au     4.959133    -3.568297     3.842636
Au     3.671262    -1.582592     5.258009
```

Au_{12}/fHNT, 3D cluster

```
  315
 O     5.979257    -8.701319     3.132121
 O     8.405337    -7.318076     2.168074
 O     0.214911    -8.932175     1.095415
 O     5.308763    -9.328681    -0.991291
 O     3.952448    -8.845618     1.619809
 O     8.655792     7.433275     0.233601
 O     9.129066    -7.315368    -2.409664
 O     4.244338     8.949316     0.046525
 O     8.621784     6.481096    -4.304314
 O     5.331454     8.843997    -2.719763
 O    -3.359443    -6.544624     5.080921
 O    -4.889911     3.572366     6.327132
 O    -3.412204    -6.385784     2.387780
 O     1.489554    -8.274871     5.019809
 O    -0.947273    -8.380077     4.007704
 O    -5.696035    -0.994753     3.438745
 O    -1.803040     6.187169     7.119753
 O    -4.459038     2.369939     8.500846
 O    -5.209507    -1.135635     8.302722
 O    -5.644324    -1.871805     6.027885
 O    -3.067641    -5.068033     7.160238
 O    11.523251    -3.013415    -4.069361
 O    11.333349     1.677453    -4.720146
 O    10.962443    -2.873810     0.757779
 O    -5.455550     2.547849     3.826852
```

O	-3.003506	6.886429	2.502432
O	0.906377	9.351371	0.123516
O	0.064459	9.242660	3.083642
O	2.423069	9.035836	4.011941
O	10.902721	2.810639	-0.093700
O	-2.478141	7.640156	5.027708
O	6.468022	8.973263	1.260706
H	-2.025922	5.236377	7.235904
H	-3.814593	2.773340	9.094365
H	11.551371	-2.285145	1.247905
H	9.028447	-6.894424	2.771310
H	-4.805018	-1.861304	8.791926
H	9.335114	7.063908	0.810478
H	3.163031	8.669745	3.458843
H	2.324442	-8.062699	4.535329
H	-3.824675	-5.532235	6.760114
H	11.431029	2.478333	0.642637
H	7.292655	8.653778	0.844555
H	6.863455	-8.501889	2.772282
H	-0.887282	9.307667	3.184574
H	-0.997307	-8.806219	3.144065
H	3.314968	9.167481	0.170903
H	3.003873	-8.954091	1.532747
H	9.113277	-7.240042	-3.367501
H	-0.090858	-9.286391	0.255270
H	-3.654156	-6.458853	3.344472
H	-6.159975	-0.338879	2.906518
H	5.753851	-9.570579	-1.809028
H	-5.503427	3.034714	4.687358
H	-2.990303	7.336137	3.388563
H	11.405686	-3.955835	-3.927063
H	11.721227	1.098737	-5.392650
H	9.128674	5.850906	-4.824228
H	4.833873	9.329166	-2.038314
H	0.562529	9.898954	0.847146
H	-2.087415	8.510083	5.157367
H	-2.983731	-7.421240	4.915316
H	-6.027282	-1.664931	5.158500
H	-4.423179	4.374551	6.060297
H	-4.940981	3.131950	8.091276
H	-5.846089	-1.580207	7.674409
H	-3.265850	-4.104174	7.126494
H	1.143510	-9.098823	4.645138
H	5.624942	-9.454606	2.620274
H	8.852980	-7.349173	1.287560

H	11.404799	-2.978086	-0.118220
H	11.428477	2.661396	-0.911061
H	8.870152	7.127529	-0.688108
H	6.077447	9.636933	0.664628
H	2.079265	9.813969	3.546694
H	-2.645364	6.642941	6.950722
H	5.596805	-1.740457	-0.752986
H	0.070783	-0.175781	-0.606323
H	3.499408	3.045085	-0.133877
Al	4.716625	-7.352345	2.239261
O	6.022723	-5.981935	2.719371
Al	7.086575	-5.852799	1.178826
O	3.474368	-6.084868	1.501748
Al	2.454164	-5.648641	3.057029
O	1.118836	-6.973614	2.651492
Si	0.403579	-7.297833	1.201293
O	5.919494	-7.278279	0.757186
Si	5.383063	-7.715198	-0.752057
O	3.668114	-7.005339	3.792563
Si	2.876679	-6.178808	-0.040976
O	2.751930	-4.681750	-0.657028
Si	2.797470	-3.042029	-0.398173
O	3.950040	-2.523393	-1.476114
O	3.907079	-7.028865	-0.974824
O	1.385416	-6.843876	-0.027433
O	6.462278	-7.140227	-1.851315
Si	7.946225	-6.429188	-1.676557
O	7.852347	-5.005250	-2.477532
Si	7.860293	-3.389178	-2.290566
O	8.325680	-3.000784	-0.723842
Al	9.530132	-1.593042	-0.322623
O	10.598558	-0.076031	0.233530
Al	9.545618	1.262174	-0.696541
O	8.541221	1.674362	0.837351
Al	7.229936	2.879698	0.144253
O	6.221948	4.243932	-0.681600
Al	7.185926	5.751504	-0.181283
O	8.308535	-6.289623	-0.104717
O	8.155772	-4.372613	1.758726
Al	7.187930	-2.987010	0.811217
O	5.994087	-1.706521	0.131243
Al	4.821958	-1.407973	1.578186
O	3.855982	-0.008796	0.763947
Al	4.872981	1.475499	1.281531
O	3.627516	2.773908	0.787488

Al	2.726537	3.185364	2.354180
O	3.880912	1.747055	2.932639
O	6.164220	-4.502718	0.242066
O	6.043934	-2.721697	2.305181
O	8.479799	-1.595187	1.282156
O	5.914721	0.101025	2.085078
Al	2.490405	-2.760704	2.799905
O	3.485583	-2.727829	1.107903
O	3.865656	-1.494533	3.198870
O	6.190579	2.833181	1.735249
O	5.886047	1.543241	-0.388955
Si	5.185570	1.526003	-1.902729
O	8.249677	4.431182	0.658064
O	8.340841	2.553220	-1.389464
Si	7.850130	2.573929	-2.983475
O	8.845988	1.648208	-3.878034
Si	10.339099	0.967235	-3.626988
O	10.726243	1.289455	-2.079303
O	5.998454	7.095296	-0.739015
Al	5.066152	7.548140	0.850101
O	8.253111	5.906399	-1.661955
Si	7.708338	5.696192	-3.179270
O	6.379842	6.256442	1.429358
Si	5.317339	7.272528	-2.247549
O	6.170087	6.320761	-3.270749
O	3.765217	6.690943	-2.126468
Si	2.835658	6.197165	-0.881130
O	3.691822	6.247306	0.533197
Al	2.944700	6.085475	2.270131
O	4.271554	7.510022	2.587433
O	7.730831	4.090100	-3.578946
O	6.372267	1.858337	-3.017539
O	4.146286	2.805356	-1.882362
O	1.465446	-4.257075	2.223499
O	3.556814	-4.176152	3.516388
O	1.431424	-5.627964	4.629191
Al	0.101874	-6.963524	4.309640
O	-1.118615	-5.605597	3.666934
Al	-2.030130	-5.324917	5.340215
O	1.243867	-1.486704	2.261706
Al	0.290900	-1.074171	3.799545
O	-0.669608	0.429872	3.150755
Al	0.465672	1.850554	3.719657
O	1.811623	3.175772	4.051367
O	1.485401	-2.447819	4.426435

O	-1.176057	-2.263226	3.445782
Al	-2.020741	-2.392509	5.162840
O	-3.081662	-3.859337	4.654863
O	-0.661658	-1.023232	5.540692
O	1.509310	0.363379	4.232586
Si	-1.987435	-2.593019	2.037242
O	-3.496974	-1.945555	2.232722
Si	-4.060916	-0.845815	3.290069
O	-3.752002	0.688046	2.763598
Si	-3.914141	2.182070	3.450481
O	-3.297194	3.231006	2.341313
Si	-1.716205	3.442539	1.966729
O	-0.842464	3.200666	3.345990
Al	-1.513027	3.415262	5.137524
O	-2.221193	3.277946	6.954690
Al	-3.747924	2.122511	6.558278
O	-1.272206	-1.910599	0.733850
O	-2.036222	-4.161313	1.620455
Si	-1.948774	-5.701366	2.236830
O	-0.706315	-6.530347	5.969103
O	-1.031084	-6.517371	1.134768
O	-3.336833	-1.068415	4.746950
Al	-4.224838	-0.828648	6.448373
O	-2.917041	0.481132	7.065755
O	-2.938426	-2.219370	6.885590
O	-0.947643	-3.798115	5.779250
O	-0.273305	2.015691	5.449760
O	1.234663	2.008397	1.961161
Si	0.352616	1.828048	0.517890
O	-2.976866	2.246219	4.813150
O	-2.514088	4.985765	4.781093
Al	-1.194438	6.338285	5.154763
O	-0.240543	4.740508	5.562315
O	-5.039407	0.840931	6.135569
O	6.402400	-2.723414	-2.632024
O	8.929549	-2.779705	-3.355854
Si	10.428224	-2.099793	-3.218091
O	10.753535	-2.003810	-1.617525
O	10.393205	-0.640528	-3.959586
O	8.544074	-0.263127	-1.192212
O	-1.486706	4.944986	1.379562
Si	-1.493650	6.470131	2.031307
O	-1.190167	2.360482	0.849891
O	-0.810281	7.370980	0.838464
Si	0.760939	7.789430	0.571663

O	-0.518164	6.489561	3.369876
Al	0.910027	7.743742	3.669908
O	1.411520	6.992906	-0.706029
O	1.643101	7.420604	1.917068
O	2.379618	4.655406	-1.236613
O	0.295220	0.159540	0.282836
O	1.744633	4.684982	1.716881
O	3.973787	4.585494	2.812889
O	2.215711	6.372245	3.965634
O	0.235256	7.542315	5.424174
H	6.461589	5.721709	2.220225
H	4.427249	1.924397	3.703639
H	2.043132	-2.307600	5.196356
H	-1.051268	-7.292760	6.443841
H	2.350782	5.836884	4.746997
H	-0.562662	1.123881	5.690955
H	-2.352961	-2.033939	7.627753
H	8.443907	0.985021	1.499144
H	6.354794	-2.532509	3.193553
H	4.144532	-6.742019	4.586026
H	0.664468	7.780398	6.246161
H	-1.520812	2.891444	7.494778
H	6.729659	2.636193	2.507544
H	3.765473	-0.677486	3.691571
H	1.719381	-5.411857	5.517225
H	0.546053	4.569477	5.019739
H	-2.077810	0.242278	6.645701
H	9.138693	4.309228	0.298328
H	6.745915	-0.093493	1.626863
H	4.418238	-4.124807	3.074793
H	4.894232	7.344575	3.302906
H	2.336249	2.947961	4.825332
H	-0.069751	-1.321436	6.241518
H	8.951784	-1.747725	2.106273
H	6.058343	-5.370736	3.456390
H	4.760304	4.379185	2.279515
H	2.242391	0.345841	3.597763
H	-0.086500	-3.856979	5.332303
H	11.364001	-0.160659	-0.356346
H	9.036968	-4.445021	1.367638
H	-3.192743	5.153271	4.114810
H	-5.919769	0.987270	5.782069
H	5.303712	4.166874	-0.975299
H	2.901677	-0.012500	0.636075
H	0.528803	-4.232079	2.462149

H	0.863724	4.785762	2.106525
H	-1.631818	0.498930	3.170184
H	-4.041677	-3.857701	4.581767
H	7.582333	-0.287691	-1.092043
H	5.224162	-4.427284	0.457429
H	3.310804	2.821502	-2.374425
H	3.716579	-2.505984	-2.420084
H	-1.031052	-0.971936	0.789602
H	1.870628	4.273409	-0.502881
H	5.638660	-3.138543	-2.205487
H	0.955033	-1.319573	1.354567
Si	0.061443	2.576299	-2.434186
O	0.877583	2.417401	-1.040104
O	-0.577688	1.159647	-3.024723
O	1.173076	3.038178	-3.616000
O	-1.117026	3.724128	-2.283491
H	1.223385	3.995421	-3.728907
H	-1.928679	3.548566	-2.794742
Si	3.332600	0.146825	-3.578999
O	4.409652	0.049710	-2.307303
O	1.814266	-0.015416	-2.959312
O	3.483195	-1.252343	-4.497783
O	3.418161	1.463241	-4.565520
H	4.373134	-1.493521	-4.777764
H	2.683923	2.089082	-4.404507
Si	0.583324	-2.807490	-2.165684
O	1.281668	-2.315605	-0.735927
O	-0.328255	-1.519585	-2.736413
O	-0.435801	-4.098613	-1.907576
O	1.526744	-3.330237	-3.423917
H	-0.953207	-4.014532	-1.095956
H	2.103374	-2.681190	-3.868250
Si	0.265452	-0.167453	-3.542403
C	0.237099	-0.304669	-5.396702
H	1.060458	-0.976737	-5.709437
H	0.567658	0.704032	-5.709013
C	-1.080005	-0.675508	-6.100858
H	-1.958134	-0.400522	-5.480849
H	-1.175184	-0.089671	-7.029604
C	-1.171826	-2.143334	-6.479718
H	-0.331563	-2.391000	-7.167473
H	-2.107987	-2.327233	-7.038594
N	-1.149030	-3.009804	-5.306564
C	-0.891092	-4.427502	-5.553950
H	-0.440760	-2.670628	-4.658903

C	-2.109391	-5.297713	-5.306884
H	-0.080514	-4.753659	-4.875973
H	-0.526692	-4.590672	-6.585963
H	-2.917578	-5.037363	-6.014401
H	-1.835131	-6.354146	-5.491982
N	-2.598980	-5.066802	-3.948446
H	-3.361296	-5.697817	-3.705374
H	-1.845788	-5.194020	-3.263527
Au	-4.283220	-0.232543	-3.949833
Au	-3.611694	1.699900	-1.467272
Au	-6.142290	1.819138	-2.855997
Au	-5.369796	-2.158484	-2.196845
Au	-4.665014	-0.297550	0.125777
Au	-7.271866	-0.123888	-1.137783
Au	-6.567406	-2.404960	0.339559
Au	-3.781014	2.544998	-4.220855
Au	-6.192940	2.293405	-0.038835
Au	-2.544389	-0.888795	-1.514380
Au	-7.407297	-0.418767	-3.922978
Au	-3.259698	-2.770927	-3.848330

Au_{13}/fHNT, 2D cluster

316

O	-12.451888	1.382943	0.342175
O	-11.559091	3.939242	-0.839704
O	-10.146856	-2.231643	4.706896
O	-11.097890	3.149351	3.932742
O	-11.367221	0.560402	2.478243
O	1.910400	5.332818	-7.007254
O	-9.875594	7.117441	2.083371
O	4.570383	1.804470	-5.500789
O	2.971229	7.856484	-3.264763
O	5.307790	4.261025	-3.995178
O	-8.698308	-7.414914	2.383959
O	-0.031518	-9.315466	-2.862322
O	-7.433947	-5.939370	4.256170
O	-11.567166	-3.386668	0.951067
O	-10.536926	-4.829571	2.908149
O	-2.551676	-8.382955	1.894819
O	1.009188	-7.195545	-6.241245
O	-2.092060	-10.192723	-4.020387
O	-4.821999	-10.723776	-1.749494

O	-4.393237	-9.805429	0.459281
O	-8.365452	-8.336343	0.011362
O	-6.165029	10.058578	-0.055548
O	-1.789296	10.299180	-1.860782
O	-7.875577	6.875290	-3.307617
O	0.278984	-8.379969	-0.283678
O	3.866061	-5.579804	-2.708907
O	5.849231	-0.993335	-4.141982
O	4.776138	-3.358018	-5.797191
O	3.533523	-1.934511	-7.499275
O	-2.596240	7.343027	-5.532781
O	3.322938	-6.564409	-5.149547
O	3.448020	2.956890	-7.458296
H	0.204205	-7.451313	-5.737500
H	-2.174612	-9.992356	-4.960249
H	-7.739371	7.089282	-4.239946
H	-11.622002	4.116637	-1.786393
H	-5.767575	-10.670181	-1.929830
H	1.157077	5.566331	-7.562859
H	3.232081	-1.013943	-7.277520
H	-11.424801	-2.422607	0.786497
H	-8.382780	-8.739109	0.898020
H	-3.339780	7.362159	-6.148005
H	3.105863	3.870508	-7.398988
H	-12.386217	2.317393	0.070987
H	5.065288	-4.200585	-5.441257
H	-10.534035	-4.386858	3.764868
H	4.975704	0.968161	-5.249616
H	-11.151222	-0.174724	3.054512
H	-9.410503	7.644808	2.738312
H	-10.017722	-2.012931	5.634421
H	-7.822233	-6.678966	3.725721
H	-1.631464	-8.461865	2.169870
H	-11.097692	3.976346	4.424057
H	0.358104	-8.901399	-1.121327
H	3.884879	-6.065456	-3.575753
H	-7.003962	9.875106	0.374279
H	-2.123503	10.994853	-1.276003
H	2.495202	8.564111	-2.820856
H	5.589154	3.469088	-4.486114
H	6.122737	-1.681664	-4.768969
H	3.908137	-6.309096	-5.869687
H	-9.495639	-7.016965	2.762042
H	-3.745059	-9.630330	1.162483
H	0.636921	-8.774309	-3.301496

H	-1.125609	-10.354940	-3.877820
H	-4.762911	-10.898299	-0.767810
H	-7.461679	-8.474742	-0.354006
H	-12.024384	-3.466670	1.801700
H	-12.789034	1.373896	1.259359
H	-11.351601	4.805104	-0.410899
H	-7.731648	7.733761	-2.842616
H	-2.539430	8.237047	-5.127456
H	1.964946	6.027439	-6.297661
H	4.380158	2.974628	-7.177011
H	4.497018	-1.951144	-7.391890
H	1.715792	-7.793015	-5.941992
H	-4.724267	3.302463	-0.199378
H	-1.838373	-1.334289	1.583565
H	-0.240565	1.252346	-2.037069
Al	-10.553535	0.852152	0.912884
O	-9.944861	1.669251	-0.753331
Al	-9.504285	3.417181	-0.231443
O	-8.795619	0.250095	1.403641
Al	-8.766481	-1.466404	0.565304
O	-9.358263	-2.349391	2.169441
Si	-8.833177	-2.124827	3.716583
O	-10.224795	2.681586	1.353368
Si	-9.824291	3.086401	2.912911
O	-10.592499	-0.887193	0.134619
Si	-8.067327	0.625417	2.844049
O	-6.483751	0.879445	2.591135
Si	-5.188933	0.784416	1.556794
O	-4.626812	2.344811	1.509756
O	-8.713468	1.997344	3.441321
O	-8.217067	-0.618277	3.891500
O	-9.185202	4.601177	2.887097
Si	-9.071378	5.733001	1.685333
O	-7.483508	6.116251	1.586577
Si	-6.166940	6.027973	0.634968
O	-6.612581	5.531763	-0.906859
Al	-5.909752	6.309621	-2.486219
O	-5.137237	6.888509	-4.165583
Al	-3.293340	6.551820	-3.664375
O	-3.280713	4.860282	-4.482956
Al	-1.574873	4.175764	-3.959247
O	0.235502	3.817632	-3.567472
Al	1.053058	4.341659	-5.151556
O	-9.704343	5.147140	0.315090
O	-8.773599	3.983077	-1.909621

Al	-6.906122	3.726818	-1.461021
O	-5.174682	3.132400	-1.041424
Al	-5.175906	1.350591	-1.659340
O	-3.352268	1.020839	-1.313815
Al	-2.577238	1.578765	-2.924214
O	-0.892197	0.837029	-2.621659
Al	-0.923583	-0.787590	-3.513424
O	-2.738055	-0.169572	-3.761776
O	-7.685056	3.192203	0.205337
O	-6.963597	1.941525	-2.109458
O	-6.271052	4.537478	-3.124574
O	-4.396998	1.977294	-3.311212
Al	-6.176024	-1.272679	-0.722938
O	-5.736231	0.503247	-0.010928
O	-5.643834	-0.353405	-2.312083
O	-1.971986	2.420164	-4.570699
O	-2.121419	3.357601	-2.253864
Si	-1.308632	3.627964	-0.819546
O	-0.741052	4.738535	-5.602109
O	-1.544284	5.955739	-3.235538
Si	-0.726629	6.449080	-1.867998
O	-1.444400	7.769829	-1.243989
Si	-2.567214	8.857131	-1.803624
O	-3.039594	8.306591	-3.260612
O	2.786874	3.684008	-4.854394
Al	2.791027	2.021151	-5.767869
O	1.490211	6.058762	-4.687096
Si	2.092293	6.462718	-3.231925
O	1.056900	2.771263	-6.167797
Si	3.759381	3.972926	-3.534629
O	3.113532	5.247822	-2.736178
O	3.654914	2.618884	-2.576698
Si	2.982460	1.145905	-2.768754
O	2.194755	1.056155	-4.220408
Al	1.553441	-0.541082	-5.019700
O	2.270110	0.385286	-6.606770
O	0.863393	6.696097	-2.147911
O	-0.904292	5.242927	-0.767501
O	0.087565	2.770678	-0.978696
O	-6.934889	-1.804113	0.939474
O	-8.002341	-0.804971	-1.038967
O	-9.102567	-3.197224	-0.074569
Al	-9.740658	-4.123971	1.470588
O	-7.950027	-4.760897	1.837418
Al	-8.135346	-6.455145	0.940381

O	-4.493510	-1.990583	-0.375984
Al	-4.497050	-3.641872	-1.222458
O	-2.652706	-4.059678	-1.050770
Al	-1.987978	-3.433384	-2.723103
O	-1.368558	-2.500018	-4.279703
O	-6.287243	-3.017583	-1.557482
O	-4.954808	-4.661865	0.339910
Al	-5.531646	-6.328276	-0.414309
O	-6.282872	-6.927791	1.201676
O	-4.897017	-5.409773	-2.032453
O	-3.785639	-2.870223	-2.846359
Si	-4.424002	-4.540212	1.906601
O	-3.509735	-5.893003	2.171625
Si	-2.833523	-6.947631	1.134355
O	-1.380276	-6.385705	0.588803
Si	-0.326451	-6.897055	-0.576602
O	0.859582	-5.755412	-0.610206
Si	0.740714	-4.237042	-1.214374
O	-0.289860	-4.294427	-2.502374
Al	-0.650629	-5.856743	-3.567405
O	-1.315139	-7.466871	-4.455304
Al	-1.708994	-8.511980	-2.850403
O	-3.502761	-3.210310	2.148964
O	-5.592612	-4.355813	3.018334
Si	-7.202719	-4.641073	3.310755
O	-9.818364	-5.724090	0.456591
O	-7.717462	-3.267209	4.065794
O	-3.836292	-7.172238	-0.146407
Al	-4.075364	-8.863207	-1.054351
O	-3.576223	-8.122475	-2.789052
O	-5.828517	-8.056208	-1.289581
O	-7.310077	-5.798573	-0.666941
O	-2.346355	-5.017910	-3.686157
O	-1.347481	-1.805972	-1.917006
Si	-0.654255	-1.721902	-0.367404
O	-1.104134	-6.890604	-2.037646
O	1.142113	-6.472414	-3.618706
Al	1.775382	-5.584232	-5.206325
O	-0.048431	-5.036511	-5.155017
O	-2.268855	-9.348787	-1.277075
O	-5.030093	5.020627	1.249884
O	-5.509191	7.515605	0.572300
Si	-5.411235	8.680239	-0.594456
O	-6.083039	8.047345	-1.945377
O	-3.834392	9.078810	-0.782059

O	-4.117289	5.994026	-2.056490
O	2.214786	-3.706703	-1.662231
Si	3.265218	-4.069866	-2.893336
O	0.115262	-3.180074	-0.125115
O	4.338700	-2.827159	-2.824648
Si	4.356881	-1.376268	-3.605411
O	2.447827	-4.018557	-4.333105
Al	2.995211	-2.999863	-5.871070
O	4.008573	-0.123894	-2.605191
O	3.228204	-1.408426	-4.809317
O	1.928981	0.959543	-1.517027
O	-1.980580	-1.647907	0.669863
O	0.918368	-1.229582	-3.337353
O	-0.263415	-0.006833	-5.150788
O	1.311519	-2.097064	-6.024094
O	2.291654	-4.547372	-6.697854
H	0.244846	2.389489	-6.503823
H	-3.060854	-0.151760	-4.667239
H	-6.644945	-2.989999	-2.448984
H	-10.573441	-6.281594	0.668027
H	0.487461	-2.429437	-6.378828
H	-3.132823	-5.398697	-3.269387
H	-6.143705	-7.989772	-2.197437
H	-4.121350	4.402359	-4.562065
H	-7.256662	1.698759	-2.990822
H	-10.830116	-0.939229	-0.796365
H	2.034080	-4.654642	-7.613668
H	-2.074135	-7.195339	-4.986160
H	-2.616593	2.428806	-5.285028
H	-5.112537	-0.708544	-3.027591
H	-9.365624	-3.458540	-0.958156
H	-0.199167	-4.082088	-5.061628
H	-3.851327	-7.194083	-2.774642
H	-0.954436	5.674812	-5.713831
H	-4.615736	2.920843	-3.287935
H	-8.024263	0.155786	-1.166053
H	1.652093	0.495214	-7.336808
H	-2.036301	-2.504455	-4.972704
H	-5.614796	-5.317875	-2.670251
H	-6.879287	4.461777	-3.865868
H	-9.731443	1.287213	-1.605700
H	-0.448402	0.942105	-5.046175
H	-3.750245	-1.906997	-2.738373
H	-7.424914	-4.835741	-0.734554
H	-5.187701	7.852683	-4.071625

H	-8.928988	4.930891	-2.020083
H	1.757770	-6.656288	-2.897780
H	-1.742416	-9.875717	-0.671582
H	0.555187	3.224255	-2.873889
H	-3.027264	0.304670	-0.758352
H	-6.741520	-2.712113	1.209722
H	1.098779	-2.176149	-3.240292
H	-2.323387	-4.864929	-0.633508
H	-5.974223	-7.679507	1.718034
H	-3.902310	5.143044	-1.650053
H	-7.437511	2.294840	0.468495
H	0.545792	2.358061	-0.230247
H	-4.154660	2.684195	2.289360
H	-2.784498	-3.036415	1.519501
H	1.442941	0.123053	-1.603053
H	-5.344397	4.146668	1.523682
H	-3.891244	-1.716722	0.328668
Si	1.273668	-0.292003	1.528204
O	0.347099	-0.406562	0.192413
O	0.458854	-0.598283	2.939720
O	1.827262	1.290958	1.560184
O	2.541675	-1.365599	1.525058
H	2.784670	1.357267	1.724149
H	3.050671	-1.415427	0.706047
Si	-1.248746	3.021126	1.978236
O	-2.177639	3.178206	0.594306
O	-1.152682	1.434676	2.407238
O	-2.109751	3.688291	3.253997
O	0.254602	3.712195	1.935077
H	-2.561492	4.520167	3.075298
H	0.964899	3.043536	1.854496
Si	-3.648563	-0.047692	3.795043
O	-3.980181	-0.264124	2.164344
O	-2.065413	-0.553415	3.969539
O	-4.668680	-0.980633	4.704585
O	-3.802665	1.462929	4.447860
H	-4.931497	-1.795669	4.265163
H	-3.170021	2.150857	4.180311
Si	-0.729296	0.357488	3.609396
C	-0.101132	1.182335	5.152265
H	-0.966372	1.585478	5.710659
H	0.309513	0.348320	5.754862
C	0.945423	2.253789	4.876182
H	1.731023	1.850782	4.203183
H	0.487712	3.098209	4.331231

C	1.622211	2.818411	6.106322
H	0.877138	3.304543	6.772175
H	2.342393	3.602233	5.799828
N	2.378065	1.805311	6.853310
C	3.098088	2.367463	7.993830
H	1.718405	1.103641	7.202016
C	3.896546	1.310599	8.732897
H	3.780477	3.146409	7.602960
H	2.406117	2.873133	8.697460
H	4.281782	1.749730	9.673545
H	3.220435	0.487138	9.027165
N	4.935589	0.757053	7.875734
H	5.724020	1.399933	7.788476
H	5.308443	-0.117397	8.242868
Au	4.701615	4.119382	1.097646
Au	5.641272	1.434666	1.005713
Au	6.259497	-1.245450	0.678834
Au	6.171404	3.262862	-1.058901
Au	4.391760	2.385164	3.402104
Au	6.802517	0.540987	-1.465849
Au	4.981846	-0.351454	3.062485
Au	7.393653	-2.123733	-1.786273
Au	5.566606	-3.136763	2.682279
Au	6.690987	-3.942872	0.267759
Au	4.295799	-2.242155	4.988897
Au	3.879955	0.482324	5.500094
Au	3.151334	4.850642	3.217432

Au_{13}/fHNT, 3D cluster

316

O	-6.661586	8.610940	2.795340
O	-8.966364	7.173239	1.633605
O	-0.752807	8.970395	1.250580
O	-5.660432	9.251706	-1.258397
O	-4.518358	8.800152	1.458688
O	-8.723470	-7.580713	-0.281892
O	-9.301801	7.152635	-2.988789
O	-4.279173	-8.997416	-0.093545
O	-8.328734	-6.629649	-4.802969
O	-5.131567	-8.917524	-2.941758
O	2.525677	6.665118	5.528186
O	4.171367	-3.414518	6.921562

O	2.808568	6.506530	2.849408
O	-2.338385	8.286061	5.055218
O	0.172053	8.445575	4.251599
O	5.115635	1.168503	4.101018
O	1.087895	-6.097667	7.457403
O	3.532231	-2.221281	9.048581
O	4.218226	1.300184	8.906447
O	4.826505	2.045108	6.674636
O	2.092873	5.183099	7.578875
O	-11.451032	2.797426	-4.834453
O	-11.102272	-1.888233	-5.456388
O	-11.295720	2.672186	0.023064
O	4.922513	-2.378465	4.475473
O	2.688229	-6.771471	2.959109
O	-0.951405	-9.324415	0.265060
O	-0.364382	-9.195765	3.285299
O	-2.796813	-9.041602	4.011292
O	-11.037550	-3.009797	-0.807576
O	1.969050	-7.535888	5.432886
O	-6.596110	-9.070835	0.929191
H	1.278911	-5.142069	7.589764
H	2.849267	-2.638832	9.586652
H	-11.910531	2.070626	0.463198
H	-9.628439	6.735926	2.183200
H	3.757871	2.016766	9.358232
H	-9.457037	-7.226480	0.234920
H	-3.495564	-8.692413	3.397046
H	-3.124563	8.055027	4.502656
H	2.870353	5.664033	7.242854
H	-11.633278	-2.689168	-0.119074
H	-7.389701	-8.770091	0.444383
H	-7.507666	8.391583	2.362797
H	0.576693	-9.239356	3.466134
H	0.285102	8.872420	3.394280
H	-3.358937	-9.194620	0.109114
H	-3.568482	8.929859	1.451540
H	-9.203732	7.077338	-3.941726
H	-0.385366	9.331082	0.438362
H	2.967407	6.585358	3.822905
H	5.637278	0.523016	3.611211
H	-6.040371	9.483258	-2.111240
H	4.908623	-2.863822	5.338048
H	2.610503	-7.221042	3.841993
H	-11.366947	3.742299	-4.684884
H	-11.444971	-1.318611	-6.160456

H	-8.803992	-6.011183	-5.365124
H	-4.682423	-9.391156	-2.219752
H	-0.657569	-9.863881	1.016296
H	1.588323	-8.414321	5.531152
H	2.145758	7.533019	5.329570
H	5.285836	1.846568	5.841042
H	3.746798	-4.227074	6.618191
H	4.063851	-2.972430	8.682749
H	4.895366	1.758706	8.332953
H	2.314699	4.223920	7.564105
H	-1.980512	9.117438	4.709154
H	-6.282342	9.371806	2.313441
H	-9.338853	7.193963	0.718468
H	-11.664957	2.766191	-0.887295
H	-11.495814	-2.872689	-1.666626
H	-8.866230	-7.280189	-1.219061
H	-6.142002	-9.725785	0.369601
H	-2.397759	-9.811990	3.578389
H	1.951431	-6.534478	7.360903
H	-5.797949	1.659015	-1.028116
H	-0.270328	0.218826	-0.413280
H	-3.653766	-3.078016	-0.223885
Al	-5.298437	7.290326	2.014972
O	-6.609388	5.891117	2.386515
Al	-7.536584	5.737577	0.762178
O	-3.970484	6.050787	1.387503
Al	-3.075386	5.638132	3.024118
O	-1.740581	6.992599	2.729446
Si	-0.913167	7.332268	1.343877
O	-6.370277	7.188744	0.437072
Si	-5.718554	7.637048	-1.022637
O	-4.376961	6.967504	3.651799
Si	-3.247274	6.157566	-0.099655
O	-3.037671	4.663495	-0.699629
Si	-3.066627	3.022936	-0.441191
O	-4.114041	2.478482	-1.611814
O	-4.214096	6.983939	-1.118822
O	-1.777664	6.855943	0.037886
O	-6.688257	7.037601	-2.207544
Si	-8.165396	6.293494	-2.156723
O	-7.972621	4.871734	-2.943747
Si	-7.960182	3.255958	-2.754479
O	-8.547054	2.857780	-1.231621
Al	-9.749268	1.423502	-0.930050
O	-10.826579	-0.116909	-0.462398

Al	-9.669432	-1.431478	-1.297522
O	-8.788813	-1.820463	0.316398
Al	-7.397254	-2.996288	-0.261155
O	-6.293053	-4.337874	-0.996142
Al	-7.261822	-5.866523	-0.575356
O	-8.655571	6.146399	-0.620651
O	-8.617485	4.233973	1.253345
Al	-7.542586	2.870104	0.393792
O	-6.267422	1.616489	-0.180391
Al	-5.214898	1.344822	1.360754
O	-4.152803	-0.032585	0.633865
Al	-5.176369	-1.539170	1.067345
O	-3.865032	-2.809451	0.682815
Al	-3.090212	-3.200014	2.320721
O	-4.321038	-1.787773	2.796708
O	-6.508697	4.408203	-0.090574
O	-6.522816	2.631074	1.979348
O	-8.838111	1.449802	0.757439
O	-6.312509	-0.188120	1.777248
Al	-3.025326	2.750028	2.771351
O	-3.873567	2.694234	1.001651
O	-4.400660	1.453433	3.055981
O	-6.496835	-2.925900	1.411589
O	-6.043357	-1.630231	-0.682320
Si	-5.215538	-1.596635	-2.132879
O	-8.421703	-4.570105	0.168497
O	-8.382045	-2.695412	-1.883661
Si	-7.758536	-2.705624	-3.430650
O	-8.695935	-1.802855	-4.407949
Si	-10.219718	-1.155481	-4.285017
O	-10.728519	-1.485752	-2.774693
O	-6.001884	-7.183521	-1.028125
Al	-5.196827	-7.614751	0.634845
O	-8.196806	-6.045868	-2.140254
Si	-7.531030	-5.824033	-3.606795
O	-6.583152	-6.352657	1.098555
Si	-5.192370	-7.345965	-2.473567
O	-5.976974	-6.413949	-3.567057
O	-3.669336	-6.729643	-2.223570
Si	-2.859207	-6.214673	-0.905517
O	-3.830094	-6.283509	0.431829
Al	-3.235692	-6.104323	2.225116
O	-4.552398	-7.558179	2.432808
O	-7.555659	-4.218992	-4.010566
O	-6.299333	-1.957141	-3.341809

O	-4.156298	-2.853514	-2.021133
O	-1.989155	4.268810	2.279908
O	-4.179634	4.141428	3.392339
O	-2.188459	5.640984	4.676835
Al	-0.866889	7.005937	4.467334
O	0.433413	5.675518	3.932704
Al	1.206828	5.415974	5.677403
O	-1.709751	1.504127	2.342861
Al	-0.880691	1.113641	3.956387
O	0.164394	-0.368697	3.394138
Al	-0.982747	-1.814292	3.868650
O	-2.321912	-3.169274	4.088864
O	-2.154124	2.460362	4.477410
O	0.583917	2.335198	3.724692
Al	1.277912	2.484030	5.506464
O	2.344810	3.974119	5.086299
O	-0.077236	1.084726	5.771649
O	-2.098821	-0.350737	4.288559
Si	1.503453	2.682612	2.388713
O	3.005254	2.069266	2.712026
Si	3.502579	0.982845	3.815564
O	3.273434	-0.557754	3.268410
Si	3.410488	-2.047513	3.969862
O	2.912718	-3.110436	2.815058
Si	1.374004	-3.357538	2.309199
O	0.382042	-3.134842	3.609468
Al	0.903986	-3.333687	5.451565
O	1.453373	-3.179853	7.321579
Al	2.981873	-1.990579	7.052495
O	0.915942	1.983839	1.031279
O	1.552120	4.251456	1.973985
Si	1.378701	5.789381	2.577346
O	-0.191835	6.591610	6.189909
O	0.539076	6.584183	1.400113
O	2.653599	1.189660	5.205818
Al	3.400309	0.970502	6.976487
O	2.074753	-0.368081	7.484535
O	2.050906	2.332154	7.300735
O	0.125591	3.865415	6.027191
O	-0.388581	-1.962182	5.655187
O	-1.597209	-1.990013	2.052005
Si	-0.601852	-1.790110	0.691891
O	2.363463	-2.132194	5.248938
O	1.966335	-4.881452	5.184200
Al	0.650449	-6.263116	5.448501

O	-0.369829	-4.687008	5.770713
O	4.275414	-0.680485	6.737119
O	-6.464208	2.622964	-2.970504
O	-8.922053	2.622252	-3.904620
Si	-10.411444	1.908922	-3.891973
O	-10.868153	1.806239	-2.324261
O	-10.281524	0.450542	-4.624604
O	-8.664027	0.115742	-1.710554
O	1.228382	-4.864970	1.708179
Si	1.214487	-6.389339	2.361624
O	0.919843	-2.287990	1.149603
O	0.654285	-7.305731	1.117520
Si	-0.879148	-7.759442	0.720344
O	0.130426	-6.430178	3.613367
Al	-1.289584	-7.715994	3.794932
O	-1.437455	-6.978183	-0.609366
O	-1.879490	-7.410025	1.985881
O	-2.409405	-4.663194	-1.224817
O	-0.561322	-0.121142	0.454571
O	-2.024882	-4.677445	1.771704
O	-4.340074	-4.627625	2.675948
O	-2.645844	-6.374046	3.976610
O	-0.769601	-7.498837	5.599315
H	-6.743130	-5.819609	1.878528
H	-4.926261	-1.977055	3.519377
H	-2.771434	2.307925	5.197962
H	0.094799	7.361745	6.690284
H	-2.858156	-5.841569	4.742623
H	-0.140546	-1.064017	5.917874
H	1.409314	2.133896	7.991396
H	-8.762994	-1.128873	0.982482
H	-6.903076	2.435277	2.838819
H	-4.912502	6.693842	4.402932
H	-1.261081	-7.746198	6.382788
H	0.701543	-2.808975	7.799923
H	-7.103298	-2.740785	2.135318
H	-4.324102	0.639024	3.557206
H	-2.545273	5.418789	5.537961
H	-1.111564	-4.533873	5.163465
H	1.268749	-0.148278	6.994791
H	-9.279767	-4.468273	-0.265081
H	-7.106304	-0.012467	1.250260
H	-4.999449	4.070600	2.879914
H	-5.236653	-7.406493	3.092952
H	-2.914803	-2.953020	4.815405

H	-0.732568	1.369821	6.419490
H	-9.381105	1.592004	1.538555
H	-6.693282	5.279539	3.119292
H	-5.083295	-4.439213	2.077798
H	-2.776053	-0.349891	3.594243
H	-0.695956	3.904773	5.509210
H	-11.541329	-0.049697	-1.114803
H	-9.464016	4.286442	0.789306
H	2.702257	-5.033923	4.577789
H	5.185465	-0.807153	6.459311
H	-5.355300	-4.240329	-1.211677
H	-3.191441	-0.007508	0.586771
H	-1.075609	4.264931	2.596611
H	-1.177883	-4.758286	2.234343
H	1.122851	-0.416131	3.494652
H	3.307367	3.994006	5.094278
H	-7.714926	0.161924	-1.529838
H	-5.588664	4.353967	0.203325
H	-3.282206	-2.851052	-2.441107
H	-3.801585	2.465939	-2.532738
H	0.691977	1.040019	1.068645
H	-1.972668	-4.269604	-0.451705
H	-5.748562	3.055286	-2.482125
H	-1.341896	1.343191	1.463635
Si	-0.037820	-2.509219	-2.210690
O	-0.985788	-2.398173	-0.899804
O	0.595782	-1.029102	-2.627388
O	-1.020014	-2.970995	-3.498034
O	1.174546	-3.613661	-2.035684
H	-0.871748	-3.889198	-3.756860
H	2.068893	-3.297866	-2.260254
Si	-3.281566	-0.168031	-3.691033
O	-4.428502	-0.107986	-2.474417
O	-1.800459	0.063715	-3.002184
O	-3.439393	1.209081	-4.632661
O	-3.253194	-1.507773	-4.650331
H	-4.331455	1.438275	-4.914995
H	-2.510537	-2.099876	-4.419963
Si	-0.694147	2.902686	-1.996503
O	-1.508960	2.334981	-0.653384
O	0.261118	1.643198	-2.553581
O	0.315416	4.167324	-1.649220
O	-1.599101	3.470303	-3.265382
H	0.932395	3.999433	-0.925618
H	-2.072882	2.800456	-3.786431

Si	-0.194021	0.252597	-3.353079
C	0.403109	0.183120	-5.108890
H	0.066556	-0.807823	-5.468430
H	1.493550	0.048259	-4.932146
C	0.191497	1.230538	-6.198382
H	0.898619	0.999611	-7.016055
H	-0.819252	1.120792	-6.629098
C	0.348031	2.693896	-5.809292
H	-0.579528	3.069144	-5.334839
H	0.474129	3.284715	-6.733823
N	1.478885	2.989669	-4.921781
C	1.755627	4.424446	-4.837613
H	1.233572	2.661097	-3.982816
C	2.760130	4.851266	-5.892194
H	2.159545	4.639869	-3.831541
H	0.825312	5.018906	-4.932618
H	2.413785	4.523932	-6.887999
H	2.823994	5.954844	-5.920545
N	4.060959	4.223287	-5.640409
H	4.676056	4.294469	-6.450606
H	4.542953	4.695431	-4.871530
Au	3.681958	-0.409342	-3.320787
Au	6.118550	1.034003	-3.655647
Au	3.884486	2.147299	-1.844905
Au	4.289817	-1.889207	-0.882224
Au	7.265754	0.871786	-0.941376
Au	4.775090	0.582492	0.443316
Au	6.702151	-1.530177	0.618750
Au	3.757576	1.991823	-4.710490
Au	5.849864	3.135152	-0.096762
Au	6.828843	-1.756996	-2.265538
Au	2.265383	0.041222	-0.640858
Au	4.571608	-3.084703	-3.388257
Au	8.698627	0.108015	-3.239945

Au_{14}/fHNT

317

O	-9.234255	7.420716	0.926681
O	-11.023706	5.187203	0.193266
O	-3.605662	9.022248	-0.857470
O	-8.327858	7.386390	-3.199206
O	-7.185981	7.883003	-0.489911

O	-6.704623	-8.981232	1.610385
O	-11.205208	4.042427	-4.294051
O	-2.049870	-9.053269	1.960222
O	-6.452374	-8.995050	-3.019588
O	-2.807863	-9.848034	-0.803664
O	0.051006	8.715706	3.713686
O	4.345410	0.043922	7.264336
O	0.444799	8.045165	1.129827
O	-5.054808	8.800764	3.055145
O	-2.660952	9.454605	2.152098
O	4.084324	3.958961	3.462297
O	2.097628	-3.187546	8.488800
O	3.342667	1.462100	9.091846
O	3.044701	4.912549	8.144677
O	3.491516	5.276166	5.783738
O	-0.020472	7.669662	6.056699
O	-12.028266	-1.031768	-5.048141
O	-10.395012	-5.460471	-4.620480
O	-11.987017	-0.019970	-0.293161
O	4.856365	0.670977	4.624884
O	3.952201	-4.388104	4.202898
O	1.228704	-8.371886	2.270437
O	1.669540	-7.415832	5.164330
O	-0.732704	-7.772248	5.919147
O	-10.162651	-5.452376	0.156495
O	3.396987	-4.746066	6.807650
O	-4.287600	-9.524779	3.051011
H	2.016439	-2.211656	8.398133
H	2.784246	1.005566	9.732198
H	-12.426784	-0.652022	0.290534
H	-11.556982	4.720448	0.848551
H	2.393113	5.558670	8.440460
H	-7.521859	-8.734113	2.059557
H	-1.481952	-7.773382	5.266376
H	-5.731464	8.246647	2.594852
H	0.605600	8.256472	5.595863
H	-10.843201	-5.160719	0.775784
H	-5.118601	-9.568143	2.538342
H	-9.975214	6.888046	0.582726
H	2.581026	-7.159631	5.318528
H	-2.643712	9.693060	1.217852
H	-1.117108	-8.941610	2.170652
H	-6.307855	8.261715	-0.557806
H	-11.062432	3.785522	-5.208923
H	-3.327067	9.278230	-1.741589

H	0.547483	8.380005	2.055288
H	4.776539	3.387569	3.111548
H	-8.731439	7.308709	-4.068918
H	4.950296	0.405976	5.573964
H	3.974383	-4.632477	5.165929
H	-12.209849	-0.091214	-5.116050
H	-10.859473	-5.178367	-5.421927
H	-7.061793	-8.671628	-3.689212
H	-2.267792	-10.007285	-0.009742
H	1.636564	-8.628539	3.112755
H	3.267931	-5.649871	7.112172
H	-0.545512	9.379854	3.339371
H	4.011822	5.029116	5.000456
H	4.167973	-0.900039	7.164359
H	4.069701	0.822288	8.885110
H	3.587370	5.397910	7.460871
H	0.455215	6.829267	6.248818
H	-4.927618	9.598872	2.520479
H	-9.063280	8.128281	0.274694
H	-11.360721	4.900333	-0.690352
H	-12.341058	-0.236311	-1.188581
H	-10.615569	-5.641173	-0.695557
H	-6.896514	-8.948580	0.635146
H	-3.655694	-10.139047	2.636664
H	-0.125918	-8.481205	5.655827
H	3.050074	-3.382482	8.463148
H	-6.393208	0.295491	-1.276400
H	-0.702813	0.592199	-0.542308
H	-3.061334	-3.372810	0.491639
Al	-7.540007	6.381962	0.415129
O	-8.429283	4.798390	1.133395
Al	-9.231302	4.038586	-0.383576
O	-5.906231	5.443683	0.035627
Al	-4.980801	5.667664	1.691960
O	-4.058613	7.233070	1.057757
Si	-3.315202	7.466512	-0.395733
O	-8.496655	5.641736	-1.063321
Si	-7.949675	5.912357	-2.607358
O	-6.613756	6.697242	2.050731
Si	-5.196485	5.408046	-1.461381
O	-4.569253	3.932950	-1.719592
Si	-4.159874	2.448155	-1.102143
O	-4.980751	1.390813	-2.084582
O	-6.321898	5.689802	-2.606037
O	-3.978103	6.492967	-1.532671

O	-8.683657	4.822006	-3.595321
Si	-9.902248	3.734400	-3.330055
O	-9.305539	2.280465	-3.786104
Si	-8.857842	0.814220	-3.241677
O	-9.358006	0.622311	-1.649487
Al	-10.131101	-0.980089	-0.995226
O	-10.759976	-2.610601	-0.159699
Al	-9.263854	-3.712101	-0.719002
O	-8.358147	-3.474997	0.910419
Al	-6.681983	-4.325172	0.563243
O	-5.232411	-5.443963	0.109247
Al	-5.758760	-7.044367	0.892833
O	-10.378394	3.806761	-1.784261
O	-9.874370	2.446797	0.466770
Al	-8.443151	1.271274	-0.102570
O	-6.857835	0.317353	-0.425237
Al	-5.816756	0.694823	1.101316
O	-4.398030	-0.467175	0.664691
Al	-4.983469	-2.058999	1.457560
O	-3.364376	-2.976261	1.322161
Al	-2.560667	-2.764112	2.978867
O	-4.143805	-1.671623	3.168980
O	-7.854907	2.884050	-0.952399
O	-7.443771	1.680290	1.461079
O	-9.311604	-0.329595	0.612195
O	-6.465692	-0.945674	1.886037
Al	-4.136514	2.922215	2.088322
O	-4.885010	2.242864	0.405293
O	-5.113152	1.397653	2.701189
O	-5.884497	-3.639628	2.146774
O	-5.741100	-2.772016	-0.197484
Si	-4.914028	-2.840846	-1.643698
O	-7.249869	-5.981019	1.367365
O	-7.663629	-4.675014	-1.051427
Si	-7.015920	-4.860751	-2.577256
O	-8.135257	-4.490068	-3.699177
Si	-9.780836	-4.272164	-3.672506
O	-10.224111	-4.382050	-2.110307
O	-4.174333	-8.034585	0.703111
Al	-3.331182	-7.846637	2.392288
O	-6.562956	-7.817144	-0.560205
Si	-5.940413	-7.756142	-2.060847
O	-5.022458	-6.939801	2.609183
Si	-3.309248	-8.289272	-0.696091
O	-4.286183	-7.875633	-1.942879

O	-2.020440	-7.241436	-0.641335
Si	-1.420724	-6.243851	0.500563
O	-2.374643	-6.273758	1.851487
Al	-1.904569	-5.542875	3.538552
O	-2.779659	-7.215812	4.109566
O	-6.390614	-6.351250	-2.811438
O	-5.820052	-3.742524	-2.706834
O	-3.553220	-3.701714	-1.292459
O	-3.540581	4.515940	1.235826
O	-5.644599	4.048458	2.421779
O	-4.177174	6.281834	3.271549
Al	-3.273077	7.872496	2.718431
O	-1.643779	6.862407	2.450585
Al	-0.880336	7.220669	4.182148
O	-2.518696	2.019114	1.904503
Al	-1.662140	2.240671	3.535609
O	-0.235948	1.012664	3.283352
Al	-0.958111	-0.546549	4.106743
O	-1.882131	-2.130344	4.668686
O	-3.269668	3.270295	3.785663
O	-0.580703	3.731169	2.988047
Al	-0.006242	4.458215	4.667300
O	0.624973	6.049423	3.889611
O	-0.934875	2.838713	5.283461
O	-2.443220	0.612611	4.226935
Si	0.247719	4.008065	1.578038
O	1.849795	3.915841	1.979257
Si	2.592376	3.281651	3.279956
O	2.808823	1.655155	3.098314
Si	3.326922	0.455380	4.109410
O	3.172418	-0.933288	3.238272
Si	1.775177	-1.697089	2.852439
O	0.722471	-1.468277	4.102889
Al	1.224685	-1.099978	5.924270
O	1.656151	-0.387928	7.693243
Al	2.808943	1.081417	7.114298
O	-0.086590	2.890361	0.431280
O	-0.121800	5.396614	0.822376
Si	-0.726243	6.923441	1.072930
O	-2.561292	8.054155	4.466284
O	-1.716155	7.174948	-0.222977
O	1.678840	3.554755	4.616823
Al	2.404816	3.949338	6.365578
O	1.480939	2.449052	7.203796
O	0.725939	4.928203	6.423152

O	-1.506763	5.553225	4.905247
O	-0.398714	-0.123380	5.860219
O	-1.447667	-1.285030	2.396854
Si	-0.504213	-1.132905	0.989405
O	2.305826	0.376855	5.409666
O	2.676714	-2.318523	5.973298
Al	1.781041	-3.911017	6.583339
O	0.360120	-2.642206	6.580002
O	3.704171	2.589419	6.471217
O	-7.240203	0.581368	-3.361309
O	-9.575955	-0.298276	-4.188392
Si	-10.813614	-1.368963	-3.966767
O	-11.270638	-1.238843	-2.401184
O	-10.268935	-2.862131	-4.359538
O	-8.707578	-2.082601	-1.500418
O	2.064466	-3.281876	2.608081
Si	2.448283	-4.565926	3.585211
O	1.080394	-1.079580	1.499452
O	2.196346	-5.854472	2.596489
Si	0.857439	-6.785901	2.362424
O	1.380403	-4.619635	4.850307
Al	0.360857	-6.169327	5.361870
O	0.146190	-6.504491	0.911516
O	-0.236925	-6.448516	3.551077
O	-1.402590	-4.740854	-0.171770
O	-0.915177	0.388791	0.388702
O	-1.116739	-3.979026	2.737725
O	-3.382990	-4.361238	3.685524
O	-1.315053	-5.242661	5.285179
O	0.748677	-5.420723	7.053770
H	-5.344710	-6.310122	3.255518
H	-4.695217	-1.852075	3.935639
H	-3.842703	3.120545	4.542439
H	-2.510670	8.964890	4.772295
H	-1.687062	-4.630906	5.919805
H	-0.413181	0.843464	5.907400
H	0.142938	4.722288	7.161845
H	-8.541713	-2.671791	1.404173
H	-7.781108	1.585681	2.354882
H	-7.075963	6.463204	2.861587
H	0.320706	-5.610904	7.888781
H	0.817936	-0.138817	8.101840
H	-6.539397	-3.469821	2.830975
H	-4.831802	0.768559	3.368596
H	-4.484806	6.169284	4.172060

H	-0.377120	-2.836951	5.979234
H	0.660253	2.325459	6.704793
H	-8.090052	-6.216617	0.951168
H	-7.261485	-1.115589	1.360184
H	-6.398524	3.644073	1.965977
H	-3.498360	-7.112722	4.741898
H	-2.532549	-1.927082	5.348221
H	-1.661841	3.071775	5.873072
H	-9.895432	-0.169809	1.359738
H	-8.364389	4.367157	1.986540
H	-4.131543	-4.521360	3.085786
H	-3.074300	0.273460	3.573148
H	-2.292287	5.250224	4.419514
H	-11.446480	-2.888496	-0.786201
H	-10.689078	2.161322	0.031505
H	3.443736	-2.396243	5.391748
H	4.621952	2.656822	6.198176
H	-4.351049	-5.145268	-0.154073
H	-3.479118	-0.192176	0.580830
H	-2.670379	4.832187	1.514454
H	-0.293795	-3.720286	3.177933
H	0.695650	1.252041	3.359570
H	1.544843	6.332212	3.860525
H	-7.812888	-1.740233	-1.366633
H	-6.964058	3.149847	-0.684969
H	-2.701089	-3.555140	-1.731542
H	-4.649929	1.258197	-2.989488
H	-0.045271	1.954479	0.685780
H	-1.112791	-4.080544	0.478892
H	-6.684460	1.290257	-3.006074
H	-2.095285	1.772100	1.071463
Si	0.325096	-2.311812	-1.706226
O	-0.651973	-2.158197	-0.417467
O	0.583135	-0.880069	-2.518722
O	-0.468823	-3.299773	-2.820291
O	1.755357	-3.005568	-1.270305
H	-0.160734	-4.213656	-2.776494
H	2.530566	-2.722022	-1.793379
Si	-3.360011	-1.308469	-3.483839
O	-4.566163	-1.306374	-2.328361
O	-2.038207	-0.564409	-2.840061
O	-3.826053	-0.248766	-4.703271
O	-2.949088	-2.754853	-4.156742
H	-4.728940	-0.341418	-5.026743
H	-2.081760	-3.072605	-3.834132

Si	-1.798321	2.581144	-2.678826
O	-2.470721	2.183013	-1.204522
O	-0.475559	1.574687	-2.888039
O	-1.242500	4.147134	-2.671549
O	-2.699078	2.508818	-4.064371
H	-0.921407	4.435772	-1.809894
H	-2.996865	1.634158	-4.371919
Si	-0.553923	-0.021932	-3.372248
C	-0.479857	-0.307153	-5.214219
H	-1.490351	-0.048256	-5.589164
H	-0.440150	-1.413062	-5.265247
C	0.575152	0.280654	-6.151384
H	1.596589	0.144840	-5.741372
H	0.554305	-0.291353	-7.094889
C	0.364811	1.739111	-6.516013
H	-0.671953	1.878274	-6.897513
H	1.045240	2.005938	-7.344982
N	0.613511	2.660273	-5.408735
C	0.338060	4.060232	-5.723259
H	0.050834	2.384663	-4.602541
C	1.555104	4.762945	-6.297173
H	0.024203	4.560838	-4.789657
H	-0.510898	4.150288	-6.429915
H	1.909781	4.230499	-7.197175
H	1.266243	5.781293	-6.620248
N	2.643096	4.759721	-5.320091
H	3.517919	5.089432	-5.726662
H	2.429355	5.385297	-4.540411
Au	6.704777	1.755634	2.118110
Au	6.662593	1.275500	-0.688632
Au	5.236649	1.817104	-3.269904
Au	4.237351	1.564889	0.766616
Au	6.244363	-0.844360	1.198104
Au	2.898614	1.759946	-1.571894
Au	6.816626	-0.704508	-2.829755
Au	2.803470	2.472021	-4.365671
Au	4.663971	-2.516818	-3.342684
Au	3.261240	-0.220776	-3.699736
Au	8.020360	1.792371	-3.050205
Au	4.253028	-0.794472	-0.967517
Au	6.497110	-2.683495	-0.771157
Au	7.359529	-3.445513	-3.313750

Au_{15}/fHNT

```
 318
O   -12.619149    2.371419    2.571158
O   -11.873611    4.782551    1.035213
O    -9.555361   -1.208131    6.473636
O   -10.291769    4.189863    5.591458
O   -11.095643    1.565175    4.427174
O    -0.307181    4.731303   -8.325467
O    -9.296152    7.945732    3.233044
O     2.391854    1.068416   -7.280110
O     1.827831    7.335568   -5.137993
O     3.651329    3.524290   -6.172092
O    -9.097888   -6.602188    4.225197
O    -2.169765   -9.481297   -2.843089
O    -7.306384   -5.149315    5.625441
O   -11.945396   -2.420565    3.268117
O   -10.562421   -3.848320    5.006630
O    -3.351027   -8.108681    2.311687
O    -1.860340   -7.623934   -6.510337
O    -4.510198  -10.236700   -3.398307
O    -6.616902  -10.424024   -0.496885
O    -5.587276   -9.438714    1.471596
O    -9.433046   -7.662882    1.911836
O    -6.041259   10.455243    0.054746
O    -2.249025   10.237144   -2.779896
O    -8.727338    7.273261   -2.454282
O    -1.160816   -8.450917   -0.489089
O     1.893493   -6.085219   -3.904413
O     3.771049   -1.757920   -6.088823
O     2.156423   -4.101287   -7.265507
O     0.630643   -2.662477   -8.704040
O    -4.151384    7.184432   -5.931967
O     0.690905   -7.138101   -6.064592
O     0.899533    2.214835   -8.976668
H    -2.529882   -7.785951   -5.808454
H    -4.810600  -10.076082   -4.300671
H    -8.813629    7.429326   -3.403904
H   -12.158351    4.918315    0.122997
H    -7.571440  -10.299587   -0.443201
H    -1.157158    5.000280   -8.694093
H     0.459091   -1.710032   -8.478414
H   -11.781729   -1.481138    3.008396
H    -9.256513   -8.019088    2.801013
```

H	-5.021773	7.236326	-6.346033
H	0.647617	3.155891	-8.897694
H	-12.558241	3.282650	2.229143
H	2.465773	-4.946933	-6.934748
H	-10.314851	-3.366242	5.804302
H	2.787685	0.214084	-7.079563
H	-10.794439	0.843367	4.981630
H	-8.646653	8.463190	3.716533
H	-9.183812	-0.956202	7.324105
H	-7.865428	-5.878416	5.257983
H	-2.399106	-8.251557	2.357543
H	-10.111219	5.036826	6.010227
H	-1.329910	-9.017337	-1.283055
H	1.661366	-6.612357	-4.714274
H	-6.757001	10.364446	0.688537
H	-2.377276	10.986212	-2.179836
H	1.528271	8.101652	-4.640230
H	3.745257	2.688451	-6.661887
H	3.830648	-2.496607	-6.715326
H	1.094117	-6.968508	-6.921857
H	-9.745807	-6.120369	4.759143
H	-4.773475	-9.284962	1.980886
H	-1.596070	-9.020572	-3.468502
H	-3.552507	-10.472844	-3.486234
H	-6.326918	-10.554902	0.449916
H	-8.661043	-7.894761	1.346419
H	-12.180193	-2.420138	4.208275
H	-12.716414	2.435546	3.541421
H	-11.505751	5.647641	1.340077
H	-8.412344	8.138126	-2.098332
H	-3.932951	8.089131	-5.614800
H	-0.028862	5.452555	-7.699914
H	1.871272	2.167442	-8.934290
H	1.586852	-2.755194	-8.835186
H	-1.144860	-8.263675	-6.353399
H	-5.156986	3.602608	0.022790
H	-2.248089	-1.169000	1.355681
H	-1.428384	1.094004	-2.713653
Al	-10.680235	1.710570	2.694013
O	-10.451359	2.391219	0.877770
Al	-9.773606	4.119015	1.155442
O	-8.901888	0.986776	2.778815
Al	-9.202797	-0.764827	2.077845
O	-9.435453	-1.515499	3.834401
Si	-8.526383	-1.261023	5.186667

O	-10.125131	3.524966	2.914816
Si	-9.320714	3.969962	4.297330
O	-11.033514	-0.055094	2.069385
Si	-7.812689	1.368939	3.967788
O	-6.328669	1.475634	3.317792
Si	-5.342956	1.221122	2.006456
O	-4.702954	2.724496	1.717054
O	-8.191914	2.817988	4.610309
O	-7.782562	0.194819	5.102206
O	-8.604241	5.422276	4.012548
Si	-8.715387	6.480569	2.745315
O	-7.179716	6.723047	2.234547
Si	-6.151895	6.477631	0.997592
O	-7.001859	5.946350	-0.350501
Al	-6.663102	6.584220	-2.103149
O	-6.295887	7.013312	-3.956035
Al	-4.413376	6.546869	-3.900276
O	-4.723559	4.822463	-4.579795
Al	-2.993042	4.022620	-4.444741
O	-1.171717	3.532324	-4.485143
Al	-0.741041	3.907651	-6.253185
O	-9.709688	5.884245	1.615180
O	-9.447401	4.538768	-0.685160
Al	-7.549640	4.147818	-0.691537
O	-5.814100	3.430400	-0.669587
Al	-6.093847	1.627079	-1.145502
O	-4.269297	1.161687	-1.235806
Al	-3.883895	1.573064	-3.021192
O	-2.232666	0.707246	-3.091019
Al	-2.598958	-0.950403	-3.835083
O	-4.370296	-0.194146	-3.672684
O	-7.923212	3.762697	1.147328
O	-7.891590	2.344310	-1.182942
O	-7.295055	4.819957	-2.511237
O	-5.710267	2.104572	-2.976620
Al	-7.009009	-0.853589	0.183916
O	-6.282609	0.911425	0.643734
O	-6.827632	-0.061040	-1.546010
O	-3.651708	2.279143	-4.819165
O	-3.152192	3.337536	-2.605782
Si	-1.990337	3.607788	-1.436513
O	-2.558623	4.432255	-6.276257
O	-2.658626	5.826755	-3.873595
Si	-1.493095	6.315274	-2.785028
O	-1.938383	7.720746	-2.095612

Si	-3.086682	8.870466	-2.435731
O	-3.945080	8.291504	-3.691520
O	0.961817	3.121139	-6.345917
Al	0.621759	1.421372	-7.117085
O	-0.083082	5.602261	-6.028112
Si	0.889906	6.024292	-4.795881
O	-1.100606	2.294927	-7.129961
Si	2.250736	3.390794	-5.327585
O	1.915250	4.753055	-4.483963
O	2.294539	2.098676	-4.283320
Si	1.494363	0.680171	-4.203912
O	0.364896	0.586701	-5.408654
Al	-0.565479	-0.987673	-5.915675
O	-0.204918	-0.203544	-7.689287
O	-0.010064	6.413161	-3.462244
O	-1.474039	5.183452	-1.595037
O	-0.741304	2.628926	-1.873954
O	-7.363958	-1.237433	2.013829
O	-8.819166	-0.249220	0.294012
O	-9.807897	-2.490168	1.659293
Al	-10.103056	-3.283352	3.373139
O	-8.326486	-4.050645	3.332448
Al	-8.847624	-5.764847	2.625751
O	-5.347469	-1.693310	0.156014
Al	-5.677423	-3.377651	-0.549260
O	-3.882411	-3.940909	-0.806765
Al	-3.614223	-3.455195	-2.629671
O	-3.339500	-2.654399	-4.350453
O	-7.446511	-2.621423	-0.477053
O	-5.800622	-4.278033	1.143389
Al	-6.662298	-5.924502	0.668869
O	-7.026213	-6.379007	2.456649
O	-6.389278	-5.142785	-1.114155
O	-5.341928	-2.748854	-2.346664
Si	-4.888326	-4.125509	2.520171
O	-4.033520	-5.536181	2.644566
Si	-3.712968	-6.693419	1.547534
O	-2.406402	-6.283502	0.625488
Si	-1.715236	-6.938189	-0.725019
O	-0.498497	-5.903815	-1.125960
Si	-0.658223	-4.412067	-1.784552
O	-1.979123	-4.444828	-2.773713
Al	-2.702455	-6.021090	-3.608864
O	-3.678255	-7.610648	-4.195015
Al	-3.730869	-8.539353	-2.475184

O	-3.845289	-2.867964	2.438189
O	-5.726563	-3.789131	3.869175
Si	-7.228524	-3.922776	4.566012
O	-10.542895	-4.918659	2.520544
O	-7.441318	-2.475208	5.328776
O	-5.016890	-6.894614	0.569958
Al	-5.592438	-8.601543	-0.134338
O	-5.491778	-7.990979	-1.984768
O	-7.288091	-7.661744	0.012854
O	-8.406054	-5.259390	0.824316
O	-4.311341	-5.048789	-3.365268
O	-2.680854	-1.850374	-2.117905
Si	-1.618871	-1.748227	-0.794447
O	-2.830656	-6.937220	-1.947851
O	-1.026756	-6.787770	-4.055899
Al	-0.749545	-6.034627	-5.807002
O	-2.459992	-5.332949	-5.347717
O	-3.937165	-9.248262	-0.759392
O	-4.970663	5.408992	1.382288
O	-5.428495	7.899559	0.674444
Si	-5.543937	8.993551	-0.557279
O	-6.574206	8.354639	-1.656006
O	-4.040029	9.247881	-1.152312
O	-4.846640	6.139635	-2.105349
O	0.690659	-4.030583	-2.615072
Si	1.372482	-4.540699	-4.038382
O	-0.916566	-3.254228	-0.649850
O	2.513113	-3.391253	-4.319697
Si	2.436962	-1.986832	-5.178072
O	0.227152	-4.490696	-5.233964
Al	0.442915	-3.597991	-6.924889
O	2.437649	-0.662295	-4.210705
O	1.044051	-1.982116	-6.063574
O	0.776407	0.644645	-2.722477
O	-2.635413	-1.513654	0.528269
O	-0.806852	-1.537319	-4.086120
O	-2.315682	-0.308871	-5.633574
O	-1.158443	-2.564713	-6.721745
O	-0.550969	-5.118953	-7.446631
H	-1.995428	1.967209	-7.229910
H	-4.906881	-0.193243	-4.470466
H	-8.012637	-2.607166	-1.253446
H	-11.258265	-5.399425	2.948057
H	-2.066084	-2.843163	-6.840209
H	-4.993440	-5.341027	-2.743522

H	-7.814529	-7.613208	-0.792420
H	-5.587156	4.433884	-4.419085
H	-8.411618	2.084539	-1.946953
H	-11.499074	-0.132144	1.230803
H	-1.035869	-5.248550	-8.261888
H	-4.524898	-7.302143	-4.540894
H	-4.452018	2.307412	-5.352607
H	-6.517909	-0.494211	-2.343999
H	-10.300793	-2.771044	0.887041
H	-2.515675	-4.365793	-5.285476
H	-5.689112	-7.043070	-1.966654
H	-2.727284	5.376658	-6.395789
H	-5.849892	3.063237	-2.964777
H	-8.805042	0.702623	0.111027
H	-0.976404	-0.077482	-8.251338
H	-4.157747	-2.636153	-4.856708
H	-7.235346	-5.021747	-1.561322
H	-8.072855	4.759900	-3.073925
H	-10.484696	1.951465	0.027159
H	-2.402031	0.656266	-5.551709
H	-5.213587	-1.787950	-2.316520
H	-8.466676	-4.294737	0.721481
H	-6.253904	7.981730	-3.918482
H	-9.558935	5.489813	-0.818474
H	-0.264982	-6.987669	-3.497044
H	-3.314312	-9.787667	-0.266939
H	-0.731193	2.948545	-3.852299
H	-3.866685	0.448523	-0.729480
H	-7.173074	-2.144280	2.289652
H	-0.674410	-2.489889	-3.971985
H	-3.516348	-4.749824	-0.429080
H	-6.651641	-7.128070	2.931587
H	-4.596905	5.294340	-1.706997
H	-7.681030	2.861513	1.402293
H	-0.140680	2.216003	-1.234298
H	-4.028631	3.060361	2.332184
H	-3.296603	-2.786227	1.641555
H	0.227148	-0.150989	-2.629572
H	-5.266835	4.579082	1.783644
H	-4.570759	-1.437318	0.671230
Si	0.811200	-0.383969	0.470076
O	-0.425474	-0.490680	-0.586255
O	0.399338	-0.541948	2.067802
O	1.505800	1.117223	0.228568
O	1.905750	-1.621862	0.175482

H	2.481478	1.101961	0.309497
H	1.984633	-1.846941	-0.760968
Si	-1.275109	3.121749	1.291275
O	-2.508340	3.301731	0.174029
O	-1.185012	1.550475	1.777378
O	-1.745358	3.910736	2.696842
O	0.215178	3.669210	0.840793
H	-2.165684	4.769976	2.580898
H	0.832348	2.937334	0.642577
Si	-3.346158	0.358558	3.841644
O	-4.097836	0.102506	2.364403
O	-1.811068	-0.273701	3.643216
O	-4.162764	-0.440597	5.038405
O	-3.221179	1.904074	4.416053
H	-4.576177	-1.256681	4.739301
H	-2.644717	2.530539	3.946448
Si	-0.545302	0.529984	2.932384
C	0.390333	1.408453	4.280869
H	-0.388050	1.759806	4.987310
H	0.951587	0.606473	4.800753
C	1.306235	2.552105	3.867494
H	2.132164	2.161631	3.233102
H	0.767930	3.281487	3.240645
C	1.893498	3.323253	5.033868
H	1.081076	3.885302	5.546105
H	2.617655	4.070634	4.661041
N	2.597137	2.476612	6.000481
C	3.040904	3.219445	7.174453
H	1.963951	1.731844	6.304669
C	3.856375	2.350997	8.111906
H	3.659784	4.066470	6.820442
H	2.187881	3.663378	7.727821
H	4.031169	2.900318	9.055904
H	3.274121	1.449246	8.373008
N	5.096274	1.914737	7.469651
H	5.774199	2.678578	7.429538
H	5.543743	1.159551	7.988878
Au	7.942146	1.446671	-1.484486
Au	8.389692	-1.225800	-0.534685
Au	5.896048	-0.544450	-1.900529
Au	6.171787	-3.065522	-0.574530
Au	4.845539	-3.496179	1.930489
Au	7.524804	3.450340	0.498957
Au	4.468119	-0.989004	0.588305
Au	5.412813	1.585768	-0.012526

Au	8.113430	0.824693	1.449191
Au	7.093676	-1.747209	1.964688
Au	4.555740	-1.336421	3.725266
Au	6.314324	2.855716	2.967266
Au	6.912742	0.261777	3.940331
Au	4.123913	1.145687	2.399873
Au	4.621698	1.312511	5.135152

Au_{16}/fHNT

319

O	-7.304489	8.165155	3.569681
O	-9.536112	6.884208	2.117910
O	-1.321435	8.693826	2.406611
O	-6.085220	9.288659	-0.315780
O	-5.091993	8.509147	2.385451
O	-9.127357	-7.530632	-1.549862
O	-9.617486	7.423281	-2.484508
O	-4.694237	-8.970883	-1.290201
O	-8.489248	-6.041099	-5.894350
O	-5.389489	-8.545188	-4.150389
O	1.727087	5.880203	6.547952
O	3.335686	-4.298056	6.801592
O	2.157099	6.045835	3.889253
O	-3.110403	7.558652	6.007807
O	-0.560358	7.807813	5.368478
O	4.414183	0.589882	4.611590
O	0.238625	-7.018537	6.839128
O	2.575933	-3.369044	9.018849
O	3.254079	0.142048	9.341118
O	3.980730	1.149726	7.252468
O	1.188635	4.162286	8.377668
O	-11.644195	3.328491	-4.958102
O	-11.242424	-1.248509	-6.121494
O	-11.754960	2.616679	-0.150158
O	4.215531	-2.975845	4.543581
O	2.085997	-7.147739	2.387133
O	-1.389813	-9.347194	-0.791467
O	-0.969827	-9.586002	2.249843
O	-3.439027	-9.514600	2.854416
O	-11.428067	-2.923886	-1.645760
O	1.235427	-8.203734	4.707162
O	-7.063446	-9.161550	-0.412662

H	0.418131	-6.086426	7.096266
H	1.866230	-3.846849	9.464193
H	-12.390482	1.967881	0.179628
H	-10.225508	6.385340	2.573436
H	2.766675	0.799928	9.850194
H	-9.889623	-7.239617	-1.035121
H	-4.104487	-9.091975	2.249407
H	-3.864133	7.398074	5.389032
H	1.981369	4.678351	8.145436
H	-12.061978	-2.687326	-0.957336
H	-7.830498	-8.802421	-0.900441
H	-8.124660	8.001815	3.067983
H	-0.039911	-9.653496	2.475510
H	-0.402239	8.334863	4.576525
H	-3.785688	-9.193449	-1.062608
H	-4.143700	8.636378	2.446218
H	-9.466999	7.463464	-3.432725
H	-0.911507	9.149111	1.665341
H	2.261989	6.006024	4.872387
H	4.964572	0.007018	4.076785
H	-6.418778	9.622546	-1.153974
H	4.156375	-3.561861	5.339122
H	1.961840	-7.700527	3.203620
H	-11.572357	4.248145	-4.691079
H	-11.548360	-0.597107	-6.769339
H	-8.935527	-5.358030	-6.402917
H	-4.978652	-9.103740	-3.467321
H	-1.135378	-9.974239	-0.095913
H	0.853531	-9.086643	4.677511
H	1.355034	6.766699	6.435082
H	4.485900	1.052239	6.427494
H	2.931765	-5.066918	6.379409
H	3.129925	-4.071804	8.594704
H	3.959740	0.664820	8.865294
H	1.414913	3.211369	8.259327
H	-2.737543	8.424858	5.784909
H	-6.902550	8.977734	3.204806
H	-9.857942	7.016323	1.192909
H	-12.074109	2.820952	-1.061389
H	-11.839100	-2.682802	-2.505820
H	-9.219757	-7.118683	-2.450273
H	-6.576623	-9.745204	-1.021486
H	-3.013642	-10.228019	2.354187
H	1.107958	-7.442652	6.738145
H	-6.203665	1.724166	-1.012432

H	-0.712167	0.206330	-0.273390
H	-4.087180	-3.080717	-0.669851
Al	-5.895134	6.945120	2.711544
O	-7.218671	5.514563	2.838732
Al	-8.054757	5.560805	1.159280
O	-4.529644	5.787172	2.012815
Al	-3.723931	5.177484	3.634268
O	-2.380605	6.554277	3.579175
Si	-1.479874	7.056840	2.292356
O	-6.878405	7.037689	1.076450
Si	-6.149485	7.657494	-0.280380
O	-5.063468	6.424514	4.345509
Si	-3.726413	6.071090	0.591455
O	-3.478054	4.659967	-0.172238
Si	-3.516457	3.000443	-0.116961
O	-4.493699	2.603903	-1.399453
O	-4.639319	7.017018	-0.371977
O	-2.269472	6.744030	0.892879
O	-7.050260	7.208094	-1.580499
Si	-8.524864	6.467016	-1.701177
O	-8.283327	5.150322	-2.642424
Si	-8.274584	3.523489	-2.649364
O	-8.942423	2.945648	-1.220325
Al	-10.153406	1.488462	-1.160752
O	-11.248341	-0.094464	-0.942530
Al	-10.041699	-1.301365	-1.865523
O	-9.249299	-1.884776	-0.264503
Al	-7.823285	-2.985681	-0.902560
O	-6.674901	-4.231359	-1.732481
Al	-7.658938	-5.797219	-1.553327
O	-9.097918	6.136576	-0.223392
O	-9.154719	4.011582	1.405059
Al	-8.028656	2.758901	0.447376
O	-6.718736	1.580673	-0.203137
Al	-5.751200	1.122098	1.349372
O	-4.645126	-0.160039	0.520841
Al	-5.684660	-1.705388	0.712231
O	-4.348958	-2.923179	0.249668
Al	-3.663510	-3.510787	1.868452
O	-4.924428	-2.163338	2.443246
O	-6.976150	4.341663	0.209924
O	-7.096382	2.327424	2.046045
O	-9.336270	1.308321	0.565040
O	-6.863618	-0.447193	1.516676
Al	-3.648112	2.340980	3.037550

O	-4.397744	2.501622	1.230166
O	-5.031593	1.022961	3.087455
O	-7.016257	-3.120238	0.813349
O	-6.454044	-1.582123	-1.080578
Si	-5.548536	-1.375898	-2.467720
O	-8.863229	-4.597300	-0.723139
O	-8.718880	-2.488416	-2.528463
Si	-8.011417	-2.313191	-4.028760
O	-8.897573	-1.296528	-4.939827
Si	-10.428477	-0.664927	-4.823473
O	-11.017957	-1.174041	-3.394364
O	-6.370616	-7.053003	-2.091995
Al	-5.656178	-7.684088	-0.451612
O	-8.505952	-5.783758	-3.177412
Si	-7.761686	-5.387972	-4.567603
O	-7.071068	-6.483811	0.084324
Si	-5.482393	-7.041589	-3.499806
O	-6.209713	-5.982271	-4.514118
O	-3.977928	-6.463831	-3.093890
Si	-3.243437	-6.113981	-1.680775
O	-4.285902	-6.341509	-0.416912
Al	-3.791476	-6.381875	1.414818
O	-5.111551	-7.846861	1.372681
O	-7.770792	-3.745842	-4.775228
O	-6.562442	-1.584505	-3.770083
O	-4.491330	-2.639373	-2.451709
O	-2.592856	3.905423	2.790872
O	-4.840492	3.650028	3.757739
O	-2.928985	4.978331	5.321431
Al	-1.603587	6.355272	5.351295
O	-0.270419	5.095957	4.732085
Al	0.407232	4.625500	6.472478
O	-2.305860	1.152703	2.534591
Al	-1.564912	0.567982	4.132207
O	-0.484431	-0.838158	3.453250
Al	-1.649857	-2.327617	3.685890
O	-2.993451	-3.695911	3.666882
O	-2.870574	1.845051	4.741375
O	-0.094882	1.804900	4.130607
Al	0.499742	1.735541	5.952722
O	1.581885	3.262802	5.774914
O	-0.862093	0.317857	5.972054
O	-2.793342	-0.922744	4.217600
Si	0.895080	2.308926	2.898936
O	2.379424	1.657225	3.227784

Si	2.819990	0.444143	4.217619
O	2.627585	-1.018450	3.476583
Si	2.732146	-2.582409	3.999367
O	2.302860	-3.496708	2.699016
Si	0.795244	-3.676987	2.083247
O	-0.267446	-3.610587	3.344425
Al	0.153523	-4.031930	5.174876
O	0.598898	-4.106623	7.077113
Al	2.134907	-2.897395	7.038062
O	0.385792	1.780820	1.436811
O	0.959909	3.916267	2.680097
Si	0.747288	5.370423	3.454403
O	-1.022295	5.734088	7.045656
O	-0.029809	6.303792	2.337488
O	1.895201	0.483544	5.573922
Al	2.544596	0.050104	7.343469
O	1.198738	-1.336734	7.612477
O	1.173808	1.365973	7.755233
O	-0.685119	3.046756	6.572438
O	-1.153942	-2.691845	5.471395
O	-2.163048	-2.280934	1.830354
Si	-1.093349	-1.920871	0.557315
O	1.616918	-2.818430	5.199378
O	1.235345	-5.538699	4.781236
Al	-0.087314	-6.938868	4.803978
O	-1.130252	-5.410696	5.257717
O	3.438350	-1.562043	6.954821
O	-6.766402	2.917486	-2.857775
O	-9.169303	3.035861	-3.918733
Si	-10.654175	2.329977	-4.074201
O	-11.195734	2.039715	-2.557852
O	-10.478229	0.970516	-4.969413
O	-9.021584	0.281901	-2.032737
O	0.689049	-5.100357	1.297416
Si	0.645664	-6.692490	1.760143
O	0.400910	-2.473994	1.038178
O	0.158331	-7.450385	0.385553
Si	-1.349121	-7.848908	-0.147162
O	-0.505227	-6.881595	2.936327
Al	-1.927712	-8.176354	2.882928
O	-1.836909	-6.911261	-1.401389
O	-2.418791	-7.652496	1.094443
O	-2.783241	-4.536420	-1.785093
O	-1.048853	-0.235307	0.529738
O	-2.563554	-4.913705	1.204322

O	-4.925035	-4.967719	1.979404
O	-3.297445	-6.862796	3.150629
O	-1.508359	-8.180184	4.726149
H	-7.275784	-6.048541	0.913011
H	-5.567577	-2.437056	3.103408
H	-3.525830	1.608199	5.403225
H	-0.766726	6.437377	7.650397
H	-3.553649	-6.426274	3.962535
H	-0.924413	-1.832639	5.853899
H	0.496136	1.087306	8.380585
H	-9.262912	-1.278829	0.480665
H	-7.522384	2.030140	2.853369
H	-5.638253	6.063421	5.027513
H	-2.041032	-8.519189	5.445806
H	-0.179563	-3.794390	7.554734
H	-7.662257	-3.022428	1.519726
H	-4.979255	0.153748	3.490053
H	-3.331557	4.654583	6.128492
H	-1.838202	-5.183425	4.633597
H	0.419898	-1.057323	7.109344
H	-9.696642	-4.441651	-1.187711
H	-7.628042	-0.207142	0.971964
H	-5.630673	3.643716	3.196254
H	-5.831599	-7.774355	2.007722
H	-3.626182	-3.567564	4.380554
H	-1.553141	0.524208	6.612608
H	-9.921865	1.356437	1.326598
H	-7.340083	4.819114	3.486528
H	-5.635112	-4.706528	1.368478
H	-3.431478	-0.836282	3.492324
H	-1.477182	3.150496	6.018664
H	-11.926514	0.052904	-1.620307
H	-9.974741	4.121880	0.904961
H	2.004039	-5.618608	4.202196
H	4.362783	-1.656494	6.714160
H	-5.727152	-4.110838	-1.882808
H	-3.682742	-0.131869	0.530006
H	-1.698042	3.860997	3.154490
H	-1.742870	-5.051998	1.699631
H	0.467262	-0.899802	3.599794
H	2.542466	3.279159	5.838103
H	-8.084020	0.303515	-1.795902
H	-6.073400	4.249989	0.545212
H	-3.595534	-2.588381	-2.819611
H	-4.131173	2.702031	-2.296586

H	0.164031	0.839969	1.347511
H	-2.391192	-4.240256	-0.947275
H	-6.080413	3.285810	-2.282170
H	-1.889681	1.098291	1.663907
Si	-0.339236	-2.302188	-2.383323
O	-1.362049	-2.330337	-1.118195
O	0.356171	-0.798025	-2.614373
O	-1.261580	-2.628919	-3.749866
O	0.836886	-3.458262	-2.342994
H	-0.991012	-3.452545	-4.175235
H	1.697292	-3.222704	-1.957422
Si	-3.477337	0.198092	-3.655680
O	-4.752896	0.142449	-2.578307
O	-2.071390	0.268954	-2.797137
O	-3.513527	1.702299	-4.410314
O	-3.399959	-0.978252	-4.806325
H	-4.356420	1.966043	-4.796285
H	-2.690090	-1.624887	-4.624189
Si	-1.070548	3.021316	-1.563823
O	-1.952633	2.330808	-0.327168
O	-0.039101	1.847097	-2.147678
O	-0.187531	4.306218	-0.971636
O	-1.856402	3.687362	-2.867229
H	0.214421	4.114888	-0.114089
H	-2.335508	3.079697	-3.462464
Si	-0.463315	0.561914	-3.131000
C	-0.236946	0.866477	-4.949814
H	-1.052356	1.538283	-5.283246
H	-0.493292	-0.115843	-5.389383
C	1.128170	1.347606	-5.461991
H	1.933933	1.103007	-4.739856
H	1.387289	0.799965	-6.382855
C	1.187736	2.826685	-5.794834
H	0.453818	3.046811	-6.605353
H	2.187790	3.066778	-6.203578
N	0.962309	3.694883	-4.647486
C	0.861863	5.099221	-5.005760
H	0.104908	3.422763	-4.158210
C	0.577575	5.946227	-3.780350
H	0.066040	5.286160	-5.759875
H	1.819114	5.403370	-5.473753
H	0.415441	6.993144	-4.088775
H	-0.364872	5.596460	-3.325538
N	1.637416	5.848652	-2.763482
H	2.227462	6.679312	-2.750313

```
 H      1.208075     5.751036    -1.841058
Au      5.599103     3.832651    -3.885397
Au      2.919039     3.856515    -2.874047
Au      7.140793     1.637677    -3.200339
Au      2.539396     1.825533    -0.963606
Au      8.554075    -0.765005    -2.507671
Au      2.080559    -0.460189     0.651251
Au      4.361617    -2.032085     0.404041
Au      6.985206    -2.298552    -0.765486
Au      6.595924    -0.955106     1.754307
Au      4.617253    -3.403440    -2.026575
Au      5.745428    -0.928967    -3.087739
Au      3.169448    -0.850992    -2.043038
Au      4.627351     0.910643     0.749819
Au      7.092856     0.771336    -0.510163
Au      4.135832     1.346906    -3.514827
Au      5.242311     2.889155    -1.187306
```

Au_{17}/fHNT

```
  320
 O     11.652273    -3.586649    -4.654942
 O     12.188762    -0.684339    -4.786814
 O      6.543485    -6.916593    -5.145778
 O      8.961387    -2.721713    -7.794479
 O      9.402334    -4.466035    -5.416730
 O      5.257695     8.889990     4.251979
 O      9.932555     1.711270    -8.050256
 O      1.491618     6.763074     6.009019
 O      2.887257    10.181973     0.482061
 O      0.619496     8.603202     3.841393
 O      5.514492    -9.669731    -0.074086
 O      1.134804    -5.355730     8.199764
 O      3.793717    -8.503654    -1.794615
 O      9.519314    -7.259107    -2.232917
 O      7.271767    -8.608949    -2.549156
 O      0.679868    -7.392189     3.150301
 O      2.688162    -1.997028    10.016468
 O      3.224091    -6.651474     8.758729
 O      3.998784    -9.139198     6.288387
 O      2.626543    -8.934905     4.293114
 O      6.372295    -9.461786     2.336301
 O      8.853213     6.579954    -6.592703
```

O	6.441789	9.450558	-3.692963
O	11.314561	4.278542	-3.088343
O	-0.347217	-5.318196	5.870990
O	-1.199666	-0.525889	7.573053
O	-0.926506	4.629176	6.962287
O	0.317568	2.755970	9.066181
O	2.586380	3.903671	9.119379
O	8.472099	7.902370	0.351107
O	0.378652	-0.759818	9.735302
O	3.746443	7.833018	6.445554
H	2.987157	-2.755337	9.466247
H	3.863309	-6.209848	9.330283
H	11.778673	4.831961	-2.446538
H	12.810956	-0.249848	-4.190381
H	4.864354	-9.480886	6.036265
H	6.222127	8.912296	4.260443
H	2.911434	4.436228	8.345941
H	9.718288	-6.351211	-2.568941
H	5.796260	-10.097415	1.874954
H	9.413382	7.774829	0.522717
H	4.193536	8.395557	5.783036
H	11.965386	-2.703056	-4.923804
H	-0.304398	2.086037	9.356995
H	6.889209	-8.536373	-3.431400
H	0.837519	6.188440	6.419804
H	8.739294	-5.157396	-5.377514
H	9.315017	2.139059	-8.649438
H	5.969692	-6.990779	-5.913936
H	4.231866	-9.110075	-1.146990
H	-0.224493	-7.115074	3.334733
H	8.873798	-2.214043	-8.606828
H	-0.058535	-5.422613	6.812032
H	-0.838471	-0.619781	8.494119
H	9.237216	5.889468	-7.138594
H	6.537745	9.661905	-4.633042
H	3.177847	10.383701	-0.411948
H	0.490915	8.256827	4.741860
H	-0.948791	4.409418	7.907236
H	0.375901	-0.032518	10.365603
H	6.027004	-9.849600	-0.875346
H	1.755980	-8.723901	3.915121
H	0.973195	-4.451250	8.496980
H	2.338374	-6.375646	9.105285
H	3.360634	-9.584980	5.662529
H	5.827151	-9.024601	3.029941

H	9.386956	-7.827302	-3.006646
H	11.403217	-4.062872	-5.471123
H	11.980281	-0.026081	-5.493841
H	11.134616	4.888568	-3.842938
H	8.403072	8.520381	-0.410515
H	4.974098	9.241789	3.366094
H	2.851132	8.192360	6.578247
H	1.755961	4.308554	9.413693
H	1.821893	-2.251210	10.378101
H	6.247933	1.806311	-2.330500
H	1.894246	-1.210039	-0.104847
H	3.246657	2.873280	1.857304
Al	9.700891	-3.315790	-4.080559
O	10.338903	-1.800388	-3.026558
Al	10.093836	-0.345216	-4.185934
O	7.889849	-3.142242	-3.461173
Al	7.946241	-4.243274	-1.900869
O	7.314638	-5.783391	-2.866394
Si	6.079238	-5.875203	-3.955038
O	9.609075	-1.819524	-5.264485
Si	8.506886	-1.827528	-6.506092
O	9.772488	-4.487849	-2.579330
Si	6.587160	-2.979323	-4.472364
O	5.527647	-1.939747	-3.814471
Si	5.055346	-1.059471	-2.489447
O	4.992143	0.495189	-3.073254
O	7.078344	-2.368679	-5.901426
O	5.834418	-4.416604	-4.656753
O	8.358409	-0.283582	-7.051848
Si	9.200884	1.097739	-6.704853
O	8.080819	2.192557	-6.230723
Si	7.548055	3.063355	-4.964003
O	8.655361	2.969633	-3.704182
Al	9.160758	4.468178	-2.659022
O	9.621542	5.871337	-1.405635
Al	7.796754	6.298045	-0.903061
O	7.860857	5.202879	0.622727
Al	6.053966	5.273568	1.241387
O	4.312393	5.703941	1.825337
Al	4.671335	7.051504	3.053171
O	10.340361	0.784662	-5.598054
O	10.584270	1.027220	-2.942427
Al	8.788607	1.548322	-2.434066
O	7.040342	1.739628	-1.775084
Al	6.982163	0.496631	-0.357942

O	5.262528	0.972009	0.250803
Al	5.677798	2.336008	1.464245
O	3.998592	2.424954	2.272587
Al	4.154067	1.388228	3.801183
O	5.877570	1.119128	2.968696
O	8.350320	0.182583	-3.703690
O	8.791531	0.286255	-1.013422
O	9.402662	3.070033	-1.368600
O	7.433343	1.932468	0.851577
Al	6.653657	-2.427958	-0.045762
O	6.308851	-1.014539	-1.364017
O	7.332511	-0.891470	0.866403
O	6.313000	3.862907	2.489066
O	5.344241	3.773373	0.182060
Si	3.956034	3.896137	-0.737286
O	6.441797	6.680197	2.499154
O	6.028906	6.493190	-0.243468
Si	4.722987	6.818979	-1.228621
O	5.244450	7.345133	-2.677810
Si	6.699663	7.889741	-3.262513
O	7.768189	7.710512	-2.047948
O	2.975528	7.233779	3.839264
Al	3.106218	6.190016	5.418196
O	4.454220	8.499345	1.952314
Si	3.250131	8.621024	0.866693
O	4.881068	6.117594	4.660256
Si	1.526663	7.482311	3.058224
O	1.882498	7.946140	1.529099
O	0.763034	6.006777	3.017551
Si	1.069945	4.554704	3.692870
O	2.491323	4.600130	4.537545
Al	3.086201	3.266633	5.749662
O	3.618772	4.895257	6.726650
O	3.671889	7.865055	-0.544127
O	3.971171	5.381213	-1.483693
O	2.737353	3.907237	0.372490
O	6.202357	-3.780302	-1.306472
O	8.390322	-2.802207	-0.751434
O	8.178363	-5.595349	-0.621756
Al	7.606747	-7.173047	-1.536995
O	5.831344	-6.970607	-0.792956
Al	6.096340	-8.134909	0.718231
O	4.956954	-2.335778	0.715367
Al	5.058768	-3.396371	2.234596
O	3.400207	-2.924233	3.029527

Al	3.953570	-1.535702	4.211308
O	4.547982	0.042435	5.124540
O	6.813064	-3.619649	1.473445
O	4.312792	-4.970719	1.424723
Al	4.815307	-6.345336	2.664239
O	4.368478	-7.733844	1.477830
O	5.428797	-4.751343	3.637695
O	5.580937	-1.883794	3.319979
Si	3.038399	-5.150243	0.378409
O	1.854011	-5.909993	1.247969
Si	1.658359	-6.099452	2.851486
O	0.937044	-4.771760	3.516519
Si	0.636891	-4.297306	5.070548
O	-0.027277	-2.796497	4.937874
Si	0.752739	-1.412004	4.538686
O	2.282733	-1.510097	5.150083
Al	2.811698	-2.593019	6.651006
O	3.471852	-3.918032	7.928107
Al	2.645441	-5.497037	7.123824
O	2.472750	-3.714271	-0.163907
O	3.385471	-5.927013	-1.004271
Si	4.436787	-7.017928	-1.685426
O	7.873298	-8.170403	0.053244
O	4.735302	-6.396964	-3.184709
O	3.124051	-6.323064	3.557240
Al	3.438672	-7.506368	5.054465
O	4.184863	-6.081727	6.159280
O	5.149114	-7.597059	4.134529
O	6.492415	-6.667909	1.895810
O	4.418302	-2.670024	5.647956
O	3.362415	-0.180333	2.977594
Si	1.960165	-0.308085	2.027211
O	2.077495	-4.165637	5.874778
O	1.274309	-2.231060	7.700322
Al	1.864829	-0.672717	8.666380
O	3.411425	-1.104675	7.641508
O	2.016281	-6.974079	6.169067
O	6.068857	2.571809	-4.458390
O	7.399253	4.606442	-5.460335
Si	8.242163	5.995174	-5.163332
O	9.390951	5.618942	-4.060795
O	7.184263	7.127271	-4.634464
O	7.421835	4.911931	-2.133284
O	-0.047220	-0.133202	5.154709
Si	-0.273993	0.482523	6.677976

O	0.879330	-1.214210	2.913968
O	-0.883058	1.977977	6.371297
Si	-0.128243	3.431839	6.193647
O	1.196477	0.624802	7.427106
Al	1.857160	2.232143	8.253340
O	-0.127500	3.949598	4.637328
O	1.438302	3.279581	6.690749
O	1.162294	3.483051	2.446117
O	2.449013	-1.224026	0.698555
O	2.489437	1.838068	4.604804
O	4.726635	2.888589	4.872391
O	3.488022	2.224489	7.246615
O	2.528320	0.919060	9.436005
H	5.628628	5.536941	4.808871
H	6.646927	1.287086	3.520289
H	7.604570	-3.464907	1.996012
H	8.227987	-9.052166	-0.096191
H	4.266902	1.684715	7.378429
H	4.722920	-3.492032	5.237179
H	5.924853	-7.385104	4.664872
H	8.469495	4.459946	0.607049
H	9.464303	0.247895	-0.329658
H	10.472509	-4.327960	-1.938781
H	3.223102	1.019428	10.086984
H	4.435575	-3.875809	7.895422
H	7.228858	3.806984	2.778763
H	7.230860	-0.688326	1.798473
H	8.824294	-5.633907	0.085006
H	3.696433	-0.430074	7.004283
H	4.607210	-5.460345	5.548286
H	6.887698	7.379726	2.002705
H	7.809339	2.589808	0.247248
H	8.698162	-1.987198	-1.176506
H	4.545409	4.939674	6.984280
H	5.467286	-0.042269	5.396302
H	6.378853	-4.799839	3.797320
H	10.285522	2.971694	-0.999687
H	10.560816	-1.722943	-2.097695
H	5.031334	3.538769	4.216582
H	5.711723	-1.119119	2.737842
H	6.841219	-5.915150	1.389369
H	9.828709	6.601013	-2.010344
H	10.985972	1.763089	-3.423918
H	0.337826	-2.333740	7.488141
H	1.137378	-7.359258	6.193202

H	3.533302	5.137333	1.739754
H	4.528796	0.354553	0.337084
H	5.689775	-4.519779	-0.952468
H	2.075641	1.119540	5.105273
H	2.720351	-3.565529	3.269376
H	3.661612	-8.374767	1.606407
H	6.828579	4.183084	-1.904345
H	7.801066	-0.520272	-3.329398
H	1.858487	3.534660	0.201668
H	4.256138	0.731117	-3.663618
H	2.294676	-3.021119	0.491976
H	1.406481	2.601495	2.772593
H	5.965822	1.619265	-4.318359
H	4.144606	-2.066491	0.266112
Si	-0.324486	1.111443	0.599283
O	1.146265	1.047505	1.283615
O	-0.501468	0.031126	-0.656445
O	-0.485981	2.633294	-0.120462
O	-1.462448	0.882873	1.767958
H	-0.960599	3.259648	0.442592
H	-2.378524	0.750866	1.459163
Si	2.215035	2.457266	-2.492875
O	3.768935	2.658854	-1.913402
O	1.638254	1.039268	-1.877947
O	2.295166	2.116372	-4.134422
O	1.140236	3.683875	-2.251441
H	2.797483	2.726194	-4.685742
H	0.478907	3.466534	-1.564196
Si	2.379012	-1.759397	-3.144770
O	3.517158	-1.572257	-1.932976
O	0.885759	-1.448692	-2.448888
O	2.372201	-3.325290	-3.694284
O	2.525253	-0.847763	-4.516048
H	2.768288	-3.949248	-3.076733
H	2.326468	0.104612	-4.462103
Si	0.302515	0.081789	-2.119307
C	-0.889808	0.693632	-3.405867
H	-0.825569	1.799553	-3.369565
H	-1.874263	0.471604	-2.946498
C	-0.911765	0.229752	-4.862618
H	-1.871041	0.569785	-5.296238
H	-0.121018	0.744816	-5.435683
C	-0.754851	-1.263418	-5.122145
H	0.316590	-1.541064	-5.120359
H	-1.133362	-1.488503	-6.134423

N	-1.482081	-2.128959	-4.180612
C	-1.276423	-3.542269	-4.518235
H	-1.033745	-2.004533	-3.265465
C	-2.149046	-4.453170	-3.680160
H	-0.208149	-3.815328	-4.401054
H	-1.527076	-3.676005	-5.587645
H	-1.845933	-5.503487	-3.851789
H	-1.981881	-4.241983	-2.607659
N	-3.558903	-4.203541	-3.971320
H	-3.810096	-4.553148	-4.897774
H	-4.175446	-4.667297	-3.302561
Au	-4.953156	0.890213	-3.353859
Au	-6.234672	2.901254	-1.778787
Au	-6.594459	-1.416657	-3.403878
Au	-8.934696	-0.956819	-1.872326
Au	-8.547036	1.642340	-0.718639
Au	-5.152781	-2.534699	-1.101810
Au	-3.577026	-0.193205	-1.075692
Au	-4.687577	1.727127	0.739167
Au	-7.125750	0.579716	1.644781
Au	-7.369089	-2.086831	0.590902
Au	-7.724518	-3.531902	-1.842392
Au	-3.805602	-1.707112	-3.638173
Au	-9.668845	-0.447042	0.868791
Au	-7.016845	3.346557	0.958285
Au	-3.420208	2.641363	-1.632048
Au	-4.787481	-1.078140	1.353244
Au	-7.844653	1.190783	-3.430854

Au_{18}/fHNT

321

O	7.764983	-8.365215	3.054299
O	9.955476	-7.020405	1.597647
O	1.744037	-8.807638	2.063530
O	6.412563	-9.281140	-0.841559
O	5.512914	-8.638121	1.927547
O	9.512588	7.566393	-1.306904
O	9.882992	-7.321141	-3.026613
O	5.098977	9.010101	-0.827270
O	8.723810	6.306194	-5.698965
O	5.697718	8.729905	-3.726971
O	-1.150554	-6.198978	6.442674

O	-2.689476	3.959291	7.273435
O	-1.668318	-6.225101	3.794605
O	3.656608	-7.867777	5.657465
O	1.085572	-8.072723	5.090614
O	-3.868091	-0.804259	4.871215
O	0.423268	6.661020	7.348935
O	-1.863107	2.913667	9.413599
O	-2.551197	-0.606480	9.576691
O	-3.351783	-1.501728	7.463994
O	-0.542315	-4.580261	8.339732
O	11.851967	-3.112578	-5.351404
O	11.439548	1.520113	-6.263487
O	12.124238	-2.650777	-0.519440
O	-3.650583	2.759339	4.980539
O	-1.568003	7.028069	2.972881
O	1.814939	9.374147	-0.201074
O	1.496128	9.457175	2.860700
O	3.983323	9.344128	3.379008
O	11.781510	2.961041	-1.715924
O	-0.635706	7.959080	5.314926
O	7.496725	9.145082	-0.019703
H	0.246736	5.717631	7.563477
H	-1.136382	3.364791	9.859322
H	12.774055	-2.022601	-0.177799
H	10.662363	-6.548687	2.055287
H	-2.051312	-1.291864	10.034831
H	10.289547	7.245921	-0.833270
H	4.626104	8.950527	2.731401
H	4.390627	-7.678623	5.023261
H	-1.345277	-5.080259	8.107489
H	12.436192	2.686515	-1.061893
H	8.245258	8.808398	-0.550379
H	8.569254	-8.179636	2.534904
H	0.574514	9.516857	3.120098
H	0.898491	-8.557447	4.278199
H	4.199702	9.224457	-0.558663
H	4.566379	-8.764298	2.012924
H	9.701319	-7.311608	-3.970159
H	1.307373	-9.222245	1.313709
H	-1.740740	-6.235728	4.781409
H	-4.432213	-0.192193	4.385606
H	6.716524	-9.572657	-1.706393
H	-3.561944	3.303185	5.802838
H	-1.413910	7.537371	3.812229
H	11.783442	-4.044495	-5.129905

H	11.720243	0.901786	-6.953781
H	9.149140	5.648444	-6.256503
H	5.312781	9.254133	-3.002845
H	1.587132	9.965467	0.533886
H	-0.249744	8.840713	5.318234
H	-0.787670	-7.080025	6.272056
H	-3.883091	-1.359576	6.662254
H	-2.295025	4.747231	6.878341
H	-2.426495	3.639763	9.044739
H	-3.275145	-1.100952	9.098096
H	-0.766690	-3.623546	8.278081
H	3.271512	-8.719711	5.402646
H	7.346498	-9.156125	2.661492
H	10.246073	-7.105883	0.656954
H	12.412177	-2.809017	-1.450018
H	12.162738	2.763004	-2.600356
H	9.573024	7.201161	-2.229906
H	6.993718	9.761494	-0.581249
H	3.546043	10.084255	2.930514
H	-0.446368	7.093476	7.298676
H	6.553101	-1.691318	-1.150979
H	1.097823	-0.190421	-0.153941
H	4.477552	3.098370	-0.491605
Al	6.335577	-7.096470	2.307136
O	7.671045	-5.680033	2.464148
Al	8.451434	-5.642931	0.757881
O	4.954863	-5.898159	1.714432
Al	4.206278	-5.369703	3.390848
O	2.853708	-6.736091	3.309219
Si	1.908378	-7.167622	2.028623
O	7.264247	-7.108551	0.637900
Si	6.487646	-7.654275	-0.724285
O	5.560935	-6.657514	3.992304
Si	4.103875	-6.104799	0.307596
O	3.839035	-4.654994	-0.373737
Si	3.888906	-3.001653	-0.232202
O	4.826208	-2.542671	-1.526228
O	4.979129	-7.003512	-0.732883
O	2.653601	-6.786229	0.621841
O	7.348048	-7.142085	-2.028521
Si	8.822291	-6.402026	-2.159425
O	8.557910	-5.037413	-3.023085
Si	8.558613	-3.412376	-2.945886
O	9.276281	-2.912034	-1.511776
Al	10.497203	-1.465031	-1.417167

O	11.608081	0.099833	-1.153896
Al	10.379076	1.357952	-1.973157
O	9.642973	1.861166	-0.318934
Al	8.203383	2.999656	-0.851997
O	7.035907	4.291416	-1.578310
Al	8.034570	5.841728	-1.351261
O	9.445351	-6.150869	-0.686285
O	9.568038	-4.113171	1.046754
Al	8.418704	-2.807881	0.192565
O	7.095218	-1.592042	-0.352801
Al	6.181777	-1.210272	1.252338
O	5.056792	0.117696	0.527937
Al	6.111199	1.646640	0.764319
O	4.768344	2.892392	0.409439
Al	4.139734	3.398430	2.078087
O	5.410758	2.017716	2.540767
O	7.349604	-4.371767	-0.091287
O	7.541826	-2.455684	1.841229
O	9.738065	-1.370891	0.341630
O	7.308356	0.343556	1.463521
Al	4.127820	-2.505850	2.943925
O	4.816916	-2.575974	1.106964
O	5.520008	-1.198052	3.016025
O	7.453780	3.048717	0.894231
O	6.820783	1.612962	-1.056904
Si	5.870063	1.485979	-2.420451
O	9.258225	4.595392	-0.624189
O	9.042352	2.583291	-2.530044
Si	8.285133	2.488892	-4.013225
O	9.134948	1.516927	-5.004209
Si	10.665060	0.873662	-4.971124
O	11.304007	1.305699	-3.537901
O	6.736793	7.129141	-1.781699
Al	6.080181	7.677597	-0.088299
O	8.827885	5.908658	-3.000931
Si	8.036186	5.588440	-4.384361
O	7.504694	6.445221	0.338103
Si	5.802924	7.194299	-3.158159
O	6.490354	6.185763	-4.249155
O	4.309142	6.602719	-2.733150
Si	3.619220	6.183398	-1.316484
O	4.703820	6.340850	-0.077652
Al	4.269849	6.288555	1.769035
O	5.596520	7.748143	1.758932
O	8.028733	3.959217	-4.676528

O	6.841127	1.753947	-3.744792
O	4.820529	2.748002	-2.306172
O	3.055789	-4.050951	2.651900
O	5.335339	-3.855426	3.555973
O	3.468163	-5.254674	5.111336
Al	2.136294	-6.625678	5.113906
O	0.791083	-5.330384	4.604732
Al	0.173556	-4.947666	6.388463
O	2.776919	-1.287470	2.547426
Al	2.092137	-0.782986	4.196624
O	0.998390	0.660953	3.627045
Al	2.179643	2.131418	3.897566
O	3.530001	3.493159	3.904786
O	3.409424	-2.095369	4.695766
O	0.615519	-2.011916	4.179775
Al	0.081264	-2.034334	6.021666
O	-1.015176	-3.545751	5.801181
O	1.451403	-0.625339	6.069098
O	3.331543	0.696101	4.318158
Si	-0.417195	-2.447380	2.957093
O	-1.886084	-1.807258	3.367876
Si	-2.286805	-0.645115	4.432905
O	-2.110066	0.853004	3.762284
Si	-2.188166	2.388272	4.368137
O	-1.796241	3.366755	3.103170
Si	-0.308527	3.572225	2.448121
O	0.794447	3.436196	3.668459
Al	0.436113	3.764119	5.531081
O	0.053677	3.742252	7.448296
Al	-1.489946	2.543192	7.397669
O	0.047102	-1.846553	1.508123
O	-0.498703	-4.040967	2.657969
Si	-0.269502	-5.534102	3.348872
O	1.614460	-6.090465	6.856280
O	0.465067	-6.411767	2.160314
O	-1.318378	-0.758513	5.754130
Al	-1.906932	-0.414389	7.564120
O	-0.544774	0.950959	7.859698
O	-0.531246	-1.755595	7.862080
O	1.277956	-3.380853	6.533563
O	1.744594	2.404957	5.714854
O	2.631558	2.178583	2.026163
Si	1.522973	1.888504	0.775812
O	-1.032883	2.557198	5.541274
O	-0.649039	5.293803	5.251516

Al	0.681954	6.685296	5.302741
O	1.730081	5.131295	5.642472
O	-2.803328	1.219476	7.288764
O	7.048048	-2.790012	-3.072925
O	9.414196	-2.863573	-4.217261
Si	10.897353	-2.156906	-4.385042
O	11.489960	-1.947733	-2.874440
O	10.700290	-0.752246	-5.202713
O	9.344644	-0.210205	-2.188001
O	-0.219651	5.033855	1.733614
Si	-0.151689	6.599736	2.276092
O	0.044215	2.423174	1.330020
O	0.294896	7.425610	0.927060
Si	1.786449	7.844740	0.366153
O	1.038178	6.722888	3.421853
Al	2.465824	8.012625	3.388354
O	2.227351	6.971130	-0.950135
O	2.894991	7.579857	1.560025
O	3.146490	4.615308	-1.486723
O	1.463279	0.207669	0.659314
O	3.026994	4.838457	1.523778
O	5.412853	4.842299	2.222295
O	3.835753	6.681181	3.542676
O	2.107044	7.922932	5.242153
H	7.733828	5.966823	1.136053
H	6.076782	2.254205	3.192578
H	4.087384	-1.895847	5.346969
H	1.374644	-6.822988	7.432074
H	4.115790	6.202181	4.322114
H	1.522602	1.528105	6.059933
H	0.168171	-1.512513	8.478267
H	9.677361	1.217450	0.393163
H	7.995777	-2.202349	2.648309
H	6.159866	-6.334609	4.672678
H	2.664986	8.222011	5.960360
H	0.845481	3.402441	7.883250
H	8.121956	2.911775	1.572932
H	5.486041	-0.350604	3.464391
H	3.898848	-4.974798	5.920290
H	2.415866	4.933593	4.984456
H	0.215511	0.694632	7.317417
H	10.075054	4.460430	-1.123398
H	8.053105	0.128745	0.882241
H	6.106744	-3.823449	2.969799
H	6.336520	7.639845	2.365293

H	4.184974	3.325399	4.589633
H	2.161804	-0.867463	6.675022
H	10.347972	-1.460801	1.079965
H	7.817721	-5.019529	3.142566
H	6.100991	4.610033	1.575627
H	3.945079	0.644545	3.568735
H	2.050844	-3.459186	5.949351
H	12.262823	-0.015172	-1.860370
H	10.370586	-4.200946	0.514857
H	-1.435780	5.406807	4.703036
H	-3.734564	1.330166	7.083916
H	6.083046	4.182853	-1.703300
H	4.095078	0.093173	0.567377
H	2.173631	-4.021583	3.046643
H	2.223793	4.954441	2.052360
H	0.052391	0.718975	3.807887
H	-1.973254	-3.561275	5.895095
H	8.415220	-0.240055	-1.921797
H	6.458869	-4.293720	0.277876
H	3.912907	2.719954	-2.646502
H	4.433904	-2.592645	-2.414749
H	0.271410	-0.903293	1.460162
H	2.780314	4.277889	-0.652806
H	6.379084	-3.184688	-2.494752
H	2.332805	-1.186344	1.694906
Si	0.729241	2.336360	-2.120047
O	1.777514	2.375724	-0.883674
O	0.138392	0.800460	-2.325578
O	1.587094	2.706971	-3.519045
O	-0.495105	3.431655	-1.974343
H	1.362515	3.580351	-3.864459
H	-1.394368	3.059901	-1.920799
Si	3.889430	-0.081660	-3.766351
O	5.052008	-0.013928	-2.562604
O	2.452332	-0.440053	-3.024629
O	4.205844	-1.439965	-4.682311
O	3.698376	1.219904	-4.754824
H	5.138563	-1.627765	-4.829459
H	2.969316	1.806616	-4.472538
Si	1.406743	-3.037604	-1.570739
O	2.312086	-2.338639	-0.356229
O	0.416976	-1.872863	-2.250204
O	0.404501	-4.215022	-0.959313
O	2.207958	-3.823731	-2.790903
H	0.211635	-4.141564	-0.017981

H	2.597885	-3.252321	-3.469252
Si	0.802113	-0.487885	-3.136418
C	0.022505	-0.492980	-4.819391
H	-0.072181	0.579263	-5.073115
H	-1.020604	-0.818430	-4.614015
C	0.602494	-1.260041	-6.002305
H	-0.132258	-1.229083	-6.825724
H	1.506818	-0.744347	-6.372140
C	0.978564	-2.703894	-5.725677
H	1.948242	-2.725206	-5.172913
H	1.190073	-3.208912	-6.685408
N	-0.060249	-3.461851	-5.043318
C	0.228392	-4.884323	-5.035783
H	-0.110639	-3.145065	-4.070342
C	-0.644657	-5.647950	-4.067867
H	1.282670	-5.102501	-4.761407
H	0.081707	-5.276982	-6.062713
H	-0.368648	-6.716689	-4.114870
H	-0.458761	-5.308621	-3.032590
N	-2.082651	-5.468046	-4.360410
H	-2.252182	-5.629118	-5.356898
H	-2.629068	-6.172878	-3.862289
Au	-6.045704	-0.199117	2.166776
Au	-8.296764	-0.368800	0.410586
Au	-3.866256	1.431009	1.462562
Au	-4.063371	3.205121	-0.715270
Au	-6.301892	3.023582	-2.465251
Au	-8.274710	1.065750	-1.995288
Au	-6.460998	1.845356	0.159463
Au	-3.813760	-1.337731	0.756271
Au	-2.617826	0.721027	-0.991662
Au	-6.442514	-2.559484	0.587827
Au	-7.335947	-1.627450	-2.001899
Au	-6.122862	0.451071	-3.773163
Au	-3.740444	2.027324	-3.317442
Au	-3.442702	-0.724277	-3.406981
Au	-5.677704	-2.334831	-4.184955
Au	-1.979695	-2.061059	-1.266506
Au	-4.702882	-2.857410	-1.548239
Au	-2.968637	-3.457420	-3.750274

Au_{19}/fHNT

322

O	-6.959981	8.948187	2.906731
O	-9.342777	7.754006	1.632045
O	-0.996875	8.838927	1.543430
O	-5.781196	9.625494	-1.092944
O	-4.767731	8.991385	1.638645
O	-10.313740	-6.897832	-0.773805
O	-9.534239	7.907142	-2.996061
O	-6.016265	-8.702001	-0.507364
O	-9.697457	-5.844066	-5.247086
O	-6.769235	-8.459128	-3.374299
O	1.935366	6.123962	5.831972
O	2.661160	-4.099394	6.929733
O	2.287032	6.025058	3.158478
O	-2.753598	8.176999	5.276179
O	-0.214893	8.141944	4.550028
O	4.084842	0.469650	4.293796
O	-0.657485	-6.518767	7.286720
O	2.061228	-2.921981	9.076893
O	3.052523	0.528936	9.073507
O	3.792182	1.287312	6.886347
O	1.312517	4.622014	7.818235
O	-11.992448	3.815798	-5.048288
O	-12.029912	-0.860699	-5.818225
O	-12.000272	3.525813	-0.195338
O	3.574888	-3.056910	4.542410
O	1.018442	-7.188755	2.815465
O	-2.741933	-9.329076	-0.065672
O	-2.240597	-9.346529	2.972641
O	-4.672144	-9.003526	3.634067
O	-12.207369	-2.128301	-1.210054
O	0.159114	-7.964425	5.240547
O	-8.361701	-8.604944	0.446097
H	-0.388980	-5.588078	7.456704
H	1.328326	-3.294968	9.580919
H	-12.678124	2.966722	0.206557
H	-10.056943	7.359158	2.147509
H	2.641813	1.268481	9.536020
H	-11.029796	-6.497278	-0.266345
H	-5.318648	-8.575701	3.012391
H	-3.539136	8.032798	4.694016
H	2.138695	5.043556	7.520889
H	-12.794427	-1.778593	-0.528294
H	-9.110866	-8.221125	-0.050600
H	-7.807908	8.817088	2.443164

H	-1.312897	-9.477672	3.178549
H	-0.038730	8.583873	3.711196
H	-5.123248	-8.984959	-0.285441
H	-3.810448	9.037899	1.662866
H	-9.413323	7.853380	-3.947830
H	-0.574509	9.191381	0.754640
H	2.421602	6.059281	4.138202
H	4.563902	-0.203262	3.797444
H	-6.112929	9.915832	-1.947910
H	3.492255	-3.565912	5.387330
H	0.874696	-7.657191	3.680072
H	-11.831885	4.744599	-4.864513
H	-12.300047	-0.241641	-6.512069
H	-10.099810	-5.169243	-5.801266
H	-6.385385	-8.992454	-2.656266
H	-2.519327	-9.915319	0.674925
H	-0.298849	-8.808937	5.298188
H	1.638114	7.027474	5.652122
H	4.258481	1.075598	6.059894
H	2.177765	-4.861943	6.587113
H	2.537238	-3.704844	8.701103
H	3.784267	0.944334	8.535312
H	1.451133	3.648072	7.777329
H	-2.314715	8.984393	4.968739
H	-6.501658	9.687751	2.461811
H	-9.683333	7.835719	0.707924
H	-12.331417	3.680058	-1.112113
H	-12.625050	-1.924974	-2.076641
H	-10.400711	-6.556873	-1.703946
H	-7.948529	-9.279209	-0.122208
H	-4.327706	-9.791887	3.186857
H	0.167745	-7.026011	7.200060
H	-6.580253	2.070266	-1.123647
H	-1.219314	0.134901	-0.401140
H	-4.878830	-2.858547	-0.419564
Al	-5.692125	7.538722	2.121158
O	-7.129843	6.248280	2.407777
Al	-8.015653	6.227021	0.753502
O	-4.457107	6.208280	1.490074
Al	-3.652479	5.668196	3.136556
O	-2.197263	6.909606	2.925895
Si	-1.300773	7.218920	1.576665
O	-6.718937	7.580363	0.510925
Si	-5.985712	8.015487	-0.913687
O	-4.853492	7.085778	3.771634

Si	-3.681225	6.297957	0.028557
O	-3.582832	4.810760	-0.615547
Si	-3.762572	3.171414	-0.415309
O	-4.814620	2.757587	-1.631250
O	-4.540926	7.236959	-0.989153
O	-2.161930	6.860846	0.231295
O	-6.966034	7.540219	-2.145253
Si	-8.502716	6.926576	-2.161601
O	-8.408816	5.518700	-2.990480
Si	-8.541713	3.902878	-2.855704
O	-9.208009	3.510036	-1.364468
Al	-10.538288	2.177376	-1.145850
O	-11.758532	0.722958	-0.761358
Al	-10.693578	-0.660809	-1.607281
O	-9.900621	-1.175350	0.016914
Al	-8.598361	-2.449422	-0.560377
O	-7.591543	-3.858638	-1.308628
Al	-8.701224	-5.309350	-0.967473
O	-9.051477	6.774889	-0.645923
O	-9.237030	4.808829	1.162630
Al	-8.257468	3.383912	0.288296
O	-7.077882	2.042390	-0.291525
Al	-6.101387	1.631969	1.268801
O	-5.139861	0.190527	0.526208
Al	-6.302608	-1.233918	0.879236
O	-5.094347	-2.601021	0.489335
Al	-4.407616	-3.108661	2.134440
O	-5.526314	-1.609995	2.622784
O	-7.080141	4.840340	-0.114202
O	-7.312127	3.007430	1.893176
O	-9.681397	2.071370	0.566756
O	-7.339573	0.188325	1.601876
Al	-3.843842	2.796021	2.787747
O	-4.637980	2.869789	0.993867
O	-5.334116	1.616104	2.989186
O	-7.747855	-2.510174	1.138723
O	-7.119075	-1.194150	-0.896389
Si	-6.249599	-1.188130	-2.320096
O	-9.767622	-3.940563	-0.213432
O	-9.502333	-2.013311	-2.199049
Si	-8.834040	-2.029519	-3.727161
O	-9.659093	-1.018349	-4.699597
Si	-11.124451	-0.244675	-4.598147
O	-11.706756	-0.576321	-3.114919
O	-7.546066	-6.716443	-1.428588

Al	-6.833528	-7.267990	0.241032
O	-9.598875	-5.357480	-2.563475
Si	-8.870978	-5.148864	-4.002173
O	-8.119622	-5.904880	0.707618
Si	-6.708843	-6.903650	-2.855127
O	-7.375600	-5.873044	-3.938300
O	-5.146875	-6.430642	-2.541275
Si	-4.336899	-6.029728	-1.184081
O	-5.351389	-6.055353	0.122061
Al	-4.800033	-5.984803	1.936536
O	-6.243080	-7.324038	2.057455
O	-8.744446	-3.535969	-4.351947
O	-7.319182	-1.414342	-3.571649
O	-5.303790	-2.535041	-2.221757
O	-2.665703	4.233264	2.377594
O	-4.892673	4.262746	3.422428
O	-2.820648	5.542043	4.812941
Al	-1.380371	6.792388	4.687348
O	-0.183617	5.370966	4.145157
Al	0.509670	4.990573	5.901335
O	-2.627915	1.454130	2.354892
Al	-1.886414	0.942602	3.977174
O	-0.956040	-0.606964	3.395005
Al	-2.237863	-1.961042	3.787575
O	-3.695131	-3.200308	3.923709
O	-3.054584	2.378334	4.507086
O	-0.315380	2.038385	3.828621
Al	0.332889	2.070380	5.633396
O	1.536940	3.474119	5.294346
O	-1.145526	0.787023	5.812503
O	-3.236097	-0.419594	4.224893
Si	0.671962	2.345826	2.531811
O	2.104429	1.594017	2.876705
Si	2.471486	0.434415	3.956535
O	2.127504	-1.062477	3.351290
Si	2.113573	-2.579695	4.005552
O	1.562280	-3.558559	2.801773
Si	0.024525	-3.654577	2.244353
O	-0.984640	-3.386914	3.522731
Al	-0.539572	-3.687978	5.371124
O	-0.037621	-3.641141	7.260117
Al	1.595419	-2.581911	7.074931
O	0.069041	1.743710	1.135379
O	0.868699	3.916631	2.171943
Si	0.809829	5.444220	2.821816

O	-0.797691	6.267149	6.413395
O	0.079062	6.345616	1.648777
O	1.600512	0.670970	5.328320
Al	2.269881	0.332307	7.111052
O	0.818586	-0.900760	7.535678
O	1.033496	1.795682	7.442728
O	-0.711678	3.530075	6.167624
O	-1.714745	-2.215978	5.584240
O	-2.808150	-2.025767	1.949302
Si	-1.754693	-1.871888	0.621417
O	1.023718	-2.612634	5.250782
O	0.393143	-5.313399	5.081827
Al	-1.044628	-6.582593	5.261760
O	-1.934712	-4.934330	5.608005
O	3.006359	-1.380436	6.840915
O	-7.099902	3.148861	-3.050451
O	-9.518277	3.391730	-4.053475
Si	-11.063313	2.810991	-4.107266
O	-11.575907	2.699617	-2.557525
O	-11.036860	1.370438	-4.884827
O	-9.546056	0.804958	-1.939016
O	-0.231741	-5.124036	1.588799
Si	-0.397466	-6.661048	2.189641
O	-0.299224	-2.513762	1.109215
O	-0.995504	-7.485770	0.899842
Si	-2.549205	-7.791360	0.444162
O	-1.519566	-6.646250	3.407998
Al	-3.050170	-7.808338	3.505564
O	-2.996248	-6.923224	-0.873676
O	-3.554704	-7.395718	1.691879
O	-3.745196	-4.514185	-1.437811
O	-1.565635	-0.205335	0.446032
O	-3.457091	-4.655518	1.566219
O	-5.786461	-4.432201	2.405530
O	-4.290627	-6.359531	3.693950
O	-2.569990	-7.693839	5.330298
H	-8.257301	-5.384535	1.500320
H	-6.167877	-1.768258	3.321153
H	-3.704940	2.257899	5.204250
H	-0.461459	6.993338	6.947474
H	-4.480033	-5.834752	4.471200
H	-1.398476	-1.351521	5.884095
H	0.355942	1.632668	8.107789
H	-9.836055	-0.509793	0.706478
H	-7.734509	2.818796	2.734391

H	-5.433835	6.836546	4.497569
H	-3.105199	-7.921738	6.090682
H	-0.769226	-3.221218	7.729192
H	-8.358394	-2.295559	1.850788
H	-5.343783	0.782865	3.464545
H	-3.222013	5.325029	5.655577
H	-2.641149	-4.698183	4.985468
H	0.050234	-0.596296	7.030960
H	-10.599740	-3.750783	-0.667516
H	-8.098415	0.448934	1.058808
H	-5.699133	4.279748	2.884825
H	-6.931979	-7.133938	2.702655
H	-4.289555	-2.955961	4.640020
H	-1.793733	1.107819	6.450747
H	-10.234227	2.235926	1.336502
H	-7.289018	5.623761	3.116768
H	-6.491614	-4.161092	1.793225
H	-3.888720	-0.337988	3.512052
H	-1.510191	3.657108	5.628151
H	-12.444099	0.872607	-1.431149
H	-10.060976	4.949410	0.676779
H	1.131746	-5.510465	4.491747
H	3.910322	-1.577265	6.584936
H	-6.642603	-3.836579	-1.494031
H	-4.178912	0.133102	0.507302
H	-1.766230	4.139816	2.719797
H	-2.635082	-4.824347	2.049729
H	-0.008860	-0.740934	3.521033
H	2.496905	3.409708	5.330324
H	-8.602616	0.762505	-1.729962
H	-6.177842	4.696902	0.203702
H	-4.420021	-2.595704	-2.616386
H	-4.475668	2.746552	-2.542794
H	-0.236559	0.822320	1.134419
H	-3.300492	-4.184534	-0.639737
H	-6.365233	3.501642	-2.527601
H	-2.248039	1.289264	1.481525
Si	-1.168350	-2.577460	-2.309470
O	-2.118952	-2.399219	-1.000436
O	-0.362242	-1.170631	-2.693450
O	-2.180752	-2.919334	-3.609243
O	-0.114277	-3.846164	-2.240962
H	-2.037046	-3.812280	-3.947728
H	0.761838	-3.664853	-1.861466
Si	-4.124226	0.099835	-3.723099

O	-5.340775	0.242997	-2.589390
O	-2.688209	0.114497	-2.913506
O	-4.045718	1.530443	-4.600010
O	-4.191859	-1.174638	-4.765331
H	-4.864109	1.833539	-5.008578
H	-3.541784	-1.868654	-4.538968
Si	-1.372832	2.857872	-1.923574
O	-2.271878	2.347344	-0.613395
O	-0.499159	1.523709	-2.452590
O	-0.304402	4.045534	-1.463576
O	-2.119872	3.497243	-3.255473
H	0.187119	3.814476	-0.660330
H	-2.673885	2.896890	-3.787628
Si	-1.077848	0.196779	-3.304111
C	-0.774452	0.309004	-5.128286
H	-1.372838	1.135882	-5.556210
H	-1.243807	-0.612819	-5.518736
C	0.701613	0.392386	-5.556404
H	1.378133	0.034772	-4.752967
H	0.876939	-0.282493	-6.409756
C	1.129875	1.781504	-5.984392
H	0.491062	2.114381	-6.834153
H	2.169554	1.753047	-6.361222
N	1.084222	2.750530	-4.896578
C	1.296927	4.119987	-5.340206
H	0.185348	2.698481	-4.408907
C	1.189250	5.076676	-4.169731
H	0.568759	4.419716	-6.123579
H	2.303486	4.176017	-5.799247
H	1.260411	6.116086	-4.535483
H	0.190599	4.965974	-3.712485
N	2.190377	4.786147	-3.138022
H	3.008409	5.390366	-3.215860
H	1.789962	4.934106	-2.210330
Au	4.572248	-2.499179	1.007457
Au	2.765436	-4.442438	0.104011
Au	8.106255	1.553219	2.325772
Au	6.318449	-0.490760	1.732496
Au	7.815425	1.056011	-0.403359
Au	7.522897	0.385200	-3.047139
Au	7.083482	-0.336199	-5.717590
Au	5.604180	-1.822132	-3.872277
Au	4.118493	-3.129819	-1.964002
Au	6.150488	-1.236735	-1.075940
Au	2.008937	-1.767031	-0.382365

Au	5.675792	2.425903	1.142909
Au	4.993490	1.072260	-4.493934
Au	3.781506	0.282233	0.599100
Au	3.396414	-0.373898	-2.599744
Au	5.350147	1.898658	-1.733532
Au	2.918835	2.438783	-3.152518
Au	1.479379	0.928323	-0.901200
Au	3.233971	3.049963	-0.100280

Au_{20}/fHNT

323

O	0.382541	-3.444220	8.367948
O	2.119279	-5.718484	7.633047
O	-3.111877	0.369273	5.100615
O	-2.137259	-5.055514	5.384660
O	-1.231067	-2.513089	6.651810
O	13.236850	-4.133330	-2.128823
O	0.507280	-8.617818	4.396568
O	12.495262	-0.212232	-4.551495
O	10.553630	-6.550884	-5.036783
O	11.712058	-2.533796	-6.237376
O	-1.046401	5.831093	5.613464
O	6.934135	9.643627	0.319647
O	-2.072461	4.559516	3.466065
O	-0.394437	1.375816	8.110604
O	-1.941228	2.910991	6.619220
O	1.717471	8.026123	0.465618
O	10.676961	7.919233	0.428851
O	7.066613	10.140335	2.787894
O	3.854177	10.019167	4.386981
O	2.123761	9.112049	2.941751
O	1.134474	6.900158	6.444070
O	4.277680	-10.665940	1.466925
O	7.683302	-9.951733	-1.751067
O	6.177834	-7.753073	4.863967
O	4.822242	8.680445	-1.174735
O	8.787421	6.757818	-3.852561
O	11.428728	2.724326	-5.795957
O	12.226175	4.895312	-3.762761
O	13.454380	3.325935	-2.182846
O	10.350963	-7.063101	0.971606
O	10.675623	7.716816	-2.198431

O	13.971035	-1.481397	-2.929490
H	9.868781	7.987628	0.985034
H	7.913541	9.967561	3.215879
H	7.106196	-7.895861	5.090547
H	2.977432	-5.864565	8.050027
H	3.650187	9.785641	5.299878
H	13.472262	-4.488698	-1.263379
H	13.243733	2.355174	-2.155765
H	-0.072377	0.467413	7.891838
H	0.272092	7.253403	6.160879
H	10.618745	-7.204127	1.888195
H	13.891296	-2.446587	-2.797876
H	0.767721	-4.334417	8.266714
H	11.915465	5.762775	-4.029574
H	-2.666744	2.441842	6.190855
H	12.326678	0.676727	-4.880321
H	-1.758416	-1.774759	6.342115
H	0.158717	-9.069052	3.623090
H	-3.878756	0.141604	4.566883
H	-1.832299	5.228561	4.154426
H	1.822145	8.275345	-0.459373
H	-2.485033	-5.885905	5.045816
H	5.553427	9.242205	-0.814743
H	9.525590	7.273772	-3.432396
H	3.532028	-10.671972	2.072063
H	7.102196	-10.724265	-1.807031
H	10.046012	-7.357443	-4.909612
H	12.175235	-1.681548	-6.156825
H	12.025095	3.479345	-5.669410
H	11.593669	7.614183	-2.468450
H	-1.658597	5.266367	6.106761
H	1.761457	9.040164	2.042389
H	7.663747	9.265871	-0.187805
H	7.300197	10.485967	1.889754
H	2.962990	10.160187	3.958549
H	1.808456	7.231493	5.807347
H	-1.359496	1.326827	8.183286
H	-0.587005	-3.541531	8.293918
H	1.915187	-6.542915	7.127759
H	5.914735	-8.584487	4.401955
H	10.111954	-7.944163	0.606398
H	12.696012	-4.832530	-2.584445
H	14.086378	-1.324667	-3.883664
H	13.736317	3.529945	-3.087854
H	10.612672	8.632462	-0.229164

H	4.167436	-3.758306	1.348699
H	3.130433	1.281290	-1.207542
H	7.370093	-0.778629	-1.575046
Al	0.551262	-2.569960	6.519063
O	2.407322	-3.177767	6.524971
Al	2.316843	-4.823140	5.627030
O	0.728570	-1.650559	4.840703
Al	1.296960	0.071188	5.442798
O	-0.503813	0.749496	5.458174
Si	-1.677161	0.568843	4.313443
O	0.500384	-4.313990	5.741440
Si	-0.711953	-4.696489	4.673544
O	1.035257	-0.841976	7.161215
Si	-0.249037	-1.933713	3.532935
O	0.639101	-1.855682	2.175998
Si	2.085347	-1.460295	1.463664
O	2.535086	-2.873846	0.715454
O	-0.883785	-3.431067	3.639922
O	-1.411269	-0.790478	3.440865
O	-0.253245	-6.050782	3.861668
Si	1.021820	-7.085131	4.067945
O	1.786797	-7.139263	2.622314
Si	3.163979	-6.749763	1.848772
O	4.331315	-6.287549	2.964901
Al	6.140894	-6.841831	2.857124
O	8.045201	-7.183179	2.760880
Al	8.278939	-6.506860	0.957420
O	8.825808	-4.813672	1.562050
Al	8.942570	-3.825350	-0.069577
O	9.260075	-3.130079	-1.794415
Al	11.088899	-3.412778	-1.961221
O	1.947981	-6.579810	5.296076
O	4.201935	-5.160171	5.569936
Al	4.502571	-4.555650	3.753745
O	4.735083	-3.645998	2.127165
Al	5.082964	-1.875671	2.676428
O	5.451219	-1.203619	0.954126
Al	7.291206	-1.526886	0.830704
O	7.594190	-0.477302	-0.681821
Al	8.198291	1.144727	-0.018626
O	7.779836	0.188116	1.607953
O	2.612889	-4.258368	3.853793
O	4.855521	-2.791876	4.366599
O	6.365884	-5.151996	3.735961
O	6.970512	-2.263760	2.555279

Al	3.519091	0.453118	3.619661
O	3.258643	-1.228015	2.639642
O	5.286992	-0.273252	3.645548
O	9.130883	-2.159993	0.827538
O	7.075353	-3.205718	-0.147230
Si	6.134055	-3.367967	-1.509557
O	10.836002	-4.140223	-0.233156
O	8.509828	-5.592448	-0.688484
Si	7.648090	-5.970293	-2.065669
O	6.954508	-7.432908	-1.897171
Si	7.149804	-8.697678	-0.839659
O	8.222795	-8.191211	0.274440
O	11.426195	-2.435554	-3.529086
Al	12.059535	-0.768115	-2.882501
O	11.045631	-5.026214	-2.827355
Si	10.008063	-5.363219	-4.032796
O	11.826769	-1.831550	-1.287278
Si	10.644803	-2.580283	-4.991782
O	9.815345	-3.991095	-4.951931
O	9.568698	-1.316178	-5.069404
Si	9.300124	-0.000224	-4.144798
O	10.298725	-0.008007	-2.826242
Al	10.580307	1.461539	-1.659012
O	12.420232	0.764965	-1.800498
O	8.562509	-5.882905	-3.416272
O	6.431156	-4.871523	-2.161005
O	6.719113	-2.244337	-2.555194
O	1.641349	0.751442	3.702846
O	3.139504	-0.355816	5.309527
O	1.538292	1.725665	6.292847
Al	-0.232397	2.440075	6.381236
O	0.066485	3.405394	4.730337
Al	0.606027	5.065061	5.545144
O	3.783514	1.476962	2.087221
Al	4.354223	3.128697	2.711199
O	4.878347	3.898497	1.056403
Al	6.739247	3.488933	1.046052
O	8.513749	2.764791	0.978026
O	4.025026	2.174504	4.350683
O	2.628089	3.969655	2.646242
Al	2.885878	5.517876	3.748534
O	1.045220	5.886357	3.855192
O	4.720648	4.813947	3.694673
O	6.206989	2.584102	2.615096
Si	1.426844	3.890064	1.505296

O	1.385200	5.385609	0.799791
Si	2.470554	6.596981	0.794743
O	3.610392	6.360010	-0.375704
Si	5.027266	7.120319	-0.755969
O	5.664890	6.240791	-1.993311
Si	6.350234	4.756249	-1.888281
O	7.107540	4.661373	-0.424810
Al	7.746360	6.163991	0.594663
O	8.097868	7.645293	1.821297
Al	6.355325	8.521786	1.685696
O	1.730987	2.761687	0.360595
O	-0.025939	3.429194	2.064456
Si	-0.964238	3.374754	3.434113
O	0.467559	4.034031	7.132232
O	-1.690349	1.895047	3.358028
O	3.212188	6.671061	2.257903
Al	3.740764	8.317246	3.124829
O	5.606595	7.765248	3.269704
O	3.357373	7.187337	4.659899
O	2.474987	4.655125	5.359142
O	7.283516	5.009441	2.025057
O	6.454738	1.989329	-0.128254
Si	5.329030	1.975153	-1.403353
O	6.049934	7.020752	0.541606
O	8.429254	7.126649	-0.889450
Al	10.232048	6.450836	-0.949657
O	9.528677	5.548503	0.573489
O	4.600789	9.162561	1.677535
O	2.933519	-5.562809	0.742737
O	3.660366	-8.072193	1.039832
Si	4.901217	-9.143315	1.240500
O	5.788759	-8.600517	2.503233
O	5.744547	-9.210879	-0.161187
O	6.421860	-6.193397	1.125720
O	7.405598	4.550214	-3.112441
Si	8.898730	5.167556	-3.487045
O	5.237936	3.550496	-1.950592
O	9.410804	4.162295	-4.682296
Si	10.303094	2.778648	-4.616278
O	9.891704	5.015252	-2.170031
Al	11.630966	4.192423	-2.195239
O	9.405126	1.440698	-4.922456
O	10.948016	2.635939	-3.103643
O	7.720966	-0.081088	-3.685358
O	3.868192	1.598591	-0.652128

O	8.714567	1.937498	-1.669106
O	10.044112	0.581523	-0.064775
O	11.211773	2.979108	-0.772072
O	11.918897	5.602037	-0.969098
H	11.765084	-1.605613	-0.358172
H	8.478333	0.144651	2.267181
H	4.698178	2.113915	5.033837
H	-0.091027	4.420159	7.813796
H	11.168296	3.155084	0.167357
H	6.546810	5.207606	2.621060
H	4.066904	7.097891	5.305286
H	8.509769	-4.529784	2.423537
H	5.512547	-2.575333	5.032218
H	1.782334	-0.798976	7.766131
H	12.637625	5.694484	-0.343436
H	8.313062	7.250522	2.675381
H	9.526810	-2.266802	1.697984
H	6.106100	0.210775	3.522578
H	2.207002	1.966456	6.935553
H	9.498643	4.580110	0.512781
H	5.596060	6.800870	3.357273
H	10.965686	-5.094656	-0.150263
H	6.976059	-3.232107	2.586199
H	3.361968	-1.295358	5.223121
H	12.853168	0.564969	-0.964095
H	8.879519	2.665269	1.862588
H	5.027929	4.607781	4.585466
H	6.790633	-5.167839	4.598831
H	3.221854	-2.725036	6.747683
H	9.989632	-0.388816	-0.098489
H	6.238467	1.643615	2.380115
H	2.606751	3.692499	5.331177
H	8.055551	-8.141135	2.608463
H	4.354836	-6.114377	5.598434
H	7.994792	7.398951	-1.707776
H	4.194830	9.757790	1.043251
H	8.684051	-2.514788	-2.268723
H	4.988265	-0.461212	0.552176
H	1.362827	1.667471	3.567941
H	8.583922	2.896053	-1.714718
H	4.532201	4.734840	0.722178
H	0.607117	6.661972	3.489866
H	6.034781	-5.334639	0.905975
H	2.363561	-3.341641	3.671507
H	6.169732	-1.780832	-3.206162

H	2.053361	-3.144825	-0.084774
H	2.609412	2.761481	-0.052593
H	7.506206	0.644468	-3.076425
H	2.454746	-4.781557	1.055765
H	3.413582	1.299165	1.212126
Si	4.401489	0.760874	-4.046273
O	5.356405	0.838104	-2.734272
O	2.803088	0.476106	-3.780241
O	4.884895	-0.569195	-4.951947
O	4.583519	2.162350	-4.905920
H	5.732596	-0.468807	-5.401087
H	3.835997	2.387421	-5.470298
Si	3.521969	-3.122281	-2.598729
O	4.466744	-3.117124	-1.223158
O	2.505077	-1.839983	-2.386858
O	2.564279	-4.503080	-2.572173
O	4.211136	-3.143061	-4.095108
H	2.782922	-5.107454	-3.291564
H	4.420826	-2.276291	-4.500334
Si	0.430549	-0.250188	-0.477208
O	1.891349	-0.206768	0.318207
O	0.749486	0.155052	-2.062900
O	-0.549032	0.853949	0.256984
O	-0.330332	-1.740696	-0.475868
H	-1.466113	0.867042	-0.061585
H	-0.305161	-2.293461	-1.312671
Si	1.699625	-0.617681	-3.177585
C	0.646750	-1.257450	-4.574724
H	1.178924	-2.096999	-5.064973
H	0.641405	-0.442668	-5.319526
C	-0.796976	-1.636841	-4.219511
H	-1.187961	-0.964064	-3.430489
H	-1.445277	-1.459181	-5.095047
C	-1.035612	-3.076551	-3.804852
H	-0.781862	-3.761468	-4.644364
H	-2.120299	-3.199431	-3.622440
N	-0.345985	-3.494454	-2.578074
C	-0.797107	-4.829928	-2.177560
H	0.662876	-3.570227	-2.758111
C	-0.207073	-5.310173	-0.869744
H	-0.561014	-5.578898	-2.963447
H	-1.901461	-4.803769	-2.089301
H	-0.497609	-6.363828	-0.729682
H	0.894921	-5.278993	-0.939914
N	-0.699653	-4.548223	0.291045

H	-0.379023	-4.985985	1.155504
H	-0.306966	-3.598018	0.282663
Au	-4.647655	-1.961535	1.439452
Au	-2.920705	-3.860346	0.290212
Au	-8.015794	2.064169	3.376934
Au	-6.283363	0.022686	2.506286
Au	-6.476664	2.757115	1.161585
Au	-4.968835	3.283716	-1.088204
Au	-3.478982	3.655229	-3.428876
Au	-3.145038	1.157055	-2.230211
Au	-2.987374	-1.321095	-0.996309
Au	-4.628197	0.713777	0.245423
Au	-5.292153	-3.262974	-1.065021
Au	-8.833116	0.872363	0.991357
Au	-5.649828	1.912958	-3.730627
Au	-7.150712	-1.307995	0.076304
Au	-5.334521	-0.743758	-2.555956
Au	-7.311631	1.591246	-1.370209
Au	-7.769836	0.153478	-3.857654
Au	-7.583022	-2.564181	-2.495675
Au	-9.457635	-0.358224	-1.405208
Au	-9.896345	-1.642303	-3.836189

Optimized geometries of the Au_n/fHNT model systems (without link atoms)

Au/fHNT, ONIOM model system

```
  43
Si     1.834490     1.508570     3.838650
 O     1.611110     1.694050     2.203250
 O     1.271730     0.095150     4.465900
 O     0.911820     2.768690     4.483980
 O     3.413000     1.651950     4.318120
 H     1.349930     3.203540     5.224910
 H     3.935540     2.273330     3.799680
Si    -2.336070     1.741990     3.236640
 O    -2.627520     1.934090     1.596450
 O    -1.135570     0.633020     3.459840
 O    -3.668000     0.962950     3.896610
 O    -2.000710     3.102070     4.109200
 H    -4.533240     1.307960     3.650220
 H    -1.061140     3.132820     4.372570
Si    -1.709400    -2.495630     3.210200
 O    -1.231750    -2.032090     1.670550
 O    -0.629830    -1.831980     4.300190
 O    -1.672380    -4.146980     3.354800
 O    -3.250000    -2.107010     3.673690
 H    -1.006940    -4.573580     2.805340
 H    -3.402800    -1.174270     3.904220
Si    -0.365800    -0.226050     4.663080
 C    -0.744980     0.215020     6.424000
 H    -0.303820     1.221780     6.542800
 H    -0.077730    -0.449150     7.012640
 C    -2.148540     0.201960     7.025950
 H    -2.045410     0.381640     8.111740
 H    -2.742120     1.037680     6.613780
 C    -2.947440    -1.072470     6.814340
 H    -3.392710    -1.075280     5.799680
 H    -3.799640    -1.087000     7.517910
 N    -2.180110    -2.292570     7.031610
 C    -2.979890    -3.496920     6.814200
 H    -1.395740    -2.324780     6.372970
 C    -2.124920    -4.745620     6.749410
 H    -3.554390    -3.415160     5.867890
 H    -3.714150    -3.572400     7.640610
 H    -2.784510    -5.585900     6.443540
```

H	-1.395890	-4.625720	5.926100
N	-1.399650	-4.965880	7.988190
H	-2.046630	-5.188950	8.746180
H	-0.764220	-5.757970	7.905350
Au	-1.099050	-2.282460	9.274090

Au_2/fHNT, ONIOM model system

44

Si	-2.821310	0.042480	2.969980
O	-2.235900	-0.168700	1.431830
O	-1.683530	0.626030	4.072180
O	-3.141210	-1.529940	3.481150
O	-4.116830	1.041430	2.968960
H	-4.053720	-1.652440	3.804530
H	-4.309410	1.560290	3.786140
Si	0.188620	-2.903660	3.087800
O	0.752320	-3.205330	1.538930
O	0.016430	-1.276240	3.306000
O	1.439990	-3.273880	4.145870
O	-1.176630	-3.677370	3.587850
H	1.925390	-4.086720	3.967870
H	-1.947890	-3.077030	3.644320
Si	2.492690	0.595000	3.898530
O	2.317070	0.637700	2.230190
O	0.964460	0.917470	4.498470
O	3.559360	1.767520	4.376620
O	3.055070	-0.787370	4.603200
H	3.600320	2.509950	3.765550
H	2.486360	-1.576730	4.588760
Si	-0.279010	-0.182210	4.521320
C	-0.352030	-0.977630	6.213920
H	0.124320	-1.964270	6.055050
H	0.371580	-0.414500	6.832670
C	-1.657850	-1.173560	6.987040
H	-2.427940	-1.658000	6.356260
H	-1.467940	-1.877760	7.814140
C	-2.234140	0.093540	7.601880
H	-1.412750	0.708720	8.026270
H	-2.911480	-0.155350	8.436900
N	-3.003580	0.900330	6.635660
C	-3.316970	2.250410	7.124990
H	-2.448420	0.997330	5.772270

C	-3.563110	3.233600	5.998400
H	-4.190000	2.192470	7.801410
H	-2.472110	2.636590	7.728780
H	-3.725740	4.227220	6.460630
H	-2.641460	3.315680	5.392080
N	-4.646990	2.824680	5.108960
H	-5.497660	2.632310	5.644630
H	-4.881860	3.591310	4.476420
Au	-6.706950	-1.714690	5.003900
Au	-4.789150	-0.264330	5.955500

Au_3/fHNT, ONIOM model system

45

Si	0.271040	2.653740	3.482570
O	0.403640	2.395460	1.838180
O	0.478740	1.315340	4.432290
O	-1.302840	3.206670	3.670860
O	1.308110	3.793700	4.077040
H	-1.929970	2.581720	3.278820
H	1.302240	4.622840	3.587150
Si	-2.748170	-0.182100	2.270110
O	-2.650170	-0.434440	0.624210
O	-1.239880	0.054110	2.886970
O	-3.280010	-1.557890	3.052250
O	-3.686200	1.131370	2.675300
H	-4.241320	-1.637270	3.188570
H	-3.717740	1.353040	3.653650
Si	0.395180	-2.774650	3.493620
O	0.858640	-2.308940	1.945610
O	0.472430	-1.379770	4.409620
O	1.455840	-3.906450	4.068580
O	-1.090890	-3.458080	3.692480
H	2.337340	-3.829280	3.690720
H	-1.866360	-2.872420	3.656880
Si	-0.498960	-0.043000	4.388000
C	-1.741180	-0.104460	5.779750
H	-2.665610	-0.551310	5.360220
H	-1.347180	-0.880080	6.458800
C	-2.060960	1.177700	6.567070
H	-1.900240	0.986670	7.640300
H	-1.354430	1.990850	6.312860
C	-3.479850	1.701890	6.421200

```
 H    -4.214990    0.875670    6.516700
 H    -3.689260    2.410450    7.250750
 N    -3.708810    2.369230    5.133930
 C    -4.926330    3.177000    5.167500
 H    -2.912360    2.999910    4.978470
 C    -5.187690    3.942580    3.885190
 H    -5.783900    2.508280    5.380150
 H    -4.887520    3.911420    6.000140
 H    -5.973830    4.687340    4.085000
 H    -4.275750    4.500680    3.592420
 N    -5.644360    3.100960    2.758870
 H    -5.970740    3.697740    1.997320
 H    -4.854600    2.549840    2.391570
Au    -7.045150    1.463150    3.218800
Au    -8.668660   -0.554020    3.712960
Au    -6.040390   -0.741900    4.751870
```

Au_4/fHNT, ONIOM model system

```
   46
Si    -2.800660    0.723290    1.939110
 O    -2.060490    0.485010    0.473840
 O    -1.819150    1.010370    3.231710
 O    -3.647800   -0.696960    2.125540
 O    -3.808260    2.082030    1.928660
 H    -4.458380   -0.675570    2.671830
 H    -4.482020    2.069320    1.235630
Si    -0.768890   -2.890720    2.338190
 O     0.001120   -3.229270    0.885710
 O    -0.631780   -1.288880    2.712030
 O     0.130370   -3.603750    3.561480
 O    -2.339800   -3.384310    2.457020
 H     0.421590   -4.508650    3.405870
 H    -2.973750   -2.642340    2.445190
Si     2.153850   -0.124250    3.826950
 O     2.310550    0.018350    2.161140
 O     0.675110    0.572530    4.161700
 O     3.377580    0.697180    4.580490
 O     2.203640   -1.630470    4.501510
 H     3.669980    1.472600    4.091030
 H     1.462550   -2.236500    4.327420
Si    -0.798550   -0.140180    3.910510
 C    -1.474720   -0.772620    5.515110
```

H	-0.670400	-1.341960	6.019560
H	-1.636120	0.146140	6.112850
C	-2.757150	-1.584700	5.389820
H	-3.514820	-0.997730	4.835490
H	-2.574670	-2.495780	4.792300
C	-3.373670	-2.005270	6.710450
H	-2.706740	-2.730430	7.232560
H	-4.319190	-2.538640	6.499260
N	-3.696130	-0.864570	7.556840
C	-4.440380	-1.211240	8.744960
H	-2.836460	-0.382080	7.825920
C	-4.842450	0.018650	9.534980
H	-5.352850	-1.756420	8.429630
H	-3.894480	-1.902170	9.426630
H	-5.212690	-0.287180	10.530300
H	-3.959990	0.661010	9.701190
N	-5.854540	0.825920	8.827470
H	-6.735970	0.306750	8.789440
H	-6.059130	1.676050	9.355510
Au	-6.471700	-2.743930	3.561640
Au	-4.771080	2.089370	4.232070
Au	-6.062660	-0.335120	4.507890
Au	-5.393480	1.350960	6.676860

Au_5/fHNT, ONIOM model system

47

Si	-1.604750	0.720850	-3.815230
O	-2.104410	0.967930	-2.248270
O	-0.355900	-0.324380	-4.073060
O	-1.081710	2.268310	-4.260940
O	-2.822140	0.220030	-4.821490
H	-1.283700	2.463560	-5.183490
H	-3.688660	0.582890	-4.608140
Si	1.637550	2.511000	-1.843810
O	1.208360	2.791070	-0.253990
O	1.036560	1.041250	-2.305800
O	3.305570	2.425780	-1.915590
O	1.183050	3.642230	-2.984810
H	3.570040	2.488920	-2.872230
H	0.429570	3.325190	-3.521630
Si	2.535520	-1.710660	-1.378930
O	1.408490	-1.418880	-0.176190

```
 O     1.705520   -1.521510   -2.802490
 O     3.126720   -3.261520   -1.262810
 O     3.904000   -0.770380   -1.356890
 H     2.498180   -3.894080   -0.896980
 H     3.835540    0.139430   -1.690530
Si     1.209850   -0.106880   -3.512380
 C     2.461280    0.392170   -4.778770
 H     3.383900    0.533410   -4.175030
 H     2.694960   -0.489620   -5.402380
 C     2.182930    1.636800   -5.627810
 H     1.813660    1.330590   -6.620230
 H     1.373240    2.251100   -5.191470
 C     3.407630    2.518240   -5.812750
 H     4.275550    1.890900   -6.099400
 H     3.242790    3.236730   -6.643970
 N     3.753150    3.221140   -4.575360
 C     4.935150    4.060170   -4.723710
 H     2.954280    3.806530   -4.302230
 C     5.445170    4.589670   -3.397440
 H     5.729950    3.445960   -5.188530
 H     4.755940    4.918240   -5.406780
 H     6.278820    5.280040   -3.600920
 H     4.652370    5.182730   -2.900560
 N     5.926560    3.525090   -2.493220
 H     6.677780    3.878580   -1.900830
 H     5.167000    3.244630   -1.860880
Au     6.384040    1.459390   -3.442570
Au     6.740650    0.050940   -5.834520
Au     5.544080   -2.482830   -6.099880
Au     5.672690   -1.160450   -3.558930
Au     4.396280   -3.568980   -3.870950
```

Au_6/fHNT, ONIOM model system

```
   48
Si     2.554590   -0.340060   -1.560610
 O     1.616250   -0.440730   -0.189400
 O     1.815790    0.348620   -2.875250
 O     2.859150   -1.954800   -1.917400
 O     3.931770    0.549680   -1.319890
 H     3.618250   -2.051770   -2.525310
 H     4.556420    0.186080   -0.679360
Si    -0.604140   -2.985410   -2.406580
```

O	-1.551020	-3.103630	-1.024380
O	-0.193280	-1.421730	-2.737370
O	-1.620230	-3.379340	-3.684850
O	0.740360	-3.942670	-2.467080
H	-2.310000	-4.019100	-3.476800
H	1.562140	-3.427270	-2.343080
Si	-2.225660	0.741550	-3.784340
O	-2.461630	0.814160	-2.124230
O	-0.578020	0.915650	-3.980370
O	-3.044010	1.972280	-4.529550
O	-2.725920	-0.610540	-4.595820
H	-3.059010	2.785070	-4.014110
H	-2.253140	-1.441510	-4.426070
Si	0.532580	-0.291920	-3.734830
C	1.073850	-1.005150	-5.376910
H	0.346920	-0.654870	-6.133200
H	2.047230	-0.528020	-5.615540
C	1.174560	-2.525720	-5.387200
H	1.868910	-2.869260	-4.596090
H	0.191530	-2.962230	-5.139820
C	1.609380	-3.151470	-6.695730
H	0.921180	-2.851070	-7.514660
H	1.522530	-4.247550	-6.599510
N	3.005070	-2.871330	-7.053250
C	3.522970	-3.754250	-8.095930
H	3.086850	-1.899560	-7.362560
C	5.037660	-3.841150	-8.082430
H	3.112850	-4.763520	-7.905250
H	3.174650	-3.467520	-9.107700
H	5.366100	-4.449140	-8.947250
H	5.474580	-2.834870	-8.212280
N	5.499080	-4.367960	-6.798770
H	5.217420	-5.343500	-6.684200
H	6.516520	-4.346040	-6.732750
Au	6.576620	1.080430	-2.808150
Au	7.848850	-0.713210	-1.233200
Au	5.054570	2.676280	-4.381920
Au	4.584330	-0.000910	-4.603910
Au	6.219900	-1.817290	-3.151230
Au	4.649810	-2.667110	-5.182880

Au_7/fHNT, ONIOM model system

49

Si	-2.092530	1.068860	1.796800
O	-1.290070	0.767940	0.373220
O	-1.132360	1.151260	3.147590
O	-3.073300	-0.286550	1.928220
O	-2.932010	2.503800	1.796900
H	-3.916900	-0.127670	2.388410
H	-3.739720	2.508890	1.259270
Si	-0.514690	-2.817110	2.085640
O	0.280490	-3.172370	0.652600
O	-0.196510	-1.275380	2.575080
O	0.220240	-3.722220	3.294790
O	-2.140650	-3.112270	2.109380
H	0.458040	-4.625940	3.060580
H	-2.670090	-2.291100	2.106740
Si	2.628710	-0.509220	3.852350
O	2.893780	-0.300800	2.207110
O	1.232920	0.351100	4.166570
O	3.904300	0.108050	4.708440
O	2.455940	-2.043140	4.440950
H	4.314100	0.866630	4.280540
H	1.655630	-2.539670	4.197580
Si	-0.304340	-0.148890	3.800480
C	-1.137020	-0.790870	5.338350
H	-0.350750	-1.260790	5.959610
H	-1.469400	0.119150	5.877440
C	-2.291710	-1.755950	5.093870
H	-3.041110	-1.291160	4.423390
H	-1.925540	-2.654220	4.569770
C	-3.001300	-2.224320	6.347680
H	-2.279450	-2.727990	7.028510
H	-3.763620	-2.976940	6.074660
N	-3.701180	-1.142870	7.040610
C	-4.422370	-1.584950	8.223390
H	-3.027750	-0.419680	7.306760
C	-5.338870	-0.498330	8.752190
H	-5.030360	-2.463200	7.931640
H	-3.744130	-1.928390	9.031550
H	-5.753800	-0.814520	9.727970
H	-4.752240	0.419980	8.935210
N	-6.372530	-0.180000	7.768780
H	-7.059800	-0.933600	7.708680
H	-6.886570	0.662110	8.025950
Au	-5.948000	1.271820	3.120760
Au	-5.304280	0.129510	5.554260

Au	-6.532730	2.246540	0.603880
Au	-6.427520	-0.438760	0.849920
Au	-5.961510	-1.618950	3.450980
Au	-6.086830	-3.106570	1.128210
Au	-3.871210	2.314510	4.629280

Au_8/fHNT, ONIOM model system

50			
Si	2.225110	-0.003840	-1.309200
O	1.221370	0.126160	-0.026470
O	1.521090	0.473540	-2.735110
O	2.574530	-1.639240	-1.436260
O	3.600800	0.907220	-1.183160
H	3.526550	-1.847740	-1.457380
H	4.175130	0.742110	-0.421190
Si	-0.564370	-3.021930	-2.134930
O	-1.603330	-3.188590	-0.831930
O	-0.320390	-1.420810	-2.428810
O	-1.380840	-3.522240	-3.513310
O	0.871640	-3.833040	-2.042930
H	-1.898620	-4.330330	-3.432180
H	1.612700	-3.213560	-1.877860
Si	-2.527850	0.352190	-3.919110
O	-2.955590	0.507010	-2.303170
O	-0.895970	0.700910	-3.962660
O	-3.385100	1.437590	-4.829700
O	-2.791000	-1.093520	-4.676620
H	-3.591000	2.249030	-4.354810
H	-2.244220	-1.853750	-4.415580
Si	0.316320	-0.347590	-3.534000
C	0.967730	-1.159770	-5.072630
H	0.098450	-1.548500	-5.636980
H	1.363900	-0.304850	-5.655830
C	2.024050	-2.237420	-4.857380
H	2.861000	-1.826800	-4.256690
H	1.603080	-3.071630	-4.269460
C	2.582790	-2.807030	-6.148480
H	1.773210	-3.310050	-6.721870
H	3.349880	-3.571110	-5.926860
N	3.215500	-1.770100	-6.967120
C	3.664270	-2.223600	-8.274970
H	2.540340	-1.012660	-7.102620

```
 C     4.289530    -1.075900    -9.046500
 H     4.406850    -3.029760    -8.120530
 H     2.838070    -2.661470    -8.872720
 H     4.529430    -1.410700   -10.072770
 H     3.545050    -0.265970    -9.146890
 N     5.449130    -0.533220    -8.337640
 H     6.277050    -1.108880    -8.501400
 H     5.683930     0.403040    -8.665880
Au     5.499420    -1.739030    -3.391690
Au     6.889340     0.532890    -4.239370
Au     6.071050    -1.434100    -0.609270
Au     7.333970     1.002930    -1.459070
Au     8.669710     2.402840    -3.337500
Au     6.656840     0.544510     1.133570
Au     5.223840    -3.807650    -1.597440
Au     5.167210    -0.746020    -5.881340
```

Au_9/fHNT, ONIOM model system

```
   51
Si     1.871240    -0.231290     1.791220
 O     0.989040    -0.322120     0.423370
 O     1.011080    -0.560230     3.172690
 O     2.416980     1.352230     1.882410
 O     3.133490    -1.309700     1.791800
 H     3.351650     1.431450     2.145800
 H     3.649560    -1.342620     0.975280
Si    -0.667130     3.062580     2.185010
 O    -1.544820     3.249040     0.771480
 O    -0.599050     1.468660     2.594310
 O    -1.577290     3.712930     3.438240
 O     0.836630     3.742680     2.210560
 H    -2.023270     4.546440     3.252450
 H     1.540580     3.065440     2.155390
Si    -3.131020    -0.020480     3.869060
 O    -3.404990    -0.213610     2.224840
 O    -1.556970    -0.536220     4.096570
 O    -4.187430    -0.961890     4.727680
 O    -3.304660     1.481410     4.536520
 H    -4.433760    -1.771070     4.268400
 H    -2.662160     2.171800     4.299800
Si    -0.209880     0.383810     3.800510
 C     0.345610     1.196880     5.380710
```

```
 H    -0.553690     1.447360     5.973140
 H     0.870640     0.386450     5.924220
 C     1.235820     2.417600     5.175160
 H     2.037840     2.187530     4.444560
 H     0.646710     3.238000     4.732820
 C     1.878510     2.946400     6.440280
 H     1.098420     3.219920     7.183020
 H     2.449450     3.864810     6.213700
 N     2.823600     1.990530     7.030320
 C     3.441160     2.477590     8.261510
 H     2.322840     1.122230     7.239480
 C     4.537130     1.546430     8.747190
 H     3.871500     3.473590     8.044730
 H     2.686290     2.623360     9.060020
 H     4.828520     1.842480     9.773650
 H     4.132150     0.520190     8.819510
 N     5.646280     1.527500     7.804450
 H     6.224360     2.363920     7.893850
 H     6.257630     0.724880     7.948700
Au     4.487250     1.319580     5.452230
Au     5.669270     0.511930     3.077110
Au     6.681890    -0.436510     0.300800
Au     4.993920     3.201210     3.348330
Au     5.107360    -1.427580     4.976990
Au     6.097860     2.242010     0.942570
Au     6.129290    -2.161850     2.473270
Au     7.186480     1.586550    -1.478390
Au     6.859320    -3.155290     0.036560
```

Au_{10}/fHNT, 2D cluster, ONIOM model system

```
   52
Si     1.663090     2.215550     1.303060
 O     0.543820     2.167420     0.122820
 O     2.039820     0.712160     1.910990
 O     0.980180     3.114320     2.556730
 O     3.040410     3.021560     0.886660
 H     1.479460     3.926360     2.709740
 H     3.829250     2.482740     0.701020
Si    -1.757060     0.965730     3.387100
 O    -3.106040     1.051580     2.402110
 O    -0.516000     0.351930     2.497490
 O    -2.056630    -0.215920     4.543380
```

O	-1.274400	2.338750	4.166680
H	-2.947450	-0.227010	4.910370
H	-0.479350	2.730870	3.753320
Si	-0.287090	-2.828900	2.014660
O	-1.133850	-2.274710	0.676840
O	1.019720	-1.798020	2.178140
O	0.279920	-4.361110	1.739420
O	-1.076070	-2.918640	3.463750
H	0.421830	-4.557080	0.807290
H	-1.265300	-2.080540	3.918490
Si	1.010130	-0.255320	2.796370
C	1.381140	-0.252050	4.617830
H	0.398390	-0.371710	5.119090
H	1.962840	-1.169380	4.833520
C	2.103730	0.992180	5.131170
H	3.167180	0.948810	4.820870
H	1.683920	1.902500	4.668070
C	2.016890	1.151360	6.637910
H	0.966650	1.398590	6.920080
H	2.641350	2.005950	6.958440
N	2.494240	-0.028930	7.342690
C	2.465980	0.081860	8.784480
H	1.953470	-0.846020	7.049680
C	3.149400	-1.100540	9.445400
H	2.996360	1.013390	9.065340
H	1.438280	0.179910	9.197920
H	2.962690	-1.070720	10.534830
H	2.709240	-2.040370	9.066090
N	4.586560	-1.130000	9.139760
H	5.060070	-0.350770	9.602260
H	5.016120	-1.980660	9.505480
Au	4.998020	-0.990830	6.832480
Au	5.231450	-0.887890	4.079570
Au	4.925060	-0.401500	1.263230
Au	6.619980	0.970750	5.610840
Au	4.079060	-3.152770	5.194060
Au	6.272510	1.461030	2.867980
Au	3.720940	-2.654440	2.449140
Au	6.170460	1.784920	0.093690
Au	3.659100	-2.255630	-0.326090
Au	4.678180	0.058910	-1.531020

Au_{10}/fHNT, 3D cluster, ONIOM model system

52

Si	0.355680	2.483570	-2.986560
O	1.055230	2.419910	-1.528520
O	-0.507920	1.110180	-3.389540
O	1.578650	2.598410	-4.133800
O	-0.657810	3.784540	-3.100460
H	1.744480	3.507500	-4.412870
H	-1.491170	3.626990	-3.565580
Si	3.241480	-0.526220	-3.519270
O	4.216750	-0.619170	-2.166370
O	1.682390	-0.307430	-3.029700
O	3.189930	-2.049700	-4.225500
O	3.632560	0.596270	-4.661780
H	4.037830	-2.481830	-4.377940
H	2.992530	1.334010	-4.668700
Si	-0.038470	-2.848820	-1.965880
O	0.664500	-2.236380	-0.582760
O	-0.679710	-1.529560	-2.774780
O	-1.240670	-3.944240	-1.654630
O	0.922950	-3.667920	-3.049820
H	-2.060240	-3.540880	-1.322650
H	1.614510	-3.166570	-3.518300
Si	0.152540	-0.409160	-3.671870
C	0.166010	-0.795300	-5.487700
H	0.924250	-1.585480	-5.655390
H	0.594430	0.109180	-5.957010
C	-1.178510	-1.171480	-6.140630
H	-2.038930	-0.834440	-5.524240
H	-1.279420	-0.641740	-7.101490
C	-1.326110	-2.657300	-6.424490
H	-0.473440	-2.985490	-7.062980
H	-2.246140	-2.825960	-7.015320
N	-1.392830	-3.453210	-5.205280
C	-1.133100	-4.876610	-5.364440
H	-0.737310	-3.087670	-4.516580
C	-2.398730	-5.691310	-5.558730
H	-0.617340	-5.224810	-4.451180
H	-0.446150	-5.074120	-6.213570
H	-2.959180	-5.324920	-6.437500
H	-2.119500	-6.742450	-5.765150
N	-3.264250	-5.558580	-4.385890
H	-4.123520	-6.097450	-4.490800
H	-2.790270	-5.911730	-3.550590
Au	-3.577210	-1.367730	-1.808160
Au	-4.576200	-0.587810	-4.465830

Au	-6.158700	-2.314980	-2.920410
Au	-4.794770	0.919290	-0.659720
Au	-5.799210	1.661690	-3.327980
Au	-7.523780	-0.114150	-1.875490
Au	-7.061940	2.566430	-0.936740
Au	-3.744450	-3.225830	-4.036820
Au	-3.056600	1.306110	-2.992670
Au	-5.934330	-1.688310	-0.154470

Au_{11}/fHNT, 2D cluster, ONIOM model system

53

Si	-1.529530	0.553780	1.657680
O	-0.615680	0.552980	0.308170
O	-0.674030	0.831850	3.051820
O	-2.213350	-0.974890	1.754790
O	-2.701820	1.731060	1.628860
H	-3.169230	-0.961360	1.943080
H	-3.188560	1.814020	0.798440
Si	0.706990	-2.936620	2.167770
O	1.595970	-3.225060	0.778130
O	0.765670	-1.331250	2.533550
O	1.531260	-3.625810	3.457260
O	-0.850580	-3.484580	2.178940
H	1.888930	-4.508580	3.313450
H	-1.494940	-2.753320	2.091300
Si	3.391520	-0.045810	3.861500
O	3.716560	0.094480	2.221380
O	1.861370	0.603010	4.040150
O	4.502020	0.821440	4.729940
O	3.423890	-1.544200	4.557020
H	4.828230	1.596350	4.261450
H	2.732720	-2.183950	4.314400
Si	0.449200	-0.203070	3.719280
C	-0.215260	-0.924380	5.296220
H	0.619040	-1.391760	5.850980
H	-0.514870	-0.028570	5.875510
C	-1.377690	-1.889570	5.099120
H	-2.121030	-1.451100	4.401750
H	-1.020260	-2.816060	4.617140
C	-2.092780	-2.268740	6.380000
H	-1.386320	-2.759060	7.084740
H	-2.891590	-3.001460	6.161110

```
 N    -2.729560    -1.113690     7.019460
 C    -3.413790    -1.443460     8.262760
 H    -2.013230    -0.409630     7.215950
 C    -4.117710    -0.232140     8.845680
 H    -4.155640    -2.233650     8.036620
 H    -2.718250    -1.867250     9.015950
 H    -4.487850    -0.484100     9.857840
 H    -3.384050     0.584050     8.974520
 N    -5.165430     0.241230     7.947940
 H    -5.994100    -0.353020     8.004870
 H    -5.468120     1.186230     8.181200
Au    -6.499750    -3.528020    -0.601710
Au    -5.813140    -1.411690     1.095800
Au    -5.263740     0.614890     2.949110
Au    -5.172060    -4.085180     1.766660
Au    -6.904220    -0.906310    -1.445470
Au    -4.879920    -2.085530     3.694050
Au    -6.264470     1.212270     0.275000
Au    -4.348740     0.006030     5.523810
Au    -5.742260     3.309270     2.165950
Au    -4.888550     2.708980     4.720000
Au    -6.456780     3.874150    -0.395550
```

Au_{11}/fHNT, 3D cluster, ONIOM model system

```
   53
FINAL HEAT OF FORMATION =     0.000000
Si     0.428650     2.469450    -2.901770
 O     1.201420     2.408180    -1.479860
 O    -0.472690     1.099980    -3.227200
 O     1.599810     2.546620    -4.106830
 O    -0.559570     3.788460    -3.011630
 H     1.691800     3.435820    -4.471380
 H    -1.454150     3.611680    -3.345300
Si     3.245860    -0.583550    -3.549720
 O     4.287580    -0.675140    -2.247350
 O     1.715800    -0.355120    -2.978710
 O     3.147720    -2.111980    -4.242110
 O     3.587250     0.523840    -4.721360
 H     3.983840    -2.552240    -4.431810
 H     2.966250     1.277630    -4.694370
Si     0.031620    -2.852100    -1.826980
 O     0.795910    -2.235930    -0.477970
```

O	-0.643980	-1.539050	-2.621750
O	-1.162970	-3.936070	-1.447150
O	0.925420	-3.690190	-2.950150
H	-1.870110	-3.567660	-0.896270
H	1.593420	-3.198580	-3.462230
Si	0.156620	-0.416740	-3.554930
C	0.108450	-0.787410	-5.374590
H	0.870920	-1.566520	-5.573590
H	0.519430	0.130870	-5.833420
C	-1.240760	-1.161750	-6.016180
H	-2.096000	-0.817230	-5.398270
H	-1.342670	-0.639740	-6.981210
C	-1.393890	-2.649020	-6.287620
H	-0.544290	-2.988840	-6.923520
H	-2.316490	-2.822230	-6.872730
N	-1.460490	-3.430480	-5.058040
C	-1.217140	-4.859910	-5.198500
H	-0.798210	-3.060500	-4.378810
C	-2.495730	-5.664560	-5.342490
H	-0.678250	-5.196970	-4.294610
H	-0.556070	-5.076330	-6.062740
H	-3.070220	-5.315490	-6.219330
H	-2.233420	-6.724450	-5.524690
N	-3.333460	-5.484750	-4.156310
H	-4.205550	-6.008130	-4.227550
H	-2.849850	-5.819950	-3.319280
Au	-3.305280	-1.243890	-1.708370
Au	-4.662700	-0.515470	-4.203090
Au	-6.046240	-2.306530	-2.517060
Au	-4.422770	0.852950	-0.318710
Au	-5.801780	1.733390	-2.758590
Au	-7.264250	-0.151260	-1.049060
Au	-6.698100	2.490630	-0.134240
Au	-3.756900	-3.129120	-3.897320
Au	-7.589510	-0.171370	-3.912500
Au	-3.071920	1.532880	-3.034030
Au	-5.410570	-1.865210	0.198220

Au_{12}/fHNT, 2D cluster, ONIOM model system

54

Si	1.325130	-0.669050	1.666900
O	0.425800	-0.700060	0.309000

O	0.446120	-0.844460	3.063470
O	2.064800	0.833150	1.683810
O	2.449960	-1.892790	1.717870
H	2.928560	0.810330	2.137790
H	2.997290	-1.980090	0.926910
Si	-0.725480	2.966710	1.978380
O	-1.596050	3.178220	0.562520
O	-0.860840	1.408540	2.498110
O	-1.525500	3.818890	3.184000
O	0.853710	3.456990	1.941370
H	-1.882700	4.676170	2.927390
H	1.472110	2.702940	1.868880
Si	-3.555730	0.276970	3.802920
O	-3.877470	0.065100	2.169850
O	-2.062940	-0.438220	4.028750
O	-4.716590	-0.480170	4.707210
O	-3.514550	1.811160	4.417780
H	-5.072050	-1.268940	4.285410
H	-2.788990	2.398960	4.150160
Si	-0.605430	0.280810	3.702920
C	0.112120	1.013000	5.262720
H	-0.707010	1.165090	5.988520
H	0.755460	0.206500	5.667430
C	0.891920	2.302640	5.029570
H	1.580550	2.183700	4.169720
H	0.193320	3.108640	4.746710
C	1.706300	2.785010	6.211160
H	1.050980	2.963980	7.091100
H	2.176610	3.754640	5.960010
N	2.789420	1.861390	6.556960
C	3.628940	2.345290	7.648680
H	2.380610	0.964210	6.833710
C	4.795750	1.413310	7.916720
H	4.011640	3.342440	7.356450
H	3.042590	2.490490	8.579000
H	5.281350	1.712080	8.865970
H	4.411430	0.387780	8.071230
N	5.705340	1.384950	6.779330
H	6.289830	2.221340	6.751660
H	6.333030	0.582350	6.816310
Au	3.961010	3.771580	3.347960
Au	5.305940	1.685240	2.052620
Au	6.279950	-0.402750	0.480500
Au	5.200610	4.256920	0.930500
Au	4.205800	1.206390	4.644410

Au	6.392310	2.247740	-0.548630
Au	5.108400	-0.915600	2.953420
Au	7.280910	0.082240	-2.099120
Au	6.172120	-3.101450	1.397710
Au	7.204790	-2.499600	-1.140890
Au	4.959130	-3.568300	3.842640
Au	3.671260	-1.582590	5.258010

Au_{12}/fHNT, 3D cluster, ONIOM model system

54			
Si	0.061440	2.576300	-2.434190
O	0.877580	2.417400	-1.040100
O	-0.577690	1.159650	-3.024720
O	1.173080	3.038180	-3.616000
O	-1.117030	3.724130	-2.283490
H	1.223380	3.995420	-3.728910
H	-1.928680	3.548570	-2.794740
Si	3.332600	0.146830	-3.579000
O	4.409650	0.049710	-2.307300
O	1.814270	-0.015420	-2.959310
O	3.483200	-1.252340	-4.497780
O	3.418160	1.463240	-4.565520
H	4.373130	-1.493520	-4.777760
H	2.683920	2.089080	-4.404510
Si	0.583320	-2.807490	-2.165680
O	1.281670	-2.315600	-0.735930
O	-0.328250	-1.519590	-2.736410
O	-0.435800	-4.098610	-1.907580
O	1.526740	-3.330240	-3.423920
H	-0.953210	-4.014530	-1.095960
H	2.103370	-2.681190	-3.868250
Si	0.265450	-0.167450	-3.542400
C	0.237100	-0.304670	-5.396700
H	1.060460	-0.976740	-5.709440
H	0.567660	0.704030	-5.709010
C	-1.080010	-0.675510	-6.100860
H	-1.958130	-0.400520	-5.480850
H	-1.175180	-0.089670	-7.029600
C	-1.171830	-2.143330	-6.479720
H	-0.331560	-2.391000	-7.167470
H	-2.107990	-2.327230	-7.038590
N	-1.149030	-3.009800	-5.306560

```
 C    -0.891090    -4.427500    -5.553950
 H    -0.440760    -2.670630    -4.658900
 C    -2.109390    -5.297710    -5.306880
 H    -0.080510    -4.753660    -4.875970
 H    -0.526690    -4.590670    -6.585960
 H    -2.917580    -5.037360    -6.014400
 H    -1.835130    -6.354150    -5.491980
 N    -2.598980    -5.066800    -3.948450
 H    -3.361300    -5.697820    -3.705370
 H    -1.845790    -5.194020    -3.263530
Au    -4.283220    -0.232540    -3.949830
Au    -3.611690     1.699900    -1.467270
Au    -6.142290     1.819140    -2.856000
Au    -5.369800    -2.158480    -2.196850
Au    -4.665010    -0.297550     0.125780
Au    -7.271870    -0.123890    -1.137780
Au    -6.567410    -2.404960     0.339560
Au    -3.781010     2.545000    -4.220860
Au    -6.192940     2.293410    -0.038840
Au    -2.544390    -0.888800    -1.514380
Au    -7.407300    -0.418770    -3.922980
Au    -3.259700    -2.770930    -3.848330
```

Au_{13}/fHNT, 2D cluster, ONIOM model system

```
   55
Si     1.273670    -0.292000     1.528200
 O     0.347100    -0.406560     0.192410
 O     0.458850    -0.598280     2.939720
 O     1.827260     1.290960     1.560180
 O     2.541680    -1.365600     1.525060
 H     2.784670     1.357270     1.724150
 H     3.050670    -1.415430     0.706050
Si    -1.248750     3.021130     1.978240
 O    -2.177640     3.178210     0.594310
 O    -1.152680     1.434680     2.407240
 O    -2.109750     3.688290     3.254000
 O     0.254600     3.712190     1.935080
 H    -2.561490     4.520170     3.075300
 H     0.964900     3.043540     1.854500
Si    -3.648560    -0.047690     3.795040
 O    -3.980180    -0.264120     2.164340
 O    -2.065410    -0.553420     3.969540
```

O	-4.668680	-0.980630	4.704590
O	-3.802660	1.462930	4.447860
H	-4.931500	-1.795670	4.265160
H	-3.170020	2.150860	4.180310
Si	-0.729300	0.357490	3.609400
C	-0.101130	1.182340	5.152270
H	-0.966370	1.585480	5.710660
H	0.309510	0.348320	5.754860
C	0.945420	2.253790	4.876180
H	1.731020	1.850780	4.203180
H	0.487710	3.098210	4.331230
C	1.622210	2.818410	6.106320
H	0.877140	3.304540	6.772170
H	2.342390	3.602230	5.799830
N	2.378070	1.805310	6.853310
C	3.098090	2.367460	7.993830
H	1.718410	1.103640	7.202020
C	3.896550	1.310600	8.732900
H	3.780480	3.146410	7.602960
H	2.406120	2.873130	8.697460
H	4.281780	1.749730	9.673540
H	3.220430	0.487140	9.027170
N	4.935590	0.757050	7.875730
H	5.724020	1.399930	7.788480
H	5.308440	-0.117400	8.242870
Au	4.701610	4.119380	1.097650
Au	5.641270	1.434670	1.005710
Au	6.259500	-1.245450	0.678830
Au	6.171400	3.262860	-1.058900
Au	4.391760	2.385160	3.402100
Au	6.802520	0.540990	-1.465850
Au	4.981850	-0.351450	3.062480
Au	7.393650	-2.123730	-1.786270
Au	5.566610	-3.136760	2.682280
Au	6.690990	-3.942870	0.267760
Au	4.295800	-2.242160	4.988900
Au	3.879960	0.482320	5.500090
Au	3.151330	4.850640	3.217430

Au_{13}/fHNT, 3D cluster, ONIOM model system

55			
Si	-0.03782	-2.50922	-2.21069

O	-0.98579	-2.39817	-0.89980
O	0.59578	-1.02910	-2.62739
O	-1.02001	-2.97099	-3.49803
O	1.17455	-3.61366	-2.03568
H	-0.87175	-3.88920	-3.75686
H	2.06889	-3.29787	-2.26025
Si	-3.28157	-0.16803	-3.69103
O	-4.42850	-0.10799	-2.47442
O	-1.80046	0.06372	-3.00218
O	-3.43939	1.20908	-4.63266
O	-3.25319	-1.50777	-4.65033
H	-4.33146	1.43827	-4.91500
H	-2.51054	-2.09988	-4.41996
Si	-0.69415	2.90269	-1.99650
O	-1.50896	2.33498	-0.65338
O	0.26112	1.64320	-2.55358
O	0.31542	4.16732	-1.64922
O	-1.59910	3.47030	-3.26538
H	0.93239	3.99943	-0.92562
H	-2.07288	2.80046	-3.78643
Si	-0.19402	0.25260	-3.35308
C	0.40311	0.18312	-5.10889
H	0.06656	-0.80782	-5.46843
H	1.49355	0.04826	-4.93215
C	0.19150	1.23054	-6.19838
H	0.89862	0.99961	-7.01606
H	-0.81925	1.12079	-6.62910
C	0.34803	2.69390	-5.80929
H	-0.57953	3.06914	-5.33484
H	0.47413	3.28471	-6.73382
N	1.47889	2.98967	-4.92178
C	1.75563	4.42445	-4.83761
H	1.23357	2.66110	-3.98282
C	2.76013	4.85127	-5.89219
H	2.15954	4.63987	-3.83154
H	0.82531	5.01891	-4.93262
H	2.41378	4.52393	-6.88800
H	2.82399	5.95484	-5.92055
N	4.06096	4.22329	-5.64041
H	4.67606	4.29447	-6.45061
H	4.54295	4.69543	-4.87153
Au	3.68196	-0.40934	-3.32079
Au	6.11855	1.03400	-3.65565
Au	3.88449	2.14730	-1.84491
Au	4.28982	-1.88921	-0.88222

Au	7.26575	0.87179	-0.94138
Au	4.77509	0.58249	0.44332
Au	6.70215	-1.53018	0.61875
Au	3.75758	1.99182	-4.71049
Au	5.84986	3.13515	-0.09676
Au	6.82884	-1.75700	-2.26554
Au	2.26538	0.04122	-0.64086
Au	4.57161	-3.08470	-3.38826
Au	8.69863	0.10802	-3.23994

Au_{14}/fHNT, ONIOM model system

56

Si	0.32510	-2.31181	-1.70623
O	-0.65197	-2.15820	-0.41747
O	0.58314	-0.88007	-2.51872
O	-0.46882	-3.29977	-2.82029
O	1.75536	-3.00557	-1.27031
H	-0.16073	-4.21366	-2.77649
H	2.53057	-2.72202	-1.79338
Si	-3.36001	-1.30847	-3.48384
O	-4.56616	-1.30637	-2.32836
O	-2.03821	-0.56441	-2.84006
O	-3.82605	-0.24877	-4.70327
O	-2.94909	-2.75485	-4.15674
H	-4.72894	-0.34142	-5.02674
H	-2.08176	-3.07260	-3.83413
Si	-1.79832	2.58114	-2.67883
O	-2.47072	2.18301	-1.20452
O	-0.47556	1.57469	-2.88804
O	-1.24250	4.14713	-2.67155
O	-2.69908	2.50882	-4.06437
H	-0.92141	4.43577	-1.80989
H	-2.99687	1.63416	-4.37192
Si	-0.55392	-0.02193	-3.37225
C	-0.47986	-0.30715	-5.21422
H	-1.49035	-0.04826	-5.58916
H	-0.44015	-1.41306	-5.26525
C	0.57515	0.28065	-6.15138
H	1.59659	0.14484	-5.74137
H	0.55431	-0.29135	-7.09489
C	0.36481	1.73911	-6.51601
H	-0.67195	1.87827	-6.89751

H	1.04524	2.00594	-7.34498
N	0.61351	2.66027	-5.40873
C	0.33806	4.06023	-5.72326
H	0.05083	2.38466	-4.60254
C	1.55510	4.76295	-6.29717
H	0.02420	4.56084	-4.78966
H	-0.51090	4.15029	-6.42991
H	1.90978	4.23050	-7.19718
H	1.26624	5.78129	-6.62025
N	2.64310	4.75972	-5.32009
H	3.51792	5.08943	-5.72666
H	2.42935	5.38530	-4.54041
Au	6.70478	1.75563	2.11811
Au	6.66259	1.27550	-0.68863
Au	5.23665	1.81710	-3.26990
Au	4.23735	1.56489	0.76662
Au	6.24436	-0.84436	1.19810
Au	2.89861	1.75995	-1.57189
Au	6.81663	-0.70451	-2.82976
Au	2.80347	2.47202	-4.36567
Au	4.66397	-2.51682	-3.34268
Au	3.26124	-0.22078	-3.69974
Au	8.02036	1.79237	-3.05020
Au	4.25303	-0.79447	-0.96752
Au	6.49711	-2.68350	-0.77116
Au	7.35953	-3.44551	-3.31375

Au_{15}/fHNT, ONIOM model system

57

Si	0.811200	-0.383970	0.470080
O	-0.425470	-0.490680	-0.586260
O	0.399340	-0.541950	2.067800
O	1.505800	1.117220	0.228570
O	1.905750	-1.621860	0.175480
H	2.481480	1.101960	0.309500
H	1.984630	-1.846940	-0.760970
Si	-1.275110	3.121750	1.291280
O	-2.508340	3.301730	0.174030
O	-1.185010	1.550480	1.777380
O	-1.745360	3.910740	2.696840
O	0.215180	3.669210	0.840790
H	-2.165680	4.769980	2.580900

H	0.832350	2.937330	0.642580
Si	-3.346160	0.358560	3.841640
O	-4.097840	0.102510	2.364400
O	-1.811070	-0.273700	3.643220
O	-4.162760	-0.440600	5.038400
O	-3.221180	1.904070	4.416050
H	-4.576180	-1.256680	4.739300
H	-2.644720	2.530540	3.946450
Si	-0.545300	0.529980	2.932380
C	0.390330	1.408450	4.280870
H	-0.388050	1.759810	4.987310
H	0.951590	0.606470	4.800750
C	1.306230	2.552100	3.867490
H	2.132160	2.161630	3.233100
H	0.767930	3.281490	3.240640
C	1.893500	3.323250	5.033870
H	1.081080	3.885300	5.546100
H	2.617660	4.070630	4.661040
N	2.597140	2.476610	6.000480
C	3.040900	3.219440	7.174450
H	1.963950	1.731840	6.304670
C	3.856370	2.351000	8.111910
H	3.659780	4.066470	6.820440
H	2.187880	3.663380	7.727820
H	4.031170	2.900320	9.055900
H	3.274120	1.449250	8.373010
N	5.096270	1.914740	7.469650
H	5.774200	2.678580	7.429540
H	5.543740	1.159550	7.988880
Au	7.942150	1.446670	-1.484490
Au	8.389690	-1.225800	-0.534690
Au	5.896050	-0.544450	-1.900530
Au	6.171790	-3.065520	-0.574530
Au	4.845540	-3.496180	1.930490
Au	7.524800	3.450340	0.498960
Au	4.468120	-0.989000	0.588300
Au	5.412810	1.585770	-0.012530
Au	8.113430	0.824690	1.449190
Au	7.093680	-1.747210	1.964690
Au	4.555740	-1.336420	3.725270
Au	6.314320	2.855720	2.967270
Au	6.912740	0.261780	3.940330
Au	4.123910	1.145690	2.399870
Au	4.621700	1.312510	5.135150

Au_{16}/fHNT, ONIOM model system

```
  58
Si    -0.339240    -2.302190    -2.383320
 O    -1.362050    -2.330340    -1.118200
 O     0.356170    -0.798030    -2.614370
 O    -1.261580    -2.628920    -3.749870
 O     0.836890    -3.458260    -2.342990
 H    -0.991010    -3.452540    -4.175230
 H     1.697290    -3.222700    -1.957420
Si    -3.477340     0.198090    -3.655680
 O    -4.752900     0.142450    -2.578310
 O    -2.071390     0.268950    -2.797140
 O    -3.513530     1.702300    -4.410310
 O    -3.399960    -0.978250    -4.806320
 H    -4.356420     1.966040    -4.796290
 H    -2.690090    -1.624890    -4.624190
Si    -1.070550     3.021320    -1.563820
 O    -1.952630     2.330810    -0.327170
 O    -0.039100     1.847100    -2.147680
 O    -0.187530     4.306220    -0.971640
 O    -1.856400     3.687360    -2.867230
 H     0.214420     4.114890    -0.114090
 H    -2.335510     3.079700    -3.462460
Si    -0.463320     0.561910    -3.131000
 C    -0.236950     0.866480    -4.949810
 H    -1.052360     1.538280    -5.283250
 H    -0.493290    -0.115840    -5.389380
 C     1.128170     1.347610    -5.461990
 H     1.933930     1.103010    -4.739860
 H     1.387290     0.799970    -6.382850
 C     1.187740     2.826680    -5.794830
 H     0.453820     3.046810    -6.605350
 H     2.187790     3.066780    -6.203580
 N     0.962310     3.694880    -4.647490
 C     0.861860     5.099220    -5.005760
 H     0.104910     3.422760    -4.158210
 C     0.577580     5.946230    -3.780350
 H     0.066040     5.286160    -5.759870
 H     1.819110     5.403370    -5.473750
 H     0.415440     6.993140    -4.088780
 H    -0.364870     5.596460    -3.325540
 N     1.637420     5.848650    -2.763480
 H     2.227460     6.679310    -2.750310
```

H	1.208080	5.751040	-1.841060
Au	5.599100	3.832650	-3.885400
Au	2.919040	3.856510	-2.874050
Au	7.140790	1.637680	-3.200340
Au	2.539400	1.825530	-0.963610
Au	8.554080	-0.765000	-2.507670
Au	2.080560	-0.460190	0.651250
Au	4.361620	-2.032080	0.404040
Au	6.985210	-2.298550	-0.765490
Au	6.595920	-0.955110	1.754310
Au	4.617250	-3.403440	-2.026580
Au	5.745430	-0.928970	-3.087740
Au	3.169450	-0.850990	-2.043040
Au	4.627350	0.910640	0.749820
Au	7.092860	0.771340	-0.510160
Au	4.135830	1.346910	-3.514830
Au	5.242310	2.889150	-1.187310

Au_{17}/fHNT, ONIOM model system

59

Si	-0.324490	1.111440	0.599280
O	1.146270	1.047510	1.283610
O	-0.501470	0.031130	-0.656450
O	-0.485980	2.633290	-0.120460
O	-1.462450	0.882870	1.767960
H	-0.960600	3.259650	0.442590
H	-2.378520	0.750870	1.459160
Si	2.215030	2.457270	-2.492880
O	3.768930	2.658850	-1.913400
O	1.638250	1.039270	-1.877950
O	2.295170	2.116370	-4.134420
O	1.140240	3.683880	-2.251440
H	2.797480	2.726190	-4.685740
H	0.478910	3.466530	-1.564200
Si	2.379010	-1.759400	-3.144770
O	3.517160	-1.572260	-1.932980
O	0.885760	-1.448690	-2.448890
O	2.372200	-3.325290	-3.694280
O	2.525250	-0.847760	-4.516050
H	2.768290	-3.949250	-3.076730
H	2.326470	0.104610	-4.462100
Si	0.302520	0.081790	-2.119310

C	-0.889810	0.693630	-3.405870
H	-0.825570	1.799550	-3.369570
H	-1.874260	0.471600	-2.946500
C	-0.911760	0.229750	-4.862620
H	-1.871040	0.569780	-5.296240
H	-0.121020	0.744820	-5.435680
C	-0.754850	-1.263420	-5.122150
H	0.316590	-1.541060	-5.120360
H	-1.133360	-1.488500	-6.134420
N	-1.482080	-2.128960	-4.180610
C	-1.276420	-3.542270	-4.518240
H	-1.033750	-2.004530	-3.265470
C	-2.149050	-4.453170	-3.680160
H	-0.208150	-3.815330	-4.401050
H	-1.527080	-3.676000	-5.587650
H	-1.845930	-5.503490	-3.851790
H	-1.981880	-4.241980	-2.607660
N	-3.558900	-4.203540	-3.971320
H	-3.810100	-4.553150	-4.897770
H	-4.175450	-4.667300	-3.302560
Au	-4.953160	0.890210	-3.353860
Au	-6.234670	2.901250	-1.778790
Au	-6.594460	-1.416660	-3.403880
Au	-8.934700	-0.956820	-1.872330
Au	-8.547040	1.642340	-0.718640
Au	-5.152780	-2.534700	-1.101810
Au	-3.577030	-0.193200	-1.075690
Au	-4.687580	1.727130	0.739170
Au	-7.125750	0.579720	1.644780
Au	-7.369090	-2.086830	0.590900
Au	-7.724520	-3.531900	-1.842390
Au	-3.805600	-1.707110	-3.638170
Au	-9.668850	-0.447040	0.868790
Au	-7.016850	3.346560	0.958280
Au	-3.420210	2.641360	-1.632050
Au	-4.787480	-1.078140	1.353240
Au	-7.844650	1.190780	-3.430850

Au_{18}/fHNT, ONIOM model system

60

Si	0.729240	2.336360	-2.120050
O	1.777510	2.375720	-0.883670

O	0.138390	0.800460	-2.325580
O	1.587090	2.706970	-3.519050
O	-0.495100	3.431650	-1.974340
H	1.362510	3.580350	-3.864460
H	-1.394370	3.059900	-1.920800
Si	3.889430	-0.081660	-3.766350
O	5.052010	-0.013930	-2.562600
O	2.452330	-0.440050	-3.024630
O	4.205840	-1.439970	-4.682310
O	3.698380	1.219900	-4.754820
H	5.138560	-1.627770	-4.829460
H	2.969320	1.806620	-4.472540
Si	1.406740	-3.037600	-1.570740
O	2.312090	-2.338640	-0.356230
O	0.416980	-1.872860	-2.250200
O	0.404500	-4.215020	-0.959310
O	2.207960	-3.823730	-2.790900
H	0.211630	-4.141560	-0.017980
H	2.597880	-3.252320	-3.469250
Si	0.802110	-0.487880	-3.136420
C	0.022510	-0.492980	-4.819390
H	-0.072180	0.579260	-5.073110
H	-1.020600	-0.818430	-4.614020
C	0.602490	-1.260040	-6.002310
H	-0.132260	-1.229080	-6.825720
H	1.506820	-0.744350	-6.372140
C	0.978560	-2.703890	-5.725680
H	1.948240	-2.725210	-5.172910
H	1.190070	-3.208910	-6.685410
N	-0.060250	-3.461850	-5.043320
C	0.228390	-4.884320	-5.035780
H	-0.110640	-3.145070	-4.070340
C	-0.644660	-5.647950	-4.067870
H	1.282670	-5.102500	-4.761410
H	0.081710	-5.276980	-6.062710
H	-0.368650	-6.716690	-4.114870
H	-0.458760	-5.308620	-3.032590
N	-2.082650	-5.468050	-4.360410
H	-2.252180	-5.629120	-5.356900
H	-2.629070	-6.172880	-3.862290
Au	-6.045700	-0.199120	2.166780
Au	-8.296760	-0.368800	0.410590
Au	-3.866260	1.431010	1.462560
Au	-4.063370	3.205120	-0.715270
Au	-6.301890	3.023580	-2.465250

Au	-8.274710	1.065750	-1.995290
Au	-6.461000	1.845360	0.159460
Au	-3.813760	-1.337730	0.756270
Au	-2.617830	0.721030	-0.991660
Au	-6.442510	-2.559480	0.587830
Au	-7.335950	-1.627450	-2.001900
Au	-6.122860	0.451070	-3.773160
Au	-3.740440	2.027320	-3.317440
Au	-3.442700	-0.724280	-3.406980
Au	-5.677700	-2.334830	-4.184960
Au	-1.979690	-2.061060	-1.266510
Au	-4.702880	-2.857410	-1.548240
Au	-2.968640	-3.457420	-3.750270

Au_{19}/fHNT, ONIOM model system

61			
Si	-1.168350	-2.577460	-2.309470
O	-2.118950	-2.399220	-1.000440
O	-0.362240	-1.170630	-2.693450
O	-2.180750	-2.919330	-3.609240
O	-0.114280	-3.846160	-2.240960
H	-2.037050	-3.812280	-3.947730
H	0.761840	-3.664850	-1.861470
Si	-4.124230	0.099840	-3.723100
O	-5.340780	0.243000	-2.589390
O	-2.688210	0.114500	-2.913510
O	-4.045720	1.530440	-4.600010
O	-4.191860	-1.174640	-4.765330
H	-4.864110	1.833540	-5.008580
H	-3.541780	-1.868650	-4.538970
Si	-1.372830	2.857870	-1.923570
O	-2.271880	2.347340	-0.613390
O	-0.499160	1.523710	-2.452590
O	-0.304400	4.045530	-1.463580
O	-2.119870	3.497240	-3.255470
H	0.187120	3.814480	-0.660330
H	-2.673890	2.896890	-3.787630
Si	-1.077850	0.196780	-3.304110
C	-0.774450	0.309000	-5.128290
H	-1.372840	1.135880	-5.556210
H	-1.243810	-0.612820	-5.518740
C	0.701610	0.392390	-5.556400

H	1.378130	0.034770	-4.752970
H	0.876940	-0.282490	-6.409760
C	1.129870	1.781500	-5.984390
H	0.491060	2.114380	-6.834150
H	2.169550	1.753050	-6.361220
N	1.084220	2.750530	-4.896580
C	1.296930	4.119990	-5.340210
H	0.185350	2.698480	-4.408910
C	1.189250	5.076680	-4.169730
H	0.568760	4.419720	-6.123580
H	2.303490	4.176020	-5.799250
H	1.260410	6.116090	-4.535480
H	0.190600	4.965970	-3.712490
N	2.190380	4.786150	-3.138020
H	3.008410	5.390370	-3.215860
H	1.789960	4.934110	-2.210330
Au	4.572250	-2.499180	1.007460
Au	2.765440	-4.442440	0.104010
Au	8.106250	1.553220	2.325770
Au	6.318450	-0.490760	1.732500
Au	7.815420	1.056010	-0.403360
Au	7.522900	0.385200	-3.047140
Au	7.083480	-0.336200	-5.717590
Au	5.604180	-1.822130	-3.872280
Au	4.118490	-3.129820	-1.964000
Au	6.150490	-1.236730	-1.075940
Au	2.008940	-1.767030	-0.382360
Au	5.675790	2.425900	1.142910
Au	4.993490	1.072260	-4.493930
Au	3.781510	0.282230	0.599100
Au	3.396410	-0.373900	-2.599740
Au	5.350150	1.898660	-1.733530
Au	2.918830	2.438780	-3.152520
Au	1.479380	0.928320	-0.901200
Au	3.233970	3.049960	-0.100280

Au_{20}/fHNT, ONIOM model system

62

Si	4.401490	0.760870	-4.046270
O	5.356400	0.838100	-2.734270
O	2.803090	0.476110	-3.780240
O	4.884890	-0.569190	-4.951950

O	4.583520	2.162350	-4.905920
H	5.732600	-0.468810	-5.401090
H	3.836000	2.387420	-5.470300
Si	3.521970	-3.122280	-2.598730
O	4.466740	-3.117120	-1.223160
O	2.505080	-1.839980	-2.386860
O	2.564280	-4.503080	-2.572170
O	4.211140	-3.143060	-4.095110
H	2.782920	-5.107450	-3.291560
H	4.420830	-2.276290	-4.500330
Si	0.430550	-0.250190	-0.477210
O	1.891350	-0.206770	0.318210
O	0.749490	0.155050	-2.062900
O	-0.549030	0.853950	0.256980
O	-0.330330	-1.740700	-0.475870
H	-1.466110	0.867040	-0.061590
H	-0.305160	-2.293460	-1.312670
Si	1.699630	-0.617680	-3.177580
C	0.646750	-1.257450	-4.574720
H	1.178920	-2.097000	-5.064970
H	0.641400	-0.442670	-5.319530
C	-0.796980	-1.636840	-4.219510
H	-1.187960	-0.964060	-3.430490
H	-1.445280	-1.459180	-5.095050
C	-1.035610	-3.076550	-3.804850
H	-0.781860	-3.761470	-4.644360
H	-2.120300	-3.199430	-3.622440
N	-0.345980	-3.494450	-2.578070
C	-0.797110	-4.829930	-2.177560
H	0.662880	-3.570230	-2.758110
C	-0.207070	-5.310170	-0.869740
H	-0.561010	-5.578900	-2.963450
H	-1.901460	-4.803770	-2.089300
H	-0.497610	-6.363830	-0.729680
H	0.894920	-5.278990	-0.939910
N	-0.699650	-4.548220	0.291050
H	-0.379020	-4.985980	1.155500
H	-0.306970	-3.598020	0.282660
Au	-4.647660	-1.961530	1.439450
Au	-2.920710	-3.860350	0.290210
Au	-8.015790	2.064170	3.376930
Au	-6.283360	0.022690	2.506290
Au	-6.476660	2.757110	1.161580
Au	-4.968830	3.283720	-1.088200
Au	-3.478980	3.655230	-3.428880

Au	-3.145040	1.157060	-2.230210
Au	-2.987370	-1.321090	-0.996310
Au	-4.628200	0.713780	0.245420
Au	-5.292150	-3.262970	-1.065020
Au	-8.833120	0.872360	0.991360
Au	-5.649830	1.912960	-3.730630
Au	-7.150710	-1.307990	0.076300
Au	-5.334520	-0.743760	-2.555960
Au	-7.311630	1.591250	-1.370210
Au	-7.769840	0.153480	-3.857650
Au	-7.583020	-2.564180	-2.495680
Au	-9.457630	-0.358220	-1.405210
Au	-9.896350	-1.642300	-3.836190
